# A Structural-Reporter Group to Determine the Core Conformation of Sialyl Lewis^x^ Mimetics

**DOI:** 10.3390/molecules28062595

**Published:** 2023-03-13

**Authors:** Beatrice Wagner, Florian P. C. Binder, Xiaohua Jiang, Tobias Mühlethaler, Roland C. Preston, Said Rabbani, Martin Smieško, Oliver Schwardt, Beat Ernst

**Affiliations:** 1 Molecular Pharmacy Group, Pharmacenter, Department Pharmaceutical Sciences, University of Basel, Klingelbergstrasse 50, CH-4056 Basel, Switzerland; 2 Computational Pharmacy Group, Pharmacenter, Department Pharmaceutical Sciences, University of Basel, Klingelbergstrasse 50, CH-4056 Basel, Switzerland

**Keywords:** E-selectin antagonists, sialyl Lewis^x^ mimetics, structural-reporter group, desolvation enthalpy, conformational entropy

## Abstract

The d-Glc*N*Ac moiety in sialyl Lewis^x^ (sLe^x^, **1**) acts predominantly as a linker to position the d-Gal and the l-Fuc moieties in the bioactive spatial orientation. The hypothesis has been made that the NHAc group of Glc*N*Ac pushes the fucose underneath the galactose and, thus, contributes to the stabilization of the bioactive conformation of the core of sLe^x^ (**1**). To test this hypothesis, Glc*N*Ac mimetics consisting of (*R*,*R*)-1,2-cyclohexanediols substituted with alkyl and aryl substituents adjacent to the linking position of the fucose moiety were synthesized. To explore a broad range of extended and spatially demanding R-groups, an enzymatic approach for the synthesis of 3-alkyl/aryl-1,2-cyclohexanediols (**3b-n**) was applied. These cyclohexanediol derivatives were incorporated into the sLe^x^ mimetics **2b-n**. For analyzing the relationship of affinity and core conformation, a ^1^H NMR structural-reporter-group concept was applied. Thus, the chemical shift of H-C5^Fuc^ proved to be a sensitive indicator for the degree of pre-organization of the core of this class of sLe^x^ mimetics and therefore could be used to quantify the contribution of the R-groups.

## 1. Introduction

Lectins, such as selectins [1], galectins [2,3], or siglecs [4,5] have gained increasing attention as drug targets. However, although being valuable leads to the development of new drugs, carbohydrates rarely find therapeutic application, as they typically suffer from complex synthesis and poor pharmacokinetic and pharmacodynamic properties. As a consequence, small molecules mimicking the carbohydrate epitope, e.g., the sialidase inhibitor oseltamivir [6], have been developed to overcome these unfavorable pharmacokinetic and pharmacodynamic properties (PK/PD).

**Figure 1:**
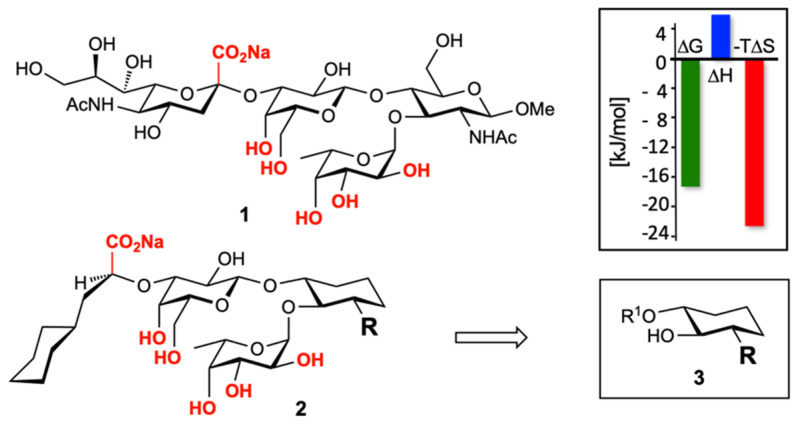
Selectin antagonists; the natural carbohydrate epitope sialyl Lewis^x^ (**1**, sLe^x^) and its thermodynamic fingerprint upon binding to E–selectin, the family of mimetics **2**, and the Glc*N*Ac mimics **3** (R = H, alkyl, aryl). The pharmacophores in **1** and **2** are highlighted in red.

One important reason for the low affinity of carbohydrate/lectin interactions are the high polarity of carbohydrate ligands and, therefore, substantial enthalpic desolvation costs. This becomes obvious from the thermodynamic profile of the sialyl Lewis^x^ (**1**, sLe^x^)/E-selectin interaction (Figure 1) [7]. The positive enthalpy term Δ*H°* is a result of the high desolvation costs (Δ*H°*_desolv_) originating from the numerous hydroxy groups and the carboxylate, which are not compensated by the interaction enthalpy Δ*H°*_int_. As described by Cabani [8], the desolvation of a single hydroxy group costs approx. 26 kJ mol^−1^. This is an amazingly high penalty, considering that it cannot be compensated by a single H-bond which yields a maximum of 20.7 kJ mol^−1^ [9]. However, when hydroxy groups are not involved in binding or are not part of extended H-bond networks, the desolvation enthalpy can be substantially reduced by tdehydroxylation.

It is generally assumed that pre-shaping a ligand into its bioactive conformation will provide increased binding affinity, primarily because the rigidified molecule is expected to benefit from a smaller entropic penalty during complexation. Thus, for example, due to the loss of conformational entropy, one rotatable bond that becomes immobilized upon binding carries a Gibbs energy penalty close to 2 kJ mol^−1^ [10]. The highly beneficial entropy term (−TΔ*S°* = 22.6 kJ mol^−1^) for the interaction of sLe^x^ (**1**)/E-selectin originates from a large desolvation entropy −TΔ*S°*_desolv_ (numerous water molecules are released to bulk), only slightly reduced by a small conformational entropy penalty −TΔ*S°*_conf_, suggesting that tetrasaccharide **1** is pre-shaped in the binding conformation.

According to the pharmacophore of sLe^x^ (**1**) (Figure 1), Neu5Ac contributes only with its carboxylate to binding. However, when Neu5Ac was replaced by glycolic acid, the induced conformational flexibility led to a substantial loss of affinity (IC_50_ 4.5 mM) [11]. To reduce flexibility, a large number of non-carbohydrate acids with substituents in the α-position were evaluated, leading to the discovery of (*S*)-cyclohexyl lactic acid as suitable substitute of Neu5Ac [11,12,13].

The d-Glc*N*Ac moiety acts predominantly as a linker to position the d-Gal and l-Fuc moieties in the bioactive spatial orientation. With the (*R*,*R*)-1,2-cyclohexanediol derivative **2a** (R = H, *K*_D_ 60.7 μM), an expedient replacement of the Glc*N*Ac moiety was identified [10]. When mimic **2b** (R = CH_3,_ *K*_D_ 14 μM) showed a four-fold improved affinity compared to **2a**, the hypothesis was raised that the methyl group of the Glc*N*Ac mimic pushes the fucose underneath the galactose moiety in the same way as the NHAc group in sLe^x^ (**1**) does. Because of the synthetic access via asymmetric CBS (Corey–Bakshi–Shibata) reduction [14,15] and epoxide opening with higher order cyanocuprates [16] was limited to Me, *n*Bu, *c*Pr and CH=CH_2_ [17], we explored an enzymatic approach to explore a broader range of R-groups. For analyzing the relationship of affinity and core conformation, a ^1^H NMR structural-reporter-group concept was applied [18,19]. It was shown on the trisaccharide Lewis^x^, that a non-conventional hydrogen bond between H5 of the fucose moiety (H-C5^Fuc^) and O5 of the galactose moiety (O5^Gal^) results in a low field shift for H-C5^Fuc^ [20,21,22]. This chemical shift proved to be a sensitive indicator for the degree of pre-organization of the core of this new class of sLe^x^ mimetics and, therefore, could be used as structural-reporter group to quantify the contribution of the R-groups.

## 2. Results

Originally, 1,2-cyclohexanediol (**3a**, R = H) was used as GlcNAc mimetic [11,12,13]. To counteract the pushing effect exerted by the NHAc group in sLe^x^, additional alkyl or aryl groups adjacent to the linking position of fucose were introduced. Access to 3-alkyl/aryl-1,2-cyclohexanediols (**3b-n**) was planned by a stereo-selective enzymatic acylation with vinyl butyrate catalyzed by immobilized *Candida antarctica* lipase B (Novozym 435) [23,24]. Since Rotticci et al. [23] successfully applied the stereo-selective enzymatic acylation to 3-methyl-2-cyclohexenol (**3b**), we were confident that the enzymatic approach can be successfully transferred to a wide range of cyclohexenol derivatives.

### 2.1. Synthesis of 3-Alkyl/aryl-2-cyclohexen-1-ols (***3b-m***)

**Scheme 1:**
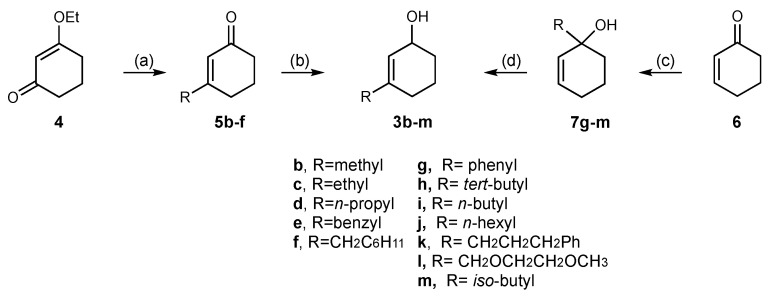
(**a**) RMgBr/Et_2_O, THF, **5b** (comm.), **5c** (89%), **5d** (quant.), or 4-BnO(CH_2_)_4_Br, Li/THF, **5e** (21%), **5f** (comm.); (**b**) NaBH_4_, CeCl_3_·7H_2_O, MeOH, **3b** (comm.), **3c** (96%), **3d** (75%), **3e** (93%), **3f** (quant); (**c**) PhLi, THF, **7g** (88%), **7h** (56%), **7i** (83%), **7j** (75%) or 4,4′-di-*tert*-butylbiphenyl/THF, Li metal, RBr, **7k** (65%), **7l** (37%), **7m** (quant); (**d**) Pd(THF)_2_, CH_3_CN/H_2_O, **3g** (66%), **3h** (82%), **3i** (60%), **3j** (71%); **3k** (73%), **3l** (46%), **3m** (77%); for experimental details see Supporting Information.

When 3-ethoxy-2-cyclohexen-1-one (**4**) was treated with the corresponding Grignard reagents followed by aqueous treatment, the 3-substituted cyclohexenones **5c** and **5d** were obtained. 3-Benzyl-2-cyclohexen-1-one (**5e**) was obtained by addition of 4-benzyloxy-1-bromobutane to a mixture of lithium and 3-ethoxycyclohexenone (**4**) [25]. The cyclohexenone derivatives **5b** and **5f** are commercially available. The required cyclohexenols **3b**-**3e** were obtained by the reduction of **5b-f** with NaBH_4_ in the presence of CeCl_3_·7H_2_O (Scheme 1) [26].

For the 3-substituted cyclohex-2-enols **3g**-**3m** an alternative approach was explored, which entails a 1,2-addition of organometallic reagents to cyclohex-2-enone to form the allylic alcohols **7g**-**7m**, followed by Pd(TFA)_2_ catalyzed 1,3-isomerization to yield **3g**-**3m** (Scheme 1) [27].

**Scheme 2:**
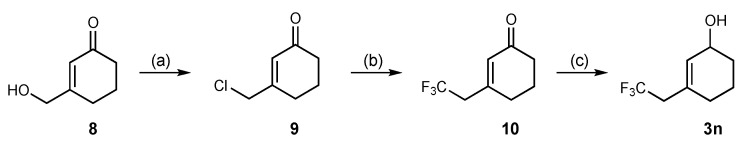
(**a**) 1-Chloro-*N*,*N*,2-trimethylpropenylamine, CH_2_Cl_2_, 0 °C (99%); (**b**) *N*-methyl-2-pyrrolidon, CuI, KF, FSO_2_CF_2_CO_2_Me, 70 °C, overnight (62%); (**c**) NaBH_4_, CeCl_3_·7H_2_O, Et_2_O/EtOH (92%); for experimental details see Supporting Information.

For the synthesis of 3-(2,2,2-trifluoroethyl)-2-cyclohexen-1-ol (**3n**), commercial 3-(hydroxymethyl)-2-cyclohexen-1-one (**8**) was treated with 1-chloro-*N*,*N*,2-trimethylpropenylamine [28] to give the corresponding chloride **9** under neutral conditions in excellent yield. With methyl fluorosulfonyldifluoroacetate in the presence of CuI, the chloride in **9** was replaced by a trifluoromethyl group (→**10**) [29]. Reduction with NaBH_4_ in the presence of CeCl_3_·7H_2_O yielded the allyl alcohol **3n** (Scheme 2) [26].

### 2.2. Enzyme-Catalyzed Kinetic Resolution of Rac-3-alkyl/aryl-2-cyclohexen-1-ol (***3b-n***)

**Scheme 3:**
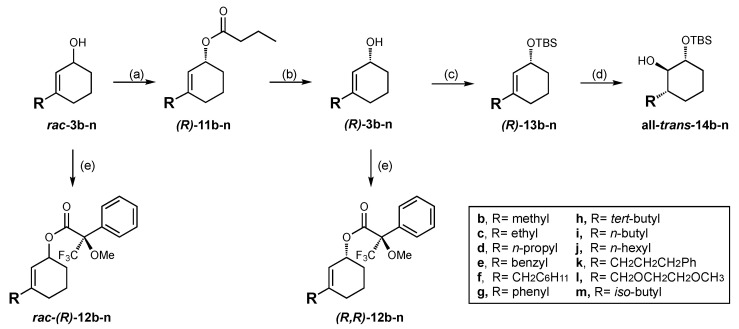
(**a**) Novozyme 435, vinylbutyrate, *n*-heptane, 23 °C, 200 rpm, 2 h 25 min, (17–46%); (**b**) aq. NaOH, MeOH, 0 °C, 5 h; (**c**) TBSCl, imidazole, DMAP, CH_2_Cl_2_, rt, 15 h; (**d**) i. BH_3_·THF, THF, 0 °C to rt, 2 h; ii. H_2_O_2_, aq. NaOH, 0 °C to rt (50%—quant.); (**e**) (*R*)-(-)-α-methoxy-α-(trifluoromethyl)phenylacetyl chloride, DMAP, CH_2_Cl_2_, 5 min, 0 °C, then 1 h, rt, ^19^F NMR were performed without purification of the Mosher derivatives; for experimental details see Supporting Information.

**Figure 2:**
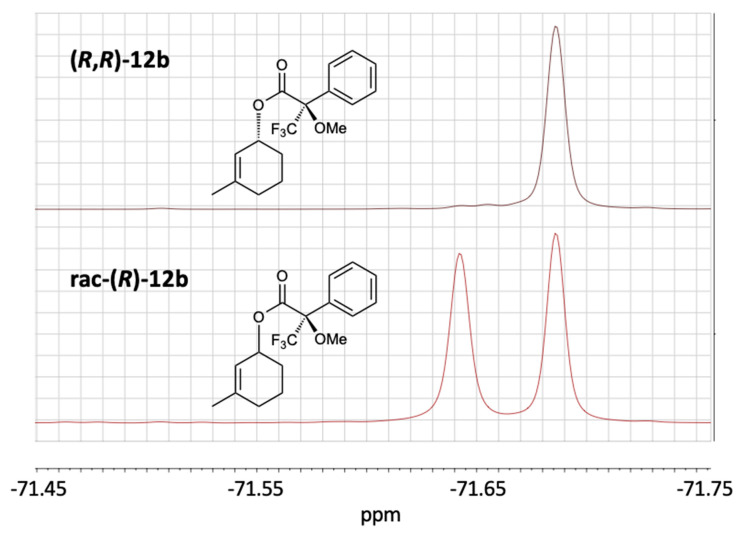
^19^F NMR spectra of Mosher esters **(*R,R*)-12b** and ***rac*,(*R*)-12b** obtained from ***rac*-3b** and (***R*)-3b**. for the ^19^F NMR spectra of Mosher esters **(*R,R*)-12c-n** see Table 1 and Supporting Information.

Starting from commercially available racemic seudenol (3-methyl-2-cyclohexen-1-ol, **3b**), the stereo-selective enzymatic acylation (→(***R*)-11b**) with vinyl butyrate catalyzed by immobilized *Candida antarctica* lipase B (Novozym 435) was described by Rotticci [23] and Ter Halle [24]. Using optimized conditions for the enzymatic reaction [24], we could isolate (*R*)-seudenolester ((***R)*-11b**) in 46% yield in up to 10 g scale. Subsequent saponification afforded (*R*)-3-methyl-2-cyclohexen-1-ol (**(*R*)-3b**), which was used without purification in the next step. By a similar approach, the 3-alkyl/arylcyclohex-2-en-1-ols (**(*R)*-3c-n**) were obtained (Scheme 3) with ee’s of 85% to 98% (Figure 2 and Table 1).

^19^F is an important nucleus in NMR spectroscopy because of its receptivity and large chemical shift dispersion. ^19^F NMR spectroscopy is therefore perfectly suited to determine the enantiomeric excess (ee) based on a comparison of the Mosher esters of ***rac*-3b** and (***R)*-3b**. The diastereomeric excess (de) for the Mosher ester (***R*,*R)*-12b** of ***R*-3b** amounted to 97%. For the Mosher esters **12c-n,** excellent de’s resulting in ee’s for **3c-n** in the range of 85 to 98% were obtained as well.

Since the protecting group of the hydroxy group in **(*R*)-3b-n** has to be stable under strongly basic and acidic conditions, should not hamper fucosylation by steric bulk, and finally, should allow cleavage under mild conditions orthogonal to benzyl protecting groups, a *tert*-butyldimethylsilyl (TBS) ether (→**(*R*)-13b-n**) was chosen. Hydroboration followed by oxidation yielded all-*trans*-**14b-n** in 50–92% over two steps. By this short sequence all-*trans*-**14b-n** were obtained in acceptable to excellent yields, requiring only two chromatographic purifications.

### 2.3. Synthesis of the Mimetics ***2b-n*** of Sialyl Lewis^x^ (**1**)

**Scheme 4:**
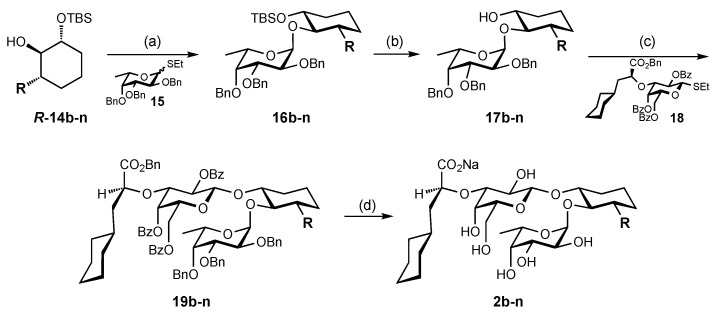
(**a**) CuBr_2_, DTBMP, TBAB, CH_2_Cl_2_, DMF, MS 4 Å, rt, 10 h; (**b**) TBAF, THF, rt, 20 h; (**c**) DMTST, MS 3Å, CH_2_Cl_2_, rt, 43 h; (**d**) i. Pd/C, H_2_, EtOH, cat. AcOH, rt, ii. LiOH, MeOH/H_2_O, rt, 2 d, iii. Dowex (Na^+^), Sephadex-G15; for experimental details see Supporting Information.

Fucosylation of all-*trans*-**14b** under in situ anomerization conditions [30] gave **16b** (Scheme 4), which was smoothly deprotected with tetrabutylammonium fluoride (TBAF), affording pseudodisaccharide **17b** [17] in excellent yield over two steps. Galactosylation with donor **18** [31] promoted by dimethyl(methylthio)sulfonium triflate (DMTST) afforded **19b** β-selectively. Debenzylation by hydrogenolysis followed by saponification with lithium hydroxide and ion exchange chromatography finally gave **2b**. In a similar approach, the test compounds **2c-n** were obtained.

### 2.4. Structural-Reporter Group for the Core Conformation

The ^1^H chemical shift of H-C5^Fuc^ was monitored as a structural-reporter group for the strength of the non-conventional H-bond between H-C5^Fuc^ and O5^Gal^. This chemical shift is highly sensitive to the frequency and distance of the non-conventional H-bond interaction and thus represents a population-weighted average of all conformations present in the solution (Table 2).

## 3. Discussion

The chemical shift of H-C5^Fuc^ of sLe^x^ (**1**) is 4.83 ppm [32] and thus 0.9 ppm higher than that of the fucose monomer **20** [33] (Table 2). When Neu5Ac in sLe^x^ (**1**) was replaced by (*S*)-cyclohexyl lactic acid (→**22**) [11,12,13,17], the chemical shift of H-C5^Fuc^ remains unchanged, i.e., the replacement has no influence on the core conformation. However, when the Glc*N*Ac moiety was replaced by (*R*,*R*)-cyclohexane-1,2-diol (→**2a**) [17], a substantially high field shift (Δδ = 0.33) occurred, indicating that compared to sLe^x^ (**1**) the pre-organization of the core conformation is disturbed. Nevertheless, the affinity was improved five-fold, although the chemical shift of H-C5^Fuc^ of 4.50 ppm in **2a** is a clear indication for raised conformational entropy costs. This penalty, however, is obviously overcompensated by lower enthalpic desolvation costs originating from a reduction of the polar surface area (PSA) [34] due to the replacement of Glc*N*Ac by (*R*,*R*)-cyclohexane-1,2-diol (PSA**_22_**= 324 Å^2^ vs. PSA**_2a_**= 205 Å^2^).

**Table 2:** The affinity of the E-selectin antagonists **2a-n** was assessed by microscale thermophoresis (MST) measurements. Chemical shifts from ^1^H NMR experiments. Affinities and ^1^H NMR chemical shift for **1**, **2a**, **20–22** are literature data [7,17,32,33,35]. Synthesis and assay procedures are described in the Supplementary Information.

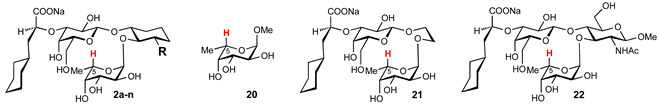									
**Comp.**	**R**	***K*_D_** **[μM]**		**H-C5^Fuc^** **δ [ppm]**	**Comp.**	**R**		***K*_D_** **[μM]**	**H-C5^Fuc^** **δ [ppm]**
**2a**	H	60.7		4.50 [17]	**2h**	*t*-Bu		51	4.03
**2b**	Me	17.8		4.84 [17]	**2i**	*n*-Bu		5.4	4.86
**2c**	Et	9.5		4.84 [17]	**2j**	*n*-Hex		4.3	4.83
**2d**	*i*-Pr	6.8		4.85	**2k**	(CH_2_)_3_Ph		28	4.75
**2e**	Benzyl	18		4.87	**2l**	CH_2_O(CH_2_)_2_OMe		31	4.84
**2f**	CH_2_C_6_H_11_	14		4.83	**2m**	*i*-Bu		6	4.85
**2g**	Phenyl	8.8		4.75	**2n**	CH_2_CF_3_		9.5	4.61
**Reference compounds**									
**Comp.**			***K*_D_ [μM]**				**H-C5^Fuc^** **δ [ppm]**		
**20**							3.93 [33]		
**21**			4922				4.12 [23]		
**22**			280 [11,12,13]				4.83 [17]		
sLe^x^ (**1**)			877 [7]				4.83 [32]		

The transition from (*R*,*R*)-cyclohexane-1,2-diol in **2a** to (*R,R,S*)-3-methylcyclohexane-1,2-diol in **2b** caused a 3.5-fold improvement of *K*_D_. Whereas binding and desolvation enthalpy should remain unchanged (almost identical water shell, same PSA and same pharmacophore), the improved pre-organization of the core in the bioactive conformation (H-C5^Fuc^ increases from 4.50 ppm for **2a** to 4.84 ppm for **2b**) is obviously responsible for the improved *K*_D_ of **2b**.

As reported earlier [17], the additional methyl group in **2b** pushes the Fuc moiety—as the NHAc group in sLe^x^ (**1**)—underneath the Gal moiety, leading to comparable pre-organization of the core as indicated by the same chemical shift for H-C5^Fuc^. Linear R-groups as in **2b-d,i,j,m** lead to chemical shifts of H-C5^Fuc^ similar to sLe^x^ (**1**). They obviously support the bioactive core conformation, resulting in single-digit micromolar binding affinities. However, with a bulkier R-group as in **2h** (R = *t*-Bu) a high-field chemical shift of H-C5^Fuc^ accompanied by a loss in affinity was observed. Finally, although **2e** (R = benzyl), **2f** (R = CH_2_C_6_H_11_), and **2l** (R = CH_2_O(CH_2_)_2_OMe) exhibit chemical shifts for H-C5^Fuc^ characteristic for an optimal core conformation, they show slightly lower affinities. According to saturation transfer difference (STD)-NMR, hydrophobic contacts of the methyl group in **2b** can be excluded [17]. This was also confirmed by the X-ray structure of **2b** (R = Me) co-crystallized with E-selectin (PDB 4C16), where both Arg84 and Gln85 as parts of the E-selectin binding site have thermal B-factors of ~74 (at carbon atom CZ) and ~78 (at carbon atom CD), respectively, which is a clear sign for a high flexibility of their amino acid side chains compared to the backbone (B-factors of ~45 at CA). However, bulky R-groups can establish van der Waals contacts with Arg84 and Gln85. This limits the flexibility of the amino acid side chains causing an entropy penalty and reduced affinities. In most pronounced cases a steric clash may also disrupt the optimal core conformation or prevent from proper access to the binding site as observed for **2g** (R = phenyl) and **2k** (R = (CH_2_)_3_Ph). In the case of **2l** (R = CH_2_O(CH_2_)_2_OMe), an additional desolvation penalty for the rather polar R-group may be involved as well. Surprisingly, although virtually the same *K*_D_’s were obtained for **2c** (R = CH_2_CH_3_) and **2n** (R = CH_2_CF_3_), there is a large difference between the chemical shifts of H-C5^Fuc^ (Δδ = 0.23) for the two compounds.

Based on the binding affinities *K*_D_, the Gibbs free energies Δ*G* were calculated (Table 3). Because in **21**, which contains a flexible ethanediol as Glc*N*Ac replacement, the chemical shift for H-C5^Fuc^ is close to that of the Fuc monosaccharide **20** (4.12 and 3.93 ppm, respectively, Table 2), a contribution related to a direct neighborhood of H-C5^Fuc^ and O5^Gal^ can therefore be excluded. The 80-fold improvement of affinity for **2a** compared to **21** is related to reduced entropy costs, predominantly as a consequence of the reduced flexibility of the linker. All other thermodynamic parameters defining affinity (similar pharmacophore and desolvation costs) remain approximately the same size. Thus, the ΔΔ*G***_21_**_→_**_2a_** of 10.89 kJ mol^−1^ is, if at all, related to the formation of the non-conventional H-bond but mainly to the rigidified linker. Finally, the introduction of an R group further limits the flexibility of the core by pushing the Fuc moiety underneath the Gal unit enabling the formation of a non-conventional H-bond between H-C5^Fuc^ and O5^Gal^. Thus, ΔΔ*G***_2a_**_→_**_2b_** of 3.06 kJ mol^−1^ and ΔΔ*G***_2a_**_→_**_2j_** of 6.57 kJ mol^−1^ result from the steric effect of the R-group as well as from the formation of a non-conventional H-bond.

## 4. Conclusions

A common strategy for the design of glycomimetics is the substitution of carbohydrate moieties by carbocyclic scaffolds. It results in lower polarity, i.e., reduced desolvation costs but also increased hydrolytic and metabolic stability. In addition, the facile synthetic accessibility of the mimetic structures, which, as a consequence of the chirality of the parent carbohydrate moiety includes stereochemical challenges, is of cardinal importance.

We developed a fast and efficient approach to (*R*)-3-alkyl/aryl-cyclohex-2-en-1-ol (**(*R*)-3b-n**) starting from the racemic precursors ***ra*c-3b-n** by stereo-selective enzymatic acylation catalyzed by immobilized *Candida antarctica* lipase B (Novozym 435) [23,24]. By subsequent hydroboration, fucosylation, and galactosylation, a novel class of selectin antagonists could efficiently be explored.

For evaluating the core conformation of the sLe^x^ mimetics **(*R*)-2a-n**, the chemical shift of H-C5^Fuc^ proved to be a valuable structural-reporter group [18,19]. When mimetics exhibit a chemical shift for H-C5^Fuc^ between 4.83 and 4.86 ppm, their core conformation is comparable to the natural counterpart sLe^x^ (**1**) [21,32]. In contrast, high-field shifts indicate a disturbed pre-organization of the core, leading to an increase in conformational entropy costs. Examples with pre-shaped cores are **2c**,**d**,**i**,**j**,**m** exhibiting affinities in the range of 4.3–9.5 μM. On the other hand, when the R-group is increasingly space-demanding as in **2h** and **2k**, the optimal core conformation is disrupted (H-C5^Fuc^ 4.03, 4.75 ppm) or optimal access of the ligand to the binding site is hindered, consequently leading to a substantial loss of affinity.

In summary, H-C5^Fuc^ proved to be a reliable reporter group for evaluating to what extent the core of the sLe^x^ mimetics **2b**-**2n** is pre-organized in the bioactive conformation. The correlation is, therefore, a qualitative tool to quickly predict the efficacy of a derivative as an E-selectin binder. Furthermore, the improvement of the Gibbs free energy Δ*G*° by > 6.5 kJ mol^−1^, i.e., a 10-fold improvement of affinity, impressively shows the relevance of the contribution by the pre-shaped core and the non-conventional H-bond to binding.

## 5. Materials and Methods

E-selectin production (cloning, transfection, expression, and purification) and labeling were previously described for the E-selectinSCR6-IgGFc construct [12] and for the E-selectinSCR2 construct [36].

E-selectinLEC2 was labeled using the amine reactive protein labeling kit BLUE-NHS as described in the Supporting Information. Buffer exchange and labeling were performed according to the manufacturer’s protocol. To protect the lysines in the binding site from being labeled, the protein was saturated with 600 µM of compound **2b**. The labeled protein was dialyzed over night against assay buffer (10 mM HEPES pH 7.4, 150 mM NaCl, 1 mM CaCl_2_) using Slide-A-Lyzer dialysis cassettes (10 kDa MWCO). Protein concentration was determined by HPLC-UV against a BSA standard.

Microscale thermophoresis (MST) experiments were carried out at 25 °C with 100% LED power, 50% laser power, a laser on time of 30 s, and a laser off time of 5 sec using standard treated capillaries. Ligands were dissolved in assay buffer supplemented with 0.05% *v*/*v* Tween20 and titrated 1:1 for a total of 16 dilution steps. The dilution series of ligand was mixed 1:1 with a solution of 0.2 μM labeled E-SelectinSCR2 and incubated for 10 min at room temperature before measurement. Datapoints were normalized using the bound and unbound borders achieved by NanoTemper Analysis 1.2.205 software (NanoTemper Technologies GmbH, Munich, Germany) and analyzed/illustrated with GraphPad Prism 5.0 (GraphPad Software, La Jolla, CA, USA). The measurements were globally fitted using Equation (1) for single site binding [37].

(1) $$\left[\mathrm{PL}\right]=\frac{{(C}_{P}{+C}_{L}{+K}_{D})\;-\;\sqrt{{(C}_{P}{+C}_{L}{+K}_{D}{)\;-\;4C}_{P}C_{L}}}{{2C}_{P}}$$

where [PL] is the protein–ligand complex concentration and *K*_D_ is the dissociation constant. C_P_ represents the total concentration of protein and C_L_ the total concentration of ligand.

## Figures and Tables

**Table 1:** Diastereomeric excess (de) for Mosher derivatives **(*R*,*R*)-12b-n**.

Comp.	R	de [%]	Comp.	R	de [%]
**12b**	Me	97	**12i**	*n*-Bu	90
**12c**	Et	92	**12j**	*n*-Hex	94
**12d**	*i*-Pr	85	**12k**	(CH_2_)_3_Ph	98
**12e**	Benzyl	97	**12l**	CH_2_O(CH_2_)_2_OMe	98
**12f**	CH_2_C_6_H_11_	97	**12m**	*i*-Bu	96
**12g**	Phenyl	97	**12n**	CH_2_CF_3_	96
**12h**	*t*-Bu	89			

**Table 3:** *K*_D_ and the calculated Gibbs free energies Δ*G*= -RT ln*K*_D_.

Comp.	*K*_D_ [μM]	H-C5^Fuc^ δ [ppm]	Δ*G* [kJ mol^−1^] (Gibbs Free Energy)
**21**	4922	4.12	−13.17
** 2a **	60.7	4.50	−24.06
** 2b **	17.8	4.84	−27.12
** 2j **	4.3	4.83	−30.63

## Data Availability

Not applicable.

## Supplementary Materials

The following supporting information can be downloaded at: https://www.mdpi.com/article/10.3390/molecules28062595/s1. Syntheses and analytical characterizations of test compounds **2b**-**2n** and Mosher esters ***rac*,(*R*)-12b-n** and **(*R*,*R*)-12b-n** including ^19^F NMR of the Mosher derivatives of **3b**-**3n**, ^1^H and ^13^C NMR spectra and HPLC traces of all test compounds **2b**-**2n**, labeling protocol of E-selectinSCR2, description of the microscale thermophoresis assay. Scheme 1: Synthesis of the cyclohex-2-en-1-one derivatives 3**b-f**; Scheme 2: Synthesis of the cyclohex-2-en-1-one derivatives 3**g-m**; Scheme 3: Synthesis of 3-(2,2,2-trifluoroethyl)cyclohex-2-en-1-ol (3**n**). Scheme 4: GlcNAc mimetics (*R*)-14**b-n**. Scheme 5: Synthesis of selectin antagonists 2**b-n**.

## References

[1] Ernst B., Magnani J.L. (2009). From carbohydrate leads to glycomimetic drugs. Nat. Rev. Drug Discov..

[2] Zetterberg F.R., MacKinnon A., Brimert T., Gravelle L., Johnsson R.E., Kahl-Knutson B., Leffler H., Nilsson U.J., Pedersen A., Peterson K. (2022). Discovery and optimization of the first highly effective and orally available galectin-3 inhibitor for the treatment of fibrotic disease. J. Med. Chem..

[3] Slack R.J., Mills R., Mackinnon A.C. (2021). The therapeutic potential of galectin-3 inhibition in fibrotic disease. Int. J. Biochem. Cell Biol..

[4] Lim J., Sari-Ak D., Bagga T. (2021). Siglecs as therapeutic targets in cancer. Biology.

[5] Crocker P.R., Paulson J.C., Varki A. (2007). Siglecs and their roles in the immune system. Nat. Rev. Immunol..

[6] Kim C.U., Lew W., Williams M.A., Liu H.T., Zhang L.J., Swaminathan S., Bischofberger N., Chen M.S., Mendel D.B., Tai C.Y. (1997). Influenza neuraminidase inhibitors possessing a novel hydrophobic interaction in the enzyme active site. J. Am. Chem. Soc..

[7] Binder F.P.C., Lemme K., Preston R.C., Ernst B. (2012). Sialyl Lewis^x^: A “pre-organized water oligomer”. Angew. Chem. Int. Ed..

[8] Cabani S., Gianni P., Mollica V., Lepori L. (1981). Group contributions to the thermodynamic properties of non-ionic organic solutes in dilute aqueous solution. J. Solution Chem..

[9] Vedani A., Huhta D.W. (1990). A new force field for modeling metalloproteins. J. Am. Chem. Soc..

[10] D’Aquino J.A., Freire E., Amzel L.M. (2000). Binding of small organic molecules to macromolecular targets: Evaluation of conformational entropy charges. Proteins.

[11] Kolb H.C., Ernst B. (1997). Development of tools for the design of selectin antagonists. Chem. Eur. J..

[12] Jahnke W., Kolb H.C., Blommers M.J.J., Magnani J.L., Ernst B. (1997). Comparison of the bioactive conformation of sialyl Lewis^x^ and a potent sialyl Lewis^x^ mimic. Angew. Chem. Int. Ed. Engl..

[13] Norman K.E., Anderson G.P., Kolb H.C., Ley K., Ernst B. (1998). Sialyl Lewis^x^ (sLe^x^) and an sLe^x^ mimetic, CGP69669A, disrupt E-selectin-dependent leukocyte rolling in vivo. Blood.

[14] Holub N., Neidhöfer J., Blechert S. (2005). The total synthesis of (+)-*trans*-195A. Org. Lett..

[15] Corey E.J., Helal C.J. (1998). Reduction of carbonyl compounds with chiral oxazaborolidine catalysts: A new paradigm for enantioselective catalysis and a powerful new synthetic method. Angew. Chem. Int. Ed..

[16] Alexakis A., Jachiet D., Normand J.F. (1986). Boron fluoride promoted opening of epoxides by organocopper and cuprate reagents. Tetrahedron.

[17] Schwizer D., Patton J.T., Cutting B., Smieško M., Wagner B., Kato A., Weckerle C., Binder F.P., Rabbani S., Schwardt O. (2012). Pre-organization of the core structure of E-selectin antagonists. Chem. Eur. J..

[18] Van Leeuwen S.S., Leeflang B.R., Gerwig G.J., Kamerling J.P. (2008). Development of a ^1^H NMR structural-reporter group concept for the primary structural characterization of α-d-glucans. Carbohydr. Res..

[19] Spik G., Debruyne V., Montreuil J., van Halbeck H., Vliegenthart J.F.G. (1985). Primary structures of two sialylated triantennary glycans from human serotransferrin. FEBS.

[20] Zierke M., Smieško M., Rabbani S., Aeschbacher T., Cutting B., Allain F.H.-T., Schubert M., Ernst B. (2013). Stabilization of branched oligosaccharides: Lewis^x^ benefits from a non-conventional C-H•••O hydrogen bond. J. Am. Chem. Soc..

[21] Aeschbacher T., Zierke M., Smieško M., Collot M., Mallet J.-M., Ernst B., Allain F.H.-T., Schubert M. (2017). A secondary structural element in a wide range of fucosylated glycoepitopes. Chem. Eur. J..

[22] Battistell M.D., Azurmendi H.F., Frank M., Freedberg D.I. (2015). Uncovering nonconventional and conventional hydrogen bonds in oligosaccharides through NMR experiments and molecular modeling: Application to sialyl Lewis^x^. J. Am. Chem. Soc..

[23] Rotticci D., Norin T., Hult K. (2000). Mass transport limitation reduce the effective stereospecificity in enzyme-catalyzed kinetic resolution. Org. Lett..

[24] Ter Halle R., Bernet Y., Billard S., Bufferne C., Carlier P., Delaitre C., Flouzat C., Humbolt G., Laigle J.C., Lombard F. (2004). Development of a practical multikilogram production of (*R*)-seudenol by enzymatic resolution. Org. Process Res. Dev..

[25] Barnier J.-P., Morisson V., Volle J., Blanco L. (1999). Chemo-enzymatic preparation of optically active endo-bicyclo [4.1.0]heptan-2-ols. Tetrahedron Asymmetry.

[26] Luche J.-L. (1978). Lanthanides in organic chemistry. 1. Selective 1,2 reduction of conjugated ketones. J. Am. Chem. Soc..

[27] Li J., Tan C., Gong J., Yang Z. (2014). Palladium-catalyzed oxidative rearrangement of tertiary allylic alcohols to enones with oxygen in aqueous solvent. Org. Lett..

[28] Munyemana F., Frique-Hesbain A.-M., Devos A., Ghosez L. (1989). Synthesis of alkyl halides under neutral conditions. Tetrahedron Lett..

[29] Chen Q.-Y., Wu S.-W. (1989). Methyl fluorosulphonyldifluoroacetate; a new trifluoromethylating agent. J. Chem. Soc. Chem. Commun..

[30] Sato S., Mori M., Ito Y., Ogawa T. (1986). An efficient approach to *O*-glycosides through CuBr_2_—Bu_4_NBr mediated activation of glycosides. Carbohydr. Res..

[31] Ernst B., Wagner B., Baisch G., Katopodis A., Winkler T., Öhrlein R. (2000). Substrate specificity of fucosyl transferase III: An efficient synthesis of sialyl Lewis^x^-, sialyl Lewis^a^-derivatives and mimetics thereof. Can. J. Chem..

[32] Ball G.E., O’Neill R.A., Schultz J.E., Lowe J.B., Weston B.W., Nagy J.O., Brown E.G., Hobbs C.J., Bednarski M.D. (1992). Synthesis and structural analysis using 2-D NMR of sialyl Lewis^x^ (sLe^x^) and Lewis^x^ (Le^x^) oligosaccharides: Ligands related to E-selectin [ELAM-1] binding. J. Am. Chem. Soc..

[33] Zehavi U., Sharon N. (1972). The synthesis of methyl 2,4-diacetamido-2,4,6-trideoxy hexapyranosides. J. Org. Chem..

[34] Ertl P., Rohde B., Selzer P. (2000). Fast calculation of molecular polar surface area as a sum of fragment-based contributions and its application to the prediction of drug transport properties. J. Med. Chem..

[35] Thoma G., Magnani J.L., Patton J.T., Ernst B., Jahnke W. (2001). Preorganization of the bioactive conformation of sialyl Lewis^x^ analogues correlates with their affinity to E-selectin. Angew. Chem. Int. Ed..

[36] Preston R.C., Jakob R.J., Binder F.P.C., Sager C.P., Ernst B., Maier T. (2016). E-selectin ligand complexes adopt an extended high-affinity conformation. J. Mol. Cell. Biol..

[37] Cooper A., Humphrey S., Macpherson J., Taylor P. (2004). Thermodynamics and Interaction. RSC Tutorial Chemistry Text No. 16. Biophysical Chemistry.

