# Supplementary material for: A Structural-Reporter Group to Determine the Core Conformation of Sialyl Lewisx Mimetics
Source: Molecules. 2023 Mar 13;28(6):2595. doi: 10.3390/molecules28062595 (PMC10054758; doi:10.3390/molecules28062595)
Supplement: Supplementary file 1 [file molecules-28-02595-s001.zip › molecules-2192572-supplementary.pdf]

Supporting Information

Content

1. Synthesis of Glc*N*Ac mimetics 2
2. Labeling of E-selectinSCR2 39
3. Microscale thermophoresis 39
4. ^{19}F NMR Mosher derivatives 40
5. Determination of chemical shift of H-C5^{Fuc} in case of superposition with the DHO peak 47
6. ^{1}H and ^{13}C NMR spectra 48
7. HPLC traces 59
8. References 63

1. Synthesis of Glc*N*Ac mimetics

General Methods. NMR spectra were recorded on a Bruker Avance DMX-500 (500 MHz) spectrometer. Assignment of ^{1}H and ^{13}C NMR spectra was achieved using 2D methods (COSY, HSQC, HMBC). Chemical shifts are expressed in ppm using residual solvent signals ($CHCl_3$, CHD_2OD, HDO) as reference. Optical rotations were measured with a PerkinElmer polarimeter 341. Electrospray ionization mass spectrometry (ESI-MS) data were obtained on a Waters Micromass ZQ instrument. High resolution mass (HR-MS) analyses were done on an Agilent 1100 LC, equipped with a photodiode array detector and a Bruker QTOF I, equipped with a 4 GHz digital-time converter. Reactions were monitored by TLC using glass plates coated with silica gel 60 F254 (Merck) and visualized by using UV light and/or by charring with a molybdate solution (a 0.02 M solution of ammonium cerium sulfate dihydrate and ammonium molybdate tetrahydrate in 10% aq. H_2SO_4). Flash chromatography was done on a CombiFlash R_f from Teledyne Isco equipped with RediSep normal phase or RP-18 reversed-phase flash columns. Size exclusion chromatography was performed on Biogel P-2 media (Bio-Rad Laboratories, Inc.). Reagents were purchased as reagent grade from Fluka, Sigma-Aldrich (Buchs, Switzerland) and Acros Organics (Geel, Belgium), and used without further purification. Solvents were purchased from Sigma-Aldrich or Acros and were dried prior to use where indicated. MeOH was dried by reflux with sodium methoxide and distilled and stored under argon atmosphere. Dichloromethane (DCM), acetonitrile (MeCN) and toluene were dried by filtration over Al_2O_3 (Fluka, type 5016 A basic) and stored over activated molecular sieves (3Å, 4Å). Pyridine was distilled over KOH under argon atmosphere and stored over KOH pellets. Dry DMF was purchased from Acros Organics. Molecular sieves (3Å, 4Å) were activated under vacuum at 500 °C for 0.5 h immediately before use.

Synthesis of cyclohexanones 3b-f

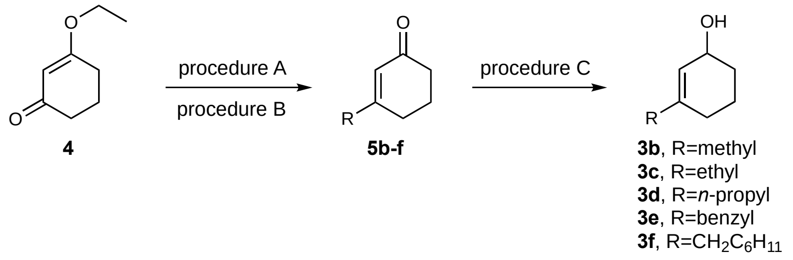

Scheme 1. Synthesis of the cyclohex-2-en-1-one derivatives **3b-f**

3-Methylcyclohex-2-en-1-one (5b). Commercially available.

General procedure A:[1]

3-Ethylcyclohex-2-en-1-one (5c). To a stirred solution of ethyl magnesium bromide (3 M in diethyl ether, 2.9 mL, 8.56 mmol) under argon a mixture of 3-ethoxycyclohex-2-en-1-one (**4**) (1.00 g, 7.13 mmol) in dry THF (8 mL) was added dropwise at 10 °C. The resulting reaction mixture was stirred at rt for 3 h prior to quenching by dropwise addition of 3 M aq. HCl (12.7 mL) at 0 °C. After stirring at 0 °C for 3 h the mixture was extracted

with diethyl ether (2 x 30 mL). The combined organic layers were washed with satd. aq. $NaHCO_3$ and brine, dried over Na_2SO_4 and evaporated (40 °C, 200 mbar). Column chromatography on silica gel (petroleum ether/diethyl ether, 3:1) yielded **5c** (802 mg, 89%). ^{1}H NMR (500 MHz, $CDCl_3$): δ = 1.10 (t, J = 7.5 Hz, 3H, CH_3), 1.99 (p, J = 6.3 Hz, 2H, H-5), 2.24 (qd, J = 7.4, 1.3 Hz, 2H, CH_2), 2.29 (td, J = 5.8, 1.2 Hz, 2H, H-4), 2.33-2.42 (m, 2H, H-6), 5.88 (p, J = 1.5 Hz, 1H, H-2).

3-Propylcyclohex-2-en-1-one (5d). According to general procedure A, propyl magnesium bromide (2 M in diethyl ether, 12.8 mL, 25 mmol) was reacted with 3-ethoxycyclohex-2-en-1-one (**4**) (3.00 g, 21.4 mmol) in dry THF (24 mL) and quenched with 3 M aq. HCl (38 mL) to give **5d** (3.01 g, quant.). ^{1}H NMR (500 MHz, $CDCl_3$): δ = 0.94 (t, J = 7.4 Hz, 3H, H-3’’’), 1.54 (h, J = 7.5 Hz, 2H, H-3’’), 1.99 (p, J = 6.3 Hz, 2H, H-5), 2.19 (t, J = 7.6 Hz, 2H, H-3’), 2.28 (t, J = 5.9 Hz, 2H, H-4), 2.36 (t, J = 6.7 Hz, 2H, H-6), 5.87 (m, 1H, H-2).

General Procedure B:[1]
3-Benzylcyclohex-2-en-1-one (5e). To a stirred suspension of Li (115 mg, 16.7 mmol) in THF (4 mL) under argon at rt, 3-ethoxycyclohex-2-en-1-one (**4**) (1.2 g, 8.3 mmol) and 4-benzyloxy-1-bromobutane (2.1 g, 8.7 mmol) were added successively. After 4 h the reaction mixture was poured into a solution of H_2SO_4 (3 mL) in ice water (100 mL). The aqueous layer was extracted with ether (3 x 50 mL) and the combined organic layers were washed with aq. satd. $NaHCO_3$ solution and brine (each 50 mL), dried over Na_2SO_4, filtered, and evaporated to dryness. Column chromatography of the residue on silica gel (petroleum ether/EtOAc, 6:1) yielded **5e** (325 mg, 21%). ^{1}H NMR (500 MHz, $CDCl_3$): δ = 1.90-2.02 (m, 2H, H-5), 2.26 (td, J = 6.1, 1.4 Hz, 2H, H-4), 2.36 (dd, J = 7.3, 6.1 Hz, 2H, H-6), 3.51 (s, 2H, $CH_2C_6H_5$), 5.86 (m, 1H, H-2), 7.12-7.18, 7.22-7.28, 7.28-7.34 (m, 5H, C_6H_5); ^{13}C NMR (126 MHz, $CDCl_3$): δ = 22.66 (C-5), 29.25 (C-4), 37.31 (C-6), 44.53 ($CH_2C_6H_5$), 126.89 (C-2), 126.93, 128.70, 129.10, 136.99 (6C, C_6H_5), 164.67 (C-3), 199.91 (C-1).

3-(Cyclohexylmethyl)cyclohex-2-en-1-one (5f). Commercially available.

3-Methylcyclohex-2-en-1-ol (3b). Commercially available.

General procedure C:[1]
3-Ethylcyclohex-2-en-1-ol (3c). To a solution of **5c** (2.60 g, 20.6 mmol) in dry ether (35 mL) under argon, $CeCl_3 \cdot 7\,H_2O$ (8.44 g, 22.7 mmol) and MeOH (3.5 mL) were added successively. After cooling to 0 °C, $NaBH_4$ (770 mg, 20.6 mmol) was added in one portion and the reaction mixture was stirred at 0 °C for 3.5 h. Then a satd. aq. NH_4Cl (20 mL, strong gas development) was added. The reaction mixture was diluted with diethyl ether/water and the layers were separated. The organic layer was washed with water and brine. The aqueous layers were extracted once with diethyl ether and the combined organic layers were dried over Na_2SO_4. Evaporation of the volatiles (40 °C, 200 mbar) and column chromatography on silica gel (petroleum ether/EtOAc, 6:1) yielded **3c** (2.5 g, 96%). ^{1}H NMR (500 MHz, $CDCl_3$): δ = 1.01 (t, J = 7.5 Hz, 3H), 1.40 (m, 1H), 1.51-1.67 (m, 2H), 1.68-2.05 (m, 6H), 4.20 (s, 1H), 5.49 (dt, J = 3.6, 1.7 Hz, 1H).

3-Propylcyclohex-2-en-1-ol (3d). According to general procedure C, **5d** (2.00 g, 14.4 mmol) in ether (24 mL) was reacted with $CeCl_3 \cdot 7\ H_2O$ (5.93 g, 15.9 mmol), MeOH (2.4 mL) and $NaBH_4$ (541 mg, 14.5 mmol) to give **3d** (1.5 g, 75%). ^{1}H NMR (500 MHz, $CDCl_3$): δ = 0.89 (t, J = 7.4 Hz, 3H), 1.37 (m, 1H), 1.43 (hd, J = 7.4, 1.3 Hz, 2H), 1.58 (dqd, J = 10.8, 5.4, 3.4, 2.9 Hz, 2H), 1.68-1.84 (m, 2H), 1.88 (m, 1H), 1.95 (t, J = 7.7 Hz, 3H), 4.19 (s, 1H), 5.49 (dt, J = 3.5, 1.7 Hz, 1H).

3-Benzylcyclohex-2-en-1-ol (3e). According to general procedure C, **5e** (650 mg, 3.49 mmol) in ether (6 mL) was reacted with $CeCl_3 \cdot 7\ H_2O$ (1.43 g, 3.84 mmol), MeOH (600 µL) and $NaBH_4$ (130 mg, 3.49 mmol) to give **3e** (605 mg, 93%). ^{1}H NMR (500 MHz, $CDCl_3$): δ = 1.41 (d, J = 4.1 Hz, 1H, OH), 1.50-1.59 (m, 2H, H-5a, H-6a), 1.64-1.98 (m, 4H, H-4, H-5b, H-6b), 3.28 (d, J = 3.0 Hz, 2H, C$H_2C_6H_5$), 4.23 (m, 1H, H-1), 5.55 (dt, J = 3.5, 1.7 Hz, 1H, H-2), 7.12-7.23, 7.24-7.31 (m, 5H, C_6H_5); ^{13}C NMR (126 MHz, $CDCl_3$): δ = 19.11 (C-5), 28.20 (C-4), 31.82 (C-6), 44.19 ($CH_2C_6H_5$), 66.02 (C-1), 125.72 (C-2), 126.14, 128.31, 128.96, 141.66 (6C, C_6H_5), 139.36 (C-3).

3-(Cyclohexylmethyl)cyclohex-2-en-1-ol (3f). According to general procedure C, **5f** (365 mg, 1.90 mmol) in ether (4 mL) was reacted with $CeCl_3 \cdot 7\ H_2O$ (777 mg, 2.09 mmol), MeOH (400 µL) and $NaBH_4$ (71 mg, 1.90 mmol) to give **3f** (365 mg, quant.). ^{1}H NMR (500 MHz, $CDCl_3$): δ = 0.77-0.92 (m, 2H), 1.07-1.31 (m, 3H), 1.35-1.46 (m, 2H), 1.56-1.72 (m, 9H), 1.82-1.89 (m, 3H), 4.19 (s, 1H), 5.46 (dt, J = 3.4, 1.6 Hz, 1H).

Synthesis of cyclohexanones 3g-m

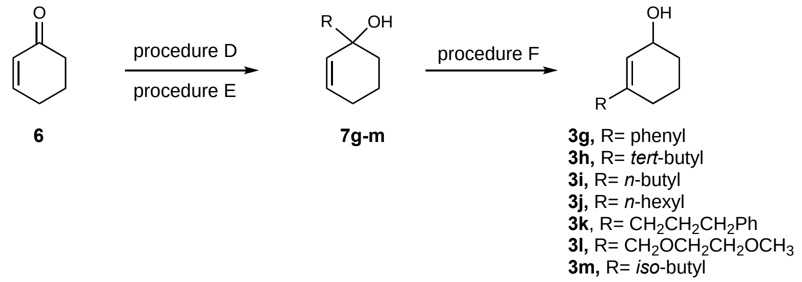

Scheme 2. Synthesis of the cyclohex-2-en-1-one derivatives **3g-m**

General procedure D:[2]

1-Phenylcyclohex-2-en-1-ol (7g). To a mixture of 2-cyclohexen-1-one (**6**) (933 mg, 9.71mmol) in dry THF (40 mL) at -78 °C under argon, phenyl lithium (1.9 M in dibutyl ether, 10.2 mL, 19.4 mmol) was added over a period of 15 min. The reaction mixture was stirred for 1 h and then quenched with satd. aq. NH_4Cl (10 mL) and water (20 mL). After warming to rt over a period of 30 min the reaction mixture was extracted with EtOAc (2 x 50 mL). The combined organic layers were dried over Na_2SO_4, filtered and evaporated to dryness. Column chromatography of the residue on silica gel (petroleum ether/EtOAc, 19:1) gave **7g** (1.5 g, 88%). Analytical data were in accordance to literature.[2]

1-(*tert*-Butyl)cyclohex-2-en-1-ol (7h). According to general procedure D, 2-cyclohexen-1-one (**6**) (933 mg, 9.71 mmol) in dry THF (40 mL) was reacted with *tert*-butyl lithium (1.6 M in pentane, 13 mL, 20.8 mmol) and the reaction was quenched with satd. aq. NH_4Cl (10 mL) and water (20 mL) to give **7h** (900 mg, 56%). Analytical data were in accordance to literature.[2]

1-Butylcyclohex-2-en-1-ol (7i). According to general procedure D, 2-cyclohexen-1-one (**6**) (933 mg, 9.71 mmol) in dry THF (40 mL) was reacted with butyl lithium (1.6 M in hexane, 13 mL, 20.8 mmol) and the reaction was quenched with satd. aq. NH_4Cl (10 mL) and water (20 mL) to give **7i** (1.42 g, 83%). Analytical data were in accordance to literature.[2]

1-Hexylcyclohex-2-en-1-ol (7j). According to general procedure D, 2-cyclohexen-1-one (**6**) (933 mg, 9.71 mmol) in dry THF (40 mL) was reacted with hexyl lithium (2.3 M in pentane, 9.0 mL, 20.8 mmol) and the reaction was quenched with satd. aq. NH_4Cl (10 mL) and water (20 mL) to give **7j** (1.35 g, 75%). ^1H NMR (500 MHz, $CDCl_3$): δ = 0.83-0.93 (m, 3H, CH_3), 1.21-1.41, 1.42-1.57 (m, 10H, 5 CH_2), 1.61-1.74 (m, 4H, H-5, H-6), 1.93 (dddt, J = 17.6, 8.4, 5.5, 2.8 Hz, 1H, H-4a), 2.06 (m, 1H, H-4b), 5.62 (d, J = 10.0 Hz, 1H, H-2), 5.79 (ddd, J = 10.0, 4.6, 2.8 Hz, 1H, H-3); ^{13}C NMR (126 MHz, $CDCl_3$): δ = 14.10 (CH_3), 19.08 (C-5), 22.63, 23.48 (2 CH_2), 25.26 (C-4), 29.89, 31.86 (2 CH_2), 35.43 (C-6), 42.37 (CH_2), 69.75 (C-1), 129.76 (C-3), 132.87 (C-2).

General procedure E:

1-(3-Phenylpropyl)cyclohex-2-en-1-ol (7k). To a mixture of 4,4'-di-*tert*-butylbiphenyl (3.00 g, 11.3 mmol) in dry THF (15 mL) at 0 °C, Li metal (77 mg, 11.3 mmol, cut in small pieces) was added and the reaction mixture was stirred for 4 h. The resulting dark green solution was cooled to -78 °C and treated dropwise with (3-bromopropyl)benzene (1.12 g, 5.63 mmol), as a result the dark color faded. After 5 min cyclohexene (200 µL) was added and after another 10 min 2-cyclohexene-1-one (**6**) (405 mg, 4.22 mmol). Over a period of 2.5 h the reaction mixture was allowed to warm to -60 °C. Satd. aq. NH_4Cl (15 mL) and H_2O (30 mL) were added successively, and the mixture was allowed to warm to rt over a period of 45 min. Extraction with diethyl ether (2 x 50 mL), drying of the combined organic layers with Na_2SO_4, filtering and evaporation to dryness gave the crude product. Column chromatography on silica gel (petroleum ether/EtOAc, 4:1) yielded **7k** (602 mg, 65%). ^1H NMR (500 MHz, $CDCl_3$): δ = 1.44 (s, 1H, OH), 1.47-1.80 (m, 8H, H-5, H-6, 2 CH_2), 1.91 (dddd, J = 17.7, 8.5, 5.6, 2.7 Hz, 1H, H-4a), 2.01 (m, 1H, H-4b), 2.62 (tt, J = 9.1, 4.6 Hz, 2H, CH_2), 5.60 (dd, J = 10.0, 2.8 Hz, 1H, H-2), 5.79 (ddd, J = 10.0, 4.6, 2.8 Hz, 1H, H-3), 7.15-7.21, 7.24-7.30 (m, 5H, C_6H_5); ^{13}C NMR (126 MHz, $CDCl_3$): δ = 19.04 (C-5), 25.24 (C-4), 25.51 (CH_2), 35.45 (C-6), 36.40, 41.93 (2 CH_2), 69.65 (C-1), 125.71, 128.28, 128.42, 142.49 (6C, C_6H_5), 129.98 (C-2), 132.66 (C-3).

1-((2-Methoxyethoxy)methyl)cyclohex-2-en-1-ol (7l). According to general procedure E, 4,4'-di-*tert*-butylbiphenyl (5.00 g, 18.8 mmol) and Li metal (129 mg, 11.3 mmol) in dry THF (30 mL) were reacted with

cyclohexene (400 µL), 1-(chloromethoxy)-2-methoxyethane (1.16 g, 9.38 mmol) and 2-cyclohexen-1-one (**6**) (676 mg, 7.04 mmol) to give **7l** (458 mg, 37 %, impure). ^{1}H NMR (500 MHz, $CDCl_3$): δ = 1.55-1.84, 1.92-2.05, 2.05-2.14 (m, 6H, H-4, H-5, H-6), 2.76 (s, 1H, OH), 3.37 (d, J = 9.6 Hz, 1H, OCH_2), 3.39 (s, 3H, OCH_3), 3.45 (d, J = 9.6 Hz, 1H, OCH_2), 3.52-3.59 (m, 2H, CH_2), 3.64-3.76 (m, 2H, CH_2), 5.65 (d, J = 10.3 Hz, 1H, H-2), 5.88 (dt, J = 10.0, 3.7 Hz, 1H, H-3).

1-Isobutylcyclohex-2-en-1-ol (7m). According to general procedure E, 4,4'-di-*tert*-butylbiphenyl (4.00 g, 15.0 mmol) and Li metal (104 mg, 9.1 mmol) in dry THF (20 mL) were reacted with cyclohexene (300 µL), 1-bromo-2-methylpropane (1.03 g, 7.51 mmol) and 2-cyclohexen-1-one (**6**) (541 mg, 5.63 mmol) to give **7m** (quant). ^{1}H NMR (500 MHz, $CDCl_3$): δ = 0.96 (t, J = 7.0 Hz, 6H, 2 CH_3), 1.39-1.54 (m, 2H, CH_2), 1.61-1.76 (m, 4H, H-5, H-6), 1.82 (dp, J = 13.1, 6.5 Hz, 1H, CH), 1.94 (dtt, J = 17.8, 5.8, 2.6 Hz, 1H, H-4a), 2.03 (dddd, J = 18.8, 6.8, 4.9, 2.4 Hz, 1H, H-4b), 5.65 (m, 1H, H-2), 5.77 (ddd, J = 10.1, 4.5, 2.8 Hz, 1H, H-3); ^{13}C NMR (126 MHz, $CDCl_3$): δ = 19.07 (C-5), 23.98 (CH), 24.52, 24.88 (2 CH_3), 25.16 (C-4), 35.92 (C-6), 50.95 (CH_2), 70.19 (C-1), 129.16 (C-3), 133.36 (C-2).

General procedure F:[2]

3-Phenylcyclohex-2-en-1-ol (3g). A mixture of **7g** (500 mg, 2.87 mmol) and $Pd(TFA)_2$ (3 mg) in acetonitrile/water (15 mL, 4:1) was stirred at 40 °C. After 2 h the reaction mixture was cooled to rt and extracted with ether (2 x 50 mL). The combined organic layers were dried over Na_2SO_4, filtered and evaporated to dryness. Column chromatography of the crude product on silica gel yielded **3g** (330 mg, 66%). ^{1}H NMR (500 MHz, $CDCl_3$): δ = 1.62-1.80 (m, 3H, H-5a, H-6a, OH), 1.85-1.99 (m, 2H, H-5b, H-6b), 2.36 (m, 1H, H-4a), 2.46 (m, 1H, H-4b), 4.38 (qd, J = 5.8, 5.0, 2.1 Hz, 1H, H-1), 6.12 (dt, J = 3.6, 1.8 Hz, 1H, H-2), 7.23-7.28, 7.28-7.34, 7.37-7.43 (m, 5H, C_6H_5); ^{13}C NMR (126 MHz, $CDCl_3$): δ = 19.42 (C-5), 27.47 (C-4), 31.65 (C-6), 66.28 (C-1), 125.33, 126.53, 127.37, 128.12, 128.25, 140.07 (7C, C-2, C_6H_5), 141.31 (C-3).

3-(*tert*-Butyl)cyclohex-2-en-1-ol (3h). According to general procedure F, **7h** (900 mg, 5.84 mmol) and $Pd(TFA)_2$ (6 mg) were reacted in acetonitrile/water (30 mL, 4:1) to give **3h** (740 mg, 82%). Analytical data were in accordance to literature.[2]

3-Butylcyclohex-2-en-1-ol (3i). According to general procedure F, **7i** (1.40 g, 9.08 mmol) and $Pd(TFA)_2$ (9 mg) were reacted in acetonitrile/water (40 mL, 4:1) to give **3i** (850 mg, 60%). Analytical data were in accordance to literature.[2]

3-Hexylcyclohex-2-en-1-ol (3j). According to general procedure F, **7j** (1.35 g, 7.41 mmol) and $Pd(TFA)_2$ (9 mg) were reacted in acetonitrile/water (40 mL, 4:1) to give **3j** (970 mg, 71%). ^{1}H NMR (500 MHz, $CDCl_3$): δ = 0.88 (t, J = 6.9 Hz, 3H, CH_3), 1.18-1.48 (m, 9H, 4 CH_2, OH), 1.52-1.66 (m, 2H, H-5a, H-6a), 1.66-1.84 (m, 2H, H-5b, H-6b), 1.84-2.02 (m, 4H, H-4, CH_2), 4.19 (m, 1H, H-1), 5.49 (dt, J = 3.4, 1.6 Hz, 1H, H-2). ^{13}C

NMR (126 MHz, $CDCl_3$): δ = 14.10 (CH_3), 19.14 (C-5), 22.61, 27.48 (2 CH_2), 28.51 (C-4), 29.05, 31.75 (2 CH_2), 31.99 (C-6), 37.60 (CH_2), 65.96 (C-1), 123.48 (C-2), 142.77 (C-3).

3-(3-Phenylpropyl)cyclohex-2-en-1-ol (3k). According to general procedure F, **7k** (380 mg, 1.76 mmol) and $Pd(TFA)_2$ (3 mg) were reacted in acetonitrile/water (12.5 mL, 4:1) to give **3k** (280 mg, 73%). ^{1}H NMR (500 MHz, $CDCl_3$): δ = 1.32 (d, *J* = 6.2 Hz, 1H, OH), 1.46-1.64 (m, 2H, H-5a, H-6a), 1.67-1.84 (m, 4H, H-5b, H-6b, CH_2), 1.84-1.99 (m, 2H, H-4), 1.99-2.06 (m, 2H, CH_2), 2.60 (t, *J* = 7.7 Hz, 2H, CH_2), 4.20 (m, 1H, H-1), 5.51 (dt, *J* = 3.5, 1.8 Hz, 1H, H-2), 7.15-7.20, 7.25-7.30 (m, 5H, C_6H_5); ^{13}C NMR (126 MHz, $CDCl_3$): δ = 19.12 (C-5), 28.51 (C-4), 29.15 (CH_2), 31.98 (C-6), 35.58, 37.14 (2 CH_2), 65.92 (C-1), 123.98 (C-2), 125.71, 128.28, 128.42, 142.15 (6C, C_6H_5), 142.40 (C-3).

3-((2-Methoxyethoxy)methyl)cyclohex-2-en-1-ol (3l). According to general procedure F, **7l** (430 mg, 2.30 mmol) and $Pd(TFA)_2$ (4 mg) were reacted in acetonitrile/water (7.5 mL, 4:1) to give **3l** (200 mg, 46%). ^{1}H NMR (500 MHz, $CDCl_3$): δ = 1.41 (d, *J* = 4.6 Hz, 1H, OH), 1.48-1.68, 1.68-1.89, 1.89-2.11 (m, 6H, H-3, H-4 H-5), 3.38 (s, 3H, OCH_3), 3.54 (s, 4H, 2 CH_2), 3.91 (t, *J* = 1.2 Hz, 2H, CH_2), 4.21 (m, 1H, H-1), 5.76 (dt, *J* = 3.3, 1.6 Hz, 1H, H-2). ^{13}C NMR (126 MHz, $CDCl_3$): δ = 18.83 (C-5), 25.71 (C-4), 31.88 (C-6), 59.01 (OCH_3), 65.57 (C-1), 69.15, 71.88, 74.88 (3 CH_2), 126.52 (C-2), 138.60 (C-3).

3-Isobutylcyclohex-2-en-1-ol (3m). According to general procedure F, **7m** (1.05 g, 6.81 mmol) and $Pd(TFA)_2$ (8 mg) were reacted in acetonitrile/water (25 mL, 4:1) to give **3m** (692 mg, 77%). ^{1}H NMR (500 MHz, $CDCl_3$): δ = 0.78-0.94 (m, 18H), 1.07-1.44 (m, 5H), 1.46-1.64 (m, 3H), 1.64-2.01 (m, 6H), 4.20 (s, 1H), 5.48 (dt, *J* = 3.4, 1.7 Hz, 1H); ^{13}C NMR (126 MHz, $CDCl_3$): δ = 11.42, 14.31, 18.75, 19.12, 19.43, 20.44, 22.33, 22.57, 22.61, 25.95, 27.66, 28.46, 28.89, 29.05, 31.97, 33.72, 36.07, 41.35, 47.39, 65.94, 125.07, 141.55.

Synthesis of cyclohexanone 3n

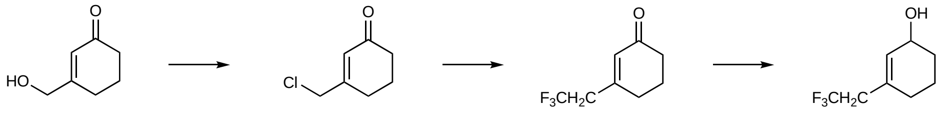

Scheme 3. Synthesis of 3-(2,2,2-trifluoroethyl)cyclohex-2-en-1-ol (**3n**).

3-(Chloromethyl)cyclohex-2-en-1-one (9). To a solution of 3-(hydroxymethyl)cyclohex-2-en-1-one (**8**)[3] (169 mg, 1.34 mmol) in dry DCM (10 mL) 1-chloro-*N*,*N*-2-trimethylpropenylamine (227 µL, 1.6 mmoL) was added dropwise at 0 °C. The reaction mixture was stirred at rt for 2 h, then the solvent was removed under reduced pressure and the residue was purified by chromatography on silica gel (petroleum ether/DCM, 1:1 to 0:1) to give **9** (191 mg, 99%) as pale-yellow oil. ^{1}H NMR (500 MHz, $CDCl_3$): δ = 2.02-2.13 (m, 2H), 2.42 (dd, *J* = 12.0, 5.5 Hz, 4H), 3.94-4.24 (m, 2H), 6.10 (s, 1H).

3-(2,2,2-Trifluoroethyl)cyclohex-2-en-1-one (10). To a solution of **9** (315 mg, 2.18 mmol) in *N*-methyl-2-pyrrolidon (5.0 mL) were added CuI (498 mg, 2.62 mmol) and KF (152 mg, 2.62 mmol) at rt under argon. Then $FSO_2CF_2CO_2Me$ (333 µL, 2.62 mmoL) was added dropwise at rt. The reaction mixture was stirred at 70 °C overnight. After cooling to rt, the reaction mixture was diluted with Et_2O and filtered through celite. The filtrate was washed with H_2O and brine, dried over Na_2SO_4 and the solvent was removed under reduced pressure. The residue was purified by chromatography on silica gel (petroleum ether/DCM, 1:1 to 0:1) to yield **10** (242 mg, 62%) as pale-yellow oil. ^{1}H NMR (500 MHz, $CDCl_3$): δ = 1.99-2.09 (m, 2H), 2.36-2.45 (m, 4H), 2.99 (q, J = 10.6 Hz, 2H), 6.03 (s, 1H); ^{19}F NMR (471 MHz, $CDCl_3$): δ = -63.60.

3-(2,2,2-Trifluoroethyl)cyclohex-2-en-1-ol (3n). To a mixture of **10** (230 mg, 1.29 mmol) and $CeCl_3 \cdot 7\ H_2O$ (192 mg, 0.514 mmol) in Et_2O/EtOH (5.0 mL/0.5 mL) $NaBH_4$ (48.7 mg, 1.29 mmol) was added at 0 °C. The reaction mixture was stirred at 0 °C for 2 h, then quenched carefully with sat. aq. NH_4Cl. The aqueous layer was extracted with Et_2O (3 x), the combined organic layers were washed with brine and dried over Na_2SO_4. After removal of the solvent under reduced pressure, the residue was purified by chromatography on silica gel (DCM/MeOH, 1:0 to 30:1) to give **3n** (214 mg, 92%) as colorless oil. ^{1}H NMR (500 MHz, $CDCl_3$): δ = 1.43 (d, J = 6.7 Hz, 1H), 1.61 (qdd, J = 8.7, 7.3, 2.3 Hz, 2H), 1.78 (m, 1H), 1.87 (m, 1H), 2.76 (tt, J = 13.6, 7.0 Hz, 2H), 1.96.-2.15 (m, 2H), 4.24 (s, 1H), 5.76 (s, 1H); ^{19}F NMR (471 MHz, $CDCl_3$): δ = -64.47 (t, J = 11.0 Hz); ^{13}C NMR (126 MHz, $CDCl_3$): δ = 19.06, 28.76, 31.30, 41.77 (q, J = 28.9 Hz), 53.41, 65.62, 125.88 (d, J = 277.7 Hz), 131.56.

Synthesis of glycomimetics (*R*)-14b-n

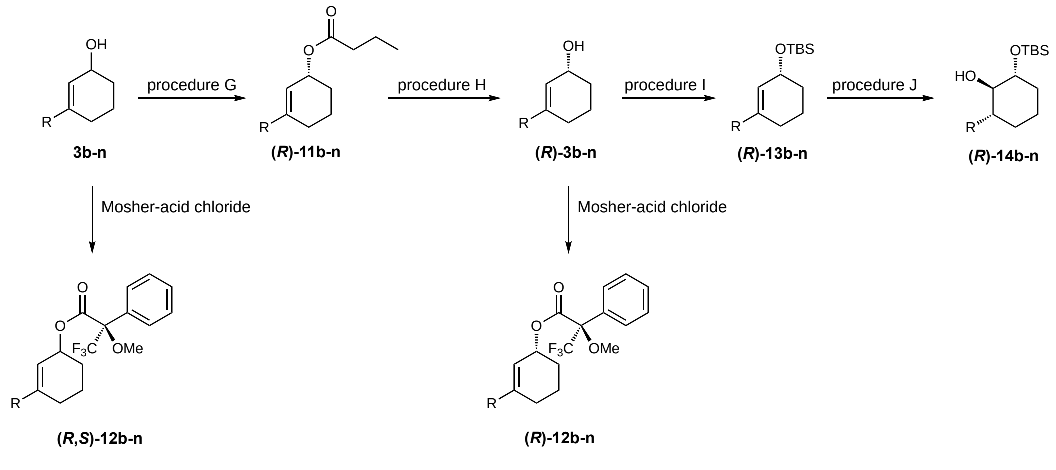

Scheme 4. Glc*N*Ac mimetics **(*R*)-14b-n**.

Procedure for the synthesis of Mosher derivatives ((*R*,*S*)-12b-n, (*R*)-12b-n). To a mixture of **3b-n** or **(*R*)-3b-n** (0.05 mmol) in DCM (100 µL) at 0 °C, DMAP (0.1 mmol) and (*R*)-(-)-α-methoxy-α-(trifluoromethyl)-phenylacetyl chloride (0.075 mmol) were added successively. The reaction was stirred at 0 °C for 5 min and an additional hour at rt. The mixture was diluted with ether (5 mL) and washed with 2N aq. HCl, satd. aq.

$NaHCO_3$ and brine (each 5 mL). The organic layer was dried over Na_2SO_4, filtered and evaporated to dryness to give **(*R*,*S*)-(*rac*)-12b-n** and **(*R*)-12b-n**. For ^{19}F NMR data see chapter 5.

General procedure G:

(*R*)-3-Methylcyclohex-2-en-1-yl butyrate ((*R*)-11b).[4,5] Novozym 435 (222 mg, 444 U, EC 232-619-9, Sigma-Aldrich cat.nr. 73940) was added to a solution of racemic **3b** (10.0 g, 89 mmol) and vinyl butyrate (22.6 mL, 20.3 g, 178 mmol) in heptane (90 mL). The mixture was stirred at 23 °C and 200 rpm. After 2 h 25 min the mixture was filtered, and volatiles were evaporated at 60 °C and 10 mbar to give 12 g of a clear oil. Column chromatography on silica (DCM) yielded pure **(*R*)-11b** (7.50 g, 41 mmol, 46%). $[\alpha]_D^{20}$ +168.7 (*c* 9.28, $CHCl_3$); ^{1}H NMR (500 MHz, $CDCl_3$): δ = 0.92 (t, *J* = 7.4Hz, 3H, H-1’’’), 1.56-1.81 (m, 9H, H-5, H-6, CH_3, H-1’’), 1.84-2.02 (m, 2H, H-4), 2.24 (t, *J* = 7.4Hz, 2H, H-1’), 5.23 (m, 1H, H-1), 5.44 (m, 1H, H-2); ^{13}C NMR (126 MHz, $CDCl_3$): δ = 13.7 (C-1’’’), 18.7, 19.1 (C-5, C-1’’), 23.8 (CH_3), 28.1 (C-6), 30.0 (C-4), 36.7 (C-1’), 68.6 (C-1), 120.2 (C-2), 141.0 (C-3), 173.5 (C=O); ESI-MS: *m/z*: Calcd for $C_{11}H_{18}NaO_2^+$ $[M+Na]^+$: 205.1; found: 204.8; Anal. Calcd. for $C_{11}H_{18}O_2$ (182.26): C 72.49, H 9.95; found: C 72.87, H 9.65.

(*R*)-3-Ethylcyclohex-2-en-1-yl butyrate ((*R*)-11c). According to general procedure G, racemic **3c** (2.00 g, 15.6 mmol) was reacted with vinyl butyrate (4.0 mL, 31.2 mmol) and lipase (40 mg) in heptane (14 mL) to give **(*R*)-11c** (1.14 g, 37%). $[\alpha]_D^{20}$ +138.9 (*c* 1.6, $CHCl_3$); ^{1}H NMR (500 MHz, $CDCl_3$): δ = 0.95 (t, *J* = 7.4 Hz, 3H, H-1’’’), 1.01 (t, *J* = 7.5 Hz, 3H, H-3’’), 1.58-1.84, 1.89-2.05 (m, 10H, H-3’, H-1’’, H-4, H-5, H-6), 2.27 (t, *J* = 7.4 Hz, 2H, H-1’), 5.28 (td, *J* = 6.1, 5.5, 2.5 Hz, 1H, H-1), 5.44 (dp, *J* = 3.1, 1.5 Hz, 1H, H-2); ^{13}C NMR (126 MHz, $CDCl_3$): δ = 11.90 (C-3’’), 13.65 (C-1’’’), 18.59 (C-1’’), 19.22, 28.38, 28.41 (C-4, C-5, C-6), 30.31 (C-3’), 36.65 (C-1’), 68.59 (C-1), 118.39 (C-2), 146.10 (C-3), 173.48 (C=O).

(*R*)-3-Propylcyclohex-2-en-1-yl butyrate ((*R*)-11d). According to general procedure G, racemic **3d** (1.26 g, 8.99 mmol) was reacted with vinyl butyrate (2.05 g, 18.0 mmol) and lipase (23 mg) in heptane (8 mL) to give **(*R*)-11d** (553 mg, 29%). $[\alpha]_D^{20}$ +155.8 (*c* 0.51, $CHCl_3$); ^{1}H NMR (500 MHz, $CDCl_3$): δ = 0.88 (t, *J* = 7.4 Hz, 3H, H-3’’’), 0.95 (t, *J* = 7.4 Hz, 3H, H-1’’’), 1.43 (hd, *J* = 7.4, 1.4 Hz, 2H, H-3’’), 1.51-1.85 (m, 6H, H-1’’, H-5, H-6), 1.85-2.06 (m, 4H, H-3’, H-4), 2.27 (t, *J* = 7.5 Hz, 2H, H-1’), 5.28 (d, *J* = 4.6 Hz, 1H, H-1), 5.44 (dt, *J* = 3.5, 1.8 Hz, 1H, H-2); ^{13}C NMR (126 MHz, $CDCl_3$): δ = 13.66 (C-1’’’), 13.79 (C-3’’’), 18.60 (C-1’’), 19.22 (C-5), 20.53 (C-3’’), 28.31, 28.38 (C-4, C-6), 36.66 (C-1’), 39.75 (C-3’), 68.57 (C-1), 119.65 (C-2), 144.49 (C-3), 173.50 (C=O).

(*R*)-3-Benzylcyclohex-2-en-1-yl butyrate ((*R*)-11e). According to general procedure G, racemic **3e** (300 mg, 1.59 mmol) was reacted with vinyl butyrate (364 mg, 3.19 mmol) and lipase (6 mg) in heptane (3 mL) to give **(*R*)-11e** (180 mg, 43%). ^{1}H NMR (500 MHz, $CDCl_3$): δ = 0.95 (t, *J* = 7.4 Hz, 3H, H-1’’’), 1.54-1.75 (m, 5H, H-1’’, H-5, H-6a), 1.75-1.91 (m, 2H, H-4a, H-6b), 1.95 (dt, *J* = 17.6, 5.7 Hz, 1H, H-4b), 2.27 (t, *J* = 7.4 Hz, 2H, H-1’), 3.22-3.40 (m, 2H, $CH_2C_6H_5$), 5.31 (m, 1H, H-1), 5.53 (dt, *J* = 3.4, 1.6 Hz, 1H, H-2), 7.14-7.23, 7.24-7.30 (m, 5H, C_6H_5); ^{13}C NMR (126 MHz, $CDCl_3$): δ = 13.66 (C-1’’’), 18.60 (C-1’’), 19.17 (C-5), 27.95

(C-4), 28.21 (C-6), 36.62 (C-1’), 44.30 ($CH_2C_6H_5$), 68.48 (C-1), 121.77 (C-2), 126.19, 128.28, 128.32, 128.38, 128.92, 143.54 (6C, C_6H_5), 139.18 (C-3), 173.45 (C=O).

(*R*)-3-(Cyclohexylmethyl)cyclohex-2-en-1-yl butyrate ((*R*)-11f). According to general procedure G, racemic **3f** (337 mg, 1.73 mmol) was reacted with vinyl butyrate (395 mg, 3.47 mmol) and lipase (7 mg) in heptane (1.4 mL) to give **(*R*)-11f** (152 mg, 33 %). $[\alpha]_D^{20}$ +142.7 (*c* 0.48, DCM); ^{1}H NMR (500 MHz, $CDCl_3$): δ = 0.73-0.91 (m, 2H), 0.94 (t, *J* = 7.4 Hz, 3H), 1.08-1.30 (m, 3H), 1.40 (ddd, *J* = 11.0, 7.4, 3.5 Hz, 1H), 1.55-1.83 (m, 10H), 1.53-2.07 (m, 4H), 2.27 (t, *J* = 7.4 Hz, 2H), 5.27 (q, *J* = 4.4 Hz, 1H), 5.41 (dt, *J* = 3.3, 1.8 Hz, 1H); ^{13}C NMR (126 MHz, $CDCl_3$): δ = 13.64, 18.60, 19.23, 26.31, 26.35, 26.58, 28.35, 28.44, 33.11, 33.45, 35.46, 36.65, 45.95, 68.54 (C-1), 120.98 (C-2), 143.03 (C-3), 173.51 (C=O).

(*R*)-3-Phenylcyclohex-2-en-1-yl butyrate ((*R*)-11g). According to general procedure G, racemic **3g** (330 mg, 1.89 mmol) was reacted with vinyl butyrate (432 mg, 3.79 mmol) and lipase (7 mg) in heptane (2.5 mL) to give **(*R*)-11g** (150 mg, 32%). $[\alpha]_D^{20}$ +144.6 (*c* 1.22, DCM); ^{1}H NMR (500 MHz, $CDCl_3$): δ = 0.96 (t, *J* = 7.4 Hz, 3H, H-3’), 1.67 (h, *J* = 7.4 Hz, 2H, H-2’), 1.73-1.85 (m, 2H, H-6a, H-5a), 1.85-1.99 (m, 2H, H-6b, H-5b), 2.30 (t, *J* = 7.4 Hz, 2H, H-1’), 2.40 (m, 1H, H-4a), 2.53 (m, 1H, H-4b), 5.47 (qd, *J* = 5.1, 3.9, 1.7 Hz, 1H, H-1), 6.07 (dt, *J* = 3.6, 1.7 Hz, 1H, H-2), 7.21-7.46 (m, 5H, C_6H_5); ^{13}C NMR (126 MHz, $CDCl_3$): δ = 13.67 (C-3’), 18.57 (C-2’), 19.45 (C-5), 27.37 (C-4), 28.06 (C-6), 36.58 (C-1’), 68.64 (C-1), 122.46 (C-2), 125.43, 127.59, 128.28, 142.02 (6C, C_6H_5), 141.13 (C-3), 173.44 (C=O).

(*R*)-3-(*tert*-Butyl)cyclohex-2-en-1-yl butyrate ((*R*)-11h). According to general procedure G, racemic **3h** (1.00 g, 6.48 mmol) was reacted with vinyl butyrate (1.48 g, 13.0 mmol) and lipase (25 mg) in heptane (7.5 mL) to give **(*R*)-11h** (380 mg, 26%). $[\alpha]_D^{20}$ +133.8 (*c* 1.05, DCM); ^{1}H NMR (500 MHz, $CDCl_3$): δ = 0.95 (t, *J* = 7.4 Hz, 3H, H-1’’’), 1.04 (s, 9H, $(CH_3)_3$), 1.57-1.89 (m, 6H, H-1’’, H-5, H-6), 1.98 (m, 1H, H-4a), 2.10 (m, 1H, H-4b), 2.27 (t, *J* = 7.4 Hz, 2H, H-1’), 5.32 (dt, *J* = 5.6, 4.0 Hz, 1H, H-1), 5.46 (dt, *J* = 3.5, 1.6 Hz, 1H, H-2); ^{13}C NMR (126 MHz, $CDCl_3$): δ = 13.63 (C-1’’’), 18.60 (C-1’’), 19.96 (C-5), 24.51 (C-4), 28.43 (C-6), 28.83 ($C(CH_3)_3$), 28.89 ($C(CH_3)_3$), 36.67 (C-1’), 69.20 (C-1), 116.89 (C-2), 151.87 (C-3), 173.54 (C=O).

(*R*)-3-Butylcyclohex-2-en-1-yl butyrate ((*R*)-11i). According to general procedure G, racemic **3i** (200 mg, 1.30 mmol) was reacted with vinyl butyrate (295 mg, 2.59 mmol) and lipase (5 mg) in heptane (1.6 mL) to give **(*R*)-11i** (92 mg, 31%). $[\alpha]_D^{20}$ +137.2 (*c* 1.08, DCM).

(*R*)-3-Hexylcyclohex-2-en-1-yl butyrate ((*R*)-11j). According to general procedure G, racemic **3j** (970 mg, 5.32 mmol) was reacted with vinyl butyrate (1.21 g, 10.6 mmol) and lipase (20 mg) in heptane (7.5 mL) to give **(*R*)-11j** (532 mg, 40%). $[\alpha]_D^{20}$ +140.9 (*c* 1.10, DCM); ^{1}H NMR (500 MHz, $CDCl_3$): δ = 0.88 (t, *J* = 6.9 Hz, 3H, CH_3), 0.94 (t, *J* = 7.4 Hz, 3H, H-1’’’), 1.21-1.34 (m, 6H, 3 CH_2), 1.34-1.45 (m, 2H, CH_2), 1.58-1.84 (m, 6H, H-1’’, H-5, H-6), 1.85-2.03 (m, 4H, CH_2, H-4), 2.26 (t, *J* = 7.4 Hz, 2H, H-1’), 5.27 (m, 1H, H-1), 5.44 (dt, *J* = 3.4, 1.6 Hz, 1H; H-2); ^{13}C NMR (126 MHz, $CDCl_3$): δ = 13.66 (C-1’’’), 14.08 (CH_3), 18.60, 19.22 (C-

5, C-1''), 22.60, 27.36 (2 CH_2), 28.38 (2C, C-4, C-6), 29.01, 31.71 (2 CH_2), 36.65 (C-1'), 37.67 (CH_2), 68.59 (C-1), 119.42 (C-2), 144.80 (C-3), 173.51 (C=O).

(*R*)-3-(3-Phenylpropyl)cyclohex-2-en-1-yl butyrate ((*R*)-11k). According to general procedure G, racemic **3k** (240 mg, 1.11 mmol) was reacted with vinyl butyrate (253 mg, 2.22 mmol) and lipase (4 mg) in heptane (1.5 mL) to give **(*R*)-11k** (80 mg, 26%). $[\alpha]_D^{20}$ +127.6 (*c* 0.56, DCM); ^{1}H NMR (500 MHz, $CDCl_3$): δ= 0.94 (t, *J* = 7.4 Hz, 3H, H-1'''), 1.58-1.84 (m, 6H, H-5, H-6, H-1'', CH_2), 1.87-2.01 (m, 2H, H-4), 2.03 (t, *J* = 7.8 Hz, 2H, CH_2), 2.27 (t, *J* = 7.4 Hz, 2H, H-1'), 2.59 (t, *J* = 7.7 Hz, 2H, CH_2), 5.28 (t, *J* = 4.3 Hz, 1H, H-1), 5.47 (dt, *J* = 3.6, 1.8 Hz, 1H, H-2), 7.15-7.20, 7.25-7.30 (m, 5H, C_6H_5); ^{13}C NMR (126 MHz, $CDCl_3$): δ = 13.68 (C-1'''), 18.61 (C-5), 19.22 (C-1''), 28.37, 28.40 (C-4, CH_2), 29.11 (C-6), 35.54 (CH_2), 36.66 (C-1'), 37.18 (CH_2), 68.52 (C-1), 119.95 (C-2), 125.74, 128.30, 128.43, 142.35 (6C, C_6H_5), 144.18 (C-3), 173.50 (C=O).

(*R*)-3-((2-Methoxyethoxy)methyl)cyclohex-2-en-1-yl butyrate ((*R*)-11l). According to general procedure G, racemic **3l** (280 mg, 1.50 mmol) was reacted with vinyl butyrate (343 mg, 3.01 mmol) and lipase (5 mg) in heptane (1.5 mL) to give **(*R*)-11l** (156 mg, 40%). $[\alpha]_D^{20}$ +117.7 (*c* 0.53, DCM).

(*R*)-3-Isobutylcyclohex-2-en-1-yl butyrate ((*R*)-11m). According to general procedure G, racemic **3m** (600 mg, 3.89 mmol) was reacted with vinyl butyrate (887 mg, 7.78 mmol) and lipase (8 mg) in heptane (1.5 mL) to give **(*R*)-11k** (150 mg, 17%). $[\alpha]_D^{20}$ +173.9 (*c* 1.15, DCM); ^{1}H NMR (500 MHz, $CDCl_3$): δ= 0.84 (d, *J* = 6.5 Hz, 3H, CH_3), 0.87 (d, *J* = 6.5 Hz, 3H, CH_3), 0.94 (t, *J* = 7.4 Hz, 3H, H-1'''), 1.49-2.03 (m, 10H, H-4, H-5, H-6, H-1'', CH, CH_2), 2.27 (t, *J* = 7.4 Hz, 2H, H-1'), 5.27 (m, 1H, H-1), 5.43 (dt, *J* = 3.3, 1.6 Hz, 1H, H-2).

(*R*)-3-(2,2,2-Trifluoroethyl)cyclohex-2-en-1-yl butyrate ((*R*)-11n). According to general procedure G, racemic **3n** (230 mg, 1.29 mmol) was reacted with vinyl butyrate (0.326 mL, 2.57 mmol) and lipase (3.2 mg) in heptane (1.3 mL) to give **(*R*)-11n** (69 mg, 20%). ^{1}H NMR (500 MHz, $CDCl_3$): δ = 0.95 (t, *J* = 7.4 Hz, 3H), 1.58-1.90 (m, 6H), 1.98-2.19 (m, 2H), 2.28 (t, *J* = 7.4 Hz, 2H), 2.61-2.92 (m, 2H), 5.29 (d, *J* = 5.5 Hz, 1H), 5.72 (s, 1H); ^{19}F NMR (471 MHz, $CDCl_3$): δ = -64.40.

General procedure H:

(*R*)-3-Methylcyclohex-2-en-1-ol ((*R*)-3b).[5] 4 N aq. NaOH (10.3 mL) was slowly added to a solution of butyrate **(*R*)-11b** (3.50 g, 19.0 mmol) in MeOH (30 mL) at 0 °C and stirred at 0 °C for 5 h. The mixture was diluted with H_2O (25 mL) and extracted with DCM (25 mL + 20 mL + 15 mL). The combined organic layers were washed with brine (25 mL) and dried over Na_2SO_4. Filtration and evaporation of volatiles (200 mbar, 40 °C) gave **(*R*)-3b (**1.81g, 84%), which was used in the next step without further purification.[5]

(*R*)-3-Ethylcyclohex-2-en-1-ol ((*R*)-3c). According to general procedure H, **(*R*)-11c** (1.14 g, 5.81 mmol) in MeOH (10 mL) was treated with 4 N NaOH (3.2 mL). The crude product **(*R*)-3c** (1,42 g, 86%) was used in the next step without further purification.

(*R*)-3-Propylcyclohex-2-en-1-ol ((*R*)-3d). According to general procedure H, **(*R*)-11d** (550 mg, 2.62 mmol) in MeOH (5 mL) was treated with 4 N NaOH (1.5 mL). The crude product **(*R*)-3d** (366 mg, 99%) was used in the next step without further purification.

(*R*)-3-Benzylcyclohex-2-en-1-ol ((*R*)-3e). According to general procedure H, **(*R*)-11e** (167 mg, 0.647 mmol) in MeOH (3 mL) was treated with 4 N NaOH (438 µL). The crude product **(*R*)-3e** (quant) was used in the next step without further purification.

(*R*)-3-(Cyclohexylmethyl)cyclohex-2-en-1-ol ((*R*)-3f). According to general procedure H, **(*R*)-11f** (148 mg, 0.560 mmol) in MeOH (3 mL) was treated with 4 N NaOH (570 µL). The crude product **(*R*)-3f** (quant) was used in the next step without further purification.

(*R*)-3-Phenylcyclohex-2-en-1-ol ((*R*)-3g). According to general procedure H, **(*R*)-11g** (130 mg, 0.532 mmol) in MeOH (1 mL) was treated with 4 N NaOH (250 µL). The crude product **(*R*)-3g** (115 mg) was used in the next step without further purification.

(*R*)-3-(*tert*-Butyl)cyclohex-2-en-1-ol ((*R*)-3h). According to general procedure H, **(*R*)-11h** (200 mg, 0.892 mmol) in MeOH (2.3 mL) was treated with 4 N NaOH (450 µL). The crude product **(*R*)-3h** (140 mg) was used in the next step without further purification.

(*R*)-3-Butylcyclohex-2-en-1-ol ((*R*)-3i). According to general procedure H, **(*R*)-11i** (70 mg, 0.312 mmol) in MeOH (1 mL) was treated with 4 N NaOH (150 µL). The crude product **(*R*)-3i** (41 mg) was used in the next step without further purification.

(*R*)-3-Hexylcyclohex-2-en-1-ol ((*R*)-3j). According to general procedure H, **(*R*)-11j** (520 mg, 2.06 mmol) in MeOH (4.6 mL) was treated with 4 N NaOH (1.2 mL). The crude product **(*R*)-3j** (360 mg) was used in the next step without further purification.

(*R*)-3-(3-Phenylpropyl)cyclohex-2-en-1-ol ((*R*)-3k). According to general procedure H, **(*R*)-11k** (73 mg, 0.268 mmol) in MeOH (2 mL) was treated with 4 N NaOH (125 µL). The crude product **(*R*)-3k** (53 mg) was used in the next step without further purification.

(*R*)-3-((2-Methoxyethoxy)methyl)cyclohex-2-en-1-ol ((*R*)-3l). According to general procedure H, **(*R*)-11l** (141 mg, 0.550 mmol) in MeOH (3.6 mL) was treated with 4 N NaOH (270 µL). The crude product **(*R*)-3l** (82 mg) was used in the next step without further purification.

(*R*)-3-Isobutylcyclohex-2-en-1-ol ((*R*)-3m). According to general procedure H, **(*R*)-11m** (150 mg, 0.669 mmol) in MeOH (3 mL) was treated with 4 N NaOH (312 µL). The crude product **(*R*)-3m** (quant) was used in the next step without further purification.

(*R*)-3-(2,2,2-Trifluoroethyl)cyclohex-2-en-1-ol ((*R*)-3n). According to general procedure H, **(*R*)-11n** (64 mg, 0.255 mmol) in MeOH (0.5 mL) was treated with 4 N NaOH (200 µL). The crude product **(*R*)-3n** (35 mg, 76%) was used in the next step without further purification.

General procedure I:

(***R***)**-*tert*-Butyldimethyl[(3-methylcyclohex-2-en-1-yl)oxy]silane ((*R*)-13b).** Imidazole (4.40 g, 65 mmol) was added to a solution of **(*R*)-3b** (3.50 g, 31 mmol), DMAP (cat.) and TBSCl (7.31 g, 48 mmol) in anhydrous DCM (65 mL) at rt under argon. After stirring for 15 h, the reaction mixture was quenched with satd. aq. $NaHCO_3$ (50 mL) and extracted with DCM (20 mL). The organic layer was washed with 0.01 N aq. HCl (20 mL), satd. aq. $NaHCO_3$ (20 mL) and brine (20 mL), and dried over Na_2SO_4. Filtration and evaporation of volatiles (200 mbar, 40 °C) gave the TBS ether **(*R*)-13b** (quant) as clear oil, which was used in the next step without further purification.

(*R*)-*tert*-Butyl[(3-ethylcyclohex-2-en-1-yl)oxy]dimethylsilane ((*R*)-13c). According to procedure I, **(*R*)-3c**, TBSCl (3.14 g, 20.8 mmol) and DMAP (cat.) in dry DCM (28 mL) were reacted with imidazole (2.00 g, 29.2 mmol) to give **(*R*)-13c** (quant) which was used in the next step without further purification.

(*R*)-*tert*-Butyldimethyl[(3-propylcyclohex-2-en-1-yl)oxy]silane ((*R*)-13d). According to procedure I, **(*R*)-3d**, TBSCl (612 mg, 4.07 mmol) and DMAP (cat.) in dry DCM (7 mL) were reacted with imidazole (387 mg, 5.69 mmol) to give **(*R*)-13d** (820 mg) which was used in the next step without further purification.

(*R*)-[(3-Benylcyclohex-2-en-1-yl)oxy](*tert*-butyl)dimethylsilane ((*R*)-13e). According to procedure I, **(*R*)-3e**, TBSCl (102 mg, 0.675 mmol) and DMAP (cat.) in dry DCM (1.5 mL) were reacted with imidazole (92 mg, 1.35 mmol) to give **(*R*)-13e** (183 mg) which was used in the next step without further purification.

(*R*)-*tert*-Butyl[(3-(cyclohexylmethyl)cyclohex-2-en-1-yl)oxy]dimethylsilane ((*R*)-13f). According to procedure I, **(*R*)-3f** (140 mg, 0.721 mmol), TBSCl (114 mg, 0.757 mmol) and DMAP (cat.) in dry DCM (1.7 mL) were reacted with imidazole (103 mg, 1.51 mmol) to give **(*R*)-13f** (quant) which was used in the next step without further purification.

(*R*)-*tert*-Butyldimethyl[(3-phenylcyclohex-2-en-1-yl)oxy]silane ((*R*)-13g). According to procedure I, **(*R*)-3g** (115 mg, 0.660 mmol), TBSCl (149 mg, 0.990 mmol) and DMAP (cat.) in dry DCM (1.5 mL) were reacted with imidazole (94 mg, 1.39 mmol) to give **(*R*)-13g** (173 mg) which was used in the next step without further purification.

(*R*)-*tert*-Butyl[(3-(*tert*-butyl)cyclohex-2-en-1-yl)oxy]dimethylsilane ((*R*)-13h). According to procedure I, **(*R*)-3h** (130 mg, 0.843 mmol), TBSCl (190 mg, 1.26 mmol) and DMAP (cat.) in dry DCM (1.5 mL) were reacted with imidazole (120 mg, 1.77 mmol) to give **(*R*)-13h** (230 mg) which was used in the next step without further purification.

(*R*)-*tert*-Butyl[(3-butylcyclohex-2-en-1-yl)oxy]dimethylsilane ((*R*)-13i). According to procedure I, **(*R*)-3i** (100 mg, 0.648 mmol), TBSCl (146 mg, 0.972 mmol) and DMAP (cat.) in dry DCM (1.5 mL) were reacted with imidazole (92 mg, 1.36 mmol) to give **(*R*)-13i** (220 mg) which was used in the next step without further purification.

(*R*)-*tert*-Butyl[(3-hexylcyclohex-2-en-1-yl)oxy]dimethylsilane ((*R*)-13j). According to procedure I, **(*R*)-3j** (360 mg, 1.98 mmol), TBSCl (446 mg, 2.96 mmol) and DMAP (cat.) in dry DCM (4.5 mL) were reacted with imidazole (282 mg, 4.15 mmol) to give **(*R*)-13j** (600 mg) which was used in the next step without further purification.

(*R*)-*tert*-Butyldimethyl[(3-(3-Phenylpropyl)cyclohex-2-en-1-yl)oxy]silane ((*R*)-13k). According to procedure I, **(*R*)-3k** (48 mg, 0.222 mmol), TBSCl (50 mg, 0.333 mmol) and DMAP (cat.) in dry DCM (1.0 mL) were reacted with imidazole (31 mg, 466 mmol) to give **(*R*)-13k** (48 mg) which was used in the next step without further purification.

(*R*)-*tert*-Butyl[(3-((2-methoxyethoxy)methyl)cyclohex-2-en-1-yl)oxy]dimethylsilane ((*R*)-13l). According to procedure I, **(*R*)-3l** (80 mg, 0.429 mmol), TBSCl (97 mg, 0.644 mmol) and DMAP (cat.) in dry DCM (1.0 mL) were reacted with imidazole (61 mg, 0.902 mmol) to give **(*R*)-13l** (quant) which was used in the next step without further purification.

(*R*)-*tert*-Butyl[(3-isobutylcyclohex-2-en-1-yl)oxy]dimethylsilane ((*R*)-13m). According to procedure I, **(*R*)-3m** (138 mg, 0.895 mmol), TBSCl (202 mg, 1.34 mmol) and DMAP (cat.) in dry DCM (2.0 mL) were reacted with imidazole (128 mg, 1.88 mmol) to give **(*R*)-13m** (quant) which was used in the next step without further purification.

(*R*)-*tert*-Butyldimethyl[(3-(2,2,2-trifluoroethyl)cyclohex-2-en-1-yl)oxy]silane ((*R*)-13n). According to procedure I, **(*R*)-3n** (47 mg, 0.26 mmol), TBSCl (58 mg, 0.39 mmol) and DMAP (1.6 mg) in dry DCM (0.2 mL) were reacted with imidazole (35 mg, 0.52 mmol) to give **(*R*)-13n** (quant) which was used in the next step without further purification.

General procedure J:

(1*R*,2*R*,6*S*)-2-((*tert*-Butyldimethylsilyl)oxy)-6-methylcyclohexan-1-ol ((*R*)-14b). A solution of BH_3·THF (1 M in THF, 60 mL) was slowly added to a solution of the crude TBS ether **(*R*)-13b** in anhydrous THF (60 mL) under argon at 0 °C. After stirring for 2 h at rt, the reaction mixture was cooled to 0 °C and 3 N aq. NaOH (180 mL) followed by 30% aq. H_2O_2 (180 mL) were slowly added *via* dropping funnel (CAUTION: strong gas development). The mixture was stirred at 0 °C for 1 h, then acidified to pH 3 by slow addition of 10 % aq. HCl *via* dropping funnel (CAUTION: strong gas development) and extracted with DCM (2 x 300 mL). The extracts were dried over Na_2SO_4, filtered, concentrated (100 mbar, 40 °C) and purified by column chromatography (petroleum ether/Et_2O, 98.5:1.5) to yield pure **(*R*)-14b** (6.20 g, 25 mmol, 81%) as clear oil. $[\alpha]_D^{20}$ -13.7 (*c* 3.14, $CHCl_3$); ^1H NMR (500 MHz, $CDCl_3$): δ = 0.05, 0.07 (2 s, 6H, 2 $SiCH_3$), 0.83-0.91 (m, 9H, $SiC(CH_3)_3$), 0.92-1.04 (m, 4H, H-4b, CH_3), 1.34-1.99 (m, 2H, H-5b, H-6b), 1.41 (m, 1H, H-3), 1.56-1.63 (m, 2H, H-4a, H-5a), 1.81 (m, 1H, H-6a), 2.47 (s, 1H, OH), 2.92 (dd, *J* = 8.5 Hz, 10.0 Hz, 1H, H-2), 3.34 (m, 1H, H-1); ^{13}C NMR (126 MHz, $CDCl_3$): δ = -4.6, -3.9 (2 $SiCH_3$), 18.1 (Si*C*($CH_3)_3$), 18.5 (CH_3), 23.6 (C-5), 25.9 (3C, SiC(*C*$H_3)_3$), 33.4, 33.9 (C-4, C-6), 37.0 (C-3), 77.0 (C-1), 81.0 (C-2); HR-MS (ESI): *m/z*: Calcd for $C_{13}H_{28}NaO_2Si^+$ [M+Na$]^+$: 267.1751; found: 267.1752.

(1*R*,2*R*,6*S*)-2-((*tert*-Butyldimethylsilyl)oxy)-6-ethylcyclohexan-1-ol ((*R*)-14c). According to general procedure J, **(*R*)-13c** in THF (30 mL) was reacted with BH_3·THF (30 mL) and quenched with 3 N NaOH (90 mL) and 30% H_2O_2 (90 mL) to give **(*R*)-14c** (3.6 g, 98%). ^1H NMR (500 MHz, $CDCl_3$): δ = 0.08, 0.10 (2 s, 6H, $Si(CH_3)_2$), 0.88-0.93 (m, 12H, $SiC(CH_3)_3$, CH_3), 1.12-1.36 (m, 4H, H-3, H-4b, H-5b, H-6b), 1.60-1.92 (m, 5H, H-4a, H-5a, H-6a, CH_2), 2.51 (s, 1H, OH), 3.04 (dd, *J* = 10.0, 8.5 Hz, 1H, H-2), 3.38 (ddd, *J* = 10.6, 8.3, 4.5 Hz, 1H, H-1).

(1*R*,2*R*,6*S*)-2-((*tert*-Butyldimethylsilyl)oxy)-6-propylcyclohexan-1-ol ((*R*)-14d). According to general procedure J, **(*R*)-13d** in THF (5 mL) was reacted with BH_3·THF (5.3 mL) and quenched with 3 N NaOH (16 mL) and 30% H_2O_2 (16 mL) to give **(*R*)-14d** (570 mg, 76%). ^1H NMR (500 MHz, $CDCl_3$): δ = 0.08, 0.10 (2 s, 6H, $Si(CH_3)_2$), 0.88-0.93 (m, 13H, $SiC(CH_3)_3$, CH_3, CH_2), 1.08-1.48 (m, 6H, H-3, H-4a, H-5a, H-6a, CH_2), 1.67 (m, 1H, H-5b), 1.71-1.81 (m, 2H, H-4b, CH_2), 1.84 (m, 1H, H-6b), 2.51 (d, *J* = 1.5 Hz, 1H, OH), 3.02 (ddd, *J* = 10.0, 8.3, 1.4 Hz, 1H, H-2), 3.37 (ddd, *J* = 10.5, 8.3, 4.4 Hz, 1H, H-1); ^{13}C NMR (126 MHz, $CDCl_3$): δ = -4.73, -3.98 ($SiCH_3)_2$), 14.46 (CH_3), 18.03 (Si*C*($CH_3)_3$), 19.72 (CH_2), 23.51 (C-5), 25.83 (SiC(*C*$H_3)_3$), 29.78 (C-4), 33.62 (C-6), 34.41 (CH_2), 41.64 (C-3), 77.17 (C-1), 79.38 (C-2).

(1*R*,2*R*,6*S*)-6-Benzyl-2-((*tert*-butyldimethylsilyl)oxy)cyclohexan-1-ol ((*R*)-14e). According to general procedure J, **(*R*)-13e** in THF (1.4 mL) was reacted with BH_3·THF (1.4 mL) and quenched with 3 N NaOH (4.2 mL) and 30% H_2O_2 (4.2 mL) to give **(*R*)-14e** (98 mg, 50%). ^1H NMR (500 MHz, $CDCl_3$): δ = 0.09, 0.12 (2 s, 6H, $Si(CH_3)_2$), 0.84-0.95 (m, 10H, $SiC(CH_3)_3$, H-4a), 1.16 (qt, *J* = 13.2, 3.3 Hz, 1H, H-5a), 1.27 (m, 1H, H-6a), 1.49-1.68 (m, 3H, H-3, H-4b, H-5b), 1.82 (m, 1H, H-6b), 2.37 (dd, *J* = 13.4, 9.4 Hz, 1H, CH_2), 2.62 (d, *J* = 1.5 Hz, 1H, OH), 3.09 (ddd, *J* = 10.0, 8.4, 1.4 Hz, 1H, H-2), 3.22 (dd, *J* = 13.4, 3.4 Hz, 1H, CH_2'), 3.42 (ddd,

J = 11.0, 8.4, 4.5 Hz, 1H, H-1), 7.15-7.21, 7.24-7.29 (m, 5H, C_6H_5); ^{13}C NMR (126 MHz, $CDCl_3$): δ = -4.68, -3.97 ($Si(CH_3)_2$), 18.04 ($Si\mathit{C}(CH_3)_3$), 23.26 (C-5), 25.85 ($SiC(\mathit{C}H_3)_3$), 29.37 (C-4), 33.62 (C-6), 38.47 (CH_2), 43.61 (C-3), 77.13 (C-1), 78.81 (C-2), 125.69, 128.06, 128.23, 128.40, 129.58, 140.55 (C_6H_5); ESI-MS: *m/z*: Calcd for $C_{19}H_{32}NaO_2Si$ $[M+Na]^+$: 343.2; found: 343.3.

(1*R*,2*R*,6*R*)-2-((*tert*-Butyldimethylsilyl)oxy)-6-(cyclohexylmethyl)cyclohexan-1-ol ((*R*)-14f). According to general procedure J, **(*R*)-13f** (0.719 mmol) in THF (2.0 mL) was reacted with BH_3·THF (2.0 mL) and quenched with 3 N NaOH (6.0 mL) and 30% H_2O_2 (6.0 mL) to give **(*R*)-14f** (162 mg, 69%). ^1H NMR (500 MHz, $CDCl_3$): δ = 0.08, 0.10 (2 s, 6H, $Si(CH_3)_2$), 0.75-1.01 (m, 4H, H-4a, C_7H_{13}), 0.90 (s, 9H, $SiC(CH_3)_3$),1.08-1.38 (m, 6H, H-5a, H-6a, C_7H_{13}), 1.42 (dddd, J = 15.6, 13.7, 7.3, 3.7 Hz, 1H, H-3), 1.59-1.81 (m, 8H, H-4b, H-5b, C_7H_{13}), 1.85 (m, 1H, H-6b), 2.50 (d, J = 1.5 Hz, 1H, OH), 2.97 (ddd, J = 9.9, 8.5, 1.3 Hz, 1H, H-2), 3.37 (ddd, J = 10.7, 8.5, 4.5 Hz, 1H, H-1); ^{13}C NMR (126 MHz, $CDCl_3$): δ = -4.73, -3.95 ($Si(CH_3)_2$), 18.00 ($Si\mathit{C}(CH_3)_3$), 23.48 (C-5), 25.82 ($SiC(\mathit{C}H_3)_3$), 30.11 (C-4), 33.60 (C-6), 26.32, 26.54, 26.72, 32.43, 34.70, 34.94 (C_6H_{11}), 38.83 (C-3), 77.11 (C-1), 80.06 (C-2); ESI-MS: *m/z*: Calcd for $C_{19}H_{28}NaO_2Si$ $[M+Na]^+$: 394.2; found: 394.0.

(1*R*,2*R*,6*R*)-2-((*tert*-Butyldimethylsilyl)oxy)-6-phenylcyclohexan-1-ol ((*R*)-14g). According to general procedure J, **(*R*)-13g** (173 mg, 0.600 mmol) in THF (1.5 mL) was reacted with BH_3·THF (1.35 mL) and quenched with 3 N NaOH (3.8 mL) and 30% H_2O_2 (3.8 mL) to give **(*R*)-14g** (128 mg, 69%). ^1H NMR (500 MHz, $CDCl_3$): δ = 0.10, 0.11 (2 s, 6H, 2 $Si(CH_3)_2$), 0.89 (s, 9H, $SiC(CH_3)_3$), 1.37-1.62 (m, 3H, H-4a, H-5a, H-6a), 1.70-1.88 (m, 2H, H-4b, H-5b), 1.96 (m, 1H, H-6b), 2.35 (d, J = 1.1 Hz, 1H, OH), 2.56 (m, 1H, H-3), 3.46-3.67 (m, 2H, H-1, H-2), 7.14-7.43 (m, 5H, C_6H_5); ^{13}C NMR (126 MHz, $CDCl_3$): δ = -4.71, -4.08 ($Si(CH_3)_2$), 18.04 ($Si\mathit{C}(CH_3)_3$), 23.94 (C-5), 25.80 ($SiC(\mathit{C}H_3)_3$), 33.29 (C-4), 33.84 (C-6), 49.37 (C-3), 76.92, 78.87 (C-1, C-2), 126.50, 127.56, 128.48, 143.10 (6C, C_6H_5).

(1*R*,2*R*,6*R*)-2-(*tert*-Butyl)-6-((*tert*-butyldimethylsilyl)oxy)cyclohexan-1-ol ((*R*)-14h). According to general procedure J, **(*R*)-13h** (230 mg, 0.843 mmol) in THF (2.0 mL) was reacted with BH_3·THF (1.9 mL) and quenched with 3 N NaOH (5.4 mL) and 30% H_2O_2 (5.4 mL) to give **(*R*)-14h** (128 mg, 53%). ^1H NMR (500 MHz, $CDCl_3$): δ = 0.09, 0.10 (2 s, 6H, $Si(CH_3)_2$), 0.90 (s, 9H, $SiC(CH_3)_3$), 1.00 (s, 9H, $C(CH_3)_3$), 0.93 (m, 1H H-4a), 1.15-1.36 (m, 3H, H-3, H-5a, H-6a), 1.62-1.73 (m, 2H, H-4b, H-5b), 1.83 (ddd, J = 11.1, 4.7, 2.7 Hz, 1H, H-6b), 2.69 (d, J = 1.0 Hz, 1H, OH), 3.25 (m, 1H, H-2), 3.40 (ddd, J = 10.7, 8.3, 4.5 Hz, 1H, H-1); ^{13}C NMR (126 MHz, $CDCl_3$): δ = -4.74, -3.81 ($Si(CH_3)_2$), 18.02 ($Si\mathit{C}(CH_3)_3$), 23.60 (C-5), 25.87 ($SiC(\mathit{C}H_3)_3$), 26.45 (C-4), 29.11 ($C(\mathit{C}H_3)_3$), 32.98 ($\mathit{C}(CH_3)_3$), 33.57 (C-6), 49.92 (C-3), 77.99, 78.09 (C-1, C-2); ESI-MS: *m/z*: Calcd for $C_{16}H_{34}NaO_2Si$ $[M+Na]^+$: 309.2; found: 309.2.

(1*R*,2*S*,6*R*)-2-Butyl-6-((*tert*-butyldimethylsilyl)oxy)cyclohexan-1-ol ((*R*)-14i). According to general procedure J, **(*R*)-13i** (220 mg, 0.643 mmol) in THF (1.5 mL) was reacted with BH_3·THF (1.46 mL) and quenched with 3 N NaOH (4.1 mL) and 30% H_2O_2 (4.1 mL) to give **(*R*)-14i** (170 mg, 91%). ^1H NMR (500 MHz, $CDCl_3$): δ = 0.08, 0.09 (2 s, 6H, ($Si(CH_3)_2$), 0.83-0.99 (m, 13H, $SiC(CH_3)_3$, CH_3, H-4a), 1.08-1.43 (m,

8H, H-3, H-5a, H-6a, 5 CH_2), 1.66 (dq, *J* = 9.8, 3.0 Hz, 1H, CH_2), 1.71-1.88 (m, 3H, H-4b, H-5b, H-6b), 2.51 (d, *J* = 1.3 Hz, 1H, OH), 3.02 (ddd, *J* = 9.9, 8.4, 1.3 Hz, 1H, H-2), 3.36 (ddd, *J* = 10.7, 8.4, 4.5 Hz, 1H, H-1); ^{13}C NMR (126 MHz, $CDCl_3$): δ = -4.73, -3.98 ($Si(CH_3)_2$), 14.14 (CH_3), 18.02 ($Si\mathit{C}(CH_3)_3$), 23.13 (CH_2), 23.52 (C-5), 25.83 ($SiC(\mathit{C}H_3)_3$), 28.84 (CH_2), 29.83 (C-4), 31.82 (CH_2), 33.64 (C-6), 41.84 (C-3), 77.17 (C-1), 79.37 (C-2); ESI-MS: *m/z*: Calcd for $C_{16}H_{34}NaO_2Si$ $[M+Na]^+$: 309.2; found: 309.4.

(1*R*,2*R*,6*S*)-2-((*tert*-Butyldimethylsilyl)oxy)-6-hexylcyclohexan-1-ol ((*R*)-14j). According to general procedure J, **(*R*)-13j** (600 mg, 2.02 mmol) in THF (4.5 mL) was reacted with BH_3·THF (4.5 mL) and quenched with 3 N NaOH (13 mL) and 30% H_2O_2 (13 mL) to give **(*R*)-14j** (470 mg, 74%). ^1H NMR (500 MHz, $CDCl_3$): δ = 0.08, 0.09 (2 s, 6H, $Si(CH_3)_2$), 0.80-0.98 (m, 14H, $SiC(CH_3)_3$, CH_3, CH_2), 1.06-1.41 (m, 13H, 4 CH_2, H-3, H-4a, H-5a, H-6a), 1.66 (dq, *J* = 9.7, 3.0 Hz, 1H, H-5b), 1.69-1.90 (m, 2H, H-4b, H-6b), 2.51 (d, *J* = 1.3 Hz, 1H, OH), 3.02 (ddd, *J* = 9.9, 8.3, 1.3 Hz, 1H, H-2), 3.37 (ddd, *J* = 10.7, 8.4, 4.6 Hz, 1H, H-1); ^{13}C NMR (126 MHz, $CDCl_3$): δ = -4.72, -3.97 ($Si(CH_3)_2$), 14.11 (CH_3), 18.02 ($Si\mathit{C}(CH_3)_3$), 22.68 (C-5), 23.53 (CH_2), 25.83 ($SiC(\mathit{C}H_3)_3$), 26.57, 29.76, 29.82, 31.93 (4 CH_2), 32.14 (C-4), 33.64 (C-6), 41.87 (C-3), 77.18 (C-1), 79.37 (C-2).

(1*R*,2*R*,6*S*)-2-((*tert*-Butyldimethylsilyl)oxy)-6-(3-phenylpropyl)cyclohexan-1-ol ((*R*)-14k). According to general procedure J, **(*R*)-13k** (48 mg, 0.145 mmol) in THF (300 µL) was reacted with BH_3·THF (280 µL) and quenched with 3 N NaOH (844 µL) and 30% H_2O_2 (844 µL) to give **(*R*)-14k** (50 mg, quant). ^1H NMR (500 MHz, $CDCl_3$): δ = 0.08, 0.10 (2 s, 6H, $Si(CH_3)_2$), 0.81-1.00 (m, 10H, $SiC(CH_3)_3$, H-4a), 1.16-1.43 (m, 4H, H-3, H-5a, H-6a, CH_2), 1.50-1.92 (m, 6H, H-4b, H-5b, H-6b, 3 CH_2), 2.51 (d, *J* = 1.4 Hz, 1H, OH), 2.60 (tq, *J* = 19.9, 7.0, 6.2 Hz, 2H, CH_2), 3.02 (ddd, *J* = 10.0, 8.4, 1.4 Hz, 1H, H-2), 3.37 (ddd, *J* = 10.4, 8.2, 4.4 Hz, 1H, H-1), 7.13-7.20, 7.23-7.29 (m, 5H, C_6H_5); ^{13}C NMR (126 MHz, $CDCl_3$): δ = -4.71, -3.96 ($Si(CH_3)_2$), 18.03 ($Si\mathit{C}(CH_3)_3$), 23.49 (C-5), 25.84 ($SiC(\mathit{C}H_3)_3$), 28.75 (CH_2), 29.83 (C-4), 32.03 (C-6), 33.60, 36.49 (2 CH_2), 41.82 (C-3), 77.14 (C-1), 79.27 (C-2), 125.57, 128.22, 128.37, 142.90 (6C, C_6H_5); ESI-MS: *m/z*: Calcd for $C_{21}H_{36}NaO_2Si$ $[M+Na]^+$: 371.2; found: 371.1.

(1*R*,2*R*,6*R*)-2-((*tert*-Butyldimethylsilyl)oxy)-6-((2-methoxyethoxy)methyl)cyclohexan-1-ol ((*R*)-14l). According to general procedure J, **(*R*)-13l** (129 mg, 0.429 mmol) in THF (1 mL) was reacted with BH_3·THF (1 mL) and quenched with 3 N NaOH (3 mL) and 30% H_2O_2 (3 mL) to give **(*R*)-14l** (97 mg, 71%). ^1H NMR (500 MHz, $CDCl_3$): δ = 0.09, 0.10 (2 s, 6H, $Si(CH_3)_2$), 0.89 (s, 9H, $SiC(CH_3)_3$), 1.10 (qd, *J* = 12.8, 3.8 Hz, 1H, H-4a), 1.20-1.36 (m, 2H, H-5a, H-6a), 1.60-1.72 (m, 2H, H-3, H-5b), 1.77 (dt, *J* = 14.2, 2.9 Hz, 1H, H-4b), 1.85 (m, 1H, H-6b), 2.83 (s, 1H, OH), 3.20 (t, *J* = 9.3 Hz, 1H, H-2), 3.38 (s, 3H, OCH_3), 3.41 (m, 1H, H-1), 3.49 (dd, *J* = 9.4, 6.4 Hz, 1H, H-3'a), 3.54 (dd, *J* = 5.6, 4.3 Hz, 2H, H-3''), 3.56-3.63 (m, 2H, H-3'''), 3.65 (dd, *J* = 9.4, 4.7 Hz, 1H, H-3'b); ^{13}C NMR (126 MHz, $CDCl_3$): δ = -4.62, -4.18 ($Si(CH_3)_2$), 18.07 ($Si\mathit{C}(CH_3)_3$), 23.16 (C-5), 25.85 ($SiC(\mathit{C}H_3)_3$), 27.84 (C-4), 33.61 (C-6), 42.45 (C-3), 59.04 (OCH_3), 70.51 (C-3'''), 71.88 (C-3''), 73.73 (C-3'), 76.80 (C-1), 77.14 (C-2); ESI-MS: *m/z*: Calcd for $C_{16}H_{34}NaO_4Si$ $[M+Na]^+$: 341.2; found: 341.1.

(1*R*,2*R*,6*R*)-2-((*tert*-Butyldimethylsilyl)oxy)-6-isobutylcyclohexan-1-ol ((*R*)-14m). According to general procedure J, **(*R*)-13m** (174 mg, 0.648 mmol) in THF (1.5 mL) was reacted with BH_3·THF (1.5 mL) and quenched with 3 N NaOH (4.5 mL) and 30% H_2O_2 (4.5 mL) to give **(*R*)-14m** (100 mg, 54%). ^{1}H NMR (500 MHz, $CDCl_3$): δ = 0.08, 0.10 (2 s, 6H, $Si(CH_3)_2$), 0.84 (d, *J* = 6.2 Hz, 3H, CH_3), 0.87-0.92 (m, 13H, $SiC(CH_3)_3$, CH_3, H-4a), 1.02 (m, 1H, CH_2), 1.17-1.45 (m, 3H, H-3, H-5a, H-6a), 1.58-1.73 (m, 3H, H-5b, CH, CH_2), 1.77 (dt, *J* = 13.8, 2.8 Hz, 1H, H-4b), 1.84 (ddt, *J* = 10.9, 4.4, 2.4 Hz, 1H, H-6b), 2.51 (d, *J* = 1.3 Hz, 1H, OH), 2.97 (ddd, *J* = 9.9, 8.4, 1.3 Hz, 1H, H-2), 3.38 (ddd, *J* = 10.6, 8.4, 4.5 Hz, 1H, H-1); ^{13}C NMR (126 MHz, $CDCl_3$): δ = -4.72, -3.95 ($Si(CH_3)_2$), 18.01 (Si*C*(CH_3)$_3$), 21.50 (CH_3), 23.49 (C-5), 24.25 (CH_3), 25.03 (CH), 25.83 (SiC(*C*H_3)$_3$), 29.93 (C-4), 33.60 (C-6), 39.58 (C-3), 41.87 (CH_2), 77.10 (C-1), 80.08 (C-2); ESI-MS: *m/z*: Calcd for $C_{16}H_{34}NaO_2Si$ $[M+Na]^+$: 309.2; found: 309.2.

(1*R*,2*R*,6*R*)-2-((*tert*-Butyldimethylsilyl)oxy)-6-(2,2,2-trifluoroethyl)cyclohexan-1-ol ((*R*)-14n). According to general procedure J, **(*R*)-13m** (52 mg, 0.176 mmol) in THF (600 µL) was reacted with BH_3·THF (441 µL) and quenched with 3 N NaOH (1.27 mL) and 30% H_2O_2 (1.27 mL) to give **(*R*)-14n** (28 mg, 50%). ^{1}H NMR (500 MHz, $CDCl_3$): δ = 0.09, 0.10 (2 s, 6H, $Si(CH_3)_2$), 0.90 (s, 9H, $SiC(CH_3)_3$), 1.07 (m, 1H), 1.26-1.34 (m, 2H), 1.66-1.75 (m, 2H), 1.80-1.90 (m, 2H, C*H*$_2$CF$_3$), 1.98 (d, *J* = 13.7 Hz, 1H), 2.54 (d, *J* = 1.3 Hz, 1H, OH), 2.68-2.84 (m, 1H, C*H*$_2$CF$_3$), 3.02 (t, *J* = 9.3 Hz, 1H, H-2), 3.39 (m, 1H, H-1); ^{19}F NMR (471 MHz, $CDCl_3$): δ = -62.92.

Syntheses of sialyl Lewis^x mimetics 2b-n

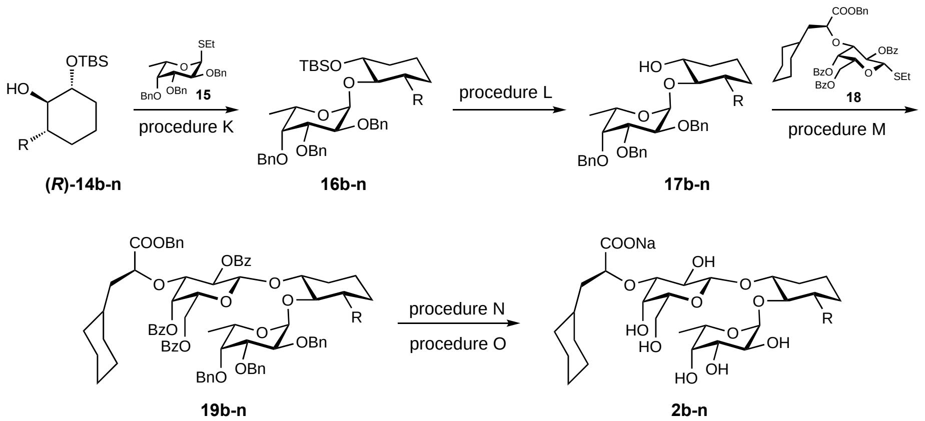

Scheme 5. Synthesis of selectin antagonists **2b-n**.

General procedure K:

[(1*R*,2*R*,3*S*)-1-((*tert*-Butyldimethylsilyl)oxy)-3-methylcyclohex-2-yl] 2,3,4-tri-*O*-benzyl-6-deoxy-α-L-galactopyranoside (16b).[6] Ethylthio fucoside **15**[7] (3.90 g, 8.15 mmol) and tetrabutylammonium bromide (TBAB, 4.00 g, 12.4 mmol) were dried at high vacuum overnight. Powdered activated molecular sieves 4 Å (5.0 g), compound **(*R*)-14b** (1.00 g, 4.09 mmol), 2,6-di-*tert*-butyl-4-methylpyridine (2.50 g, 12.2 mmol),

anhydrous DCM (35 ml) and DMF (5 ml) were added and the mixture was stirred for 4 h at rt under argon. $CuBr_2$ (2.70 g, 12.1 mmol), dried under high vacuum overnight at 70 °C, was added and the resulting dark mixture was stirred at rt under argon. After completion of the reaction (17 h), the solution was filtered through a pad of celite and the filtrate was washed with a solution of satd. aq. NH_4Cl and aq. NH_3 (9:1 (v/v), 2 x 200 mL) and brine (100 mL). The combined aqeous layers were extracted with DCM (2 x 200 mL) and the combined organic layers were dried (Na_2SO_4) and concentrated. Column chromatography on silica (petroleum ether/EtOAc, 98:2 to 97:3) gave the pseudodisaccharide **16b** (2.34 g, 3.54 mmol, 87%) as clear oil. $[\alpha]_D^{20}$ -53.7 (*c* 2.1, $CHCl_3$); ^{1}H NMR (500 MHz, $CDCl_3$): δ = 0.06, 0.09 (2 s, 6H, $SiCH_3$), 0.93 (s, 9H, $SiC(CH_3)_3$), 1.11-1.34 (m, 8H, H-6^F, CH_3, H-4b^{cyc}, H-5b^{cyc}), 1.43 (m, 1H, H-6b^{cyc}), 1.68-1.76 (m, 2H, H-4a^{cyc}, H-5a^{cyc}), 1.77-1.88 (m, 2H, H-3^{cyc}, H-6a^{cyc}), 3.36 (t, *J* = 6.4 Hz, H-2^{cyc}), 3.70 (m, 1H, H-4^F,), 3.75 (m, 1H, H-1^{cyc}), 4.05 (dd, *J* = 2.6, 10.2 Hz, 1H, H-3^F), 4.10 (dd, *J* = 3.4, 10.2 Hz, 1H, H-2^F), 4.26 (q, *J* = 6.4 Hz, 1H, H-5^F), 4.70 (A of AB, *J* = 11.6 Hz, 1H, CH_2Ph), 4.76, 4.78, 4.85, 4.89 (4d, *J* = 11.8 Hz, 4H, CH_2Ph), 5.03 (B of AB, *J* = 11.6 Hz, 1H, CH_2Ph), 5.16 (d, *J* = 3.4 Hz, 1H, H-1^F), 7.27-7.47 (m, 15H, 3 C_6H_5); ^{13}C NMR (126 MHz, $CDCl_3$): δ = -5.0, -3.9 (2C, 2 Si*C*H_3), 17.0 (CH_3), 18.2 (Si*C*$(CH_3)_3$), 18.9 (C-6^F), 19.8 (C-4^{cyc}), 26.1 (3C, SiC(*C*$H_3)_3$), 31.1 (C-5^{cyc}), 33.2 (C-6^{cyc}), 35.6 (C-3^{cyc}), 66.4 (C-5^F), 73.0 (*C*H_2Ph), 73.3 (C-1^{cyc}), 73.6 (*C*H_2Ph), 74.9 (*C*H_2Ph), 76.7 (C-2^F), 78.2 (C-4^F), 79.3, 81.5 (C-2^{cyc}, C-3^F), 96.8 (C-1^F), 127.5, 127.6, 128.2, 128.3, 128.4, 138.8, 138.9, 139.1 (18C, 3 C_6H_5); HR-MS (ESI): *m/z*: Calcd for $C_{40}H_{56}NaO_6Si^+$ $[M+Na]^+$: 683.3738; found: 683.3740.

[(1*R*,2*R*,3*S*)-1-((*tert*-Butyldimethylsilyl)oxy)-3-ethylcyclohex-2-yl] 2,3,4-tri-*O*-benzyl-6-deoxy-α-L-galactopyranoside (16c). According to general procedure K, **15** (5.56 g, 11.5 mmol), **(*R*)-14c** (1.49 g, 5.77 mmol), TBAB (5.58 g, 17.3 mmol) and 2,6-di-*tert*-butyl-4-methylpyridine (3.55 g, 17.3 mmol) in DCM/DMF (49:7 mL) were reacted with $CuBr_2$ (3.86 g, 15.0 mmol) to give **16c** (2.35 g, 60%). ^{1}H NMR (500 MHz, $CDCl_3$): δ = 0.01, 0.03 (2 s, 6H, $Si(CH_3)_2$), 0.84-0.89 (m, 12H, $SiC(CH_3)_3$, CH_2C*H*$_3$), 1.10 (d, *J* = 6.5 Hz, 2H, H-6^F), 1.17-1.28 (m, 2H, H-4b^{cyc}, H-6b^{cyc}), 1.39-1.50 (m, 2H, H-4a^{cyc}, H-5b^{cyc}), 1.55 (m, 1H, H-3^{cyc}), 1.63-1.81 (m, 4H, C*H*$_2CH_3$, H-6a^{cyc}, H-5a^{cyc}), 3.41 (t, *J* = 4.7 Hz, 1H, H-2^{cyc}), 3.66 (d, *J* = 1.9 Hz, 1H, H-4^F), 3.76 (m, 1H, H-1^{cyc}), 3.96 (dd, *J* = 10.2, 2.7 Hz, 1H, H-3^F), 4.03 (dd, *J* = 10.2, 3.6 Hz, 1H, H-2^F), 4.08 (q, *J* = 6.5 Hz, 1H, H-5^F), 4.63-4.69 (m, 2H, CH_2Ph), 4.74 (d, *J* = 11.8 Hz, 1H, A of AB, CH_2Ph), 4.79 (d, *J* = 11.8 Hz, 1H, B of AB, CH_2Ph), 4.86 (d, *J* = 11.8 Hz, 1H, B of AB, CH_2Ph), 4.97 (d, *J* = 4.3 Hz, 1H, H-1^F), 4.99 (d, *J* = 12.1 Hz, 1H, B of AB, CH_2Ph), 7.21-7.45 (m, 15H, 3 C_6H_5).

[(1*R*,2*R*,3*S*)-1-((*tert*-Butyldimethylsilyl)oxy)-3-propylcyclohex-2-yl] 2,3,4-tri-*O*-benzyl-6-deoxy-α-L-galactopyranoside (16d). According to general procedure K, **15** (702 mg, 1.47 mmol), **(*R*)-14d** (200 mg, 0.73 mmol), TBAB (709 mg, 2.20 mmol) and 2,6-di-*tert*-butyl-4-methylpyridine (452 mg, 2.20 mmol) in DCM/DMF (7.2:0.8 mL) were reacted with $CuBr_2$ (492 mg, 2.02 mmol) to give **16d** (209 mg, 41%). $[\alpha]_D^{20}$ -84.8 (*c* 0.86, DCM); ^{1}H NMR (500 MHz, $CDCl_3$): δ = -0.01, 0.00 (2 s, 6H, $Si(CH_3)_2$), 0.80-0.90 (m, 12H, $SiC(CH_3)_3$, CH_3), 1.07 (d, *J* = 6.4 Hz, 3H, H-6^F), 1.12-1.47, 1.58-1.79 (m, 11H, H-3^{cyc}, H-4^{cyc}, H-5^{cyc}, H-6^{cyc}, 2 CH_2), 3.39 (t, *J* = 4.2 Hz, 1H, H-2^{cyc}), 3.63 (d, *J* = 2.7 Hz, 1H, H-4^F), 3.74 (td, *J* = 4.9, 2.6 Hz, 1H, H-1^{cyc}), 3.93 (dd, *J* = 10.1, 2.8 Hz, 1H, H-3^F), 3.97-4.06 (m, 2H, H-2^F, H-5^F), 4.62 (d, *J* = 11.8 Hz, 1H, A of AB,

CH_2Ph), 4.65 (d, *J* = 12.3 Hz, 1H, A of AB, CH_2Ph), 4.71 (d, *J* = 11.8 Hz, 1H, A of AB, CH_2Ph), 4.75 (d, *J* = 11.9 Hz, 1H, B of AB, CH_2Ph), 4.83 (d, *J* = 11.8 Hz, 1H, B of AB, CH_2Ph), 4.92-4.98 (m, 2H, H-1^{F}, CH_2Ph), 7.14-7.45 (m, 15H, 3 C_6H_5); ^{13}C NMR (126 MHz, $CDCl_3$): δ = -5.11, -4.51 ($Si(CH_3)_2$), 14.31 (CH_3), 16.78 (C-6^{F}), 17.29 (CH_2), 18.10 ($Si\mathit{C}(CH_3)_3$), 21.02 (C-5^{cyc}), 25.93 ($SiC(\mathit{C}H_3)_3$), 26.30 (CH_2), 31.36 (C-4^{cyc}), 33.29 (C-6^{cyc}), 38.27 (C-3^{cyc}), 66.43 (C-5^{F}), 71.68 (C-1^{cyc}), 73.08, 73.17, 74.80 (3 CH_2Ph), 76.72 (C-2^{F}), 77.96 (C-4^{F}), 79.00 (C-2^{cyc}), 79.30 (C-3^{F}), 96.66 (C-1^{F}), 127.38, 127.44, 127.48, 127.50, 127.90, 128.16, 128.25, 128.32, 128.37, 138.81, 138.90, 139.10 (18C, 3 C_6H_5); ESI-MS: *m/z*: Calcd for $C_{42}H_{60}NaO_6Si$ $[M+Na]^+$: 711.4; found: 711.6.

[(1*R*,2*R*,3*R*)-3-Benzyl-1-((*tert*-butyldimethylsilyl)oxy)cyclohex-2-yl] 2,3,4-tri-*O*-benzyl-6-deoxy-α-L-galactopyranoside (16e). According to general procedure K, **15** (260 mg, 0.54 mmol), **(*R*)-14e** (87 mg, 0.271 mmol), TBAB (263 mg, 0.814 mmol) and 2,6-di-*tert*-butyl-4-methylpyridine (267 mg, 0.814 mmol) in DCM/DMF (2.8:0.4 mL) were reacted with $CuBr_2$ (182 mg, 0.814 mmol) to give **16e** (117 mg, 58%). $[\alpha]_D^{20}$ -162.3 (c 0.72, DCM); ^{1}H NMR (500 MHz, $CDCl_3$): δ = 0.03, 0.06 (2 s, 6H, $Si(CH_3)_2$), 0.90 (s, 9H, $SiC(C\mathit{H}_3)_3$), 1.10 (d, *J* = 6.5 Hz, 3H, H-6^{F}), 1.04-1.23 (m, 2H, H-5b^{cyc}, H-4b^{cyc}), 1.48 (m, 1H, H-6b^{cyc}), 1.59 (m, 1H, H-4a^{cyc}), 1.68-1.83 (m, 2H, H-5a^{cyc}, H-6a^{cyc}), 1.97 (m, 1H, H-3^{cyc}), 2.73 (dd, *J* = 13.3, 11.4 Hz, 1H, $PhCH_2^{cyc}$), 2.98 (dd, *J* = 13.7, 4.4 Hz, 1H, $PhCH_2^{cyc}$), 3.48 (t, *J* = 4.5 Hz, 1H, H-2^{cyc}), 3.66 (s, 1H, H-4^{F}), 3.81 (m, 1H, H-1^{cyc}), 3.96 (dd, *J* = 10.1, 2.6 Hz, 1H, H-3^{F}), 4.00-4.09 (m, 2H, H-5^{F}, H-2^{F}), 4.62 (d, *J* = 11.7 Hz, 1H, A of AB, CH_2Ph), 4.65 (d, *J* = 11.6 Hz, 1H, A of AB, CH_2Ph), 4.73 (d, *J* = 11.8 Hz, 1H, A of AB, CH_2Ph), 4.78 (d, *J* = 11.7 Hz, 1H, B of AB, CH_2Ph), 4.84 (d, *J* = 11.8 Hz, 1H, B of AB, CH_2Ph), 4.98-5.01 (m, 2H, H-1^{F}, CH_2Ph), 7.09-7.40 (m, 20H, 4 C_6H_5); ^{13}C NMR (126 MHz, $CDCl_3$): δ = -4.45, -5.06 ($Si(CH_3)_2$), 16.75 (C-6^{F}), 17.16 (C-5^{cyc}), 18.15 ($Si\mathit{C}(CH_3)_3$), 24.93 (C-4^{cyc}), 26.00 ($SiC(\mathit{C}H_3)_3$), 31.42 (C-6^{cyc}), 37.50 ($PhCH_2^{cyc}$), 40.53 (C-3^{cyc}), 66.58 (C-5^{F}), 71.88 (C-1^{cyc}), 73.00, 73.38, 74.77 (3 CH_2Ph), 76.86 (C-2^{F}), 77.89 (C-4^{F}), 79.13 (C-3^{F}), 80.25 (C-2^{cyc}), 97.03 (C-1^{F}), 125.56, 127.37, 127.44, 127.47, 127.50, 127.91, 128.12, 128.15, 128.23, 128.29, 128.35, 129.12, 138.75, 138.98, 142.07 (24C, 4 C_6H_5); ESI-MS: *m/z*: Calcd for $C_{46}H_{60}NaO_6Si$ $[M+Na]^+$: 759.4; found: 759.5

[(1*R*,2*R*,3*R*)-1-((*tert*-Butyldimethylsilyl)oxy)-3-(cyclohexylmethyl)cyclohex-2-yl] 2,3,4-tri-*O*-benzyl-6-deoxy-α-L-galactopyranoside (16f). According to general procedure K, **15** (475 mg, 0992 mmol), **(*R*)-14f** (162 mg, 0.496 mmol), TBAB (480 mg, 1.49 mmol) and 2,6-di-*tert*-butyl-4-methylpyridine (305 mg, 1.49 mmol) in DCM/DMF (5.0:0.7 mL) were reacted with $CuBr_2$ (332 mg, 1.49 mmol) to give **16f** (244 mg, 66%). $[\alpha]_D^{20}$ -73.3 (c 0.48, DCM); ^{1}H NMR (500 MHz, $CDCl_3$): δ = 0.02 (d, *J* = 2.9 Hz, 6H), 0.70-0.94 (m, 12H), 1.10 (d, *J* = 6.5 Hz, 3H, H-6^{F}), 1.13-1.24 (m, 5H), 1.36 (m, 1H), 1.44 (m, 1H), 1.52 (m, 1H), 1.60-1.90 (m, 9H), 3.39 (t, *J* = 3.7 Hz, 1H, H-2^{cyc}), 3.67 (d, *J* = 1.8 Hz, 1H, H-4^{F}), 3.79 (m, 1H, H-1^{cyc}), 3.95 (dd, *J* = 10.1, 2.8 Hz, 1H, H-3^{F}), 3.99 (q, *J* = 6.6 Hz, 1H, H-5^{F}), 4.02 (dd, *J* = 10.1, 3.6 Hz, 1H, H-2F), 4.65 (d, *J* = 11.6 Hz, 1H, A of AB, CH_2Ph), 4.67 (d, *J* = 11.8 Hz, 1H, A of AB, CH_2Ph), 4.74 (d, *J* = 11.8 Hz, 1H, A of AB, CH_2Ph), 4.77 (d, *J* = 11.8 Hz, 1H, B of AB, CH_2Ph), 4.86 (d, *J* = 11.8 Hz, 1H, B of AB, CH_2Ph), 4.97-4.99 (m, 2H, H-1^{F}, CH_2Ph), 7.12-7.54 (m, 15H, 3 C_6H_5); ^{13}C NMR (126 MHz, $CDCl_3$): δ = -4.68, -5.14 ($Si(CH_3)_2$), 16.71 (C-

6^F), 18.07 (Si*C*(CH$_3$)$_3$), 25.86, 25.89, 26.37, 26.54, 26.80, 32.96, 33.96, 34.14, 35.32, 38.50 (15C), 66.43 (C-5^F), 71.20 (C-1^{cyc}), 73.04 (2C, 2 CH$_2$Ph), 74.74 (CH$_2$Ph), 76.71 (C-2^F), 77.83 (C-4^F), 79.26, 79.31 (C-3^F, C-2^{cyc}), 96.64 (C-1^F), 127.35, 127.40, 127.48, 127.76, 128.13, 128.21, 128.29, 128.37, 138.75, 139.08 (18C, 3 C$_6$H$_5$); ESI-MS: *m/z*: Calcd for $C_{46}H_{66}NaO_6Si$ [M+Na]$^+$: 765.5; found: 765.5.

[(1*R*,2*R*,3*R*)-1-((*tert*-Butyldimethylsilyl)oxy)-3-phenylcyclohex-2-yl] 2,3,4-tri-*O*-benzyl-6-deoxy-α-L-galactopyranoside (16g). According to general procedure K, **15** (156 mg, 0.326 mmol), **(*R*)-14g** (50 mg, 0.163 mmol), TBAB (158 mg, 0.489 mmol) and 2,6-di-*tert*-butyl-4-methylpyridine (100 mg, 0.489 mmol) in DCM/DMF (2 mL) were reacted with CuBr$_2$ (109 mg, 0.489 mmol) to give **16g** (111 mg, 95%). $[\alpha]_D^{20}$ -68.6 (*c* 2.4, DCM); ^{1}H NMR (500 MHz, CDCl$_3$): δ = 0.03, 0.14 (2 s, 6H, Si(CH$_3$)$_2$), 0.91 (s, 9H, SiC(CH$_3$)$_3$), 1.11 (d, *J* = 6.5 Hz, 3H, H-6^F), 1.33-1.67 (m, 3H, H-4a^{cyc}, H-5a^{cyc}, H-6a^{cyc}), 1.68-1.78 (m, 2H, H-4b^{cyc}, H-5b^{cyc}), 2.01 (m, 1H, H-6b^{cyc}), 2.77 (m, 1H, H-3^{cyc}), 3.60 (dd, *J* = 2.9, 1.3 Hz, 1H, H-4^F), 3.66 (dd, *J* = 10.2, 3.6 Hz, 1H, H-2^F), 3.84 (ddd, *J* = 10.9, 8.3, 4.4 Hz, 1H, H-1^{cyc}), 3.92-4.03 (m, 2H, H-3^F, H-2^{cyc}), 4.26 (d, *J* = 12.4 Hz, 1H, A of AB, CH$_2$Ph), 4.36 (d, *J* = 3.7 Hz, 1H, H-1^F), 4.48-4.54 (m, 2H, H-5^F, CH$_2$Ph), 4.57 (d, *J* = 11.4 Hz, 1H, A of AB, CH$_2$Ph), 4.67 (d, *J* = 11.8 Hz, 1H, A of AB, CH$_2$Ph), 4.80 (d, *J* = 11.8 Hz, 1H, B of AB, CH$_2$Ph), 4.89 (d, *J* = 11.4 Hz, 1H, B of AB, CH$_2$Ph), 7.21-7.46 (m, 20H, 4 C$_6$H$_5$); ^{13}C NMR (126 MHz, CDCl$_3$): δ = -5.02, -3.06 (Si(CH$_3$)$_2$), 16.85 (C-6^F), 18.16 (Si*C*(CH$_3$)$_3$), 23.69 (C-5^{cyc}), 26.11 (SiC(*C*H$_3$)$_3$), 35.51 (C-4^{cyc}), 35.80 (C-6^{cyc}), 50.72 (C-3^{cyc}), 65.43 (C-5^F), 72.31, 72.94, 74.98 (3 CH$_2$Ph), 75.38 (C-1^{cyc}), 75.74 (C-2^F), 77.24 (C-4^F), 78.54, 78.76 (C-3^F, C-2^{cyc}), 93.86 (C-1^F), 126.41, 127.23, 127.37, 127.88, 128.06, 128.22, 128.33, 128.75, 138.67, 138.81, 139.19, 144.31 (24C, 4 C$_6$H$_5$); ESI-MS: *m/z*: Calcd for $C_{45}H_{58}NaO_6Si$ [M+Na]$^+$: 745.4; found: 745.5.

[(1*R*,2*R*,3*R*)-3-*tert*-Butyl-1-((*tert*-butyldimethylsilyl)oxy)cyclohex-2-yl] 2,3,4-tri-*O*-benzyl-6-deoxy-α-L-galactopyranoside (16h). According to general procedure K, **15** (167 mg, 0.349 mmol), **(*R*)-14h** (50 mg, 0.175 mmol), TBAB (168 mg, 0.524 mmol) and 2,6-di-*tert*-butyl-4-methylpyridine (107 mg, 0.524 mmol) in DCM/DMF (2 mL) were reacted with CuBr$_2$ (116 mg, 0.524 mmol) to give **16h** (45 mg, 37%, slightly impure). ^{1}H NMR (500 MHz, CDCl$_3$): δ= 0.03, 0.04 (2 s, 6H, Si(CH$_3$)$_2$), 0.87 (s, 9H, SiC(CH$_3$)$_3$), 0.92 (s, 9H, C(CH$_3$)$_3$), 1.09 (d, *J* = 6.6 Hz, 3H, H-6^F), 1.34-1.55, 1.58-1.79 (m, 7H, H-3^{cyc}, H-4^{cyc}, H-5^{cyc,} H-6^{cyc}), 3.52 (m, 1H, H-2^{cyc}), 3.67 (dd, *J* = 3.0, 1.2 Hz, 1H, H-4^F), 3.83-3.94 (m, 2H, H-3^F, H-5^F), 4.01 (dd, *J* = 10.2, 3.7 Hz, 1H, H-2^F), 4.04 (m, 1H, H-1^{cyc}), 4.62-4.71 (m, 2H, CH$_2$Ph), 4.71-4.77 (m, 2H, CH$_2$Ph), 4.84 (m, 1H, CH$_2$Ph), 4.95-5.00 (m, 2H, H-1^F, CH$_2$Ph), 7.21-7.40 (m, 15H, 3 C$_6$H$_5$); ^{13}C NMR (126 MHz, CDCl$_3$): δ = -4.98, -4.69 (Si(CH$_3$)$_2$), 16.62 (C-6^F), 17.93 (C-5^{cyc}), 18.06 (Si*C*(CH$_3$)$_3$), 19.51, 30.14 (C-4^{cyc}, C-6^{cyc}), 25.90 (SiC(*C*H$_3$)$_3$) , 28.86 (C(*C*H$_3$)$_3$), 32.78 (*C*(CH$_3$)$_3$), 49.14 (C-3^{cyc}), 66.56 (C-5^F), 69.75 (C-1^{cyc}), 72.84, 73.39, 74.69 (3 CH$_2$Ph), 76.49 (C-2^F), 77.58 (C-4^F), 79.33 (C-3^F), 80.47 (C-2^{cyc}), 98.08 (C-1^F), 127.33, 127.38, 127.49, 127.82, 127.92, 128.02, 128.10, 128.12, 128.14, 128.24, 128.27, 128.39, 128.43, 138.66, 138.73, 139.00 (18C, 3 C$_6$H$_5$); ESI-MS: *m/z*: Calcd for $C_{43}H_{62}NaO_6Si$ [M+Na]$^+$: 725.4; found: 725.6.

[(1*R*,2*R*,3*S*)-3-Butyl-1-((*tert*-butyldimethylsilyl)oxy)cyclohex-2-yl] 2,3,4-tri-*O*-benzyl-6-deoxy-α-L-galactopyranoside (16i). According to general procedure K, **15** (167 mg, 0.349 mmol), **(*R*)-14i** (50 mg, 0.175 mmol), TBAB (168 mg, 0.524 mmol) and 2,6-di-*tert*-butyl-4-methylpyridine (107 mg, 0.524 mmol) in DCM/DMF (2 mL) were reacted with $CuBr_2$ (116 mg, 0.524 mmol) to give **16i** (93 mg, 76%). $[\alpha]_D^{20}$ -78.5 (*c* 1.86, DCM); ^{1}H NMR (500 MHz, $CDCl_3$): δ = 0.08, 0.10 (2 s, 3H, $Si(CH_3)_2$), 0.88-1.00 (m, 12H, $SiC(CH_3)_3$, CH_3), 1.17 (d, J = 6.5 Hz, 3H, H-6^F), 1.23-1.39 (m, 6H, H-5^{cyc}, 2 CH_2), 1.44-1.60 (m, 2H, H-4a^{cyc}, H-6a^{cyc}), 1.68-1.89 (m, 5H, H-3^{cyc}, H-4b^{cyc}, H-6b^{cyc}, CH_2), 3.49 (t, J = 4.2 Hz, 1H, H-2^{cyc}), 3.73 (m, 1H, H-4^F), 3.84 (dt, J = 4.9, 2.7 Hz, 1H, H-1^{cyc}), 4.03 (dd, J = 10.1, 2.8 Hz, 1H, H-3^F), 4.08-4.14 (m, 2H, H-2^F, H-5^F), 4.69-4.77 (m, 2H, CH_2Ph), 4.80 (d, J = 11.8 Hz, 1H, A of AB, CH_2Ph), 4.84 (d, J = 11.8 Hz, 1H, B of AB, CH_2Ph), 4.92 (d, J = 11.8 Hz, 1H, B of AB, CH_2Ph), 5.03-5.08 (m, 2H, H-1^F, CH_2Ph), 7.25-7.53 (m, 15H, 3 C_6H_5); ^{13}C NMR (126 MHz, $CDCl_3$): δ = -5.15, -4.59 ($Si(CH_3)_2$), 14.17 (CH_3), 16.72 (C-6^F), 17.12, 26.10, 30.30 (3 CH_2), 18.04 (Si*C*($CH_3)_3$), 22.96 (C-5^{cyc}), 25.87 (SiC(*C*$H_3)_3$), 30.69 (C-4^{cyc}), 31.24 (C-6^{cyc}), 38.40 (C-3^{cyc}), 66.38 (C-5^F), 71.58 (C-1^{cyc}), 73.02, 73.10, 74.74 (3 CH_2Ph), 76.69 (C-2^F), 77.90 (C-4^F), 79.06 (C-3^F), 79.23 (C-2^{cyc}), 96.61 (C-1^F), 127.32, 127.37, 127.41, 127.44, 127.83, 128.10, 128.18, 128.25, 128.31, 138.74, 138.84, 139.04 (18C, 3 C_6H_5); ESI-MS: *m/z*: Calcd for $C_{43}H_{62}NaO_6Si$ $[M+Na]^+$: 725.4; found: 725.4

[(1*R*,2*R*,3*S*)-1-((*tert*-Butyldimethylsilyl)oxy)-3-hexylcyclohex-2-yl] 2,3,4-tri-*O*-benzyl-6-deoxy-α-L-galactopyranoside (16j). According to general procedure K, **15** (456 mg, 0.954 mmol), **(*R*)-14j** (150 mg, 0.477 mmol), TBAB (461 mg, 1.43 mmol) and 2,6-di-*tert*-butyl-4-methylpyridine (294 mg, 1.43 mmol) in DCM/DMF (6 mL) were reacted with $CuBr_2$ (319 mg, 1.43 mmol) to give **16j** (240 mg, 69%); $[\alpha]_D^{20}$ -70.1 (*c* 0.86, DCM); ^{1}H NMR (500 MHz, $CDCl_3$): δ = 0.01, 0.02 (2 s, 6H, $Si(CH_3)_2$), 0.85-0.90 (m, 12H, $SiC(CH_3)_3$, CH_3), 1.10 (d, J = 6.5 Hz, 3H, H-6^F), 1.16-1.35 (m, 10H, H-5a^{cyc}, 9 CH_2), 1.44 (s, 2H, H-4a^{cyc}, H-6a^{cyc}), 1.58-1.81 (m, 5H, H-3^{cyc}, H-4b^{cyc}, H-5b^{cyc}, H-6b^{cyc}, CH_2), 3.41 (t, J = 4.2 Hz, 1H, H-2^{cyc}), 3.66 (dd, J = 2.8, 1.3 Hz, 1H, H-4^F), 3.77 (td, J = 4.8, 2.6 Hz, 1H, H-1^{cyc}), 3.95 (dd, J = 10.1, 2.8 Hz, 1H, H-3^F), 4.00-4.08 (m, 2H, H-2^F, H-5^F), 4.61-4.70 (m, 2H, CH_2Ph), 4.73 (d, J = 11.8 Hz, 1H, A of AB, CH_2Ph), 4.77 (d, J = 11.8 Hz, 1H, B of AB, CH_2Ph), 4.85 (d, J = 11.7 Hz, 1H, B of AB, CH_2Ph), 4.95-5.01 (m, 2H, H-1^F, CH_2Ph), 7.22-7.43 (m, 15H, 3 C_6H_5); ^{13}C NMR (126 MHz, $CDCl_3$): δ = -5.12, -4.54 ($Si(CH_3)_2$), 14.15 (CH_3), 16.76 (C-6^F), 18.09 (Si*C*($CH_3)_3$), 22.72 (C-5^{cyc}), 25.92 (SiC(*C*$H_3)_3$), 26.14, 28.08, 29.64, 31.02 (5C, 5 CH_2), 31.29 (C-4^{cyc}), 32.02 (C-6^{cyc}), 38.49 (C-3^{cyc}), 66.41 (C-5^F), 71.62 (C-1^{cyc}), 73.07, 73.12, 74.79 (3 CH_2Ph), 76.72 (C-2^F), 77.95 (C-4^F), 79.09 (C-3^F), 79.28 (C-2^{cyc}), 96.63 (C-1^F), 127.36, 127.42, 127.45, 127.48, 127.87, 128.14, 128.23, 128.30, 128.36, 138.79, 138.89, 139.09 (18C, 3 C_6H_5). ESI-MS: *m/z*: Calcd for $C_{45}H_{66}NaO_6Si$ $[M+Na]^+$: 753.4; found: 753.4.

[(1*R*,2*R*,3*S*)-1-((*tert*-Butyldimethylsilyl)oxy)-3-(3-phenylpropyl)cyclohex-2-yl] 2,3,4-tri-*O*-benzyl-6-deoxy-α-L-galactopyranoside (16k). According to general procedure K, **15** (134 mg, 0.287 mmol), **(*R*)-14k** (50 mg, 0.144 mmol), TBAB (139 mg, 0.431 mmol) and 2,6-di-*tert*-butyl-4-methylpyridine (88 mg, 0.431 mmol) in DCM/DMF (2 mL) were reacted with $CuBr_2$ (96 mg, 0.431 mmol) to give **16k** (12 mg, 11%). $[\alpha]_D^{20}$ -65.0 (*c* 0.89, DCM); ^{1}H NMR (500 MHz, $CDCl_3$): δ = 0.04-0.06 (m, 3H, $Si(CH_3)_2$), 0.06 (s, 3H, $Si(CH_3)_2$),

0.92 (s, 9H, $SiC(CH_3)_3$), 1.13 (d, J = 6.4 Hz, 3H, H-6^F), 1.20-1.37 (m, 4H, H-5a^{cyc}, 3 CH_2), 1.48 (dt, J = 10.1, 4.2 Hz, 1H, H-6a^{cyc}), 1.59 (m, 1H, H-4a^{cyc}), 1.64-1.86 (m, 5H, H-3^{cyc}, H-4b^{cyc}, H-5b^{cyc}, H-6b^{cyc}, CH_2), 2.59 (ddq, J = 20.9, 14.0, 7.7, 6.9 Hz, 2H, CH_2), 3.44 (d, J = 4.0 Hz, 1H, H-2^{cyc}), 3.70 (m, 1H, H-4^F), 3.81 (m, 1H, H-1^{cyc}), 3.98 (m, 1H, H-3^F), 4.02-4.12 (m, 2H, H-2^F, H-5^F), 4.67-4.72 (m, 2H, CH_2Ph), 4.77 (d, J = 11.9 Hz, 1H, A of AB, CH_2Ph), 4.81 (d, J = 12.0 Hz, 1H, B of AB, CH_2Ph), 4.89 (d, J = 11.8 Hz, 1H, B of AB, CH_2Ph), 4.97-5.06 (m, 2H, H-1^F, CH_2Ph), 7.16-7.23, 7.25-7.46 (m, 20H, 4 C_6H_5); ^{13}C NMR (126 MHz, $CDCl_3$): δ = -5.08, -4.58 ($Si(CH_3)_2$), 16.76 (C-6^F), 16.92 (C-5^{cyc}), 18.11 (Si*C*$(CH_3)_3$), 25.95 (SiC(*C*$H_3)_3$), 25.97 (CH_2), 30.28, 30.71 (CH_2, C-4^{cyc}), 31.08 (C-6^{cyc}), 36.25 (CH_2), 38.28 (C-3^{cyc}), 66.48 (C-5^F), 71.44 (C-1^{cyc}), 73.08, 73.15, 74.79 (3 CH_2Ph), 76.82 (C-2^F), 77.89 (C-4^F), 79.11 (C-3^F), 79.25 (C-2^{cyc}), 96.70 (C-1^F), 125.57, 127.38, 127.42, 127.47, 127.51, 127.67, 127.83, 128.16, 128.17, 128.22, 128.24, 128.26, 128.31, 128.39, 128.43, 138.77, 138.89, 139.08, 142.85 (24C, 4 C_6H_5); ESI-MS: *m/z*: Calcd for $C_{48}H_{64}NaO_6Si$ $[M+Na]^+$: 787.4; found: 787.3.

[(1*R*,2*R*,3*R*)-1-((*tert*-Butyldimethylsilyl)oxy)-3-((2-methoxyethoxy)methyl)cyclohex-2-yl] 2,3,4-tri-*O*-benzyl-6-deoxy-α-L-galactopyranoside (16l). According to general procedure K, **15** (153 mg, 0.327 mmol), **(*R*)-14l** (52 mg, 0.163 mmol), TBAB (156 mg, 0.490 mmol) and 2,6-di-*tert*-butyl-4-methylpyridine (100 mg, 0.490 mmol) in DCM/DMF (2 mL) were reacted with $CuBr_2$ (109 mg, 0.490 mmol) to give **16l** (120 mg, quant). $[\alpha]_D^{20}$ -84.7 (*c* 0.82, DCM); ^1H NMR (500 MHz, $CDCl_3$): δ = 0.06, 0.08 (2 s, 6H, $Si(CH_3)_2$), 0.93 (s, 9H, $SiC(CH_3)_3$), 1.15 (d, J = 6.5 Hz, 3H, H-6^F), 1.30 (m, 1H, H-5a^{cyc}), 1.48 (dd, J = 9.8, 5.2 Hz, 1H, H-6a^{cyc}), 1.55 (m, 1H, H-4a^{cyc}), 1.67-1.86 (m, 3H, H-4b^{cyc}, H-5b^{cyc}, H-6b^{cyc}), 2.06 (dt, J = 9.2, 4.8 Hz, 1H, H-3^{cyc}), 3.41 (s, 3H, OCH_3), 3.48-3.61 (m, 6H, H-2^{cyc}, CH_2), 3.71 (m, 1H, H-4^F), 3.71-3.83 (m, 2H, H-1^{cyc}, CH_2), 4.00 (m, 1H, H-3^F), 4.05-4.13 (m, 2H, H-2^F, H-5^F), 4.65-4.84 (m, 4H, CH_2Ph), 4.89 (d, J = 11.8 Hz, 1H, B of AB, CH_2Ph), 5.00-5.06 (m, 2H, H-1^F, CH_2Ph), 7.06-7.63 (m, 15H, 3 C_6H_5); ^{13}C NMR (126 MHz, $CDCl_3$): δ = -5.13, -4.57 ($Si(CH_3)_2$), 16.73 (C-6^F), 17.35 (C-5^{cyc}), 18.07 (Si*C*$(CH_3)_3$), 23.93 (C-4^{cyc}), 25.89 (SiC(*C*$H_3)_3$), 31.06 (C-6^{cyc}), 38.97 (C-3^{cyc}), 59.03 (OCH_3), 66.44 (C-5^F), 70.01, 71.49, 71.68 , 71.89 (C-1^{cyc}, 3 CH_2), 72.99, 73.07, 74.76 (3 CH_2Ph), 76.50 (C-2^F), 76.93 (C-2^{cyc}), 77.83 (C-4^F), 79.21 (C-3^F), 96.40 (C-1^F), 127.33, 127.37, 127.40, 127.46, 127.89, 127.91, 128.03, 128.10, 128.19, 128.25, 128.34, 138.68, 138.73, 138.97 (18C, 3 C_6H_5); ESI-MS: *m/z*: Calcd for $C_{43}H_{62}NaO_8Si$ $[M+Na]^+$: 575.4; found: 575.4.

[(1*R*,2*R*,3*R*)-1-((*tert*-Butyldimethylsilyl)oxy)-3-isobutylcyclohex-2-yl] 2,3,4-tri-*O*-benzyl-6-deoxy-α-L-galactopyranoside (16m). According to general procedure K, **15** (131 mg, 0.279 mmol), **(*R*)-14m** (40 mg, 0.140 mmol), TBAB (135 mg, 0.419 mmol) and 2,6-di-*tert*-butyl-4-methylpyridine (86 mg, 0.419 mmol) in DCM/DMF (2 mL) were reacted with $CuBr_2$ (94 mg, 0.419 mmol) to give **16m** (76 mg, 77%). $[\alpha]_D^{20}$ -93.7 (*c* 1.12, DCM); ^1H NMR (500 MHz, $CDCl_3$): δ = 0.02 (2 s, 6H, $Si(CH_3)_2$), 0.83 (d, J = 6.4 Hz, 3H, CH_3), 0.84-0.90 (m, 12H, $SiC(CH_3)_3$, CH_3), 1.10 (d, J = 6.4 Hz, 3H, H-6^F), 1.15-1.29 (m, 2H, H-4a^{cyc}, H-5a^{cyc}), 1.35 (m, 1H, CH_2), 1.45 (m, 1H, H-6a^{cyc}), 1.52 (dt, J = 13.9, 6.6 Hz, 1H, CH_2), 1.61 (m, 1H, CH), 1.65-1.86 (m, 4H, H-3^{cyc}, H-4b^{cyc}, H-5b^{cyc}, H-6b^{cyc}), 3.39 (d, J = 3.6 Hz, 1H, H-2^{cyc}), 3.66 (d, J = 2.8 Hz, 1H, H-4^F), 3.79 (d, J = 4.6 Hz, 1H, H-1^{cyc}), 3.91-4.07 (m, 3H, H-2^F, H-3^F, H-5^F), 4.59-4.71 (m, 2H, CH_2Ph), 4.71-4.81 (m, 2H, CH_2Ph), 4.86 (d, J = 11.8 Hz, 1H, B of AB, CH_2Ph), 4.94-5.03 (m, 2H, H-1^F, CH_2Ph), 7.22-7.43 (m, 15H, 3 C_6H_5); ^{13}C

NMR (126 MHz, $CDCl_3$): *δ* = -5.10, -4.63 ($Si(CH_3)_2$), 16.52 (C-5^{cyc}), 16.75 (C-6^{F}), 18.10 (Si*C*$(CH_3)_3$), 22.16 (CH_3), 23.26 (CH_3), 25.62 (CH), 25.91 (C-4^{cyc}), 25.93 (SiC(*C*$H_3)_3$), 30.85 (C-6^{cyc}), 35.14 (C-3^{cyc}), 40.14 (CH_2), 66.49 (C-5^{F}), 71.24 (C-1^{cyc}), 73.05, 73.09, 74.77 (3 CH_2Ph), 76.73 (C-2^{F}), 77.86 (C-4^{F}), 79.20 (C-2^{cyc}), 79.34 (C-3^{F}), 96.67 (C-1^{F}), 127.38, 127.43, 127.44, 127.50, 127.78, 128.15, 128.24, 128.31, 128.38, 138.79, 138.92, 139.10 (18C, 3 C_6H_5); ESI-MS: *m/z*: Calcd for $C_{43}H_{62}NaO_6Si$ $[M+Na]^+$: 725.4; found: 725.8.

[(1*R*,2*R*,3*R*)-1-((*tert*-Butyldimethylsilyl)oxy)-3-(2,2,2-trifluoroethyl)cyclohex-2-yl] 2,3,4-tri-*O*-benzyl-6-deoxy-α-L-galactopyranoside (16n). According to general procedure K, **15** (181mg, 0.377 mmol), **(*R*)-14n** (59 mg, 0.188 mmol), TBAB (182 mg, 0.564 mmol) and 2,6-di-*tert*-butyl-4-methylpyridine (116 mg, 0.564 mmol) in DCM/DMF (3.5:0.5 mL) were reacted with $CuBr_2$ (126 mg, 0.564 mmol) to give crude **16n** (95 mg, 69%) which was used for the next step without further purification.

[(1*R*,2*R*,3*S*)-1-Hydroxy-3-methylcyclohex-2-yl] 2,3,4-tri-*O*-benzyl-6-deoxy-α-L-galactopyranoside (17b). Compound **16b** (2.10 g, 3.18 mmol) was dissolved in a solution of tetrabutylammonium fluoride in THF (1 M, 20 mL) and stirred for 24 h at rt. The solution was diluted with DCM (50 mL) and washed with H_2O (100 mL). The aquous layer was extracted with DCM (2 x 50 mL) and the combined organic layers were dried (Na_2SO_4) and concentrated. Column chromatography on silica (petroleum ether/EtOAc, 80:20) gave **17b** (1.74 g, 3.18 mmol, quant.) as white solid. $[\alpha]_D^{20}$ -42.0 (*c* 0.45, $CHCl_3$); NMR data were in accordance with literature.[7]

General procedure L:

[(1*R*,2*R*,3*S*)-3-Ethyl-1-hydroxycyclohex-2-yl] 2,3,4-tri-*O*-benzyl-6-deoxy-α-L-galactopyranoside (17c). To a solution of **16c** (1.42 g, 2.10 mmol) in MeOH (7.5 mL) was added 1 N H_2SO_4 in MeOH (420 µL, 0.42 mmol) at rt. The reaction mixture was stirred at rt overnight, then the solvent was removed under reduced pressure and the residue was purified by column chromatography on silica gel (toluene/EtOAc, 24:1 to 19:1) to give **17c** (1.05 g, 89%). $[\alpha]_D^{20}$ -63.7 (*c* 0.38, DCM); ^1H NMR (500 MHz, $CDCl_3$): *δ* = 0.79 (t, *J* = 7.5 Hz, 3H, CH_2CH_3), 0.90 (m, 1H, H-4b^{cyc}), 1.16 (m, 1H, H-6b^{cyc}), 1.16 (d, *J* = 6.5 Hz, 3H, H-6^{F}), 1.07-1.37 (m, 4H, H-3^{cyc}, H-5b^{cyc}, H-5ab^{cyc}, C*H*$_2CH_3$), 1.67 (m, 1H, H-5a^{cyc}), 1.79 (m, 1H, H-4a^{cyc}), 1.94-2.02 (m, 2H, C*H*$_2CH_3$, H-6a^{cyc}), 2.97 (dd, *J* = 10.2, 8.6 Hz, 1H, H-2^{cyc}), 3.38 (m, 1H, H-1^{cyc}), 3.70 (d, *J* = 1.6 Hz, 1H, H-4^{F}), 3.99 (dd, *J* = 10.2, 2.7 Hz, 1H, H-3^{F}), 4.10-4.14 (m, 2H, H-5^{F}, H-2^{F}), 4.66 (A of AB d, *J* = 11.5 Hz, 1H, CH_2Ph), 4.71 (A of AB d, *J* = 11.7 Hz, 1H, CH_2Ph), 4.76-4.79 (m, 2H, CH_2Ph, 1-OH), 4.80 (B of AB d, *J* = 11.7 Hz, 1H, CH_2Ph), 4.85 (B of AB d, *J* = 11.9 Hz, 1H, CH_2Ph), 4.99 (B of AB d, *J* = 11.1 Hz, 1H, CH_2Ph), 5.01 (d, *J* = 3.0 Hz, 1H, H-1^{F}), 7.24-7.49 (m, 15H, 3 C_6H_5); ^{13}C NMR (126 MHz, $CDCl_3$): *δ* = 10.98 (CH_2CH_3), 16.60 (H-6^{F}), 23.12 (C-5^{cyc}), 24.19, 29.55 (C-4^{cyc}), 32.64 (C-6^{cyc}), 42.69 (C-3^{cyc}), 67.53 (C-5^{F}), 72.87 (CH_2Ph), 73.18 (C-1^{cyc}), 73.52, 74.87 (2 CH_2Ph), 76.40 (C-2^{F}), 77.51(C-4$^{F)}$, 78.90 (C-3$^{F)}$, 91.09 (C-2^{cyc}), 98.36 (C-1^{F}), 127.43, 127.47, 127.51, 127.64, 127.83, 128.22, 128.34, 128.37, 138.36, 138.47, 138.75 (18C, 3 C_6H_5); ESI-MS: *m/z*: Calcd for $C_{35}H_{44}NaO_6$ $[M+Na]^+$: 583.3; found: 583.3.

[(1*R*,2*R*,3*S*)-1-Hydroxy-3-propylcyclohex-2-yl] 2,3,4-tri-*O*-benzyl-6-deoxy-α-L-galactopyranoside (17d). According to general procedure L, **16d** (160 mg, 0.232 mmol) in MeOH (2.8 mL) was treated with 1 M methanolic H_2SO_4 (23 µL) to give **17d** (109 mg, 81%). $[\alpha]_D^{20}$ -63.6 (*c* 0.90, DCM); ^1H NMR (500 MHz, $CDCl_3$): δ = 0.77 (t, *J* = 7.3 Hz, 3H, CH_3), 0.87 (m, 1H, H-4a^{cyc}), 1.01 (m, 1H, CH_2), 1.09-1.42 (m, 5H, H-3^{cyc}, H-5a^{cyc}, H-6a^{cyc}, CH_2), 1.15 (d, *J* = 6.5 Hz, 3H, H-6^F), 1.65 (dq, *J* = 13.1, 3.2 Hz, 1H, H-5b^{cyc}), 1.80 (dt, *J* = 13.5, 3.0 Hz, 1H, H-4b^{cyc}), 1.95-2.06 (m, 2H, H-6b^{cyc}, CH_2), 2.95 (dd, *J* = 10.3, 8.4 Hz, 1H, H-2^{cyc}), 3.36 (m, 1H, H-1^{cyc}), 3.68 (dd, *J* = 2.8, 1.3 Hz, 1H, H-4^F), 3.98 (dd, *J* = 10.2, 2.8 Hz, 1H, H-3^F), 4.07-4.16 (m, 2H, H-2^F, H-5^F), 4.65 (d, *J* = 11.5 Hz, 1H, A of AB, CH_2Ph), 4.71 (d, *J* = 11.8 Hz, 1H, A of AB, CH_2Ph), 4.74-4.86 (m, 4H, OH, CH_2Ph), 4.97-5.01 (m, 2H, H-1^F, CH_2Ph), 7.04-7.57 (m, 15H, 3 C_6H_5); ^{13}C NMR (126 MHz, $CDCl_3$): δ = 14.24 (CH_3), 16.60 (C-6^F), 19.94 (CH_2), 23.23 (C-5^{cyc}), 30.34 (C-4^{cyc}), 32.58 (C-6^{cyc}), 33.93 (CH_2), 41.02 (C-3^{cyc}), 67.56 (C-5^F), 72.90 (CH_2Ph), 73.20 (C-1^{cyc}), 73.48, 74.88 (2 CH_2Ph), 76.39 (C-2^F), 77.62 (C-4^F), 78.77 (C-3^F), 91.36 (C-2^{cyc}), 98.18 (C-1^F), 127.46, 127.50, 127.56, 127.64, 127.91, 128.23, 128.26, 128.37, 128.40, 138.36, 138.80 (18C, 3 C_6H_5); ESI-MS: *m/z*: Calcd for $C_{36}H_{46}NaO_6$ $[M+Na]^+$: 597.3; found: 597.4.

[(1*R*,2*R*,3*R*)-3-Benzyl-1-hydroxycyclohex-2-yl] 2,3,4-tri-*O*-benzyl-6-deoxy-α-L-galactopyranoside (17e). According to general procedure L, **16e** (106 mg, 0.144 mmol) in MeOH (1.5 mL) was treated with 1 M methanolic H_2SO_4 (29 µL) to give **17e** (70 mg, 80%). $[\alpha]_D^{20}$ -71.9 (c 0.96, DCM); ^1H NMR (500 MHz, $CDCl_3$): δ = 0.87 (m, 1H, H-4b^{cyc}), 1.11 (m, 1H, H-5b^{cyc}), 1.17 (d, *J* = 6.4 Hz, 1H, H-6^F), 1.26 (m, 1H, H-6b^{cyc}), 1.55-1.65 (m, 3H, H-3^{cyc}, H-4a^{cyc}, H-5a^{cyc}), 1.98 (m, 1H, H-6a^{cyc}), 2.06 (m, 1H, PhCH$_2$^{cyc}), 3.06 (t, *J* = 9.3 Hz, 1H, H-2^{cyc}), 3.39 (m, 1H, H-1^{cyc}), 3.64 (d, *J* = 13.1 Hz, 1H, PhCH$_2$^{cyc}), 3.73 (s, 1H, H-4^F), 4.06 (d, *J* = 10.2 Hz, 1H, H-3^F), 4.14-4.20 (m, 2H, H-5^F, H-2^F), 4.68 (d, *J* = 11.3 Hz, 1H, A of AB, CH_2Ph), 4.73 (d, *J* = 11.9 Hz, 1H, A of AB, CH_2Ph), 4.78 (d, *J* = 12.1 Hz, 1H, A of AB, CH_2Ph), 4.83 (m, 3H, 1-OH, CH_2Ph), 5.01 (d, *J* = 11.5 Hz, 1H, B of AB, CH_2Ph), 5.06 (d, *J* = 3.4 Hz, 1H, H-1^F), 7.05-7.46 (m, 20H, 4 C_6H_5); ^{13}C NMR (126 MHz, $CDCl_3$): δ = 16.64 (C-6^F), 22.87 (C-5^{cyc}), 29.98 (C-4^{cyc}), 32.48 (C-6^{cyc}), 38.44 (PhCH$_2$^{cyc}), 43.46 (C-3^{cyc}), 67.67 (C-5^F), 72.76 (CH_2Ph), 72.85 (C-1^{cyc}), 73.78, 74.90 (2 CH_2Ph), 76.23 (C-2^F), 77.53 (C-4^F), 78.84 (C-3^F), 90.89 (C-2^{cyc}), 98.00 (C-1^F), 125.50, 127.43, 127.51, 127.58, 127.65, 127.96, 127.99, 128.22, 128.25, 128.38, 129.27, 138.08, 138.42, 138.64, 141.45 (24C, 4 C_6H_5); ESI-MS: *m/z*: Calcd for $C_{40}H_{46}NaO_6$ $[M+Na]^+$: 645.3; found: 645.4.

[(1*R*,2*R*,3*R*)-3-(Cyclohexylmethyl)-1-hydroxycyclohex-2-yl] 2,3,4-tri-*O*-benzyl-6-deoxy-α-L-galactopyranoside (17f). According to general procedure L, **16f** (150 mg, 0.20 mmol) in MeOH (2.0 mL) was treated with 1 M methanolic H_2SO_4 (40 µL) to give **17f** (115 mg, 92%). $[\alpha]_D^{20}$ -61.5 (c 0.44, DCM); ^1H NMR (500 MHz, $CDCl_3$): δ = 0.63-0.90 (m, 4H), 1.04-1.31 (m, 9H), 1.43 (m, 2H), 1.55-1.72 (m, 5H), 1.81 (m, 1H), 1.98 (m, 1H), 2.06 (m, 1H), 2.88 (t, *J* = 9.2 Hz, 1H, H-2^{cyc}), 3.34 (m, 1H, H-1^{cyc}), 3.67 (s, 1H, H-4^F), 3.97 (d, *J* = 10.2 Hz, 1H, H-3^F), 4.12 (m, 2H, H-5^F, H-2^F), 4.63-4.73 (m, 2H, CH_2Ph), 4.75-4.79 (m, 2H, CH_2Ph), 4.86-4.89 (m, 2H, 1-OH, CH_2Ph), 5.01 (m, 2H, H-1^F, CH_2Ph), 7.22-7.43 (m, 15H, 3 C_6H_5); ^{13}C NMR (126 MHz, $CDCl_3$): δ = 16.58 (C-6^F), 23.22, 26.17, 26.40, 26.72, 30.80, 31.92, 32.51, 34.68, 34.96, 38.52, 39.42 (14C), 67.58 (C-5^F), 73.13, 73.36 (2 CH_2Ph), 73.55 (C-1^{cyc}), 74.89 (CH_2Ph), 76.67 (C-2^F), 77.79 (C-4^F), 78.64 (C-3^F),

92.09 (C-2^{cyc}), 98.58 (C-1^{F}), 127.47, 127.50, 127.52, 127.64, 127.91, 128.22, 128.24, 128.38, 128.44, 138.38, 138.47, 138.88 (18C, 3 C_6H_5); ESI-MS: *m*/*z*: Calcd for $C_{40}H_{52}NaO_6$ $[M+Na]^+$: 651.4; found: 651.5.

[(1*R*,2*R*,3*R*)-2-Hydroxy-3-phenylcyclohex-2-yl] 2,3,4-tri-*O*-benzyl-6-deoxy-α-L-galactopyranoside (17g). According to general procedure L, **16g** (85 mg, 0.118 mmol) in MeOH (1.5 mL) was treated with 1 M methanolic H_2SO_4 (17.6 µL) to give **17g** (46 mg, 64%). $[\alpha]_D^{20}$ -42.8 (*c* 0.73, DCM); ^{1}H NMR (500 MHz, $CDCl_3$): δ = 0.76 (d, *J* = 6.5 Hz, 3H, H-6^{F}), 1.32-1.50 (m, 3H, H-4a^{cyc}, H-5a^{cyc}, H-6a^{cyc}), 1.74 (ddd, *J* = 9.2, 5.9, 3.1 Hz, 1H, H-5b^{cyc}), 1.83 (m, 1H, H-4b^{cyc}), 2.11 (ddd, *J* = 11.7, 4.5, 2.3 Hz, 1H, H-6b^{cyc}), 2.71 (td, *J* = 11.2, 4.2 Hz, 1H, H-3^{cyc}), 3.51 (t, *J* = 2.0 Hz, 1H, H-4^{F}), 3.59-3.67 (m, 2H, H-5^{F}, H-1^{cyc}), 3.71 (dd, *J* = 10.8, 8.7 Hz, 1H, H-2^{cyc}), 3.79-3.87 (m, 2H, H-2^{F}, H-3^{F}), 4.42 (d, *J* = 12.2 Hz, 1H, A of AB, CH_2Ph), 4.47 (d, *J* = 12.2 Hz, 1H, B of AB, CH_2Ph), 4.53 (d, *J* = 11.5 Hz, 1H, A of AB, CH_2Ph), 4.61-4.65 (m, 2H, OH, CH_2Ph), 4.71 (d, *J* = 11.9 Hz, 1H, B of AB, CH_2Ph), 4.84-4.88 (m, 2H, H-1^{F}, CH_2Ph), 7.05-7.15, 7.17-7.37 (m, 20H, 4 C_6H_5); ^{13}C NMR (126 MHz, $CDCl_3$): δ = 16.08 (C-6^{F}), 23.68 (C-5^{cyc}), 32.56 (C-6^{cyc}), 35.53 (C-4^{cyc}), 47.76 (C-3^{cyc}), 67.47 (C-5^{F}), 71.77 (C-1^{cyc}), 72.79, 73.21, 74.56 (3 CH_2Ph), 75.30 (C-2^{F}), 77.43 (C-4^{F}), 79.05 (C-3^{F}), 86.65 (C-2^{cyc}), 94.98 (C-1^{F}), 126.07, 127.38, 127.47, 127.51, 127.65, 127.84, 128.15, 128.16, 128.21, 128.28, 128.36, 138.11, 138.59, 138.84, 143.53 (24C, 4 C_6H_5); ESI-MS: *m/z*: Calcd for $C_{39}H_{44}NaO_6$ $[M+Na]^+$: 631.3; found: 631.3.

[(1*R*,2*R*,3*R*)-3-*tert*-Butyl-1-hydroxycyclohex-2-yl] 2,3,4-tri-*O*-benzyl-6-deoxy-α-L-galactopyranoside (17h). According to general procedure L, **16h** (44 mg, 0.063 mmol) in MeOH (1.0 mL) was treated with 1 M methanolic H_2SO_4 (20 µL) to give slightly impure **17h** (46 mg, 64%) which was directly used in the next step.

[(1*R*,2*R*,3*S*)-3-Butyl-1-hydroxycyclohex-2-yl] 2,3,4-tri-*O*-benzyl-6-deoxy-α-L-galactopyranoside (17h). According to general procedure L, **16i** (93 mg, 0.132 mmol) in MeOH (1.5 mL) was treated with 1 M methanolic H_2SO_4 (13 µL) to give slightly impure **17i** (67 mg, 87%) which was directly used in the next step.

[(1*R*,2*R*,3*S*)-3-Hexyl-1-hydroxycyclohex-2-yl] 2,3,4-tri-*O*-benzyl-6-deoxy-α-L-galactopyranoside (17j). According to general procedure L, **16j** (240 mg, 0.328 mmol) in MeOH (4 mL) was treated with 1 M methanolic H_2SO_4 (64 µL) to give **17j** (192 mg, 95%). $[\alpha]_D^{20}$ -59.1 (*c* 1.3, DCM); ^{1}H NMR (500 MHz, $CDCl_3$): δ = 0.80-0.92 (m, 4H, CH_3, CH_2), 0.97 (tdd, *J* = 14.6, 9.6, 5.5 Hz, 1H, H-4a^{cyc}), 1.07-1.40 (m, 14H, H-6^{F}, H-3^{cyc}, H-5^{cyc}, H-6a^{cyc}, CH_2), 1.66 (m, 1H, CH_2), 1.81 (m, 1H, CH_2), 1.99 (m, 1H, H-6b^{cyc}), 2.09 (m, 1H, H-4b^{cyc}), 2.94 (dd, *J* = 10.3, 8.4 Hz, 1H, H-2^{cyc}), 3.36 (td, *J* = 10.1, 9.4, 4.8 Hz, 1H, H-1^{cyc}), 3.68 (dd, *J* = 2.9, 1.2 Hz, 1H, H-4^{F}), 3.98 (dd, *J* = 10.2, 2.7 Hz, 1H, H-3^{F}), 4.08-4.16 (m, 2H, H-2^{F}, H-5^{F}), 4.61-4.80 (m, 4H, CH_2Ph), 4.80-4.87 (m, 2H, CH_2Ph, OH), 4.97-5.02 (m, 2H, H-1^{F}, CH_2Ph), 7.17-7.50 (m, 15H, 3 C_6H_5); ^{13}C NMR (126 MHz, $CDCl_3$): δ = 14.16 (CH_3), 16.60 (C-6^{F}), 22.70 (C-5^{cyc}), 23.22, 26.94, 29.51, 30.30, 31.69 (5 CH_2), 31.99 (C-4^{cyc}), 32.59 (C-6^{cyc}), 41.31 (C-3^{cyc}), 67.57 (C-5^{F}), 73.05 (CH_2Ph), 73.26 (C-1^{cyc}), 73.42, 74.88 (2 CH_2Ph), 76.46 (C-2^{F}), 77.67 (C-4^{F}), 78.87 (C-3^{F}), 91.53 (C-2^{cyc}), 98.30 (C-1^{F}), 127.43, 127.49, 127.56, 127.64, 127.88,

128.23, 128.25, 128.36, 128.41, 138.36, 138.49, 138.84 (18C, 3 C_6H_5); ESI-MS: *m/z*: Calcd for $C_{39}H_{52}NaO_6$ $[M+Na]^+$: 639.4; found: 639.5.

[(1*R*,2*R*,3*S*)-1-Hydroxy-3-(3-phenylpropyl)cyclohex-2-yl] 2,3,4-tri-*O*-benzyl-6-deoxy-α-L-galactopyranoside (17k). According to general procedure L, **16k** (17 mg, 0.022 mmol) in MeOH (500 µL) was treated with 1 M methanolic H_2SO_4 (5.5 µL) to give **17k** (11.5 mg, 82%). $[\alpha]_D^{20}$ -56.5 (*c* 0.77, DCM); ^{1}H NMR (500 MHz, $CDCl_3$): δ = 0.88 (m, 1H, H-4a^{cyc}), 1.08 (m, 1H, CH_2), 1.15 (d, *J* = 6.6 Hz, 3H, H-6^F), 1.18-1.31 (m, 2H, H-5a^{cyc}, H-6a^{cyc}), 1.39 (m, 1H, H-3^{cyc}), 1.49 (m, 1H, CH_2), 1.57-1.73 (m, 2H, H-5b^{cyc}, CH_2), 1.80 (dd, *J* = 13.4, 3.7 Hz, 1H, H-4b^{cyc}), 1.99 (m, 1H, H-6b^{cyc}), 2.14 (m, 1H, CH_2), 2.31-2.48 (m, 2H, CH_2), 2.95 (t, *J* = 9.3 Hz, 1H, H-2^{cyc}), 3.36 (td, *J* = 9.8, 5.0 Hz, 1H, H-1^{cyc}), 3.68 (d, *J* = 2.7 Hz, 1H, H-4^F), 3.97 (dd, *J* = 10.3, 2.6 Hz, 1H, H-3^F), 4.11 (dt, *J* = 13.0, 4.9 Hz, 2H, H-2^F, H-5^F), 4.62-4.87 (m, 6H, CH_2Ph, OH), 4.97-5.03 (m, 2H, H-1^F, CH_2Ph), 7.04-7.18, 7.17-7.43 (m, 20H, 4 C_6H_5); ^{13}C NMR (126 MHz, $CDCl_3$): δ = 16.65 (C-6^F), 23.20 (C-5^{cyc}), 29.06 (CH_2), 30.38 (C-4^{cyc}), 31.85 (CH_2), 32.57 (C-6^{cyc}), 36.14 (CH_2), 41.31 (C-3^{cyc}), 67.61 (C-5^F), 72.96 (CH_2Ph), 73.24 (C-1^{cyc}), 73.54, 74.92 (2 CH_2Ph), 76.43 (C-2^F), 77.58 (C-4^F), 78.88 (C-3^F), 91.54 (C-2^{cyc}), 98.37 (C-1^F), 125.51, 127.44, 127.52, 127.67, 127.95, 128.18, 128.21, 128.26, 128.33, 128.39, 128.41, 128.47, 128.73, 138.38, 138.48, 138.79, 143.02 (24C, 4 C_6H_5); ESI-MS: *m/z*: Calcd for $C_{42}H_{50}NaO_6$ $[M+Na]^+$: 673.2; found: 673.3.

[(1*R*,2*R*,3*R*)-1-Hydroxy-3-((2-methoxyethoxy)methyl)cyclohex-2-yl] 2,3,4-tri-*O*-benzyl-6-deoxy-α-L-galactopyranoside (17l). According to general procedure L, **16l** (43 mg, 0.059 mmol) in MeOH (1.0 mL) was treated with 1 M methanolic H_2SO_4 (18 µL) to give **17l** (28 mg, 78%). $[\alpha]_D^{20}$ -58.3 (*c* 0.90, DCM); ^{1}H NMR (500 MHz, $CDCl_3$): δ = 1.09 (m, 1H, H-4a^{cyc}), 1.17 (d, *J* = 6.5 Hz, 3H, H-6^F), 1.20-1.31 (m, 2H, H-5a^{cyc}, H-6a^{cyc}), 1.62-1.74 (m, 2H, H-3^{cyc}, H-5b^{cyc}), 1.88-2.03 (m, 2H, H-4b^{cyc}, H-6b^{cyc}), 3.08 (dd, *J* = 10.4, 8.3 Hz, 1H, H-2^{cyc}), 3.24-3.35 (m, 4H, CH_2), 3.33 (s, 3H, OCH_3), 3.34-3.42 (m, 2H, H-1^{cyc}, CH_2), 3.70 (d, *J* = 2.6 Hz, 1H, H-4^F), 3.83 (dd, *J* = 9.2, 2.8 Hz, 1H, CH_2), 3.96 (dd, *J* = 10.2, 2.6 Hz, 1H, H-3^F), 4.07-4.14 (m, 2H, H-2^F, H-5^F), 4.61-4.89 (m, 8H, CH_2Ph, OH), 4.95-5.01 (m, 2H, H-1^F, CH_2Ph), 7.23-7.42 (15H, 3 C_6H_5); ^{13}C NMR (126 MHz, $CDCl_3$): δ = 16.61 (C-6^F), 22.77 (C-5^{cyc}), 28.58 (C-4^{cyc}), 32.46 (C-6^{cyc}), 42.25 (C-3^{cyc}), 59.01 (OCH_3), 67.64 (C-5^F), 70.18 , 71.81, 72.40 (3 CH_2), 72.97 (CH_2Ph), 73.55, 73.64 (C-1^{cyc}, CH_2Ph), 74.89 (CH_2Ph), 76.38 (C-2^F), 77.40 (C-4^F), 79.02 (C-3^F), 88.28 (C-2^{cyc}), 99.03 (C-1^F), 127.39, 127.52, 127.57, 127.65, 127.82, 128.22, 128.27, 128.37, 138.36, 138.43, 138.67 (18C, 3 C_6H_5); ESI-MS: *m/z*: Calcd for $C_{37}H_{48}NaO_8$ $[M+Na]^+$: 643.3; found: 643.3.

[(1*R*,2*R*,3*R*)-1-Hydroxy-3-isobutylcyclohex-2-yl] 2,3,4-tri-*O*-benzyl-6-deoxy-α-L-galactopyranoside (17m). According to general procedure L, **16m** (50 mg, 0.071 mmol) in MeOH (1.0 mL) was treated with 1 M methanolic H_2SO_4 (21 µL) to give **17m** (33 mg, 80%). $[\alpha]_D^{20}$ -55.7 (*c* 1.32, DCM); ^{1}H NMR (500 MHz, $CDCl_3$): δ = 0.73-0.79 (m, 6H, 2 CH_3), 0.79-0.90 (m, 2H, H-4a^{cyc}, CH_2), 1.15 (d, *J* = 6.5 Hz, 3H, H-6^F), 1.16-1.30 (m, 2H, H-5a^{cyc}, H-6a^{cyc}), 1.38 (q, *J* = 11.6, 11.1 Hz, 1H, H-3^{cyc}), 1.53-1.68 (m, 3H, H-5b^{cyc}, CH), 1.81 (m, 1H, H-4b^{cyc}), 1.92-2.02 (m, 2H, H-6b^{cyc}, CH_2), 2.89 (t, *J* = 9.3 Hz, 1H, H-2^{cyc}), 3.35 (td, *J* = 9.9, 5.0 Hz,

1H, H-1^{cyc}), 3.66 (d, J = 2.6 Hz, 1H, H-4^{F}), 3.95 (dd, J = 10.2, 2.6 Hz, 1H, H-3^{F}), 4.07-4.16 (m, 2H, H-2^{F}, H-5^{F}), 4.62-4.80 (m, 4H, CH_2Ph), 4.85 (d, J = 12.2 Hz, 1H, B of AB, CH_2Ph), 4.89 (s, 1H, OH), 4.97-5.04 (m, 2H, H-1^{F}, CH_2Ph), 7.17-7.44 (m, 15H, 3 C_6H_5); ^{13}C NMR (126 MHz, $CDCl_3$): δ = 16.61 (C-6^{F}), 21.29 (CH_3), 23.27 (C-5^{cyc}), 24.14 (CH_3), 25.46 (CH), 30.73 (C-4^{cyc}), 32.53 (C-6^{cyc}), 39.29 (C-3^{cyc}), 41.18 (CH_2), 67.61 (C-5^{F}), 73.10 (CH_2Ph), 73.37 (C-1^{cyc}), 73.53 , 74.92 (2 CH_2Ph), 76.70 (C-2^{F}), 77.81 (C-4^{F}), 78.58 (C-3^{F}), 92.04 (C-2^{cyc}), 98.51 (C-1^{F}), 127.53, 127.56, 127.67, 127.91, 128.11, 128.24, 128.28, 128.37, 128.40, 128.44, 128.47, 138.40, 138.50, 138.87 (18C, 3 C_6H_5); ESI-MS: *m/z*: Calcd for $C_{37}H_{48}NaO_6$ $[M+Na]^+$: 611.2; found: 611.3.

[(1*R*,2*R*,3*R*)-1-Hydroxy-3-(2,2,2-trifluoroethyl)cyclohex-2-yl] 2,3,4-tri-*O*-benzyl-6-deoxy-α-L-galactopyranoside (17n). According to general procedure L, **16n** (95 mg, 0.13 mmol) in MeOH (1.0 mL) was treated with 1 M methanolic H_2SO_4 (33 µL) to give **17n** (71 mg, 88%). ^{1}H NMR (500 MHz, $CDCl_3$): δ = 1.01 (m, 1H), 1.16 (d, J = 6.5 Hz, 3H, H-6^{F}), 1.19-1.28 (m, 2H), 1.63-1.78 (m, 3H), 1.97-2.01 (m, 2H), 2.84 (m, 1H, H-2^{cyc}), 3.18 (p, J = 12.8 Hz, 1H, CH_2CF_3), 3.36 (m, 1H, H-1^{cyc}), 3.66 (d, J = 1.5 Hz, 1H, H-4^{F}), 3.95 (dd, J = 10.2, 2.7 Hz, 1H, H-3^{F}), 4.04-4.17 (m, 2H, H-2^{F}, H-5^{F}), 4.65 (d, J = 11.7 Hz, 1H, A of AB, CH_2Ph), 4.66 (d, J = 11.4 Hz, 1H, A of AB, CH_2Ph), 4.75 (d, J = 12.6 Hz, 2H, CH_2Ph), 4.80-4.89 (m, 2H, CH_2Ph), 4.93 (d, J = 3.7 Hz, 1H, H-1^{F}), 5.01 (d, J = 11.4 Hz, 1H, B of AB, CH_2Ph), 7.17-7.51 (m, 15H, 3 C_6H_5); ^{13}C NMR (126 MHz, $CDCl_3$): δ = 16.56 (C-6^{F}), 22.85, 30.75, 32.07, 34.86 (q, J = 27.4 Hz, CH_2CF_3), 35.08, 35.29, 35.51, 36.58, 68.04 (C-5^{F}), 72.90 (CH_2Ph), 73.61 (C-1^{cyc}), 74.00, 74.95 (2 CH_2Ph), 76.52 (C-2^{F}), 77.53 (C-4^{F}), 78.45 (C-3^{F}), 90.02 (C-2^{cyc}), 99.63 (C-1^{F}), 126.56, 127.56, 127.60, 127.69, 127.72, 127.80, 128.27, 128.36, 128.42, 128.76, 138.16, 138.35, 138.54 (18C, 3 C_6H_5).

Experimental information for compounds **19b** and **19c** is given in reference.[8]

General procedure M:

{(1*R*,2*R*,3*S*)-2-[(2,3,4-Tri-*O*-benzyl-6-deoxy-α-L-galactopyranosyl)oxy]-3-propylcyclohex-1-yl} 2,4,6-tri-*O*-benzoyl-3-*O*-[(1*S*)-1-benzyloxycarbonyl-2-cyclohexylethyl]-β-D-galactopyranoside (19d). A mixture of **17d** (84 mg, 0.146 mmol), **18** (136 mg, 0.175 mmol) and powdered activated molecular sieves 4 Å (1 g) in dry DCM (3 mL) was stirred at rt under argon for 4 h. A 4 h pre-stirred solution of DMTST (113 mg, 0.438 mmol) and powdered activated molecular sieves 4 Å (100 mg) in dry DCM (1 mL) was added slowly. After 16 h the reaction mixture was quenched with NEt_3 (400 µL), filtered over celite and evaporated to dryness. Column chromatography of the residue on silica gel (petroleum ether/EtOAc, 6:1) gave **19d** (140 mg, 74%). $[\alpha]_D^{20}$ -70.0 (*c* 0.64, DCM); ^{1}H NMR (500 MHz, $CDCl_3$): δ = 0.43-0.58, 0.61-0.92, 0.94-1.52, 1.53-1.63, 1.75-1.89 (m, 24H, H-3^{cyc}, H-4^{cyc}, H-5^{cyc}, H-6^{cyc}, $C_7H_{13}^{L}$, 2 CH_2), 0.77 (t, J = 7.2 Hz, 3H, CH_3), 1.40 (d, J = 6.4 Hz, 3H, H-6^{F}), 3.38 (m, 1H, H-2^{cyc}), 3.48-3.61 (m, 2H, H-4^{F}, H-1^{cyc}), 3.84 (dd, J = 9.9, 3.4 Hz, 1H, H-3^{G}), 3.93 (t, J = 6.5 Hz, 1H, H-5^{G}), 3.98-4.08 (m, 2H, H-2^{F}, H-3^{F}), 4.14 (dd, J = 8.0, 4.6 Hz, 1H, H-2^{L}), 4.23-4.34 (m, 2H, H-6a^{G}, CH_2Ph), 4.41 (dd, J = 11.4, 5.7 Hz, 1H, H-6b^{G}), 4.51-4.58 (m, 2H, H-1^{G}, CH_2Ph), 4.63-4.72 (m, 2H, CH_2Ph), 4.72-4.83 (m, 3H, H-5^{F}, CH_2Ph), 4.99-5.16 (m, 3H, H-1^{F}, CH_2Ph), 5.61 (t, J = 9.0 Hz, 1H, H-2^{G}), 5.85 (d, J = 3.3 Hz, 1H, H-4^{G}), 7.14-7.50, 7.50-7.61, 8.01-8.17 (m, 35H, 7 C_6H_5); ^{13}C NMR (126

MHz, $CDCl_3$): δ = 14.05 (CH_3), 16.79 (H-6^F), 19.27, 21.76, 25.45, 25.69, 26.06, 28.24, 30.14, 32.61, 32.66, 33.20, 33.37, 40.45 (C-4^{cyc}, C-5^{cyc}, C-6^{cyc}, $C_7H_{13}^L$, 2 CH_2), 42.52 (C-3^{cyc}), 62.52 (C-6^G), 66.38 (C-5^F), 66.59 (CH_2Ph), 70.14 (C-4^G), 71.46 (C-5^G), 72.12, 72.23 (C-2^G, CH_2Ph), 73.86, 74.88 (2 CH_2Ph), 76.23 (C-2^F), 78.03 (C-3^G), 78.39 (C-2^L), 78.77 (C-2^{cyc}), 79.24 (C-4^F), 79.71 (C-3^F), 80.70 (C-1^{cyc}), 97.70 (C-1^F), 100.01 (C-1^G), 126.93, 127.01, 127.17, 127.28, 127.74, 128.00, 128.03, 128.05, 128.11, 128.36, 128.41, 128.44, 128.46, 128.51, 129.63, 129.71, 129.76, 129.89, 129.91, 129.94, 132.99, 133.13, 133.20, 135.42, 138.71, 138.99, 139.17 (42C, 7 C_6H_5), 164.54, 166.04, 166.17, 172.41 (4 C=O); ESI-MS: *m/z*: Calcd for $C_{79}H_{88}NaO_{16}$ [M+Na]$^+$: 1315.6; found: 1315.9.

{(1*R*,2*R*,3*R*)-3-Benzyl-2-[(2,3,4-tri-*O*-benzyl-6-deoxy-α-L-galactopyranosyl)oxy]cyclohex-1-yl} 2,4,6-tri-*O*-benzoyl-3-*O*-[(1*S*)-1-benzyloxycarbonyl-2-cyclohexylethyl]-β-D-galactopyranoside (19e). According to general procedure M, **17e** (30 mg, 0.048 mmol) and **18** (75 mg, 0.096 mmol) in DCM (2.0 mL) were reacted with DMTST (25 mg, 0.096 mmol) in DCM (2.0 mL) for 16 h to give **19e** (59 mg, 92%). $[\alpha]_D^{20}$ -69.8 (c 1.15, DCM); ^{1}H NMR (500 MHz, $CDCl_3$): δ = 0.50 (m, 1H), 0.60-0.94 (m, 3H), 1.02-1.50 (m, 8H), 1.82 (m, 1H), 2.14 (t, *J* = 12.1 Hz, 1H, PhCH_2^{cyc}), 3.41-3.51 (m, 2H, H-2^{cyc}, PhCH_2^{cyc}), 3.54 (s, 1H, H-4^F), 3.60 (td, *J* = 9.2, 4.2 Hz, 1H, H-1^{cyc}), 3.86 (dd, *J* = 9.9, 3.3 Hz, 1H, H-3^G), 3.95 (t, *J* = 6.4 Hz, 1H, H-5^G), 4.04 (dd, *J* = 10.3, 3.4 Hz, 1H, H-2^F), 4.08 (dd, *J* = 10.3, 2.2 Hz, 1H, H-3^F), 4.16 (dd, *J* = 7.0, 3.6 Hz, 1H, H-2^L), 4.29 (dd, *J* = 11.4, 7.4 Hz, 1H, H-6b^G), 4.35 (d, *J* = 11.4 Hz, 1H, A of AB, CH_2Ph), 4.44 (dd, *J* = 11.4, 5.4 Hz, 1H, H-6a^G), 4.58 (m, 3H, H-1^G, CH_2Ph), 4.71 (d, *J* = 12.0 Hz, 1H, A of AB, CH_2Ph), 4.73 (m, 1H, H-5^F), 4.77 (d, *J* = 11.2 Hz, 1H, B of AB, CH_2Ph), 4.81 (d, *J* = 11.4 Hz, 1H, B of AB, CH_2Ph), 5.06 (d, *J* = 11.9 Hz, 1H, B of AB, CH_2Ph), 5.08 (s, 1H, H-1^F), 5.14 (d, *J* = 12.1 Hz, 1H, B of AB, CH_2Ph), 5.66 (t, *J* = 9.0 Hz, 1H, H-2^G), 5.87 (d, *J* = 3.4 Hz, 1H, H-4^G), 7.00 (d, *J* = 7.3 Hz, 2H), 7.08-7.10, 7.10-7.37, 7.40-7.49, 7.52-7.65, 8.05-8.18 (40 H, 8 C_6H_5); ^{13}C NMR (126 MHz, $CDCl_3$): δ = 16.79 (C-6^F), 21.01, 25.46, 25.72, 26.07, 27.70, 29.84, 32.61, 33.23, 33.41, 37.80, 40.47, 44.47 (11 C, PhCH_2^{cyc}, Cy-Lac), 62.61(C-6^G), 66.55 (C-5^F), 66.63 (CH_2Ph), 70.17 (C-4^G), 71.52 (C-5^G), 72.21 (2C, C-2^G, CH_2Ph), 74.32, 74.93 (2 CH_2Ph), 76.54 (C-2^F), 77.96 (C-3^G), 78.41 (C-2^L), 79.13 (C-4^F), 79.73 (C-3^F), 80.04 (C-2^{cyc}), 80.24 (C-1^{cyc}), 98.20 (C-1^F), 100.05 (C-1^G), 125.49, 127.02, 127.11, 127.26, 127.31, 127.82, 127.97, 128.07, 128.12, 128.40, 128.45, 128.50, 128.52, 128.55, 129.26, 129.66, 129.74, 129.89, 129.97, 133.03, 133.20, 133.25, 135.44, 138.43, 139.00, 139.14, 140.95 (48C, 8 C_6H_5), 164.63, 166.10, 166.21, 172.46 (4 C=O); ESI-MS: *m*/*z*: Calcd for $C_{83}H_{86}NaO_{16}$ [M+Na]$^+$: 1363.6; found 1364.2.

{(1*R*,2*R*,3*R*)-2-[(2,3,4-Tri-*O*-benzyl-6-deoxy-α-L-galactopyranosyl)oxy]-3-(cyclohexylmethyl)cyclohex-1-yl} 2,4,6-tri-*O*-benzoyl-3-*O*-[(1*S*)-1-benzyloxycarbonyl-2-cyclohexylethyl]-β-D-galactopyranoside (19f). According to general procedure M, **17f** (34 mg, 0.054 mmol) and **18** (84 mg, 0.108 mmol) in DCM (2.0 mL) were reacted with DMTST (28 mg, 0.108 mmol) in DCM (2.0 mL) for 16 h to give **19f** (73 mg, impure). ^{1}H NMR (500 MHz, $CDCl_3$): δ = 0.34-1.89 (m, 24H), 3.34 (t, *J* = 7.7 Hz, 1H, H-2^{cyc}), 3.52 (s, 1H, H-4^F), 3.57 (m, 1H, H-1^{cyc}), 3.84 (dd, *J* = 9.8, 3.0 Hz, 1H, H-3^G), 3.99-4.05 (m, 3H, H-3^F, H-2^F, H-5^G), 4.14 (dd, *J* = 7.7, 4.6 Hz, 1H, H-2^L), 4.31 (d, *J* = 11.6 Hz, 1H, A of AB, CH_2Ph), 4.36-4.52 (m, 2H, H-6^G), 4.55 (d, *J* = 11.7 Hz,

1H, A of AB, CH_2Ph), 4.64-4.71 (m, 3H, CH_2Ph, H-5^F), 4.76 (d, *J* = 11.7 Hz, B of AB, CH_2Ph), 4.77 (d, *J* = 11.4 Hz, 1H, B of AB, CH_2Ph), 5.08 (s, 1H, H-1^F), 5.61 (t, *J* = 8.9 Hz, 1H, H-2^G), 5.85 (d, *J* = 2.9 Hz, 1H, H-4^G), 7.03-8.28 (m, 35H, 7 C_6H_5); ESI-MS: *m/z*: Calcd for $C_{83}H_{94}NaO_{16}$ $[M+Na]^+$: 1369.6; found: 1369.6.

{(1*R*,2*R*,3*R*)-2-[(2,3,4-Tri-*O*-benzyl-6-deoxy-α-L-galactopyranosyl)oxy]-3-phenylcyclohex-1-yl} 2,4,6-tri-*O*-benzoyl-3-*O*-[(1*S*)-1-benzyloxycarbonyl-2-cyclohexylethyl]-β-D-galactopyranoside (19g). According to general procedure M, **17g** (23 mg, 0.038 mmol) and **18** (35 mg, 0.045 mmol) in DCM (1.0 mL) were reacted with DMTST (20 mg, 0.076 mmol) in DCM (500 µL) for 16 h to give **19g** (34 mg, 68%). $[\alpha]_D^{20}$ -51.6 (*c* 0.88, DCM); ^1H NMR (500 MHz, $CDCl_3$): δ = 0.42-0.58, 0.61-0.98, 1.02-1.44 (m, 16H, H-4a^{cyc}, H-5a^{cyc}, H-6a^{cyc}, $C_7H_{13}{}^L$), 1.42 (d, *J* = 6.5 Hz, 3H, H-6^F), 1.51-1.63 (m, 2H, H-4b^{cyc}, H-5b^{cyc}), 2.02 (ddd, *J* = 13.9, 6.3, 3.2 Hz, 1H, H-6b^{cyc}), 2.63 (ddd, *J* = 13.7, 10.3, 3.7 Hz, 1H, H-3^{cyc}), 3.49 (d, *J* = 3.3 Hz, 1H, H-4^F), 3.58 (dd, *J* = 10.2, 3.7 Hz, 1H, H-2^F), 3.71 (ddd, *J* = 11.2, 8.6, 4.7 Hz, 1H, H-1^{cyc}), 3.86 (dd, *J* = 9.9, 3.5 Hz, 1H, H-3^G), 3.91-4.03 (m, 3H, H-3^F, H-5^G, H-2^{cyc}), 4.05-4.19 (m, 3H, H-2^L, CH_2Ph), 4.22-4.37 (m, 4H, H-1^F, H-6^G, CH_2Ph), 4.45 (d, *J* = 11.8 Hz, 1H, A of AB, CH_2Ph), 4.57-4.70 (m, 3H, H-1^G, CH_2Ph), 4.92 (q, *J* = 6.4 Hz, 1H, H-5^F), 5.05 (d, *J* = 12.1 Hz, 1H, A of AB, CH_2Ph), 5.10 (d, *J* = 12.1 Hz, 1H, B of AB, CH_2Ph), 5.61 (dd, *J* = 9.9, 8.1 Hz, 1H. H-2^G), 5.87 (d, *J* = 3.4 Hz, 1H, H-4^G), 7.05-7.35, 7.36-7.63, 8.05-8.18 (m, 40H, 8 C_6H_5); ^{13}C NMR (126 MHz, $CDCl_3$): δ = 16.82 (C-6^F), 23.21 (C-5^{cyc}), 25.48, 25.72, 26.08, 32.66, 33.23, 33.39, 40.47 ($C_7H_{13}{}^L$), 30.75 (C-6^{cyc}), 35.01 (C-4^{cyc}), 50.91 (C-3^{cyc}), 62.17 (C-6^G), 65.99 (C-5^F), 66.62 (CH_2Ph), 69.97 (C-4^G), 71.39 (C-5^G), 72.25, 72.27 (3C, C-2^G, CH_2Ph), 74.94 (CH_2Ph), 75.74 (C-2^F), 76.94 (C-2^{cyc}), 78.08 (C-3^G), 78.41 (C-2^L), 79.09 (C-3^F), 79.72 (C-4^F), 81.06 (C-1^{cyc}), 94.79 (C-1^F), 99.62 (C-1^G), 126.45, 126.69, 126.80, 126.92, 127.06, 127.65, 127.76, 127.87, 127.94, 128.23, 128.37, 128.45, 128.46, 128.49, 128.51, 128.53, 128.59, 129.69, 129.79, 129.85, 130.02, 133.09, 133.24, 133.27, 135.44, 138.83, 139.16, 139.19, 143.55 (48C, 8 C_6H_5), 164.59, 165.95, 166.16, 172.38 (4 C=O); ESI-MS: *m/z*: Calcd for $C_{82}H_{86}NaO_{16}$ $[M+Na]^+$: 1349.6; found: 1349.8.

{(1*R*,2*R*,3*R*)-2-[(2,3,4-Tri-*O*-benzyl-6-deoxy-α-L-galactopyranosyl)oxy]-3-(*tert*-butyl)cyclohex-1-yl} 2,4,6-tri-*O*-benzoyl-3-*O*-[(1*S*)-1-benzyloxycarbonyl-2-cyclohexylethyl]-β-D-galactopyranoside (19h). According to general procedure M, **17h** (16 mg, 0.027 mmol) and **18** (25 mg, 0.036 mmol) in DCM (700 µL) were reacted with DMTST (21 mg, 0.081 mmol) in DCM (300 µL) for 16 h to give **19h** (34 mg, 68%). $[\alpha]_D^{20}$ -56.1 (*c* 0.85, DCM); ^1H NMR (500 MHz, $CDCl_3$): δ = 0.41-0.59, 0.60-0.86, 1.12-1.46, 1.51-1.64 (m, 20H, $C_7H_{13}{}^L$, C-3^{cyc}, C-4^{cyc}, C-5^{cyc}, C-6^{cyc}), 0.87 (s, 9H, $C(CH_3)_3$), 1.09 (d, *J* = 6.5 Hz, 3H, H-6^F), 3.59 (m, 1H, H-4^F), 3.79-3.92 (m, 6H, H-3^G, H-5^G, H-2^F, H-3^F, H-5^F, H-2^{cyc}), 4.09 (d, *J* = 3.2 Hz, 1H, H-1^{cyc}), 4.17 (dd, *J* = 8.6, 4.2 Hz, 1H, H-2^L), 4.34 (dd, *J* = 11.6, 7.2 Hz, 1H, H-6a^G), 4.41 (dd, *J* = 11.7, 4.7 Hz, 1H, H-6b^G), 4.50 (s, 2H, CH_2Ph), 4.57 (d, *J* = 11.2 Hz, 1H, A of AB, CH_2Ph), 4.62 (d, *J* = 7.9 Hz, 1H, H-1^G), 4.67 (d, *J* = 11.9 Hz, 1H, A of AB, CH_2Ph), 4.79 (d, *J* = 11.9 Hz, 1H, B of AB, CH_2Ph), 4.90-4.99 (m, 2H, H-1^F, CH_2Ph), 5.04 (d, *J* = 12.1 Hz, 1H, A of AB, CH_2Ph), 5.19 (d, *J* = 12.1 Hz, 1H, B of AB, CH_2Ph), 5.59 (m, 1H, H-2^G), 5.88 (d, *J* = 3.2 Hz, 1H, H-4^G), 7.12-7.39, 7.38-7.51, 7.51-7.62, 7.92-8.20 (m, 35H, 7 C_6H_5); ^{13}C NMR (126 MHz, $CDCl_3$): δ = 16.73 (C-6^F), 18.06, 19.28, 22.54, 25.50, 25.80, 26.14, 32.51, 32.70, 33.52, 40.48 ($C_7H_{13}{}^L$, C-4^{cyc},

C-5^{cyc}, H-6^{cyc}), 28.44 (C(CH_3)$_3$), 33.26 (*C*(CH_3)$_3$), 50.09 (C-3^{cyc}), 63.37 (C-6^G), 66.64 (C-5^F), 66.73 (CH_2Ph), 70.12 (C-4^G), 71.83 (C-5^G), 72.80, 72.82 (C-2^G, CH_2Ph), 73.01, 74.94 (2 CH_2Ph), 76.57 (C-2^F), 78.15 (2C, C-3^G, C-1^{cyc}), 78.21 (C-3^F), 78.99 (C-2^L), 79.12 (2C, C-4^F, C-2^{cyc}), 97.78 (C-1^F), 100.69 (C-1^G), 127.21, 127.28, 127.30, 127.50, 127.72, 128.01, 128.16, 128.24, 128.36, 128.40, 128.42, 128.55, 128.59, 129.52, 129.54, 129.70, 129.73, 130.01, 130.12, 133.05, 133.08, 133.13, 135.54, 138.62, 138.90, 139.08 (42C, 7 C_6H_5), 164.78, 165.62, 166.19, 172.57 (4 C=O); ESI-MS: *m/z*: Calcd for $C_{80}H_{90}NaO_{16}$ [M+Na]$^+$: 1329.6; found: 1329.7.

{(1*R*,2*R*,3*S*)-2-[(2,3,4-Tri-*O*-benzyl-6-deoxy-α-L-galactopyranosyl)oxy]-3-butylcyclohex-1-yl} 2,4,6-tri-*O*-benzoyl-3-*O*-[(1*S*)-1-benzyloxycarbonyl-2-cyclohexylethyl]-β-D-galactopyranoside (19i). According to general procedure M, **17i** (67 mg, 0.114 mmol) and **18** (106 mg, 0.136 mmol) in DCM (3 mL) were reacted with DMTST (59 mg, 0.227 mmol) in DCM (500 µL) for 16 h to give **19i** (79 mg, 53%). $[\alpha]_D^{20}$ -55.5 (*c* 1.36, DCM); ^{1}H NMR (500 MHz, $CDCl_3$): δ = 0.42-0.57, 0.64-0.90, 0.94-1.62, 1.75-1.91 (m, 26H, $C_7H_{13}^L$, H-3^{cyc}, H-4^{cyc}, H-5^{cyc}, H-6^{cyc}, 3 CH_2), 0.80 (t, *J* = 6.9 Hz, 3H, CH_3), 1.40 (d, *J* = 6.5 Hz, 3H, H-6^F), 3.37 (t, *J* = 8.4 Hz, 1H, H-2^{cyc}), 3.51-3.58 (m, 2H, H-4^F, H-1^{cyc}), 3.84 (dd, *J* = 9.9, 3.5 Hz, 1H, H-3^G), 3.94 (m, 1H, H-5^G), 4.02 (dd, *J* = 10.3, 3.4 Hz, 1H, H-2^F), 4.05 (dd, *J* = 10.3, 2.5 Hz, 1H, H-3^F), 4.14 (m, 1H, H-2^L), 4.25-4.32 (m, 2H, H-6a^G, CH_2Ph), 4.41 (dd, *J* = 11.4, 5.7 Hz, 1H, G-6b^G), 4.53 (d, *J* = 11.8 Hz, 1H, A of AB, CH_2Ph), 4.56 (d, *J* = 8.2 Hz, 1H, H-1^G), 4.63-4.71 (m, 2H, CH_2Ph), 4.72-4.82 (m, 3H, H-5^F, CH_2Ph), 5.02 (d, *J* = 3.4 Hz, 1H, H-1^F), 5.05 (d, *J* = 12.1 Hz, 1H, A of AB, CH_2Ph), 5.12 (d, *J* = 12.1 Hz, 1H, B of AB, CH_2Ph), 5.61 (dd, *J* = 9.9, 8.1 Hz, 1H, H-2^G), 5.85 (dd, *J* = 3.5, 1.0 Hz, 1H, H-4^G), 7.13-7.37, 7.39-7.50, 7.50-7.62, 8.01-8.17 (m, 35H, 7 C_6H_5); ^{13}C NMR (126 MHz, $CDCl_3$): δ = 14.21 (CH_3), 16.78 (C-6^F), 21.73 (C-5^{cyc}), 22.91, 25.44, 25.69, 26.06, 28.29, 30.12, 32.61, 33.19 , 33.37, 40.45 (C_7H_{13}L, 2 CH_2), 28.56 (C-4^{cyc}), 30.37 (C-6^{cyc}), 42.72 (C-3^{cyc}), 62.52 (C-6^G), 66.37 (C-5^F), 66.59 (CH_2Ph), 70.15 (C-4^G), 71.46 (C-5^G), 72.12 (C-2^G), 72.12, 73.83, 74.89 (3 CH_2Ph), 76.22 (C-2^F), 78.03 (C-3^G), 78.39 (C-2^L), 78.74 (C-2^{cyc}), 79.24 C-4^F), 79.71 (C-3^F), 80.72 (C-1^{cyc}), 97.69 (C-1^F), 100.01 (C-1^G), 126.93, 127.01, 127.17, 127.25, 127.75, 128.00, 128.05, 128.09, 128.33, 128.35, 128.40, 128.44, 128.45, 128.51, 129.62, 129.72, 129.77, 129.89, 129.91, 129.94, 132.98, 133.12, 133.20, 135.42, 138.71, 138.99, 139.16 (42C, 7 C_6H_5), 164.53, 166.05, 166.16, 172.40 (4 C=O); ESI-MS; *m/z*: Calcd for $C_{80}H_{90}NaO_{16}$ [M+Na]$^+$: 1329.6; found: 1329.9.

{(1*R*,2*R*,3*S*)-2-[(2,3,4-Tri-*O*-benzyl-6-deoxy-α-L-galactopyranosyl)oxy]-3-hexylcyclohex-1-yl} 2,4,6-tri-*O*-benzoyl-3-*O*-[(1*S*)-1-benzyloxycarbonyl-2-cyclohexylethyl]-β-D-galactopyranoside (19j). According to general procedure M, **17j** (100 mg, 0.162 mmol) and **18** (151 mg, 0.195 mmol) in DCM (3.0 mL) were reacted with DMTST (125 mg, 0.486 mmol) in DCM (1.0 mL) for 18 h to give **19j** (180 mg, 84%). $[\alpha]_D^{20}$ -59.1 (*c* 0.68, DCM); ^{1}H NMR (500 MHz, $CDCl_3$): δ = 0.43-0.54, 0.63-1.63, 1.73-1.88 (m, 30H, H-3^{cyc}, H-4^{cyc}, H-5^{cyc}, H-6^{cyc}, $C_7H_{13}^L$, 6 CH_2), 0.83 (t, *J* = 7.0 Hz, 3H, CH_3), 1.39 (d, *J* = 6.6 Hz, 3H, H-6^F), 3.35 (t, *J* = 8.5 Hz, 1H, H-2^{cyc}), 3.48-3.60 (m, 2H, H-4^F, H-1^{cyc}), 3.83 (dd, *J* = 9.9, 3.5 Hz, 1H, H-3^G), 3.92 (t, *J* = 6.5 Hz, 1H, H-5^G), 4.01 (dd, *J* = 10.3, 3.4 Hz, 1H, H-2^F), 4.04 (dd, *J* = 10.2, 2.5 Hz, 1H, H-3^F), 4.13 (m, 1H, H-6a^G), 4.23-4.31 (m, 2H, H-6b^G, CH_2Ph), 4.40 (dd, *J* = 11.5, 5.7 Hz, 1H, H-2^L), 4.52 (d, *J* = 11.7 Hz, 1H, A of AB, CH_2Ph), 4.55 (d, *J* = 8.2 Hz, 1H, H-1^G), 4.65 (d, *J* = 11.7 Hz, 1H, B of AB, CH_2Ph), 4.68 (d, *J* = 11.7 Hz, 1H, A of AB,

CH_2Ph), 4.72-4.82 (m, 3H, H-5^F, CH_2Ph), 5.01 (d, *J* = 3.4 Hz, 1H, H-1^F), 5.05 (d, *J* = 12.1 Hz, 1H, A of AB, CH_2Ph), 5.11 (d, *J* = 12.0 Hz, 1H, B of AB, CH_2Ph), 5.60 (m, 1H, H-2^G), 5.84 (d, *J* = 3.5 Hz, 1H, H-4^G), 7.13-7.38, 7.40-7.48, 7.50-7.61, 8.02-8.15 (m, 35H, 7 C_6H_5); ^{13}C NMR (126 MHz, $CDCl_3$): δ = 14.13 (CH_3), 16.84 (C-6^F), 22.68 (C-5^{cyc}), 21.94, 25.50, 25.75, 26.11, 26.40, 28.35, 29.62, 30.25, 30.69, 33.25, 33.42, 40.50 ($C_7H_{13}^L$, 5 CH_2), 32.14 (C-4^{cyc}), 32.67 (C-6^{cyc}), 42.91 (C-3^{cyc}), 62.55 (C-6^G), 66.41 (C-5^F), 66.65 (CH_2Ph), 70.20 (C-4^G), 71.51 (C-5^G), 72.17, 73.89 (3C, C-2^G, 2 CH_2Ph), 74.95 (CH_2Ph), 76.26 (C-2^F), 78.10 (C-3^G), 78.45 (C-2^L), 78.91 (C-2^{cyc}), 79.33 (C-4^F), 79.77 (C-3^F), 80.84 (C-1^{cyc}), 97.76 (C-1^F), 100.04 (C-1^G), 126.98, 127.05, 127.22, 127.31, 127.81, 128.06, 128.10, 128.13, 128.41, 128.46, 128.51, 128.56, 129.69, 129.78, 129.82, 129.96, 129.99, 133.04, 133.17, 133.26, 135.47, 138.77, 139.05, 139.23 (42C, 7 C_6H_5), 164.58, 166.12, 166.23, 172.47 (4 C=O); ESI-MS: *m/z*: Calcd for $C_{82}H_{94}NaO_{16}$ [M+Na]$^+$: 1357.6; found: 1357.9.

{(1*R*,2*R*,3*S*)-2-[(2,3,4-Tri-*O*-benzyl-6-deoxy-α-L-galactopyranosyl)oxy]-3-(3-phenylpropyl)cyclohex-1-yl} 2,4,6-tri-*O*-benzoyl-3-*O*-[(1*S*)-1-benzyloxycarbonyl-2-cyclohexylethyl]-β-D-galactopyranoside (19k). According to general procedure M, **17k** (11.5 mg, 0.018 mmol) and **18** (20 mg, 0.026 mmol) in DCM (600 µL) were reacted with DMTST (14 mg, 0.053 mmol) in DCM (500 µL) for 17 h to give **19k** (10 mg, 42%). $[\alpha]_D^{20}$ -61.1 (*c* 0.63, DCM); ^{1}H NMR (500 MHz, $CDCl_3$): δ = 0.44-0.56, 0.62-1.65, 1.72-1.96, 2.28-2.53 (m, 26H, $C_7H_{13}^L$, H-3^{cyc}, H-4^{cyc}, H-5^{cyc}, H-6^{cyc}, 3 CH_2), 1.38 (d, *J* = 6.5 Hz, 3H, H-6^F), 3.36 (m, 1H, H-2^{cyc}), 3.51 (m, 1H, H-4^F), 3.57 (td, *J* = 8.9, 4.0 Hz, 1H, H-1^{cyc}), 3.84 (dd, *J* = 10.1, 3.3 Hz, 1H, H-3^G), 3.04 (m, 1H, H-5^G), 3.99-4.06 (m, 2H, H-2^F, H-3^F), 4.13 (dd, *J* = 8.1, 5.0 Hz, 1H, H-2^L), 4.25-4.33 (m, 2H, H-6a^G, CH_2Ph), 4.40 (dd, *J* = 11.5, 5.6 Hz, 1H, H-6b^G), 4.53 (d, *J* = 11.9 Hz, 1H, A of AB, CH_2Ph), 4.56 (d, *J* = 8.4 Hz, 1H, H-1^G), 4.63-4.79 (m, 5H, H-5^F, CH_2Ph), 5.01-5.08 (m, 2H, H-1^F, CH_2Ph), 5.12 (d, *J* = 12.1 Hz, 1H, B of AB, CH_2Ph), 5.62 (m, 1H, H-2^G), 5.85 (d, *J* = 3.3 Hz, 1H, H-4^G), 6.99-7.07, 7.08-7.38, 7.39-7.49, 7.49-7.63, 8.00-8.17 (m, 40H, 8 C_6H_5); ^{13}C NMR (126 MHz, $CDCl_3$): δ = 16.84 (C-6^F), 21.52, 25.51, 25.76, 26.12, 28.17, 28.76, 32.68, 33.27, 33.44, 36.10, 40.51 ($C_7H_{13}^L$, C-5^{cyc}, 3 CH_2), 30.00 (C-4^{cyc}), 30.56 (C-6^{cyc}), 42.70 (C-3^{cyc}), 62.61 (C-6^G), 66.49 (C-5^F), 66.66 (CH_2Ph), 70.21 (C-4^G), 71.58 (C-5^G), 72.24 (2C, C-2^G, CH_2Ph), 73.86 , 74.96 (2 CH_2Ph), 76.45 (C-2^F), 78.06 (C-3^G), 78.46 (C-2^L), 79.07 (C-2^{cyc}), 79.22 (C-4^F), 79.74 (C-3^F), 80.46 (C-1^{cyc}), 97.76 (C-1^F), 99.97 (C-1^G), 125.55, 127.02, 127.10, 127.27, 127.37, 127.86, 128.05, 128.09, 128.13, 128.16, 128.18, 128.29, 128.42, 128.47, 128.51, 128.52, 128.58, 129.69, 129.79, 129.82, 129.95, 129.98, 133.07, 133.19, 133.26, 135.49, 138.84, 139.04, 139.20, 142.73 (48C, 8 C_6H_5), 164.59, 166.13, 166.22, 172.48 (4 C=O); ESI-MS: *m/z*: Calcd for $C_{85}H_{92}NaO_{16}$ [M+Na]$^+$: 1392.6; found: 1392.0.

{(1*R*,2*R*,3*R*)-2-[(2,3,4-Tri-*O*-benzyl-6-deoxy-α-L-galactopyranosyl)oxy]-3-((2-methoxyethoxy)methyl)-cyclohex-1-yl} 2,4,6-tri-*O*-benzoyl-3-*O*-[(1*S*)-1-benzyloxycarbonyl-2-cyclohexylethyl]-β-D-galacto-pyranoside (19k). According to general procedure M, **17l** (27 mg, 0.044 mmol) and **18** (51 mg, 0.065 mmol) in DCM (1.4 mL) were reacted with DMTST (34 mg, 0.131 mmol) in DCM (600 µL) for 16 h to give **19l** (46 mg, 79%). $[\alpha]_D^{20}$ -56.6 (*c* 0.92, DCM); ^{1}H NMR (500 MHz, $CDCl_3$): δ = 0.37-0.58, 0.61-1.62, 1.76-1.89 (m, 20H, $C_7H_{13}^L$, H-3^{cyc}, H-4^{cyc}, H-5^{cyc}, H-6^{cyc}) 1.40 (d, *J* = 6.6 Hz, 3H, H-6^F), 3.24-3.45 (m, 5H, CH_2), 3.29 (s, 3H, OCH_3) 3.47-3.58 (m, 3H, H-1^{cyc}, H-2^{cyc}, H-4^F), 3.68 (dd, *J* = 9.3, 5.4 Hz, 1H, CH_2), 3.83 (dd, *J* = 10.0, 3.3

Hz, 1H, H-3^G), 3.92 (t, *J* = 6.5 Hz, 1H, H-5^G), 3.99-4.06 (m, 2H, H-2^F, H-3^F), 4.13 (dd, *J* = 8.1, 4.9 Hz, 1H, H-2^L), 4.22-4.35 (m, 2H, H-6a^G, CH_2Ph), 4.40 (dd, *J* = 11.5, 5.6 Hz, 1H, H-6b^G), 4.52 (d, *J* = 11.8 Hz, 1H, A of AB, CH_2Ph), 4.55 (d, *J* = 8.1 Hz, H-1^G), 4.62 (d, *J* = 11.8 Hz, 1H, B of AB, CH_2Ph), 4.66 (d, *J* = 11.5 Hz, 1H, A of AB, CH_2Ph), 4.71-4.85 (m, 3H, H-5^F, CH_2Ph), 4.90 (m, 1H, H-1^F), 5.05 (d, *J* = 12.1 Hz, 1H, A of AB, CH_2Ph), 5.12 (d, *J* = 12.1 Hz, 1H, B of AB, CH_2Ph), 5.61 (m, 1H, H-2^G), 5.84 (d, *J* = 3.3 Hz, 1H, H-4^G), 7.14-7.36, 7.40-7.48, 7.50-7.60, 8.00-8.15 (m, 35H, 7 C_6H_5); ^{13}C NMR (126 MHz, $CDCl_3$): δ = 16.86 (C-6^F), 21.92 (C-5^{cyc}), 25.52, 25.77, 26.13, 32.69, 33.27, 33.44, 40.52 ($C_7H_{13}{}^L$), 27.39 (C-4^{cyc}), 30.14 (C-6^{cyc}), 43.83 (C-3^{cyc}), 59.01 (OCH_3), 62.62 (C-6^G), 66.35 (C-5^F), 66.67 (CH_2Ph), 70.07, 70.26 (C-4^G, CH_2), 70.86 (CH_2), 71.55 (2C, H-5^G, CH_2), 71.83 (CH_2Ph), 72.11 (C-2^G), 74.23, 75.00 (2 CH_2Ph), 76.37 (2C, C-2^F, C-2^{cyc}), 78.11 (C-3^G), 78.50 (C-2^L), 79.26 (C-4^F), 80.01 (C-3^F), 80.86 (C-1^{cyc}), 97.64 (C-1^F), 100.07 (C-1^G), 127.03, 127.12, 127.28, 127.44, 127.88, 128.09, 128.14, 128.18, 128.40, 128.43, 128.48, 128.51, 128.54, 128.59, 129.71, 129.79, 129.95, 130.00, 133.09, 133.22, 133.29, 135.49, 138.77, 139.00, 139.21 (42C, 7 C_6H_5), 164.62, 166.16, 166.23, 172.48 (4 C=O); ESI-MS: *m/z*: Calcd for $C_{80}H_{90}NaO_{18}$ $[M+Na]^+$: 1361.6; found: 1361.9.

{(1*R*,2*R*,3*R*)-2-[(2,3,4-Tri-*O*-benzyl-6-deoxy-α-L-galactopyranosyl)oxy]-3-isobutylcyclohex-1-yl} 2,4,6-tri-*O*-benzoyl-3-*O*-[(1*S*)-1-benzyloxycarbonyl-2-cyclohexylethyl]-β-D-galactopyranoside (19m). According to general procedure M, **17m** (32 mg, 0.054 mmol) and **18** (51 mg, 0.065 mmol) in DCM (1.4 mL) were reacted with DMTST (42 mg, 0.163 mmol) in DCM (600 µL) for 16 h to give **19m** (56 mg, 79%). $[\alpha]_D^{20}$ -58.0 (*c* 1.12, DCM); ^{1}H NMR (500 MHz, $CDCl_3$): δ = 0.41-0.56 (m, 2H, $C_7H_{13}{}^L$), 0.63-1.48 (m, 16H, H-4a^{cyc}, H-5^{cyc}, H-6a^{cyc}, CH_2, $C_7H_{13}{}^L$), 0.72 (d, *J* = 6.5 Hz, 3H, CH_3), 0.79 (d, *J* = 6.9 Hz, 3H, CH_3), 1.35 (d, *J* = 6.4 Hz, 3H, H-6^F), 1.51-1.60 (m, 2H, H-3^{cyc}, CH), 1.65 (m, 1H, H-4b^{cyc}), 1.73 (ddd, *J* = 13.5, 9.9, 4.7 Hz, 1H, CH_2), 1.81 (m, 1H, H-6b^{cyc}), 3.38 (t, *J* = 7.5 Hz, 1H, H-2^{cyc}), 3.52 (m, 1H, H-4^F), 3.60 (m, 1H, H-1^{cyc}), 3.84 (dd, *J* = 10.1, 3.3 Hz, 1H, H-3^G), 3.92 (t, *J* = 6.6 Hz, 1H, H-5^G), 3.96-4.07 (m, 2H, H-2^F, H-3^F), 4.14 (dd, *J* = 7.8, 4.7 Hz, 1H, H-2^L), 4.26-4.35 (m, 2H, H-6a^G, CH_2Ph), 4.41 (dd, *J* = 11.5, 5.6 Hz, 1H, H-6b^G), 4.52-4.59 (m, 2H, H-1^G, CH_2Ph), 4.61-4.71 (m, 3H, H-5^F, CH_2Ph), 4.71-4.80 (m, 2H, CH_2Ph) , 5.05 (d, *J* = 12.1 Hz, 1H, A of AB, CH_2Ph), 5.08 (d, *J* = 3.2 Hz, 1H, H-1^F), 5.12 (d, *J* = 12.1 Hz, 1H, B of AB, CH_2Ph), 5.62 (m, 1H, H-2^G), 5.85 (d, *J* = 3.3 Hz, 1H, H-4^G), 7.11-7.49, 7.49-7.63, 7.93-8.20 (m, 35H, 7 C_6H_5); ^{13}C NMR (126 MHz, $CDCl_3$): δ = 16.78 (C-6^F), 20.77 (C-5^{cyc}), 20.94 , 23.85 (2 CH_3), 24.75 (CH), 25.46, 25.71, 26.08, 32.62, 33.22, 33.39, 40.46 ($C_7H_{13}{}^L$), 27.59 (C-4^{cyc}), 29.57 (C-6^{cyc}), 39.65 (C-3^{cyc}), 40.17 (CH_2), 62.59 (C-6^G), 66.45, 66.61 (C-5^F, CH_2Ph), 70.10 (C-4^G), 71.50 (C-5^G), 72.23, 72.31 (C-2^G, CH_2Ph), 73.65, 74.85 (2 CH_2Ph), 76.34 (C-2^F), 77.96 (C-3^G), 78.38 (C-2^L), 79.07 (C-4^F), 79.39 (C-2^{cyc}), 79.64 (C-3^F), 80.11 (C-1^{cyc}), 97.61 (C-1^F), 100.07 (C-1^G), 126.99, 127.06, 127.20, 127.22, 127.78, 127.94, 128.03, 128.04, 128.09, 128.36, 128.37, 128.41, 128.42, 128.48, 128.51, 128.53, 128.57, 129.64, 129.74, 129.81, 129.88, 129.91, 129.96, 133.00, 133.13, 133.20, 135.45, 138.80, 139.02, 139.17 (42C, 7 C_6H_5), 164.55, 166.01, 166.19, 172.44 (4 C=O); ESI-MS: *m/z*: Calcd for $C_{80}H_{90}NaO_{16}$ $[M+Na]^+$: 1329.6; found: 1329.8.

{(1*R*,2*R*,3*R*)-2-[(2,3,4-Tri-*O*-benzyl-6-deoxy-α-L-galactopyranosyl)oxy]-3-(2,2,2-trifluoromethyl)cyclo-hex-1-yl} 2,4,6-tri-*O*-benzoyl-3-*O*-[(1*S*)-1-benzyloxycarbonyl-2-cyclohexylethyl]-β-D-galactopyranoside

(19n). According to general procedure M, **17n** (20 mg, 0.032 mmol) and **18** (51 mg, 0.065 mmol) in DCM (2.0 mL) were reacted with DMTST (17 mg, 0.065 mmol) in DCM (2.0 mL) for 3 h to give **19n** (23.8 mg, 56%). $[\alpha]_D^{20}$ -53.1 (c 1.19, DCM); ^{1}H NMR (500 MHz, $CDCl_3$): δ = 0.44-0.58 (m, 2H), 0.68 (dd, *J* = 24.9, 12.4 Hz, 1H), 0.82 (m, 1H), 0.97-1.13 (m, 3H), 1.21 (d, *J* = 6.4 Hz, 3H, H-6^F), 1.14-1.46 (m, 9H), 1.56 (m, 1H), 1.70-1.79 (m, 2H), 1.88 (m, 1H), 2.03 (m, 1H, CH_2CF_3), 2.72 (m, 1H, CH_2CF_3), 3.45 (t, *J* = 6.0 Hz, 1H, H-2^{cyc}), 3.51 (d, *J* = 1.1 Hz, 1H, H-4^F), 3.67 (dd, *J* = 9.7, 6.5 Hz, 1H, H-1^{cyc}), 3.86 (dd, *J* = 10.0, 3.4 Hz, 1H, H-3^G), 3.88-3.94 (m, 2H, H-5^G, H-3^F), 3.97 (dd, *J* = 10.2, 3.6 Hz, 1H, H-2^F), 4.16 (dd, *J* = 8.1, 4.4 Hz, 1H, H-2^L), 4.25 (q, *J* = 6.2 Hz, 1H, H-5^F), 4.31 (dd, *J* = 11.5, 7.5 Hz, 1H, H-6b^G), 4.41 (dd, *J* = 11.6, 4.9 Hz, 1H, H-6a^G), 4.43 (d, *J* = 11.4 Hz, 1H, A of AB, CH_2Ph), 4.55 (d, *J* = 8.0 Hz, 1H, H-1^G), 4.56 (d, *J* = 11.5 Hz, 1H, A of AB, CH_2Ph), 4.61 (d, *J* = 11.8 Hz, 1H, A of AB, CH_2Ph), 4.72 (d, *J* = 11.8 Hz, 1H, B of AB, CH_2Ph), 4.73 (d, *J* = 11.5 Hz, 1H, B of AB, CH_2Ph), 4.85 (d, *J* = 11.7 Hz, 1H, B of AB, CH_2Ph), 4.86 (s, 1H, H-1^F), 5.05 (d, *J* = 12.1 Hz, 1H, A of AB, CH_2Ph), 5.15 (d, *J* = 12.1 Hz, 1H, B of AB, CH_2Ph), 5.60 (dd, *J* = 9.6, 8.4 Hz, 1H, H-2^G), 5.86 (d, *J* = 3.3 Hz, 1H, H-4^G), 7.15-7.41, 7.45 (td, *J* = 7.7, 4.2 Hz), 7.50-7.64, 8.02-8.05, 8.13 (d, *J* = 7.2 Hz, 35H, 7 C_6H_5); ^{13}C NMR (126 MHz, $CDCl_3$): δ = 16.74 (C-6^F), 17.47, 20.77, 23.81, 25.47, 25.75, 26.09, 27.72, 32.58, 33.27, 33.45, 34.30 (q, *J* = 27.4 Hz, *C*H_2CF_3), 34.52, 34.74, 40.46, 62.80 (C-6^G), 66.66 (CH_2Ph), 66.92 (C-5^F), 70.01 (C-4^G), 71.76 (C-5^G), 72.50 (C-2^G), 72.57, 73.75, 74.91 (3 CH_2Ph), 76.33 (C-2^F), 77.57 (C-3^G), 78.15 (C-2^L), 78.37 (C-1^{cyc}), 78.49 (C-4^F), 78.69 (C-2^{cyc}), 79.47 (C-3^F), 98.13 (C-1^F), 99.72 (C-1^G), 127.21, 127.28, 127.40, 127.49, 128.05, 128.07, 128.13, 128.20, 128.23, 128.43, 128.47, 128.49, 128.55, 128.58, 129.62, 129.64, 129.71, 129.72, 129.78, 130.06, 133.13, 133.23, 133.28, 135.47, 138.44, 138.92, 138.94 (42C, 7 C_6H_5), 164.74, 165.96, 166.16, 172.50 (4 C=O); ESI-MS: *m/z*: Calcd for $C_{78}H_{83}FNaO_{16}$ $[M+Na]^+$: 1355.6; found: 1355.6.

Experimental information for compounds **2b** and **2c** is given in reference.[8]

General procedure N:

{(1*R*,2*R*,3*S*)-2-[(α-L-Fucopyranosyl)oxy]-3-propylcyclohex-1-yl} 3-*O*-[sodium (1*S*)-1-carboxy-2-cyclohexylethyl]-β-D-galactopyranoside (2d). A mixture of **19d** (33 mg, 0.026 mmol) and $Pd(OH)_2$/C (10.0 mg) in dioxane/water (4:1, 1.25 mL) was stirred at rt under a hydrogen atmosphere. After 52 h the catalyst was filtered off and the solvents were evaporated. The residue was diluted in THF (1.2 mL) and LiOH (12 mg, 0.499 mmol) in H_2O (400 µL) was added at rt. After 4 d the reaction mixture was cooled to 0 °C, neutralized with 3 N aq. HCl and evaporated. Purification by column chromatography on silica gel (DCM/MeOH/H_2O, 10:3:0.5), followed by ion-exchange chromatography (Dowex, Na^+ form) and reversed-phase chromatography (RP18, H_2O/MeOH, 3:7) yielded **2d** (8.4 mg, 60%) as a white solid. $[\alpha]_D^{20}$ -51.7 (*c* 0.054, MeOH); ^{1}H NMR (500 MHz, CD_3OD): δ = 0.87-1.05, 1.15-1.50, 1.52-1.87, 1.88-2.01, 2.11-2.18 (m, 24H, H-3^{cyc}, H-4^{cyc}, H-5^{cyc}, H-6^{cyc}, $C_7H_{13}^L$, 2 CH_2), 0.94 (d, *J* = 7.0 Hz, 3H, CH_3), 1.23 (d, *J* = 6.7 Hz, 3H, H-6^F), 3.21 (dd, *J* = 9.4, 3.1 Hz, 1H, H-3^G), 3.37 (m, 1H, H-2^{cyc}), 3.45 (dd, *J* = 7.0, 4.7 Hz, 1H, H-5^G), 3.60-3.82 (m, 6H, H-2^F, H-4^F, H-2^G, H-6^G, H-1^{cyc}), 3.83-3.93 (m, 3H, H-3^F, H-4^G, H-2^L), 4.33 (d, *J* = 7.9 Hz, 1H, H-1^G), 4.85 (m, 1H, H-5^F), 5.00 (d, *J* = 3.9 Hz, 1H, H-1^F); ^{13}C NMR (126 MHz, CD_3OD): δ = 14.59 (CH_3), 16.79 (C-6^F), 20.58, 27.29, 27.58,

27.85, 33.54, 34.62, 34.82, 35.51, 43.34 ($C_7H_{13}^L$, 2 CH_2), 23.91 (C-5^{cyc}), 30.56 (C-4^{cyc}), 31.66 (C-6^{cyc}), 44.64 (C-3^{cyc}), 63.33 (C-6^G), 67.69, 67.70 (C-4^G, C-5^F), 70.31 (C-2^F), 71.32, 71.35 (C-2^G, C-3^F), 73.81 (C-4^F), 75.89 (C-5^G), 79.86 (C-1^{cyc}), 80.78 (C-2^L), 82.32 (C-2^{cyc}), 85.61 (C-3^G), 100.19 (C-1^F), 102.26 (C-1^G), 183.42 (C=O); HR-MS: *m/z*: Calcd for $C_{30}H_{52}NaO_{13}$ $[M+H]^+$: 643.3300; found: 643.3301.

{(1*R*,2*R*,3*R*)-3-Benzyl-[(α-L-fucopyranosyl)oxy]cyclohex-1-yl} 3-*O*-[sodium (1*S*)-1-carboxy-2-cyclohexylethyl]-β-D-galactopyranoside (2e). According to general procedure N, a mixture of **19e** (42 mg, 0.031 mmol) and $Pd(OH)_2$/C (18.0 mg) in THF (5.0 mL) was stirred under hydrogen for 3 h. Then, the catalyst was filtered off and the solvents were evaporated. The residue was diluted in MeOH/H_2O (3:1, 4.0 mL) and LiOH (40 mg) was added at rt. The reaction mixture was stirred at rt for 2 d, one benzoate was still left. Therefore, stirring was continued at 45°C for 20 h. Then, the mixture was cooled to 0 °C, neutralized with HOAc and evaporated. Purification by column chromatography on silica gel (DCM/(MeOH/H_2O, 10:1), 6:1 to 3:1), followed by ion-exchange chromatography (Dowex, Na^+ form) and reversed-phase chromatography (RP18, H_2O/MeOH, 3:7) yielded **2e** (8.4 mg, 60%) as a white solid. $[\alpha]_D^{20}$ -80.5 (c 0.18, MeOH); ^1H NMR (500 MHz, CD_3OD): δ = 1.22 (d, J = 6.5 Hz, 3H, H-6^F), 0.87-1.51 (m, 10H), 1.53-1.85 (m, 9H), 1.93 (m, 1H), 2.10-2.16 (m, 2H), 3.25 (dd, J = 9.4, 3.0 Hz, 1H, H-3^G), 3.40 (t, J = 9.2Hz, 1H, H-2^{cyc}), 3.43 (m, 1H, H-5^G), 3.61-3.81 (m, 8H, H-2^F, H-1^{cyc}, H-6^G, H-4^F, H-2^G, $PhCH_2$), 3.88-3.92 (m, 2H, H-3^F, H-4^G), 4.17 (dd, J = 9.5, 3.2 Hz, 1H, H-2^L), 4.31 (d, J = 7.8 Hz, 1H, H-1^G), 4.87 (m, 1H, H-5^F), 5.08 (d, J = 4.0 Hz, 1H, H-1^F), 6.98-7.31 (m, 5H, C_6H_5); ^{13}C NMR (126 MHz, CD_3OD): δ = 16.86 (C-6^F), 23.53, 27.51, 27.24, 27.75, 30.21, 31.63, 33.54, 34.75, 35.31, 39.38, 42.81, 63.02 (12C, $C_7H_{13}^L$, $PhCH_2$), 67.80 (C-5^F), 68.11 (C-4^G), 70.31 (C-2^F), 71.41 (C-3^F), 71.74 (C-2^G), 73.84 (C-4^F), 75.85 (C-5^G), 79.75 (2C, C-1^{cyc}, C-2^L), 83.64 (C-2^{cyc}), 84.77 (C-3^G), 100.83 (C-1^F), 102.30 (C-1^G), 126.71, 129.13, 130.31, 142.50 (6C, C_6H_5); HR-MS: *m/z*: Calcd for $C_{34}H_{52}NaO_{13}$ $[M+H]^+$: 691.3300; found: 691.3299.

General procedure O:

{(1*R*,2*R*,3*R*)-3-(Cyclohexylmethyl)-[(α-L-fucopyranosyl)oxy]cyclohex-1-yl} 3-*O*-[sodium (1*S*)-1-carboxy-2-cyclohexylethyl]-β-D-galactopyranoside (2f). To a solution of **19f** (42 mg) in MeOH/H_2O (4:1, 5.0 mL) was added LiOH (57 mg), the reaction mixture was stirred at 50 °C overnight and filtered through cotton. The filtrate was neutralized with HOAc, concentrated, and purified by column chromatography on silica gel (DCM/MeOH, 15:1 to 9:1) to provide the crude intermediate (14 mg) which was used in the next step without further purification. A suspension of the intermediate (14 mg) and $Pd(OH)_2$/C (4.1 mg, 10% Pd) in THF (3.0 mL) was hydrogenated with (1 bar H_2, balloon) at rt overnight. The reaction mixture was filtered through celite and the solvent was removed under reduced pressure. The residue was purified by silica gel chromatography (DCM/(MeOH:H_2O, 10:1), 4:1 to 7:3), followed by ion-exchange chromatography (Dowex, Na^+ form) and reversed-phase chromatography (RP18, H_2O/MeOH, 9:1 to 1:1) to afford **2f** (8.2 mg, 82%) as white solid. $[\alpha]_D^{20}$ -46.3 (c 0.41, MeOH); ^1H NMR (500 MHz, CD_3OD): δ = 0.76-1.09 (m, 6H), 1.20 (d, J = 6.5 Hz, 3H, H-6^F), 1.11-1.45 (m, 9H), 1.53-1.87 (m, 15H), 1.88-2.02 (m, 2H), 2.11 (m, 1H), 3.19 (m, 1H, H-3^G), 3.25 (t, J = 9.0 Hz, 1H, C-2^{cyc}), 3.43 (m, 1H, H-5^G), 3.59-3.80 (m, 5H, H-2^F, H-6^G, H-4^F, H-1^{cyc}), 3.84-

3.86 (m, 2H, H-3^F, H-4^G), 3.97 (m, 1H, H-2^L), 4.29 (d, J = 7.7 Hz, 1H, H-1^G), 4.83 (m, 1H, H-5^F), 4.99 (d, J = 4.0 Hz, 1H, H-1^F); ^{13}C NMR (126 MHz, CD_3OD): δ = 16.83 (C-6^F), 23.73, 27.27, 27.33, 27.56, 27.65, 27.82, 27.87, 30.46, 31.60, 32.92, 33.54, 34.80, 35.45, 35.85, 36.23, 40.45, 41.86, 43.19, (18C, $C_7H_{13}{}^L$, C^{cyc}), 63.29 (C-6^G), 67.68 (C-5^F), 67.81(C-4^G), 70.30 (C-2^F), 71.36 (C-3^F), 71.45 (C-2^G), 73.82 (C-4^F), 75.92 (C-5^G), 79.94 (C-1^{cyc}, C-2^L), 83.29 (C-2^{cyc}), 85.38 (C-3^G), 100.63 (C-1^F), 102.38 (C-1^G); HR-MS: *m/z*: Calcd for $C_{34}H_{58}NaO_{13}$ [M+H]$^+$: 697.3770; found: 697.3771.

{(1*R*,2*R*,3*R*)-[(α-L-Fucopyranosyl)oxy]-3-phenylcyclohex-1-yl} 3-*O*-[sodium (1*S*)-1-carboxy-2-cyclohexylethyl]-β-D-galactopyranoside (2g). According to general procedure N, **19g** (34 mg, 0.026 mmol) was hydrogenated with $Pd(OH)_2/C$ (14.0 mg) in dioxane/water (4:1, 2.0 mL) for 20 h, and then dissolved in THF (1.0 mL) and treated with LiOH (11 mg, 0.459 mmol) in H_2O (300 µL) for 7 d to give **2g** (8.8 mg, 52%). $[\alpha]_D^{20}$ -47.8 (*c* 0.046, MeOH); ^{1}H NMR (500 MHz, CD_3OD): δ = 0.86-1.07, 1.15-1.27, 1.27-1.87, 1.87-1.99 (m, 18H, H-4^{cyc}, H-5^{cyc}, H-6a^{cyc}, $C_7H_{13}{}^L$), 1.12 (d, J = 6.6 Hz, 3H, H-6^F), 2.24 (dt, J = 12.6, 3.5 Hz, 1H, H-6b^{cyc}), 2.74 (ddd, J = 13.4, 9.7, 4.0 Hz, 1H, H-3^{cyc}), 3.22 (dd, J = 9.4, 3.2 Hz, 1H, H-3^G), 3.26 (dd, J = 10.1, 4.0 Hz, 1H, H-2^F), 3.47 (ddd, J = 7.1, 4.6, 1.2 Hz, 1H, H-5^G), 3.59 (dd, J = 9.4, 7.8 Hz, 1H, H-2^G), 3.64 (dd, J = 3.5, 1.2 Hz, 1H, H-4^F), 3.69 (dd, J = 11.6, 4.5 Hz, 1H, H-6a^G), 3.79 (dd, J = 11.6, 7.2 Hz, 1H, H-6b^G), 3.81-3.91 (m, 5H, H-3^F, H-4^G, H-2^L, H-1^{cyc}, H-2^{cyc}), 3.93 (d, J = 4.0 Hz, 1H, H-1^F), 4.38 (d, J = 7.8 Hz, 1H, H-1^G), 4.75 (m, 1H, H-5^F), 7.17-7.23, 7.26-7.35 (m, 5H, C_6H_5); ^{13}C NMR (126 MHz, CD_3OD): δ = 16.53 (C-6^F), 24.58, 27.30, 27.59, 27.86, 31.75, 33.53, 34.82, 35.47, 35.50, 43.33 (C-4^{cyc}, C-5^{cyc}, C-6^{cyc}, $C_7H_{13}{}^L$), 52.02 (C-3^{cyc}), 63.33 (C-6^G), 67.23 (C-5^F), 67.67 (C-4^G), 69.98 (C-2^F), 71.15 (C-3^F), 71.57 (C-2^G), 73.76 (C-4^F), 75.93 (C-5^G), 80.07, 80.23, 80.74 (C-2^L, C-1^{cyc}, C-2^{cyc}), 85.61 (C-3^G), 97.58 (C-1^F), 102.16 (C-1^G), 127.59, 129.49, 144.88 (6C, C_6H_5), 183.39 (C=O); HR-MS: *m/z*: Calcd for $C_{33}H_{50}O_{13}$ [M+H]$^+$: 677.3144; found: 677.3145.

{(1*R*,2*R*,3*R*)-3-*tert*-Butyl-[(α-L-fucopyranosyl)oxy]cyclohex-1-yl} 3-*O*-[sodium (1*S*)-1-carboxy-2-cyclohexylethyl]-β-D-galactopyranoside (2h). According to general procedure N, **19h** (17 mg, 0.013 mmol) was hydrogenated with $Pd(OH)_2/C$ (8.0 mg) in dioxane/water (4:1, 1.0 mL) for 20 h, and then dissolved in dioxane (500 µL) and treated with 3 N aq. NaOH (32 µL) to give **2h** (4.7 mg, 58%). $[\alpha]_D^{20}$ -100.0 (*c* 0.12, MeOH); ^{1}H NMR (500 MHz, CD_3OD): δ = 0.98 (s, 9H, $C(CH_3)_3$), 0.84-1.06, 1.13-1.25, 1.26-1.39, 1.39-1.51, 1.51-1.85, 1.88-1.97 (m, 20 H, H-3^{cyc}, H-4^{cyc}, H-5^{cyc}, H-6^{cyc}, $C_7H_{13}{}^L$), 1.21 (d, J = 6.6 Hz, H-6^F), 3.21 (dd, J = 9.3, 3.3 Hz, 1H, H-3^G), 3.50 (td, J = 6.1, 1.3 Hz, 1H, H-5^G), 3.62 (dd, J = 9.3, 7.8 Hz, 1H, H-2^G), 3.67 (dd, J = 3.0, 1.2 Hz, 1H, H-4^F), 3.69-3.74 (m, 2H, H-2^F, H-3^F), 3.74-3.79 (m, 2H, H-6^G), 3.87 (dd, J = 9.8, 3.7 Hz, 1H, H-2^L), 3.91 (dd, J = 3.3, 1.2 Hz, 1H, H-4^G), 3.94 (t, J = 3.6 Hz, 1H, H-2^{cyc}), 4.03 (dt, J = 7.3, 6.0 Hz, 1H, H-5^F), 4.14 (q, J = 3.2 Hz, 1H, H-1^{cyc}), 4.35 (d, J = 7.9 Hz, 1H, H-1^G), 4.99 (d, J = 3.5 Hz, 1H, H-1^F); ^{13}C NMR (126 MHz, CD_3OD): δ = 16.70 (C-6^F), 19.60, 21.21, 23.51, 27.25, 27.55, 27.86, 29.34, 33.50, 33.75, 34.72, 35.52, 43.38, 51.15 (C-3^{cyc}, C-4^{cyc}, C-5^{cyc}, C-6^{cyc}, $C_7H_{13}{}^L$, $\mathit{C}(CH_3)_3$, $C(\mathit{C}H_3)_3$), 62.70 (C-6^G), 67.44 (C-4^G), 68.00 (C-5^F), 70.07 (C-2^F), 71.55, 71.62 (C-2^G, C-3^F), 73.66 (C-4^F), 75.92 (C-5^G), 77.76 (C-1^{cyc}), 80.78 (2C, C-2^{cyc}, C-2^L), 85.89 (C-3^G), 100.69 (C-1^F), 103.71 (C-1^G), 183.39 (C=O); HR-MS: *m/z*: Calcd for $C_{31}H_{54}NaO_{13}$ [M+H]$^+$: 657.3457; found: 657.3456.

{(1*R*,2*R*,3*S*)-3-Butyl-[(α-L-fucopyranosyl)oxy]cyclohex-1-yl} 3-*O*-[sodium (1*S*)-1-carboxy-2-cyclohexyl-ethyl]-β-D-galactopyranoside (2i). According to general procedure N, **19i** (18.7 mg, 0.014 mmol) was hydrogenated with $Pd(OH)_2$/C (5.0 mg) in dioxane/water (4:1, 1.0 mL) for 20 h, and then dissolved in THF (600 µL) and treated with LiOH (5.3 mg, 0.222 mmol) in H_2O (200 µL) for 4 d to give **2i** (4.5 mg, 46%). $[\alpha]_D^{20}$ -55.6 (*c* 0.22, MeOH); ^{1}H NMR (500 MHz, CD_3OD): δ = 0.92 (t, *J* = 7.1 Hz, 3H, CH_3), 0.85-1.04, 1.12-1.42, 1.46-1.84, 1.84-2.03, 2.08-2.19 (m, 26H, H-3^{cyc}, H-4^{cyc}, H-5^{cyc}, H-6^{cyc}, $C_7H_{13}{}^L$, 3 CH_2), 1.20 (d, *J* = 6.6 Hz, 3H, H-6^F), 3.19 (m, 1H, H-3^G), 3.33 (m, 1H, H-2^{cyc}), 3.43 (ddd, *J* = 11.8, 4.9, 3.1 Hz, 1H, H-5^G), 3.60-3.78 (m, 6H, H-1^{cyc}, H-2^G, H-6^G, H-2^F, H-4^F), 3.82-3.90 (m, 3H, H-3^F, H-4^G, H-2^L), 4.30 (d, *J* = 7.8 Hz, 1H, H-1^G), 4.86 (m, 1H, H-5^F), 4.98 (d, *J* = 4.1 Hz, 1H, H-1^F); ^{13}C NMR (126 MHz, CD_3OD): δ = 14.51 (CH_3), 16.80 (C-6^F), 23.93, 23.98, 27.29, 27.58, 27.85, 29.82, 30.61, 31.68, 32.05, 33.53, 34.82, 35.50, 43.33, 44.87 (C-3^{cyc}, C-4^{cyc}, C-5^{cyc}, C-6^{cyc}, $C_7H_{13}{}^L$, 3 CH_2), 63.33 (C-6^G), 67.70 (2C, C-4^G, C-5^F), 70.29 (C-2^F), 71.35 (2C, C-2^G, C-3^F), 73.81 (C-4^F), 75.89 (C-5^G), 79.89 (C-1^{cyc}), 80.77 (C-2^L), 82.31 (C-2^{cyc}), 85.59 (C-3^G), 100.21 (C-1^F), 102.27 (C-1^G), 183.42 (C=O); HR-MS: *m/z*: Calcd for $C_{31}H_{54}NaO_{13}$ $[M+H]^+$: 657.3457; found: 657.3457.

{(1*R*,2*R*,3*S*)-[(α-L-Fucopyranosyl)oxy]-3-hexylcyclohex-1-yl} 3-*O*-[sodium (1*S*)-1-carboxy-2-cyclo-hexylethyl]-β-D-galactopyranoside (2j). According to general procedure N, **19j** (44 mg, 0.033 mmol) was hydrogenated with $Pd(OH)_2$/C (10.0 mg) in dioxane/water (4:1, 1.5 mL) for 20 h, and then dissolved in THF (600 µL) and treated with LiOH (12.0 mg, 0.492 mmol) in H_2O (200 µL) for 6 d to give **2j** (10.2 mg, 51%). $[\alpha]_D^{20}$ -81.1 (*c* 0.064, MeOH); ^{1}H NMR (500 MHz, CD_3OD): δ = 0.88-0.94 (m, 3H, CH_3), 0.94-1.05 (m, 3H, $C_7H_{13}{}^L$, CH_2), 1.22 (d, *J* = 6.6 Hz, 3H, H-6^F), 1.24-1.46 (m, 14H, H-4a^{cyc}, H-5a^{cyc}, H-6a^{cyc}, $C_7H_{13}{}^L$, CH_2), 1.50-1.87 (m, 10H, H-3^{cyc}, H-4b^{cyc}, H-5b^{cyc}, $C_7H_{13}{}^L$, CH_2), 1.86-2.07 (m, 2H, $C_7H_{13}{}^L$, CH_2), 2.14 (dd, *J* = 12.7, 4.7 Hz, 1H, H-6b^{cyc}), 3.22 (m, 1H, H-3^G), 3.35 (m, 1H, H-2^{cyc}), 3.45 (dd, *J* = 6.9, 4.7 Hz, 1H, H-5^G), 3.60-3.83 (m, 6H, H-1^{cyc}, H-2^F, H-4^F, H-2^G, H-6^G), 3.84-3.90 (m, 2H, H-3^F, H-4^G), 3.94 (m, 1H, H-2^L), 4.33 (d, *J* = 7.7 Hz, 1H, H-1^G), 4.83 (m, 1H, H-5^F), 5.01 (d, *J* = 4.0 Hz, 1H, H-1^F); ^{13}C NMR (126 MHz, CD_3OD): δ = 14.44 (CH_3), 16.82 (C-6^F), 23.76 (C-5^{cyc}), 23.93, 27.29, 27.58, 27.85, 30.65, 30.72, 32.43, 33.11, 33.56, 34.80, 35.48, 43.25 ($C_7H_{13}{}^L$, CH_2), 27.53 (C-4^{cyc}), 31.67 (C-6^{cyc}), 44.81 (C-3^{cyc}), 63.26 (C-6^G), 67.73 (C-5^F), 67.76 C-4^G), 70.28 (C-2^F), 71.36 (C-3^F), 71.38 (C-2^G), 73.82 (C-4^F), 75.84 (C-5^G), 79.84 (C-1^{cyc}), 82.34 (C-2^{cyc}), 85.45 (C-3^G), 100.06 (C-1^F), 102.27 (C-1^G); HR-MS: *m/z*: Calcd for $C_{33}H_{58}NaO_{13}$ $[M+H]^+$: 685.3770; found: 685.3769.

{(1*R*,2*R*,3*S*)-[(α-L-Fucopyranosyl)oxy]-3-(3-phenylpropyl)cyclohex-1-yl} 3-*O*-[sodium (1*S*)-1-carboxy-2-cyclohexylethyl]-β-D-galactopyranoside (2k). According to general procedure N, **19k** (10 mg, 0.007 mmol) was hydrogenated with $Pd(OH)_2$/C (5.0 mg) in dioxane/water (4:1, 1.0 mL) for 4 h, and then dissolved in dioxane (500 µL) and treated with 3 N aq. NaOH (14.5 µL) for 12 d to give **2k** (2.8 mg, 55%). $[\alpha]_D^{20}$ -41.7 (*c* 0.32, MeOH); ^{1}H NMR (500 MHz, CD_3OD): δ = 1.19 (d, *J* = 6.5 Hz, 3H, H-6^F), 0.81-1.06, 1.16-1.41, 1.44-1.83, 1.85-1.95, 1.96-2.07 (m, 24H, H-3^{cyc}, H-4^{cyc}, H-5^{cyc}, H-6^{cyc}, $C_7H_{13}{}^L$, 2 CH_2), 2.56 (ddd, *J* = 13.6, 9.3, 6.3 Hz, 1H, CH_2), 2.64 (ddd, *J* = 14.9, 9.4, 5.9 Hz, 1H, CH_2), 3.18 (dd, *J* = 9.3, 3.2 Hz, 1H, H-3^G), 3.33 (m, 1H, H-2^{cyc}), 3.43 (m, 1H, H-5^G), 3.59-3.78 (m, 6H, H-2^G, H-6^G, H-2^F, H-4^F, H-1^{cyc}), 3.80-3.89 (m, 3H, H-2^L, H-3^F,

H-4^{G}), 4.30 (d, J = 7.8 Hz, 1H, H-1^{G}), 4.75 (m, 1H, H-5^{F}), 4.98 (d, J = 4.1 Hz, 1H, H-1^{F}), 7.10-7.19, 7.21-7.28 (m, 5H, C_6H_5); ^{13}C NMR (126 MHz, CD_3OD): δ = 16.81 (C-6^{F}), 23.79 (C-5^{cyc}), 27.29, 27.58, 27.85, 29.85, 32.28, 33.53, 34.82, 35.51, 37.31 43.34 ($C_7H_{13}{}^{L}$, 3 CH_2), 30.62 (C-4^{cyc}), 31.54 (C-6^{cyc}), 44.64 (C-3^{cyc}), 63.30 (C-6^{G}), 67.68, 67.73 (C-4^{G}, C-5^{F}), 70.28 (C-2^{F}), 71.31, 71.36 (C-2^{G}, C-3^{F}), 73.78 (C-4^{F}), 75.90 (C-5^{G}), 79.60 (C-1^{cyc}), 80.79 (C-2^{L}), 82.19 (C-2^{cyc}), 85.58 (C-3^{G}), 99.98 (C-1^{F}), 102.20 (C-1^{G}), 126.62, 129.25, 129.42, 144.06 (6C, C_6H_5), 183.40 (C=O); HR-MS: *m/z*: Calcd for $C_{36}H_{56}NaO_{13}$ $[M+H]^+$: 719.3613; found: 719.3613.

{(1*R*,2*R*,3*R*)-[(α-L-Fucopyranosyl)oxy]-3-((2-methoxyethoxy)methyl)cyclohex-1-yl} 3-*O*-[sodium (1*S*)-1-carboxy-2-cyclohexylethyl]-β-D-galactopyranoside (2l). According to general procedure N, **19l** (17 mg, 0.013 mmol) was hydrogenated with $Pd(OH)_2$/C (8.0 mg) in dioxane/water (4:1, 1.0 mL) for 20 h, and then dissolved in dioxane (500 µL) and treated with 3 N aq. NaOH (25 µL) for 2 d to give **2l** (7.6 mg, 83%). $[\alpha]_D^{20}$ -58.9 (*c* 0.28, MeOH); ^{1}H NMR (500 MHz, CD_3OD): δ = 0.86-1.06, 1.09-1.48, 1.51-1.83, 1.88-1.99, 2.09-2.18 (m, 20H, $C_7H_{13}{}^{L}$, H-3^{cyc}, H-4^{cyc}, H-5^{cyc}, H-6^{cyc}), 1.22 (d, J = 6.6 Hz, 3H, H-6^{F}), 3.20 (dd, J = 9.4, 3.2 Hz, 1H, H-3^{G}), 3.38 (s, 3H, OCH_3), 3.44 (m, 1H, H-5^{G}), 3.49 (dd, J = 9.4, 2.4 Hz, 1H, CH_2), 3.53-3.62 (m, 5H, H-2^{cyc}, 2 CH_2), 3.61-3.81 (m, 6H, H-2^{G}, H-6^{G}, H-2^{F}, H-4^{F}, H-1^{cyc}), 3.82-3.96 (m, 4H, H-2^{L}, H-3^{F}, H-4^{G}, CH_2), 4.31 (d, J = 7.8 Hz, 1H, H-1^{G}), 4.84 (m, 1H, H-5^{F}), 4.95 (d, J = 4.0 Hz, 1H, H-1^{F}); ^{13}C NMR (126 MHz, CD_3OD): δ = 16.78 (C-6^{F}), 23.90, 27.28, 27.58, 27.85, 29.72, 31.62, 33.54, 34.82, 35.50, 43.33, 45.74 (C-3^{cyc}, C-4^{cyc}, C-5^{cyc}, C-6^{cyc}, $C_7H_{13}{}^{L}$), 59.17 (OCH_3), 63.34 (C-6^{G}), 67.55 (C-5^{F}), 67.68 (C-4^{G}), 70.31 (C-2^{F}), 71.16 (CH_2), 71.33, 71.41 (C-2^{G}, C-3^{F}), 72.26, 72.82 (2 CH_2), 73.86 (C-4^{F}), 75.82 (C-5^{G}), 79.22 (C-2^{cyc}), 80.10 (C-1^{cyc}), 80.82 (C-2^{L}), 85.58 (C-3^{G}), 100.12 (C-1^{F}), 102.36 (C-1^{G}), 183.43 (C=O); HR-MS: *m/z*: Calcd for $C_{31}H_{54}NaO_{15}$ $[M+H]^+$: 689.3355; found: 689.3355.

{(1*R*,2*R*,3*R*)-[(α-L-Fucopyranosyl)oxy]-3-isobutylcyclohex-1-yl} 3-*O*-[sodium (1*S*)-1-carboxy-2-cyclohexylethyl]-β-D-galactopyranoside (2m). According to general procedure N, **19m** (56 mg, 0.043 mmol) was hydrogenated with $Pd(OH)_2$/C (20.0 mg) in dioxane/water (4:1, 2.0 mL) for 68 h, and then dissolved in dioxane (500 µL) and treated with 3 N aq. NaOH (38 µL) for 3 d to give **2m** (8.5 mg, 62%). $[\alpha]_D^{20}$ -92.0 (*c* 0.042, MeOH); ^{1}H NMR (500 MHz, CD_3OD): δ = 0.80-1.46, 1.53-2.01 (m, 22H, CH, CH_2, H-3^{cyc}, H-4^{cyc}, H-5^{cyc}, H-6a^{cyc}, $C_7H_{13}{}^{L}$), 0.88 (d, J = 6.6 Hz, 3H, CH_3), 0.95 (d, J = 6.9 Hz, 3H, CH_3), 1.23 (d, J = 6.6 Hz, 3H, H-6^{F}), 2.14 (dd, J = 13.5, 4.2 Hz, 1H, H-6b^{cyc}), 3.21 (dd, J = 9.5, 2.9 Hz, 1H, H-3^{G}), 3.29 (t, J = 9.1 Hz, 1H, H-2^{cyc}), 3.45 (t, J = 5.9 Hz, 1H, H-5^{G}), 3.61-3.83 (m, 6H, H-2^{F}, H-4^{F}, H-2^{G}, H-6^{G}, H-1^{cyc}), 3.83-3.94 (m, 3H, H-3^{F}, H-4^{G}, H-2^{L}), 4.33 (d, J = 7.9 Hz, 1H, H-1^{G}), 4.85 (m, 1H, H-5^{F}), 5.03 (d, J = 4.0 Hz, 1H, H-1^{F}); ^{13}C NMR (126 MHz, CD_3OD): δ = 16.81 (C-6^{F}), 21.29 (CH_3), 23.72 (C-5^{cyc}), 24.86 (CH_3), 26.06 (CH), 27.28, 27.57, 27.84, 33.53, 34.80, 35.49, 42.05, 42.64 ($C_7H_{13}{}^{L}$, CH_2), 30.28 (C-4^{cyc}), 31.57 (C-6^{cyc}), 43.32 (C-3^{cyc}), 63.36 (C-6^{G}), 67.69 (2C, C-4^{G}, C-5^{F}), 70.29 (C-2^{F}), 71.33 (2C, C-2^{G}, C-3^{F}), 73.80 (C-4^{F}), 75.89 (C-5^{G}), 79.86 (C-1^{cyc}), 80.77 (C-2^{L}), 83.23 (C-2^{cyc}), 85.60 (C-3^{G}), 100.51 (C-1^{F}), 102.32 (C-1^{G}), 183.41 (C=O); HR-MS: *m/z*: Calcd for $C_{31}H_{54}NaO_{13}$ $[M+H]^+$: 657.3457; found: 657.3459.

{(1*R*,2*R*,3*R*)-[(α-L-Fucopyranosyl)oxy]-3-(2,2,2-trifluoroethyl)cyclohex-1-yl} 3-*O*-[sodium (1*S*)-1-carboxy-2-cyclohexylethyl]-β-D-galactopyranoside (2n). According to general procedure O, **19n** (23.8 mg, 0.0178 mmol) was treated with LiOH (22 mg) in MeOH/H_2O (4:1, 2.5 mL) at 55 °C for 24 h, and then hydrogenated with $Pd(OH)_2$/C (6.5 mg) in THF (2.5 mL) to give **2n** (7.4 mg, 61%). $[\alpha]_D^{20}$ -92.0 (*c* 0.042, MeOH); ^{1}H NMR (500 MHz, CD_3OD): δ = 0.84-1.04 (m, 2H), 1.21 (d, *J* = 6.5 Hz, 3H, H-6^F), 1.12-1.37 (m, 5H), 1.47 (m, 1H), 1.54-1.80 (m, 8H), 1.87-2.02 (m, 3H), 2.06-2.23 (m, 2H), 2.95 (m, 1H, CH_2CF_3), 3.19 (dd, *J* = 9.0, 2.3 Hz, 1H, H-3^G), 3.39-3.50 (m, 2H, H-5^G, H-2^{cyc}), 3.63-3.69 (m, 3H, H-4^F, H-6b^G, H-2^G), 3.71-3.79 (m, 3H, H-1^{cyc}, H-2^F, H-6a^G), 3.81 (dd, *J* = 10.3, 3.1 Hz, 1H, H-3^F), 3.86 (s, 1H, H-4^G), 3.88 (s, 1H, H-2^L), 4.31 (d, *J* = 7.9 Hz, 1H, H-1^G), 4.61 (q, *J* = 6.3 Hz, 1H, H-5^F), 4.88 (d, *J* = 3.8 Hz, 1H, H-1^F); ^{13}C NMR (126 MHz, CD_3OD): δ = 16.83 (C-6^F), 21.82, 27.29, 27.58, 27.85, 29.29, 30.22, 33.52, 34.81, 35.51, 37.33, 38.20, 43.34, 63.16 (C-6^G), 67.65 (C-4^G), 68.04 (C-5^F), 70.11 (C-2^F), 71.31, 71.34 (C-3^F, C-2^G), 73.74 (C-4^G), 75.98 (C-5^G), 78.89 (C-1^{cyc}), 80.60 (C-2^L), 81.69 (C-2^{cyc}), 85.61 (C-3^G), 100.84 (C-1^F), 102.47 (C-1^G); ^{19}F NMR (471 MHz, CD_3OD): δ = -63.85; HR-MS: *m*/*z*: Calcd for $C_{29}H_{47}F_3NaO_{13}$ $[M+H]^+$: 683.2861; found: 683.2862.

2. Labeling of E-selectinSCR2

E-selectinSCR2 was labeled using the amine reactive protein labeling kit BLUE-NHS. Buffer exchange and labeling were performed according to the manufacturer's protocol. To protect the lysines in the binding site from being labeled, the protein was saturated with 600 µM of compound **31**. The labeled protein was dialyzed overnight against assay buffer (10 mM HEPES pH 7.4, 150 mM NaCl, 1 mM $CaCl_2$) using Slide-A-Lyzer dialysis cassettes (10 kDa MWCO). Protein concentration was determined by HPLC-UV against a BSA standard.

3. Microscale thermophoresis

MST experiments were carried out at 25 °C with 100% LED power, 50% laser power, a laser on time of 30 s, and a laser off time of 5 s using standard treated capillaries. Ligands were dissolved in assay buffer supplemented with 0.05% v/v Tween20 and titrated 1:1 for a total of 16 dilution steps. The dilution series of ligand was 1:1 mixed with a solution of 0.2 µM labeled E-SelectinSCR2 and incubated for 10 min at room temperature before measurement. Datapoints were normalized using the bound and unbound borders achieved by NanoTemper Analysis 1.2.205 software (NanoTemper Technologies GmbH, Munich, Germany) and analyzed/illustrated with GraphPad Prism 5.0 (GraphPad Software, La Jolla, CA, USA). The measurements were globally fitted using equation 1 for single site binding.[9]

$$[PL]=\frac{(C_P+ C_L+K_D) - \sqrt{(C_P+C_L+K_D) - 4C_PC_L}}{2C_P} \qquad \text{(eq. 1)}$$

where [PL] is the protein-ligand complex concentration and K_D is the dissociation constant. CP represents the total concentration of protein and CL the total concentration of ligand.

5. ^{19}F NMR of Mosher-derivatives

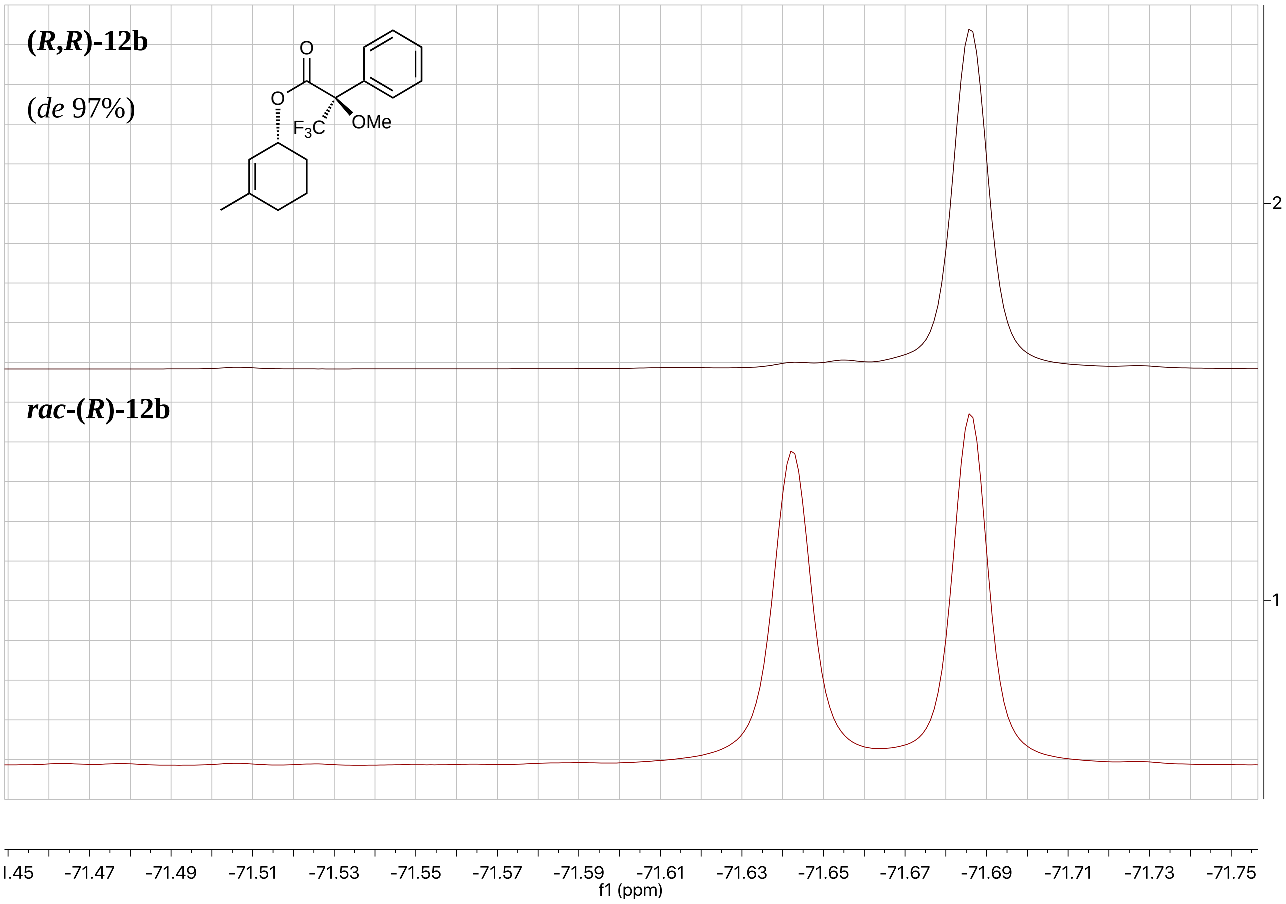

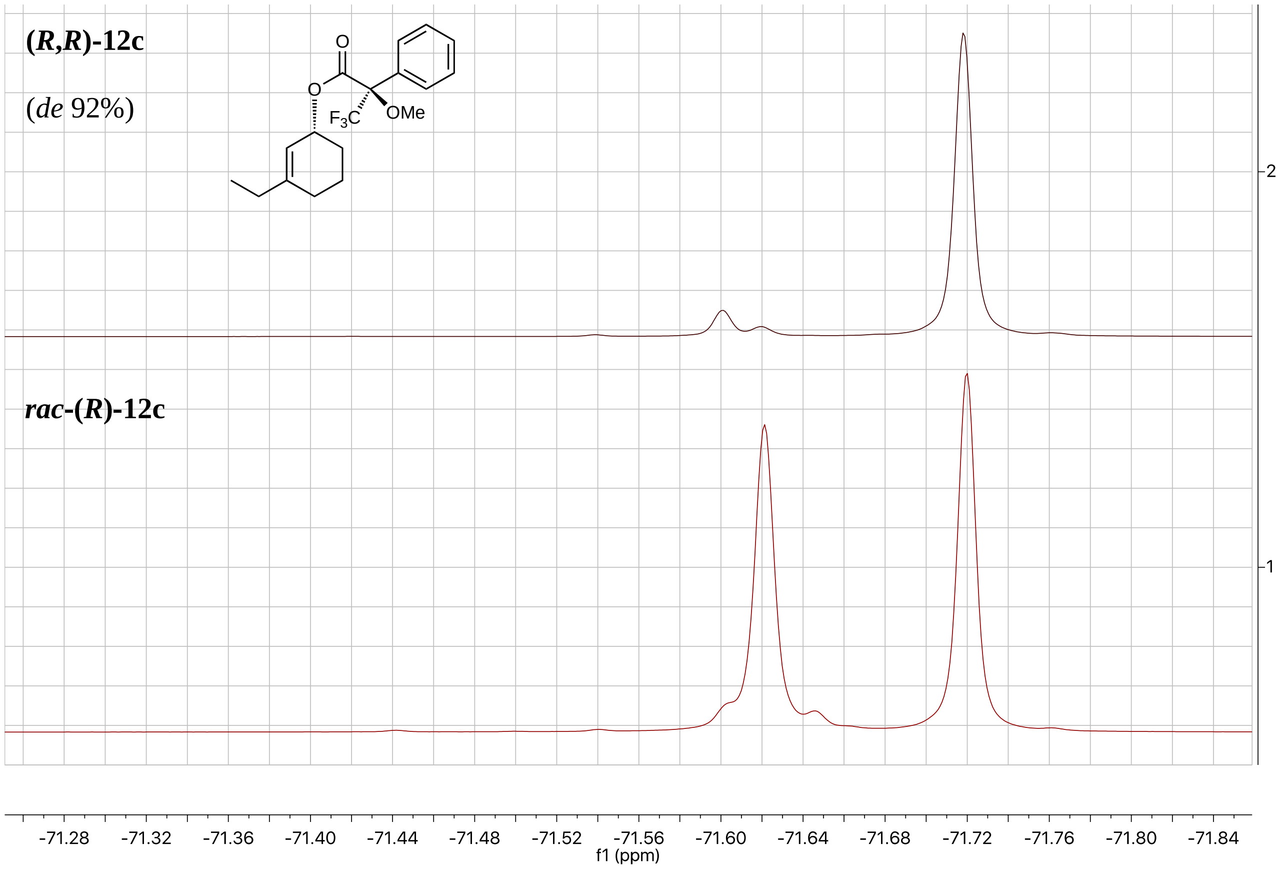

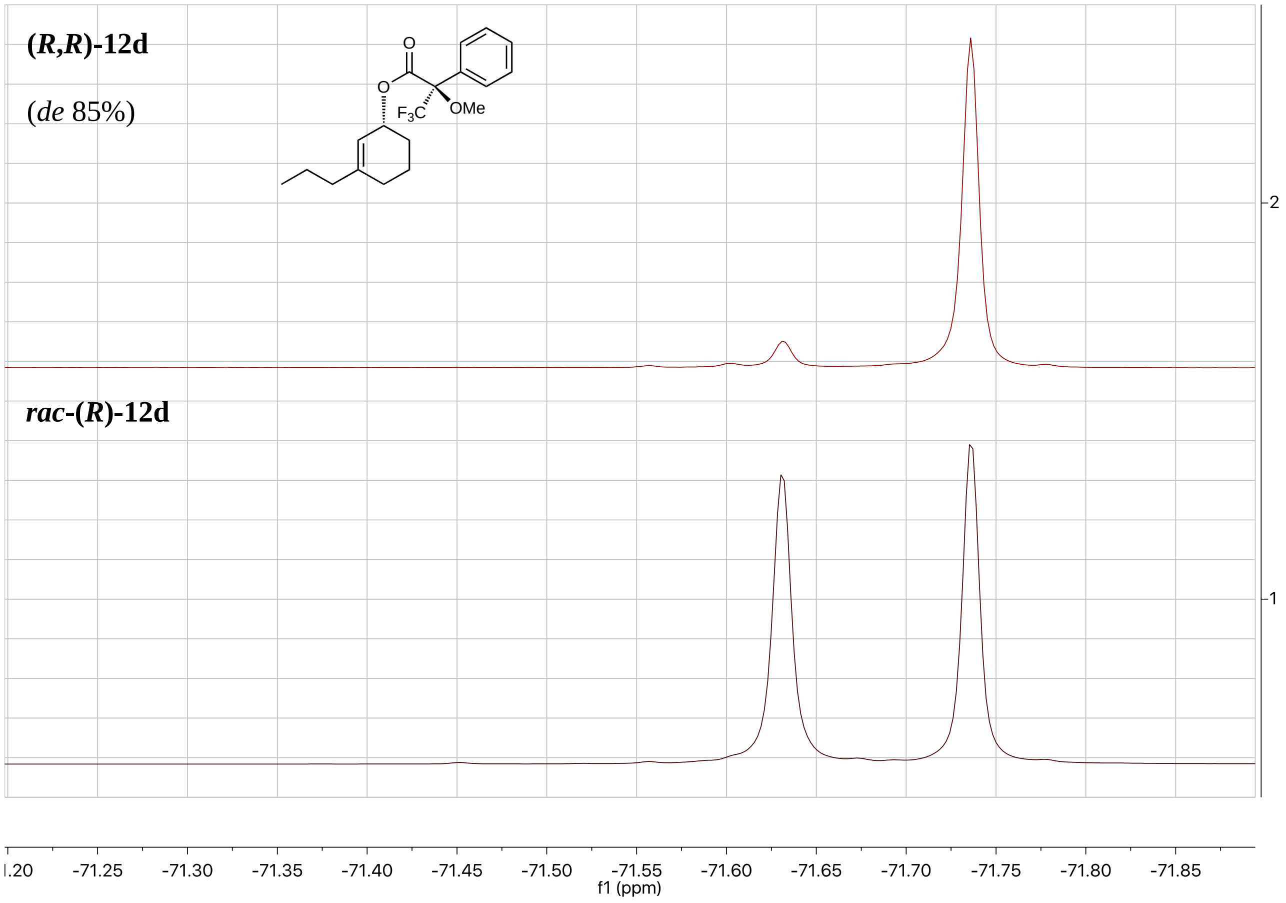
(R,R)-12d
(de 85%)
O
O
F_3C
OMe
rac-(R)-12d
2
1
-71.20
-71.25
-71.30
-71.35
-71.40
-71.45
-71.50
-71.55
-71.60
-71.65
-71.70
-71.75
-71.80
-71.85
f1 (ppm)

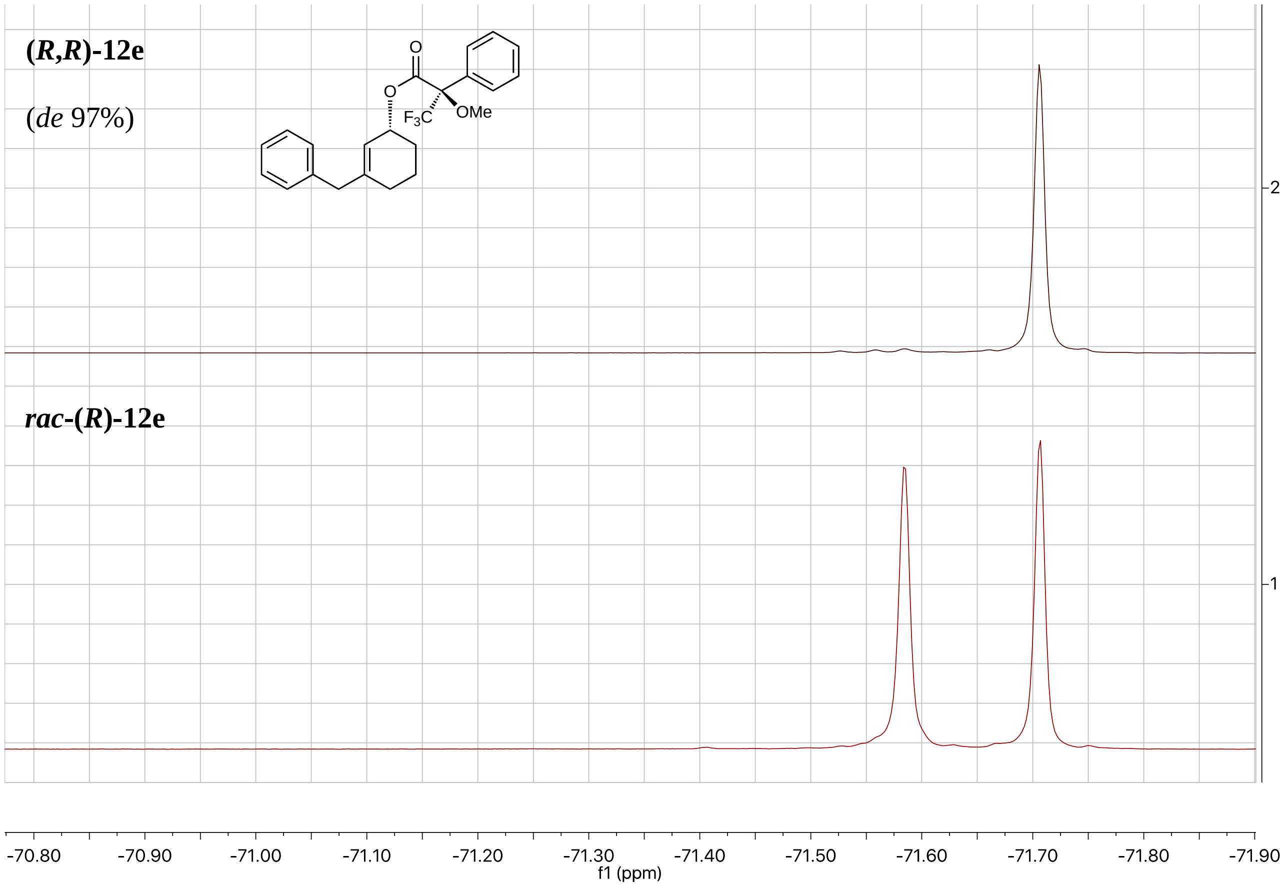
(R,R)-12e
(de 97%)
O
O
F_3C
OMe
rac-(R)-12e
2
1
-70.80
-70.90
-71.00
-71.10
-71.20
-71.30
-71.40
-71.50
-71.60
-71.70
-71.80
-71.90
f1 (ppm)

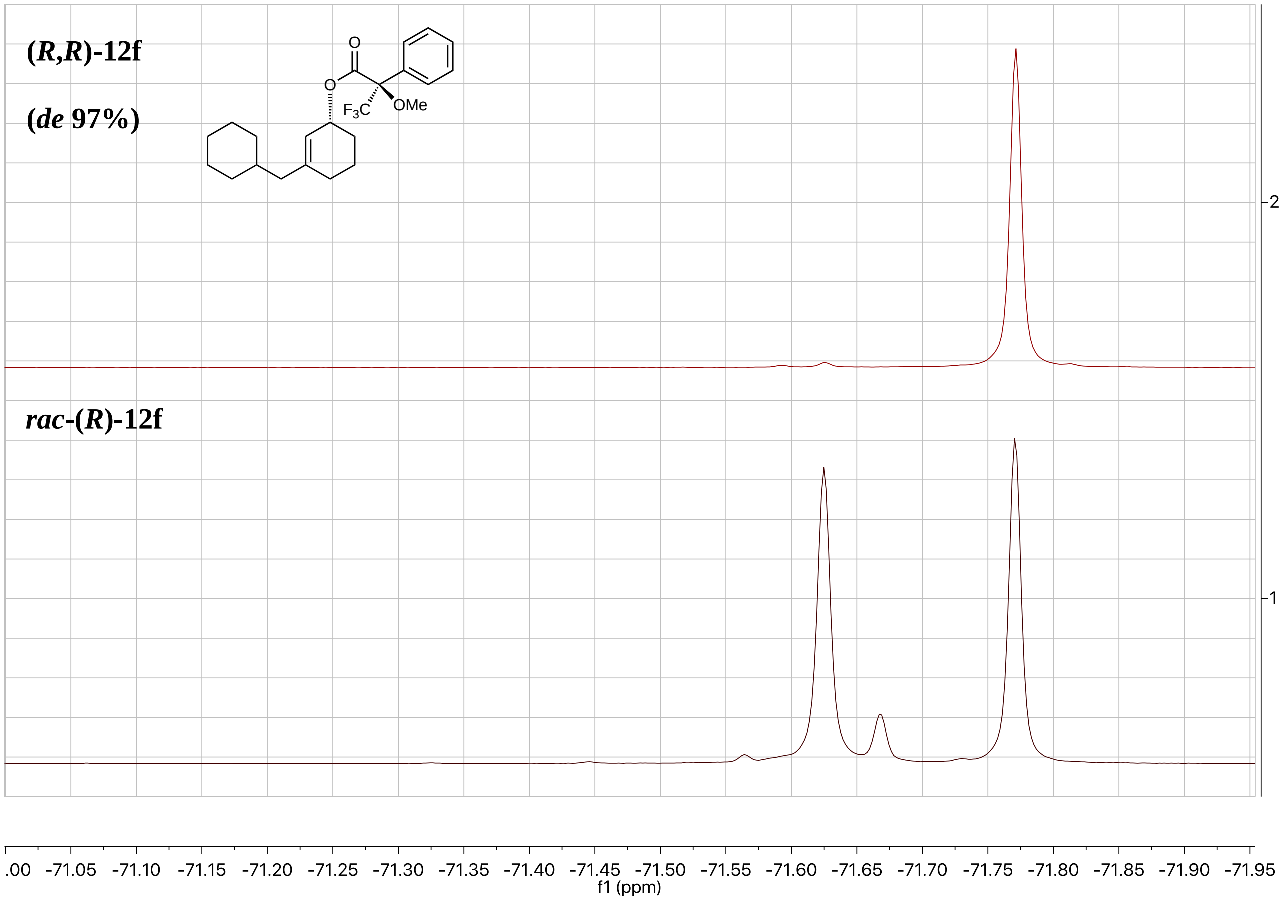
(R,R)-12f
(de 97%)
O
O
F3C
OMe
rac-(R)-12f
2
1
.00 -71.05 -71.10 -71.15 -71.20 -71.25 -71.30 -71.35 -71.40 -71.45 -71.50 -71.55 -71.60 -71.65 -71.70 -71.75 -71.80 -71.85 -71.90 -71.95
f1 (ppm)

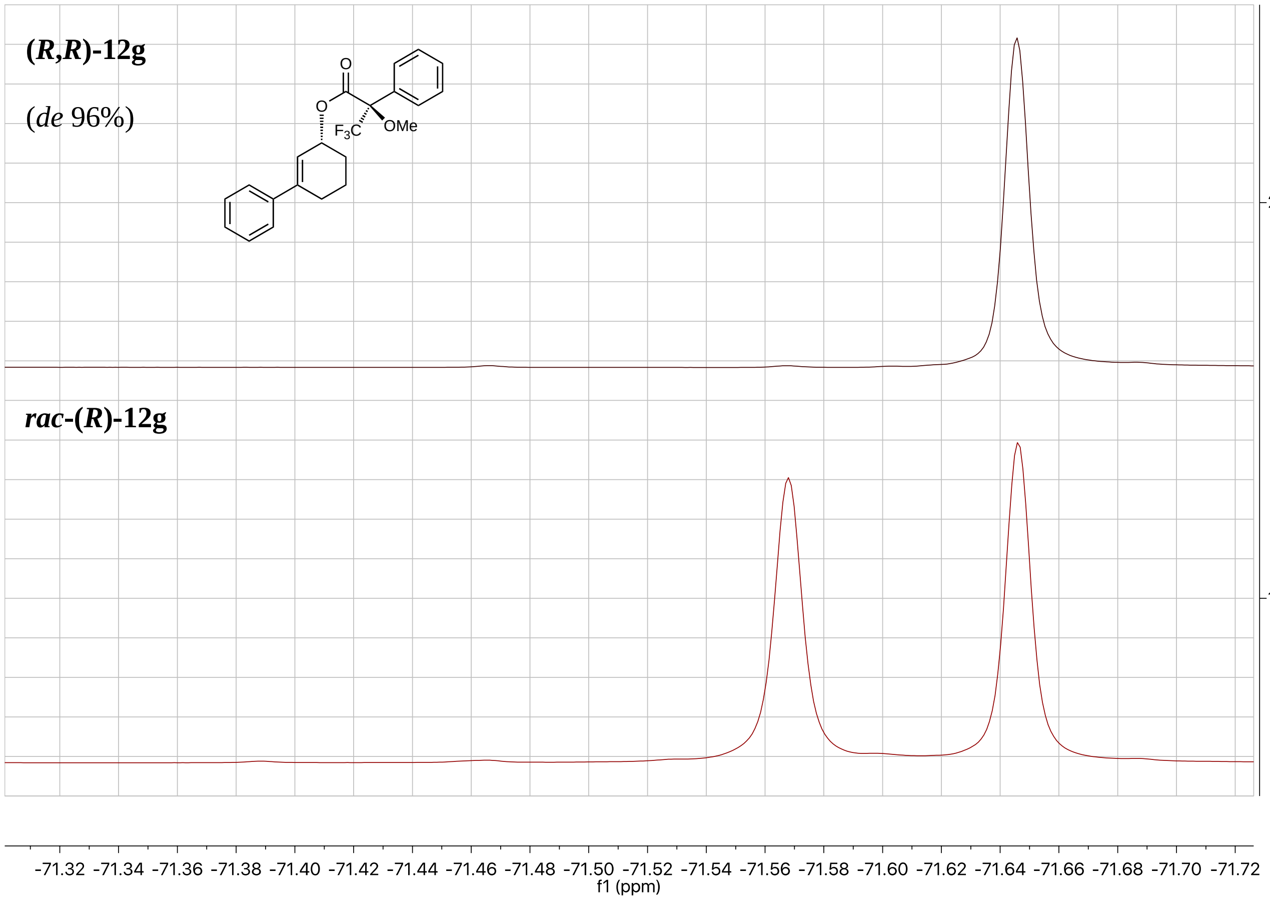
(R,R)-12g
(de 96%)
O
O
F3C
OMe
rac-(R)-12g
2
1
-71.32 -71.34 -71.36 -71.38 -71.40 -71.42 -71.44 -71.46 -71.48 -71.50 -71.52 -71.54 -71.56 -71.58 -71.60 -71.62 -71.64 -71.66 -71.68 -71.70 -71.72
f1 (ppm)

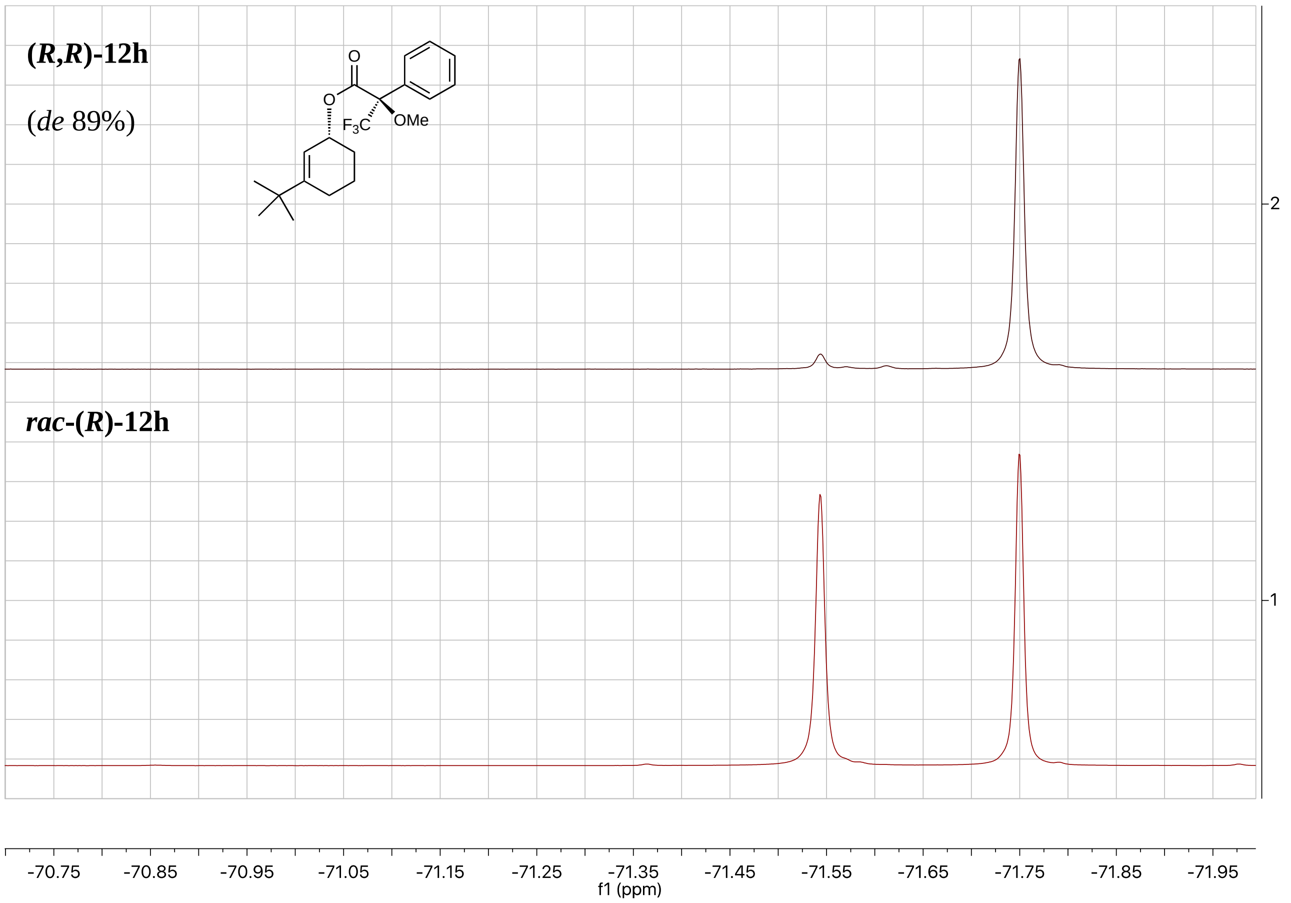
(R,R)-12h
(de 89%)
O
O
F3C
OMe
rac-(R)-12h
2
1
-70.75 -70.85 -70.95 -71.05 -71.15 -71.25 -71.35 -71.45 -71.55 -71.65 -71.75 -71.85 -71.95
f1 (ppm)

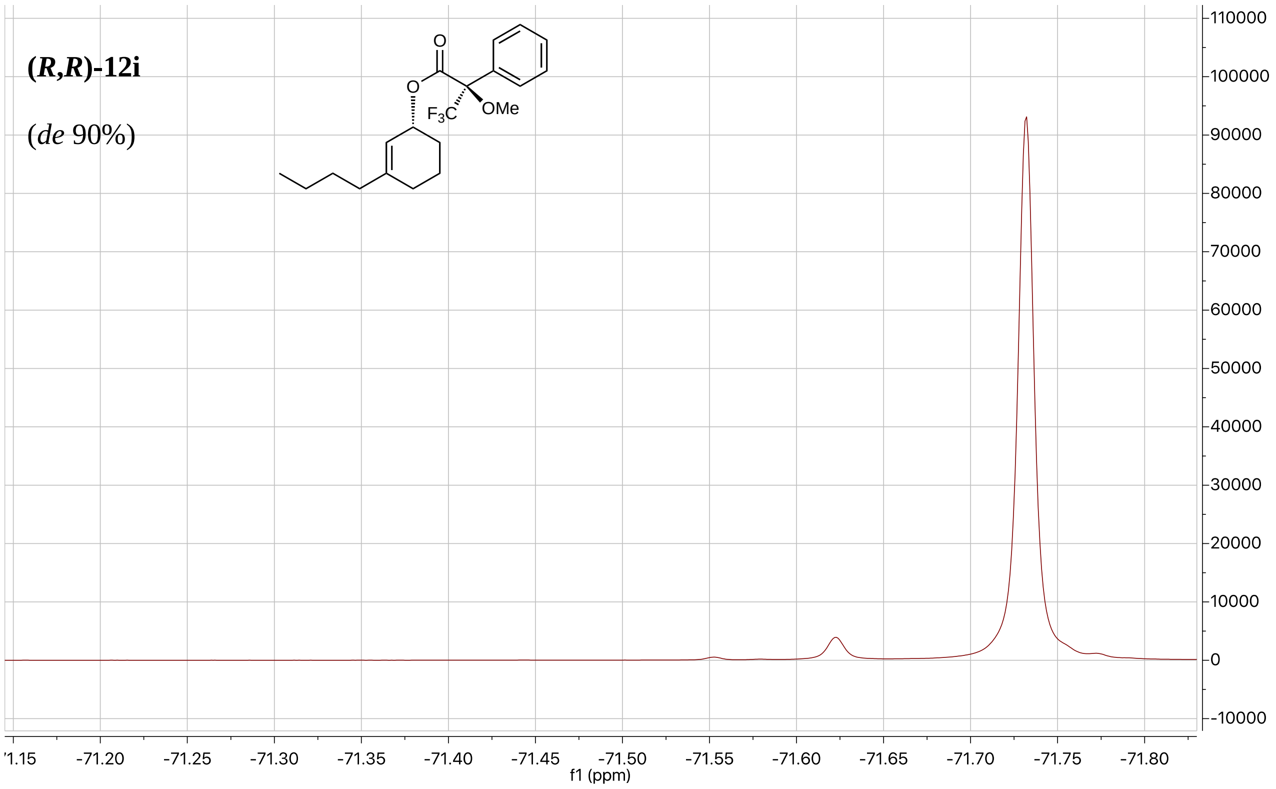
(R,R)-12i
(de 90%)
O
O
F3C
OMe
110000
100000
90000
80000
70000
60000
50000
40000
30000
20000
10000
0
-10000
'1.15 -71.20 -71.25 -71.30 -71.35 -71.40 -71.45 -71.50 -71.55 -71.60 -71.65 -71.70 -71.75 -71.80
f1 (ppm)

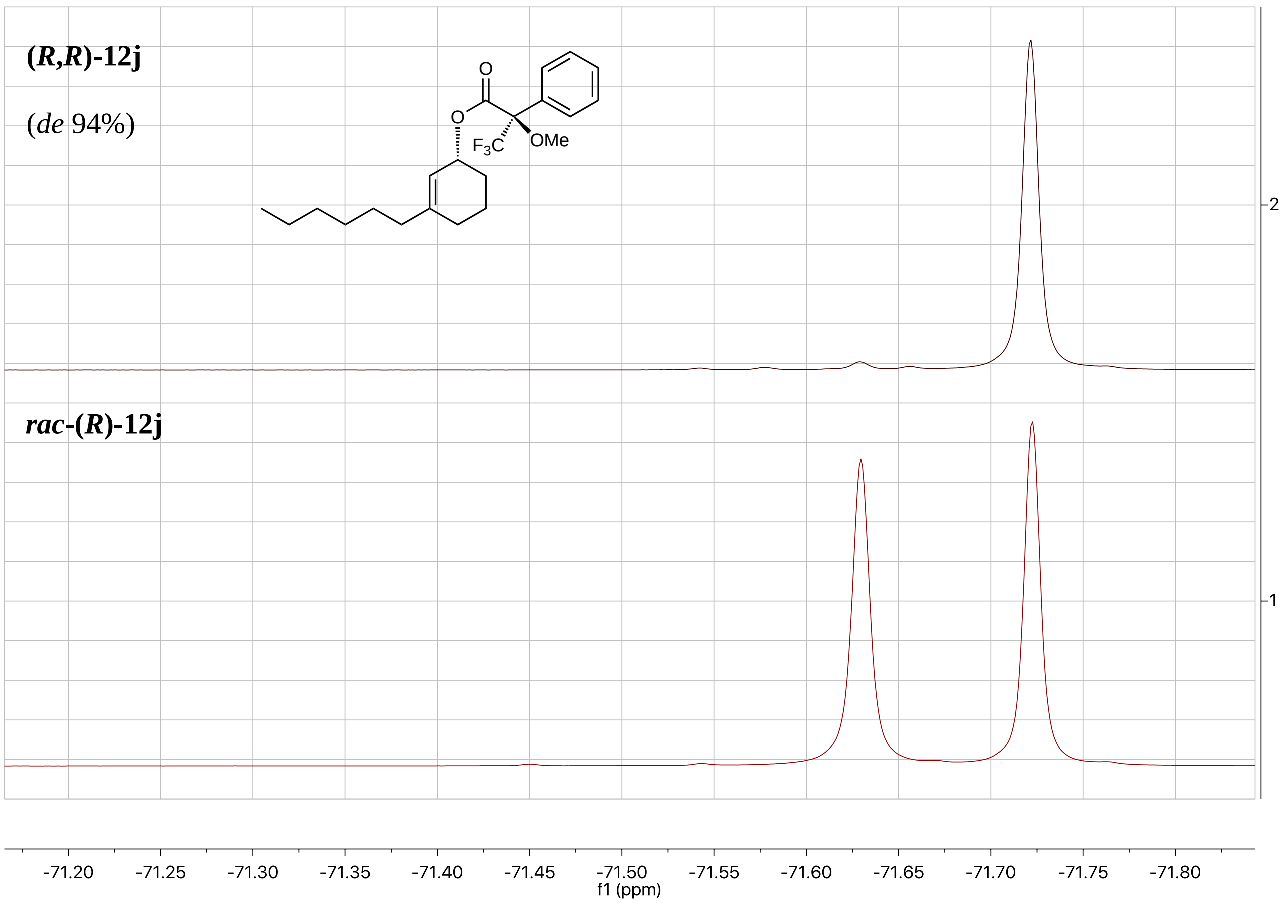
(R,R)-12j
(de 94%)
O
O
F3C
OMe
rac-(R)-12j
2
1
-71.20
-71.25
-71.30
-71.35
-71.40
-71.45
-71.50
-71.55
-71.60
-71.65
-71.70
-71.75
-71.80
f1 (ppm)

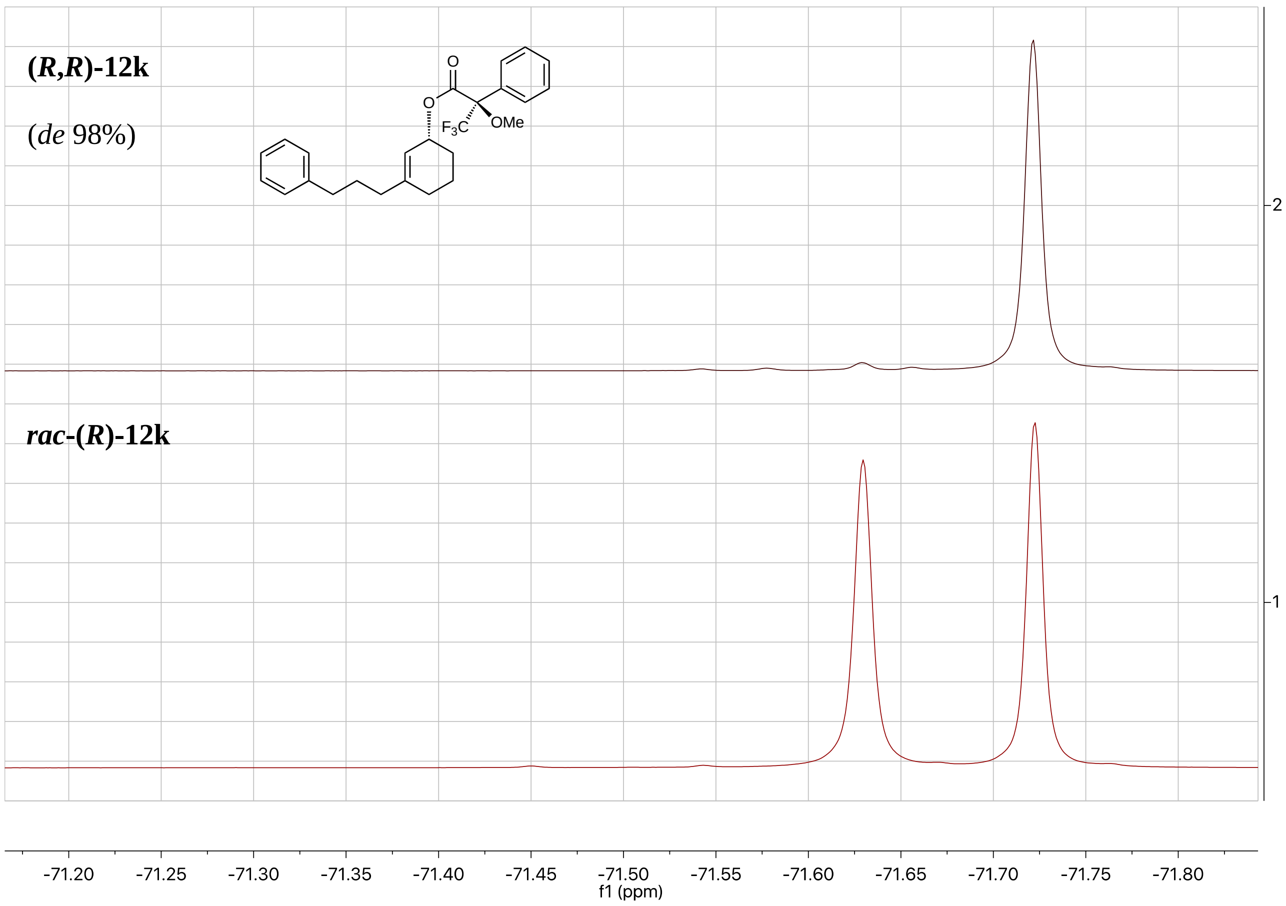
(R,R)-12k
(de 98%)
O
O
F3C
OMe
rac-(R)-12k
2
1
-71.20
-71.25
-71.30
-71.35
-71.40
-71.45
-71.50
-71.55
-71.60
-71.65
-71.70
-71.75
-71.80
f1 (ppm)

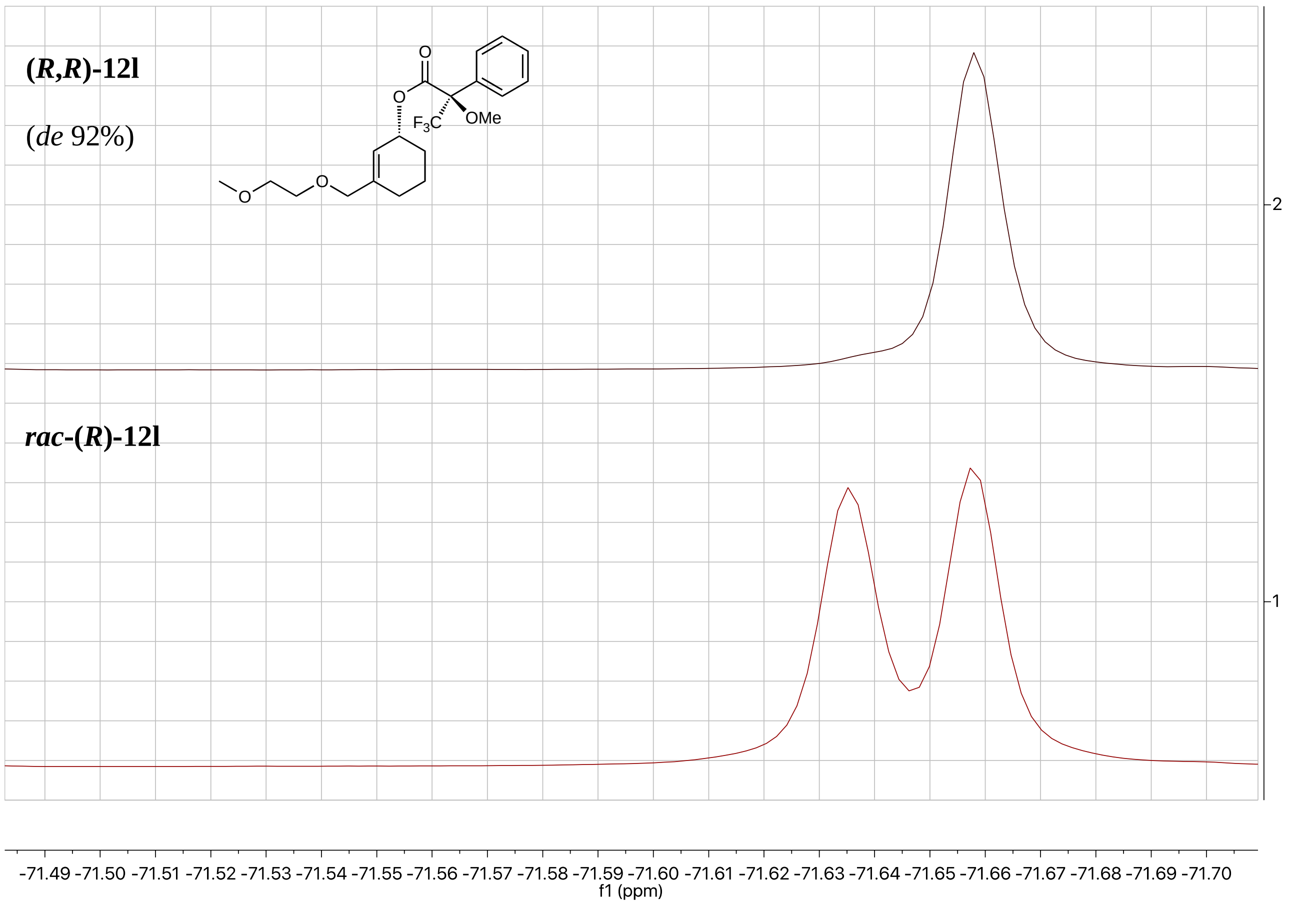
(R,R)-12l
(de 92%)
rac-(R)-12l
O
O
F3C
OMe
O
O
-71.49 -71.50 -71.51 -71.52 -71.53 -71.54 -71.55 -71.56 -71.57 -71.58 -71.59 -71.60 -71.61 -71.62 -71.63 -71.64 -71.65 -71.66 -71.67 -71.68 -71.69 -71.70
f1 (ppm)
2
1

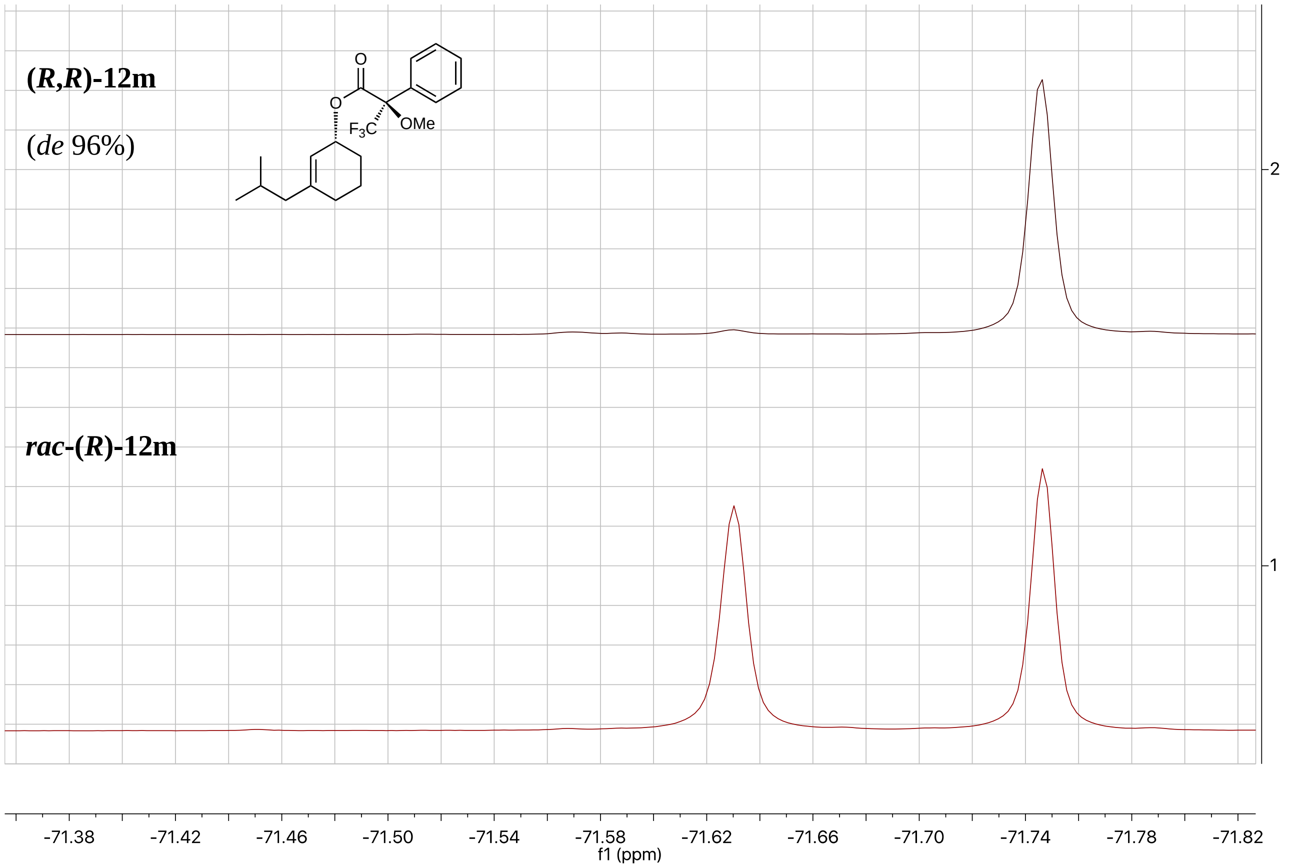
(R,R)-12m
(de 96%)
rac-(R)-12m
O
O
F3C
OMe
-71.38 -71.42 -71.46 -71.50 -71.54 -71.58 -71.62 -71.66 -71.70 -71.74 -71.78 -71.82
f1 (ppm)
2
1

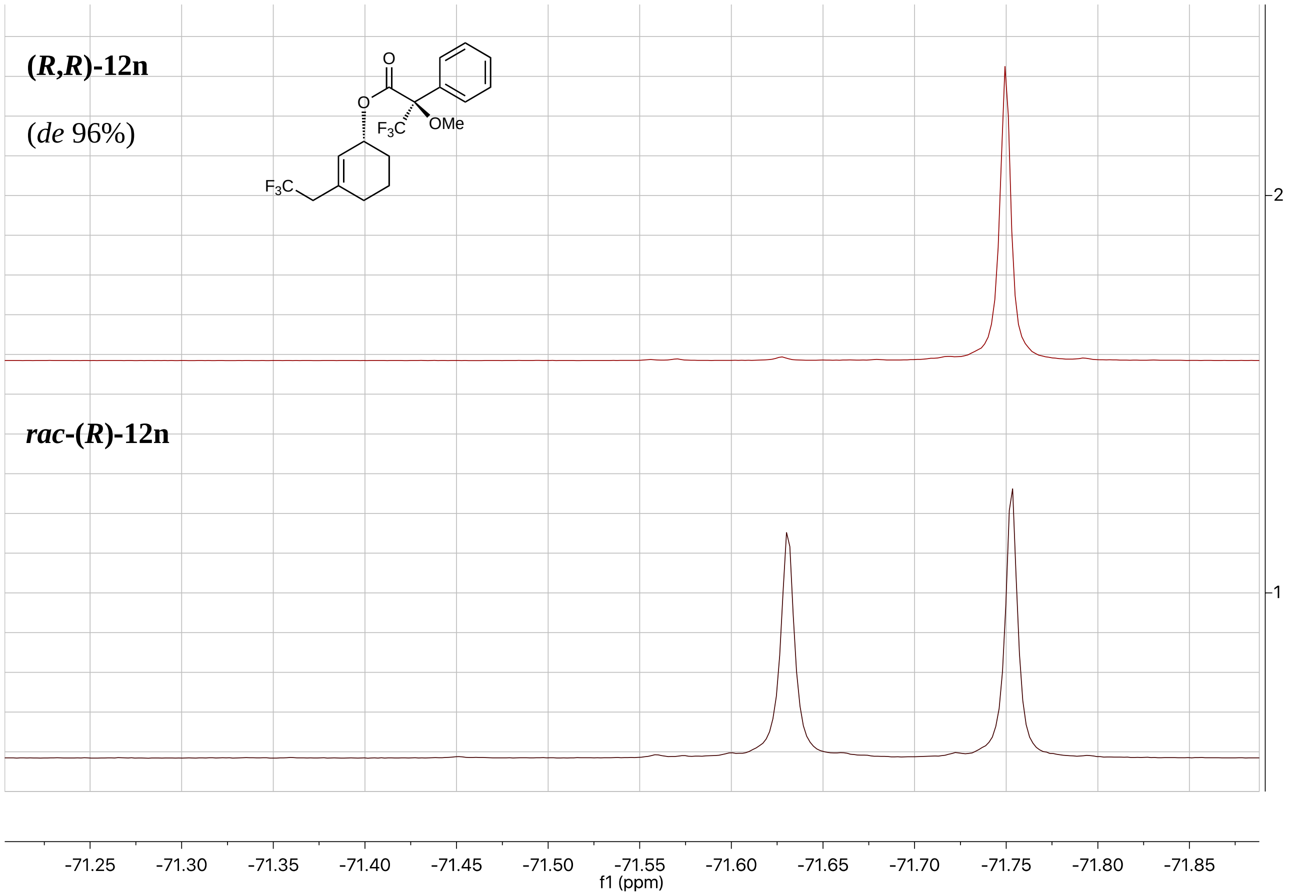
(R,R)-12n
(de 96%)
rac-(R)-12n
O
O
F3C
OMe
F3C
2
1
-71.25
-71.30
-71.35
-71.40
-71.45
-71.50
-71.55
-71.60
-71.65
-71.70
-71.75
-71.80
-71.85
f1 (ppm)

5. Determination of chemical shift of H-C5^{Fuc} in case of superposition with the DHO peak

^{1}H NMR of **2k**: H-C5^{Fuc} = 4.75 ppm; separated from DHO peak

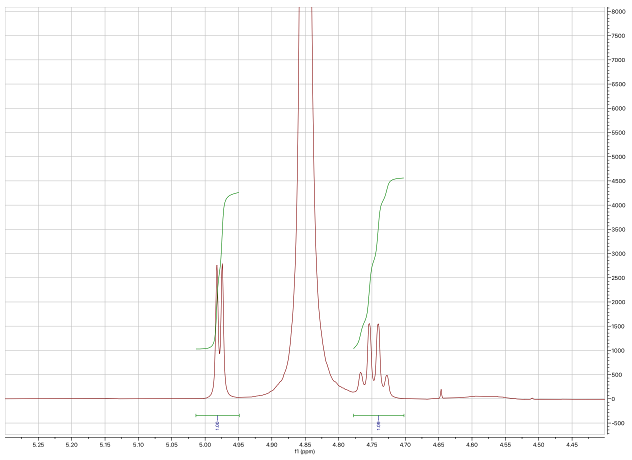

^{1}H NMR of **2m:** H-C5^{Fuc} = 4.84 ppm; superposition with DHO

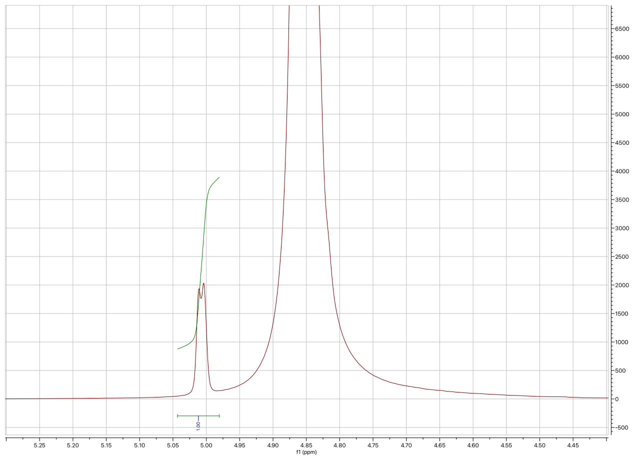

Then, the chemical shift of H-C5^{Fuc} is obtained from COSY spectrum

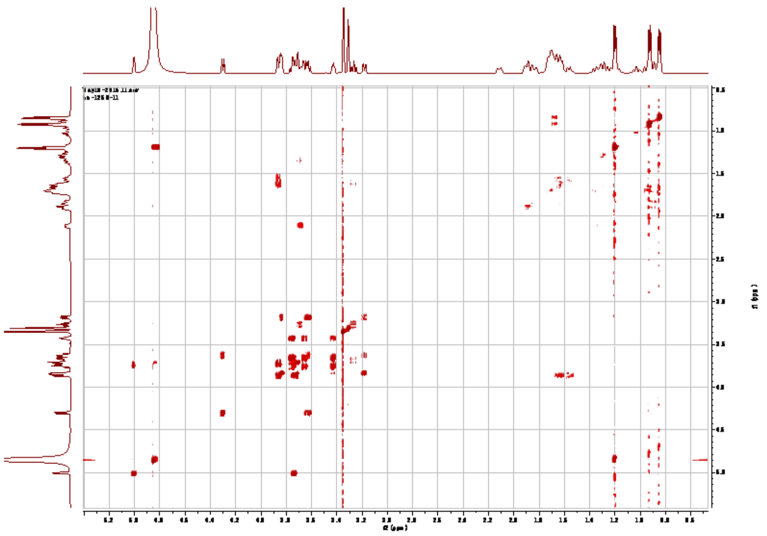

6. ^{1}H and ^{13}C NMR spectra

For spectroscopic data of **2a**, **2b** and **2c** see [8].

Compound 2d

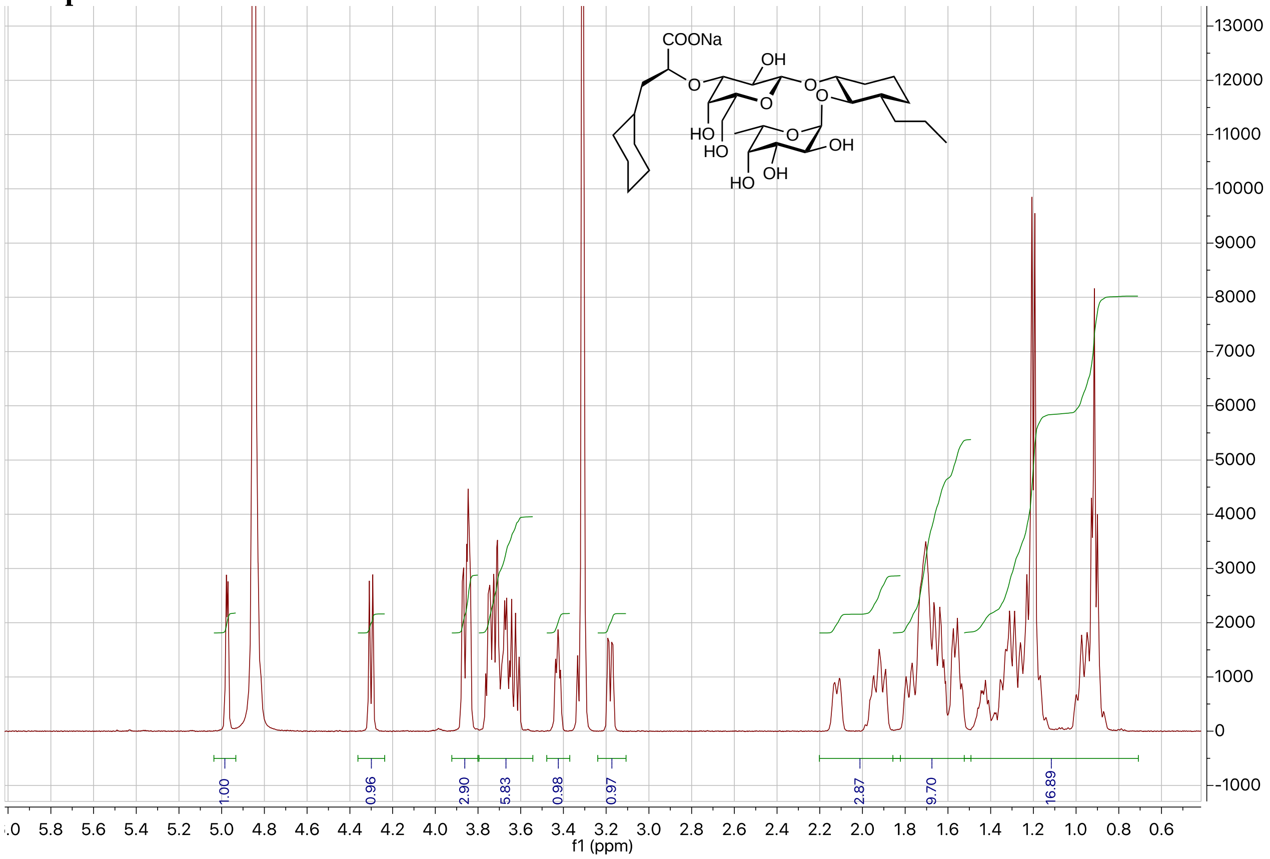

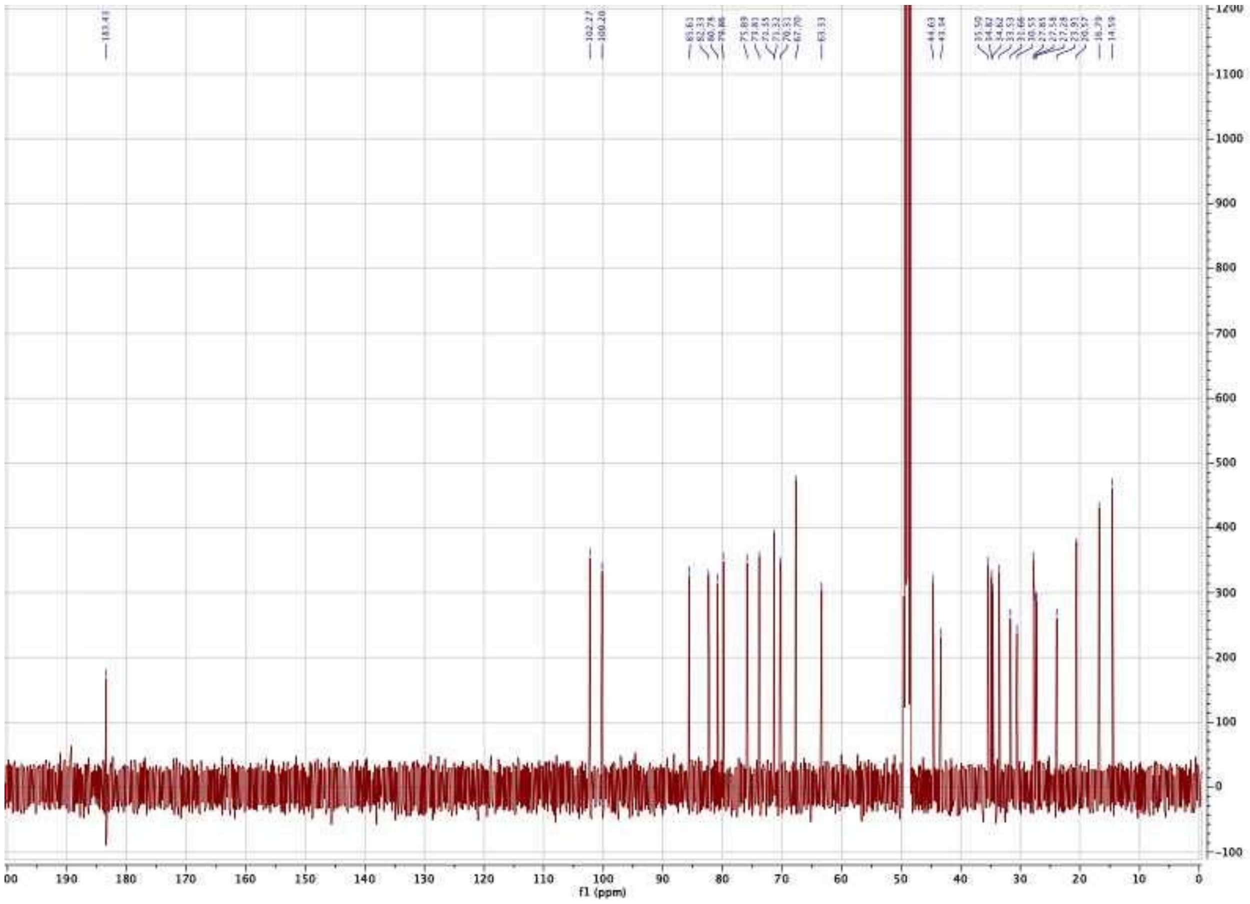

Compound 2e

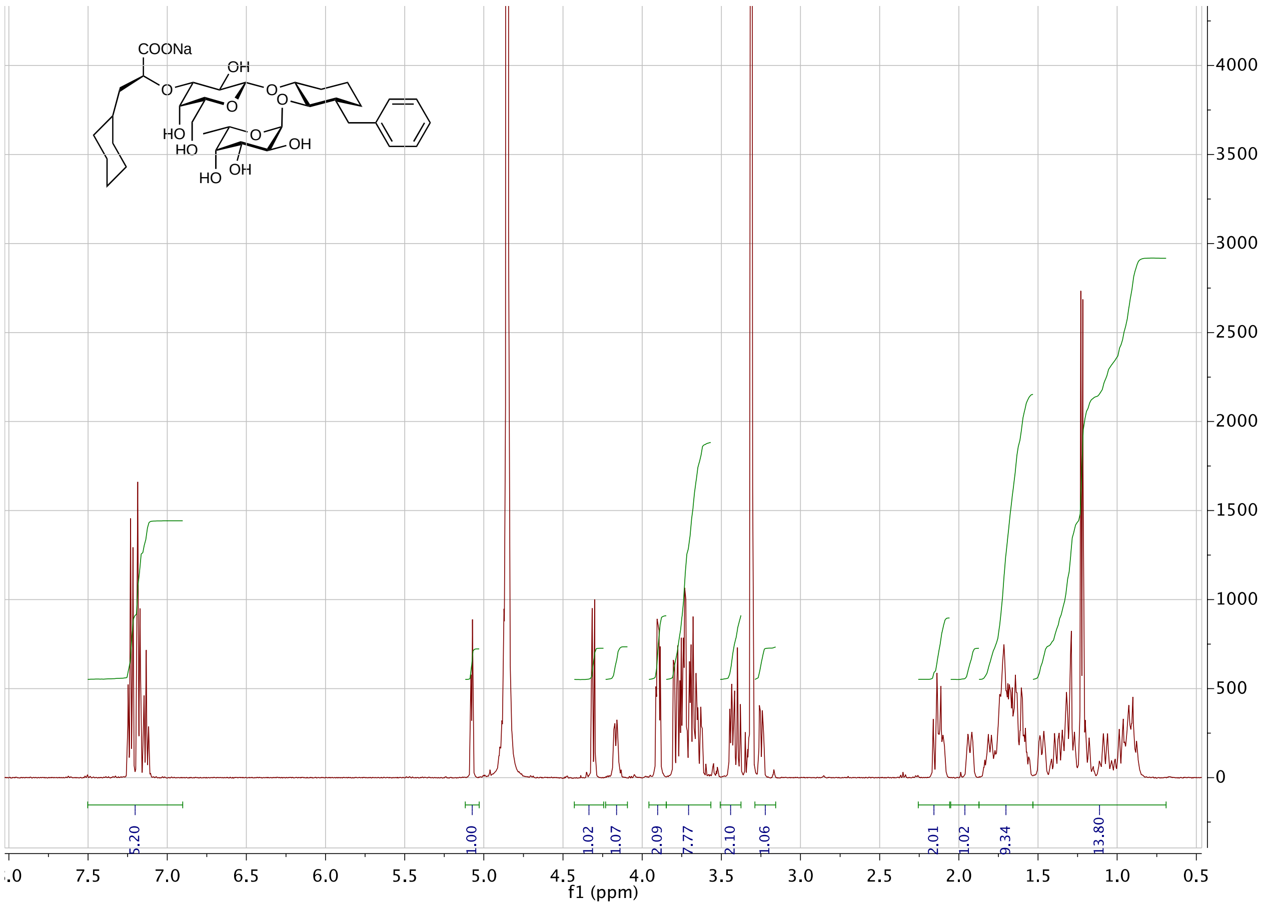
COONa
OH
O
HO
HO
OH
HO
OH
f1 (ppm)
5.20
1.00
1.02
1.07
2.09
7.77
2.10
1.06
2.01
1.02
9.34
13.80

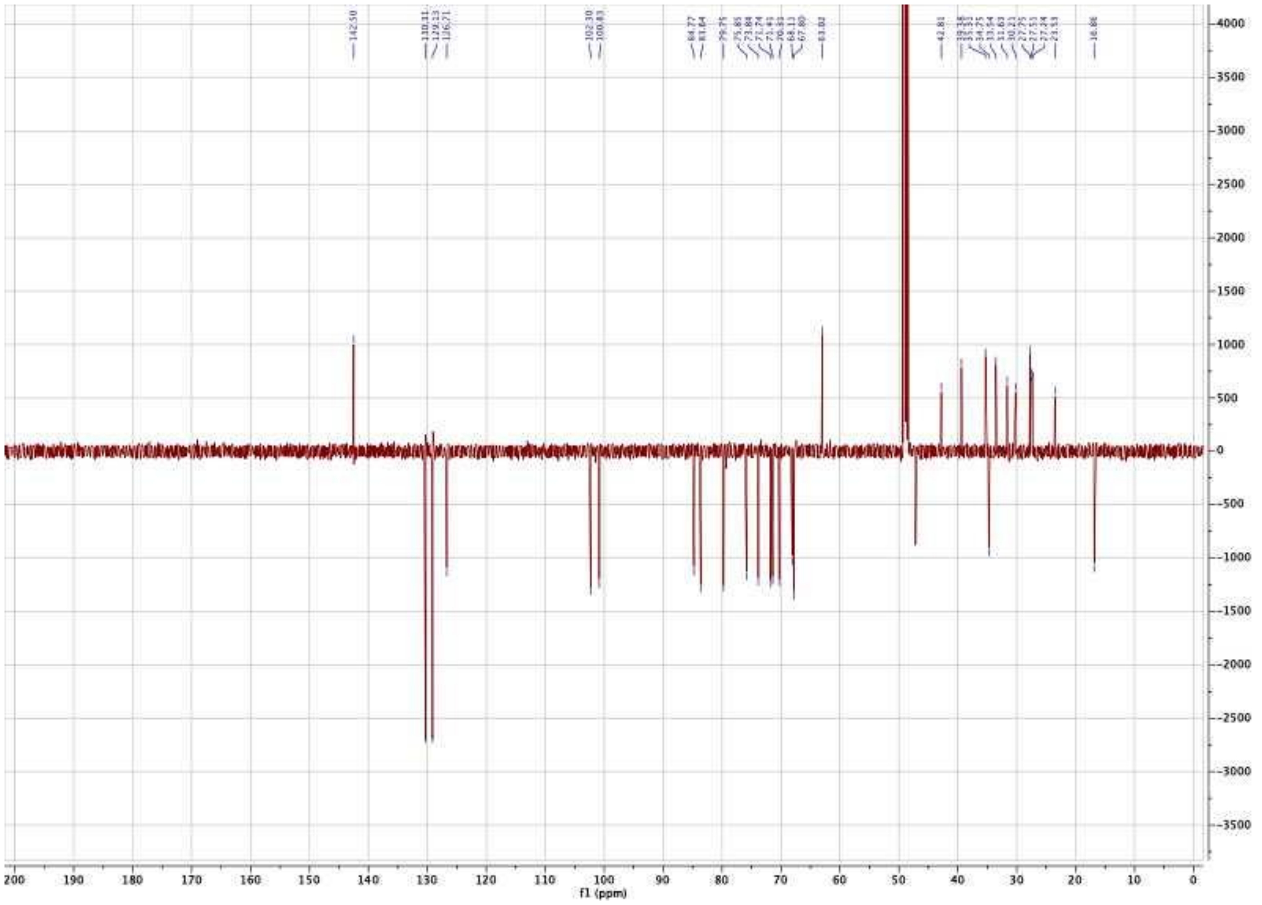
142.50
130.11
129.13
126.71
102.30
100.83
84.77
83.64
79.75
75.85
73.84
71.74
71.41
70.81
68.13
67.80
63.02
42.81
f1 (ppm)

Compound 2f

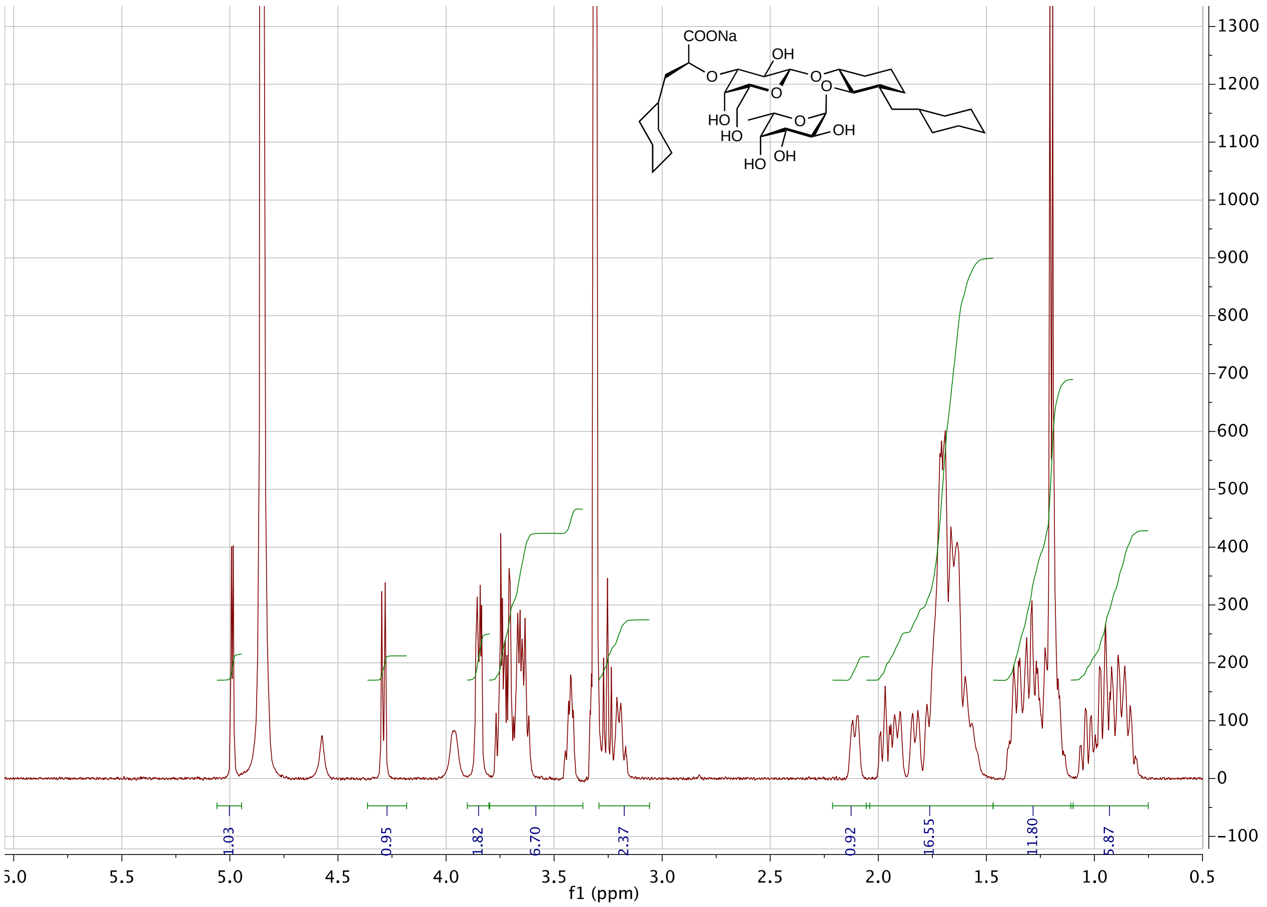

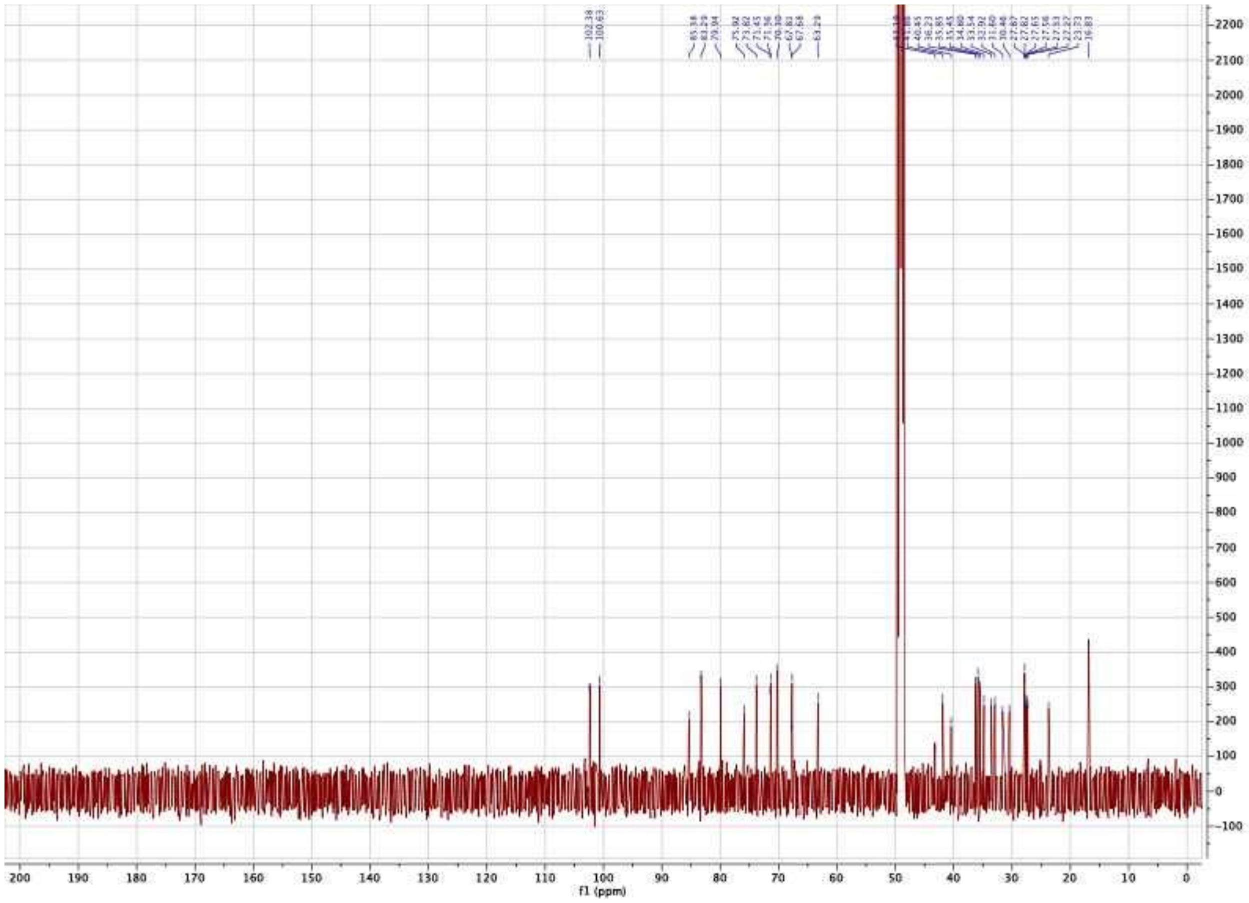

Compound 2g

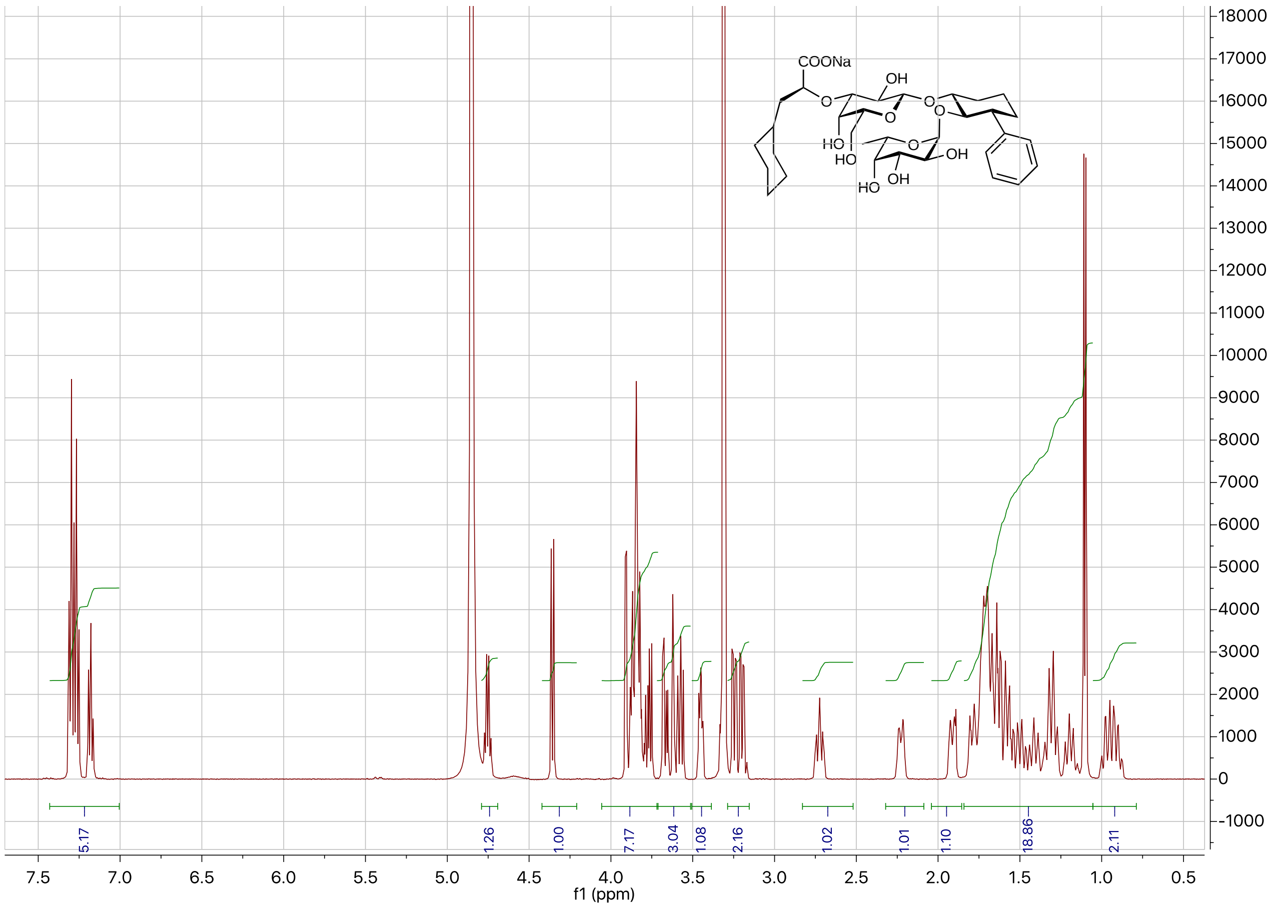
COONa
OH
O
HO
HO
OH
HO
OH
5.17
1.26
1.00
7.17
3.04
1.08
2.16
1.02
1.01
1.10
18.86
2.11
f1 (ppm)

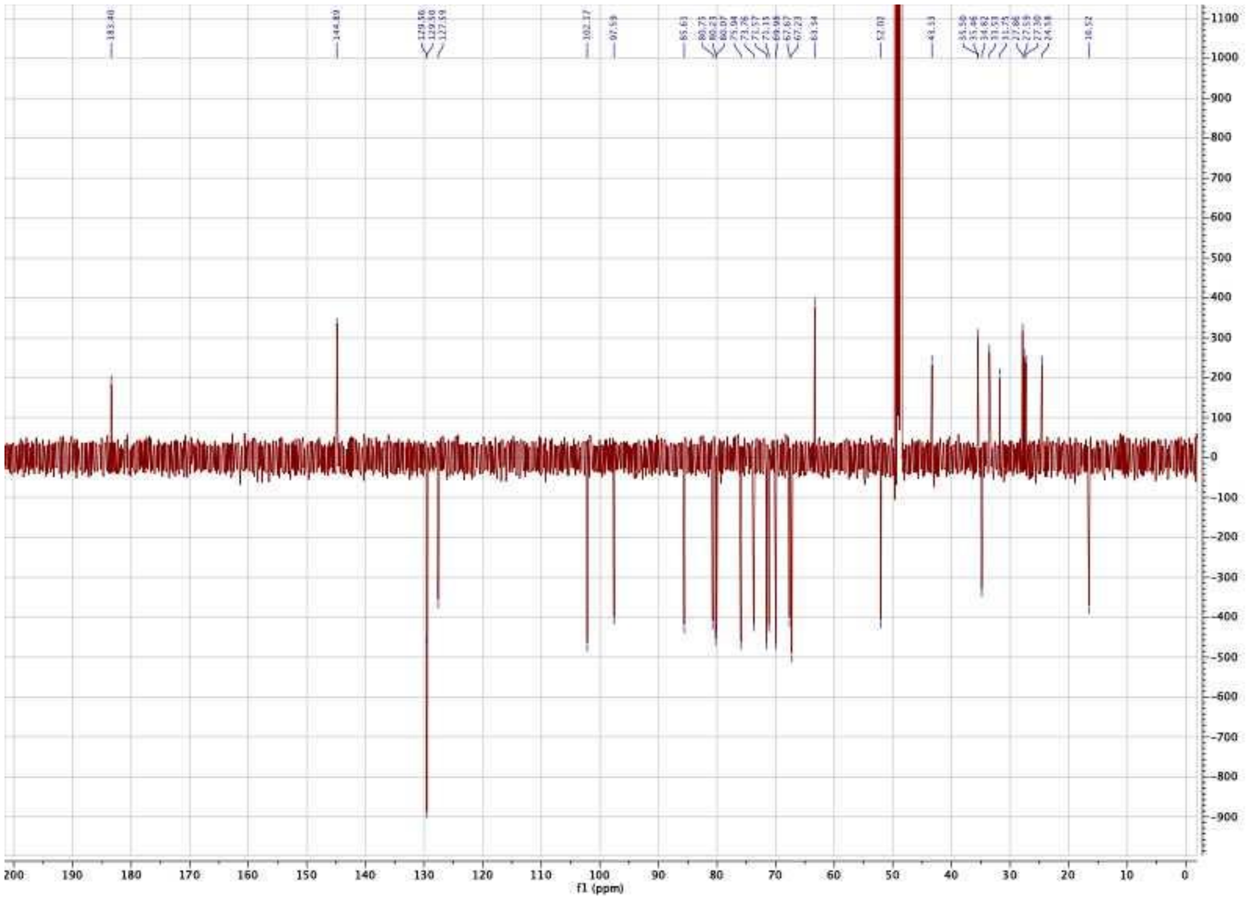
f1 (ppm)

Compound 2h

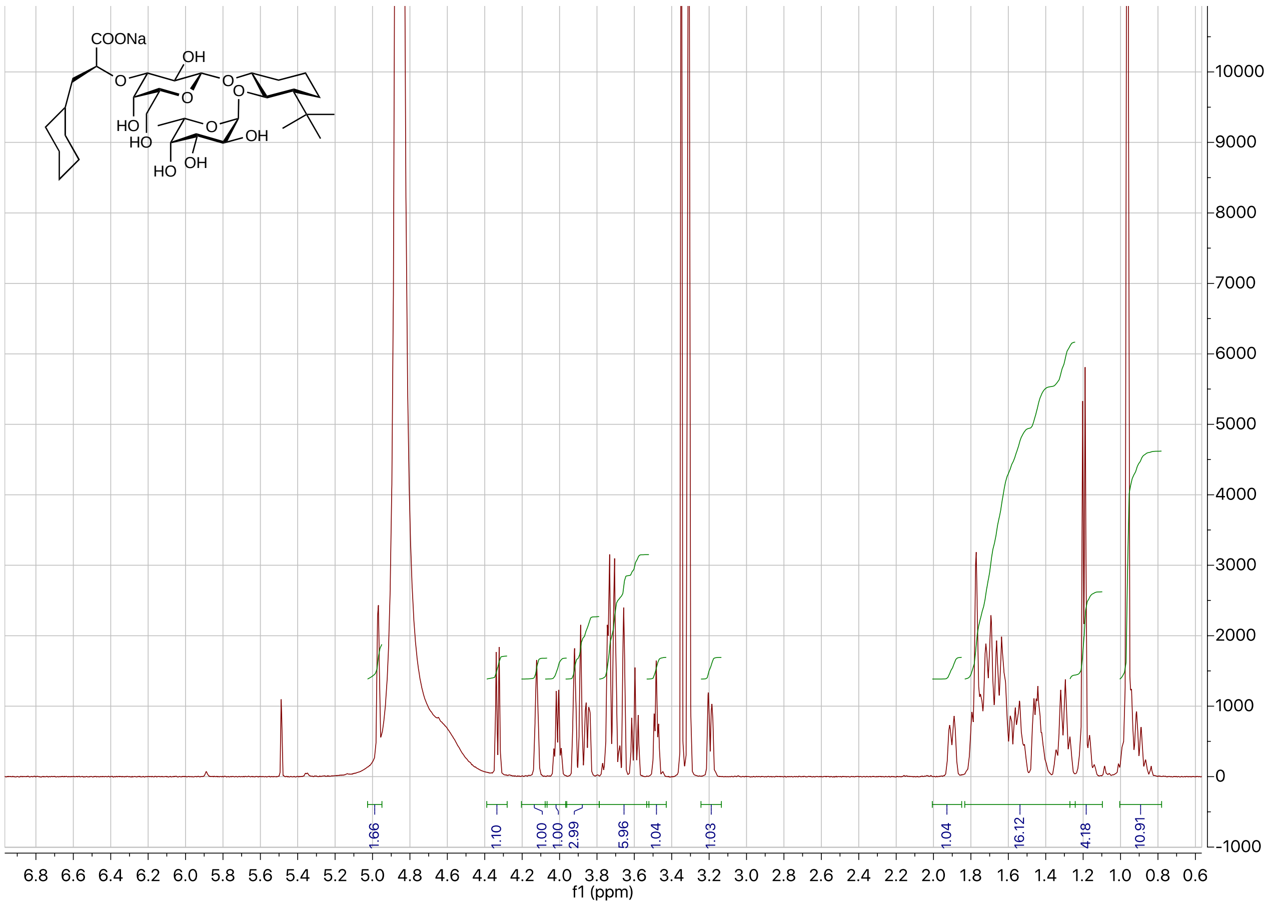

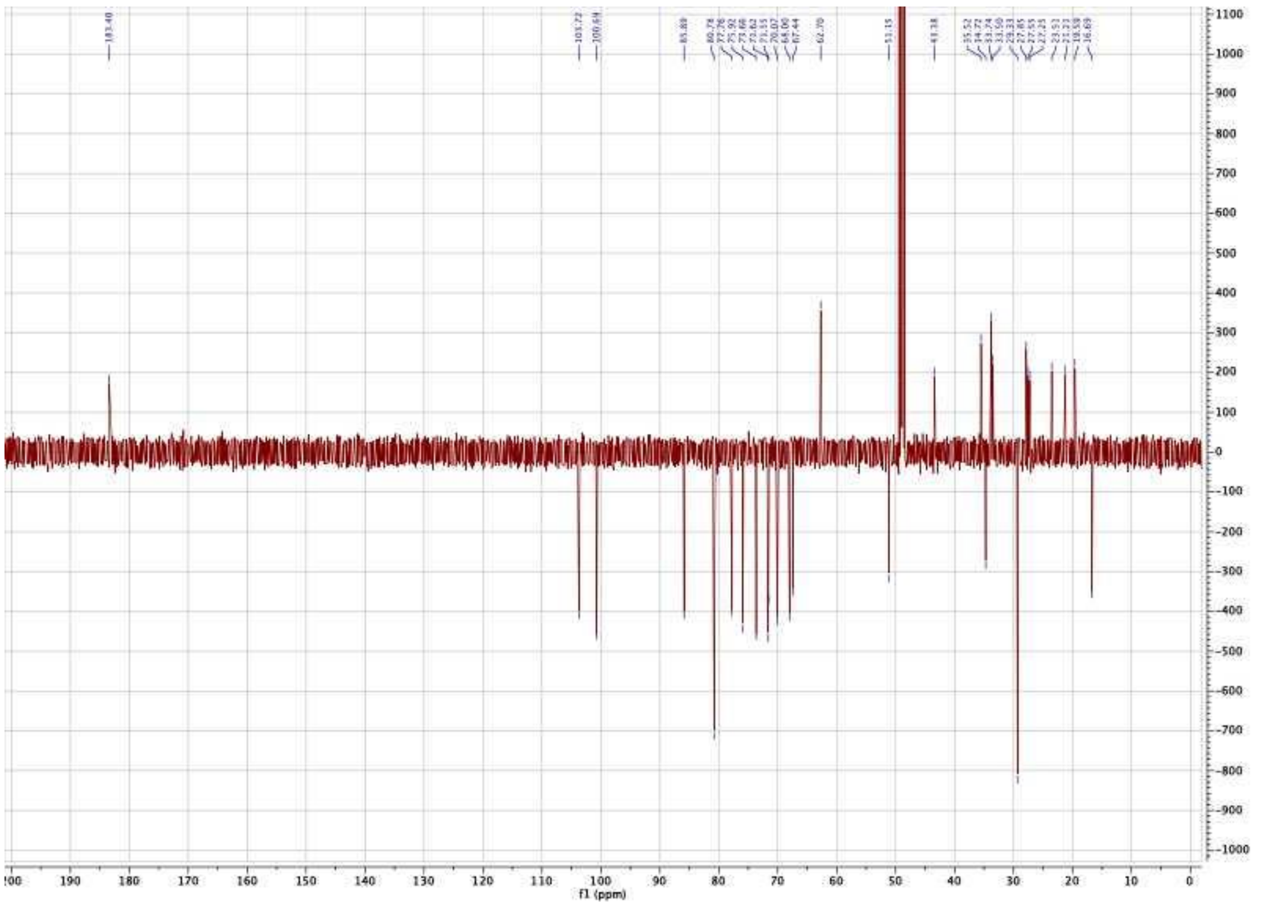

Compound 2i

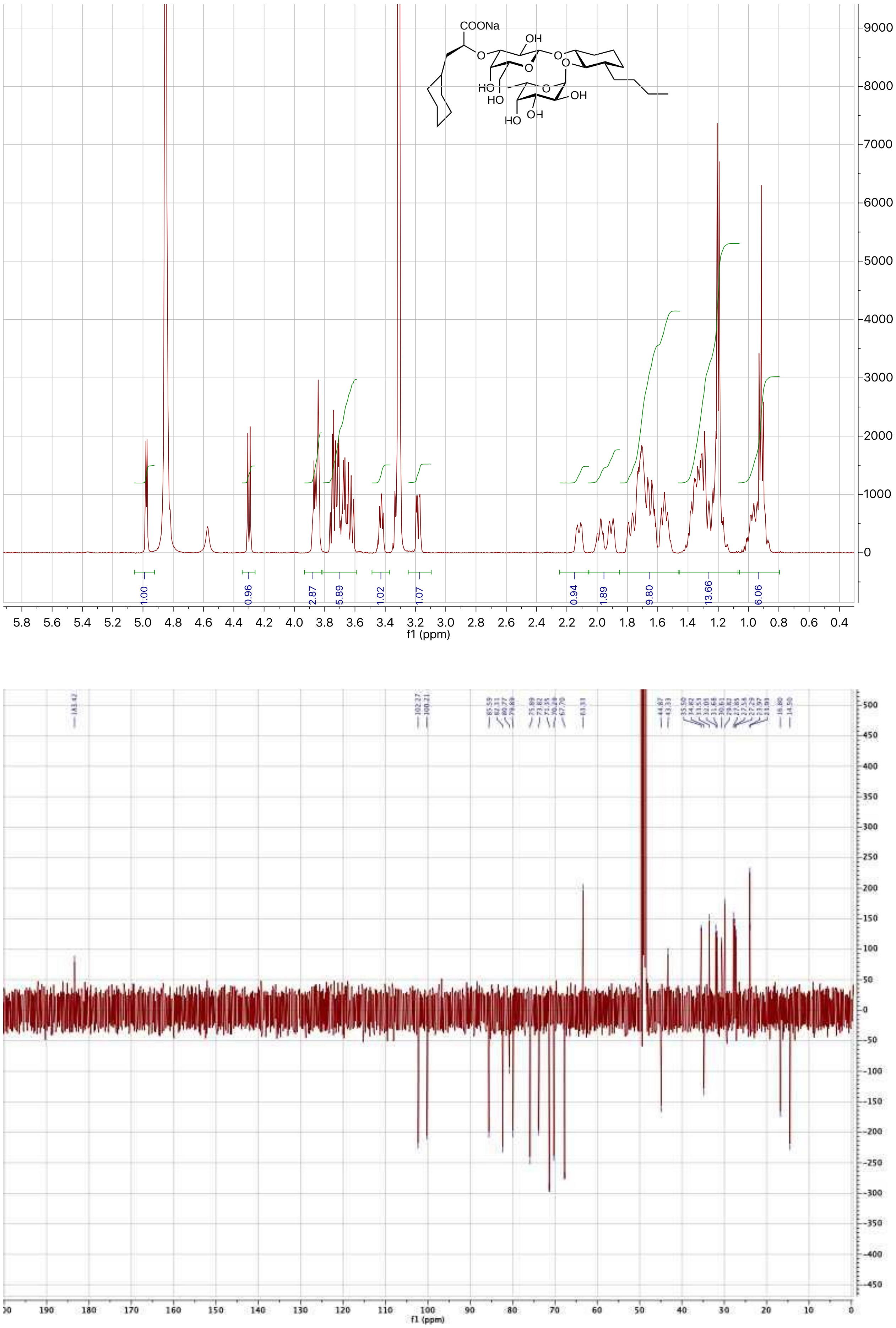
COONa
OH
O
HO
HO
OH
HO
OH
1.00
0.96
2.87
5.89
1.02
1.07
0.94
1.89
9.80
13.66
6.06
f1 (ppm)
183.42
102.27
100.21
f1 (ppm)

Compound 2j

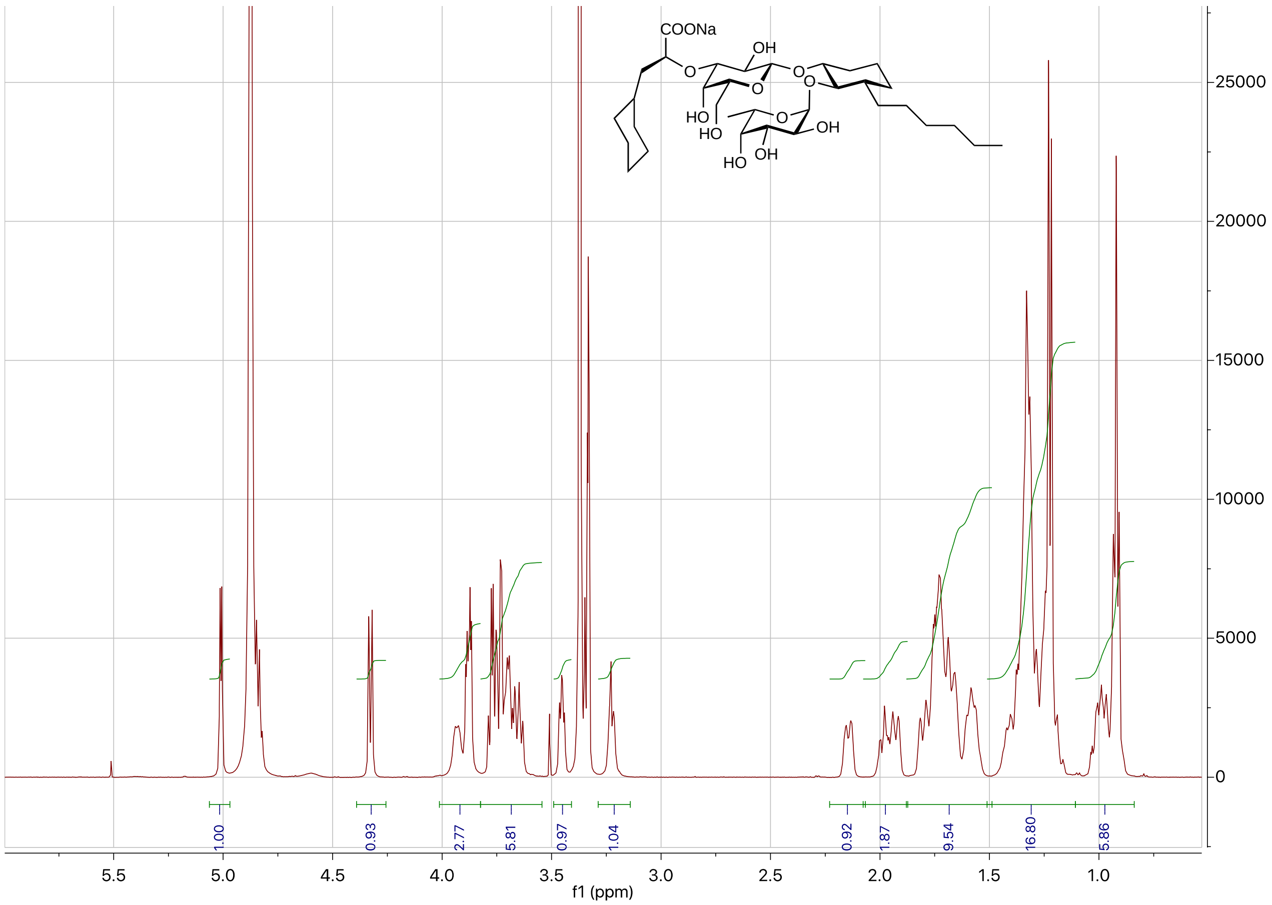
COONa
OH
O
HO
HO
OH
HO
OH
1.00
0.93
2.77
5.81
0.97
1.04
0.92
1.87
9.54
16.80
5.86
f1 (ppm)

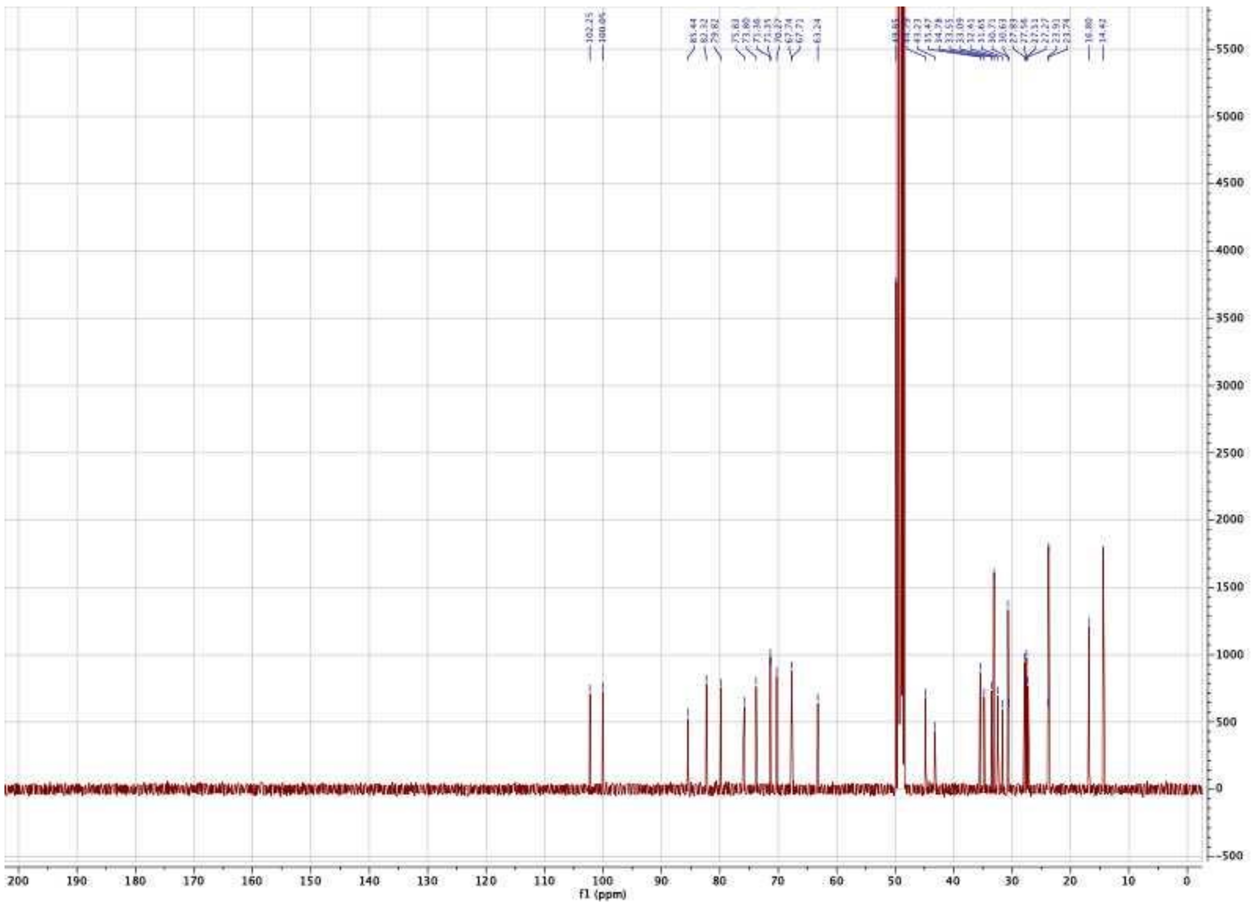
f1 (ppm)

Compound 2k

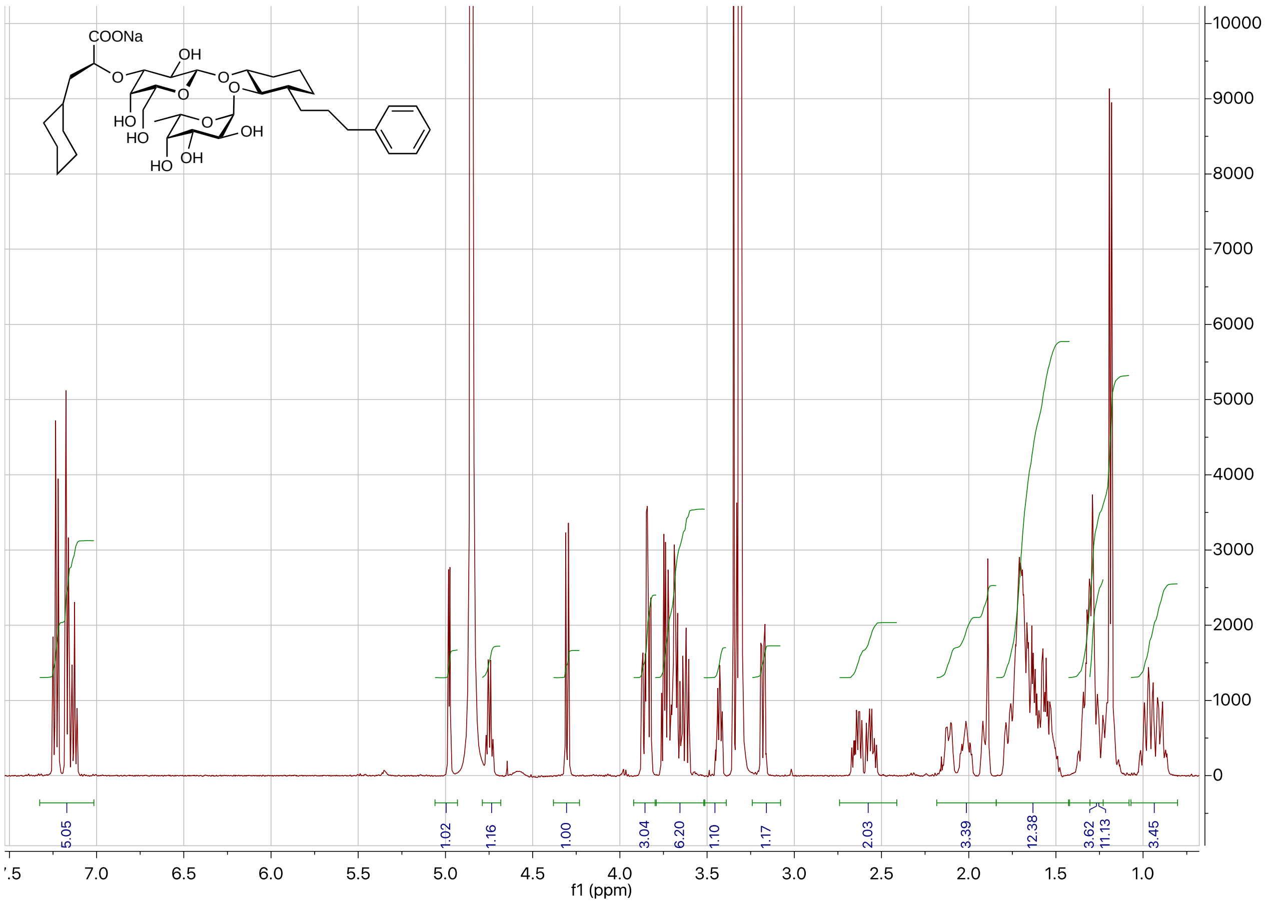

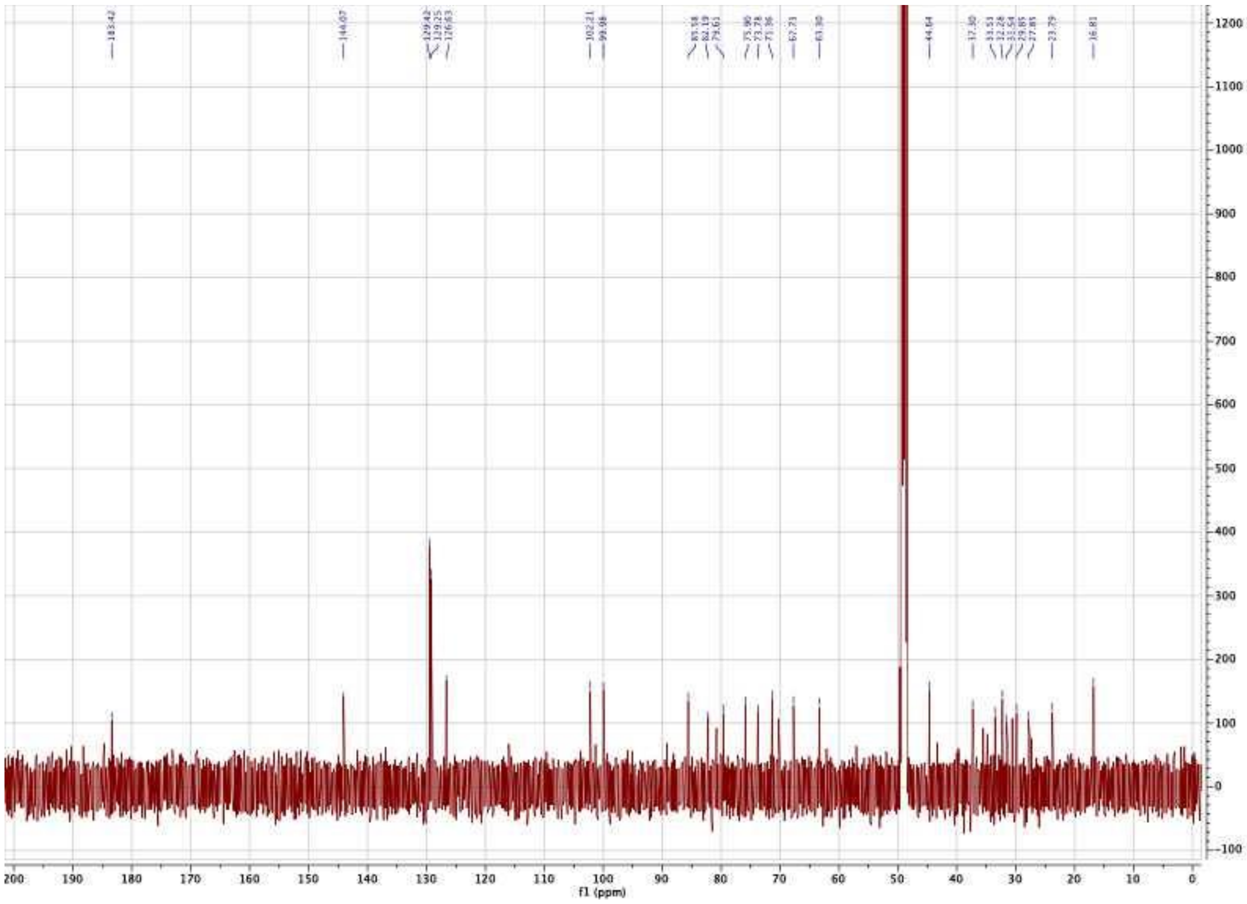

Compound 2l

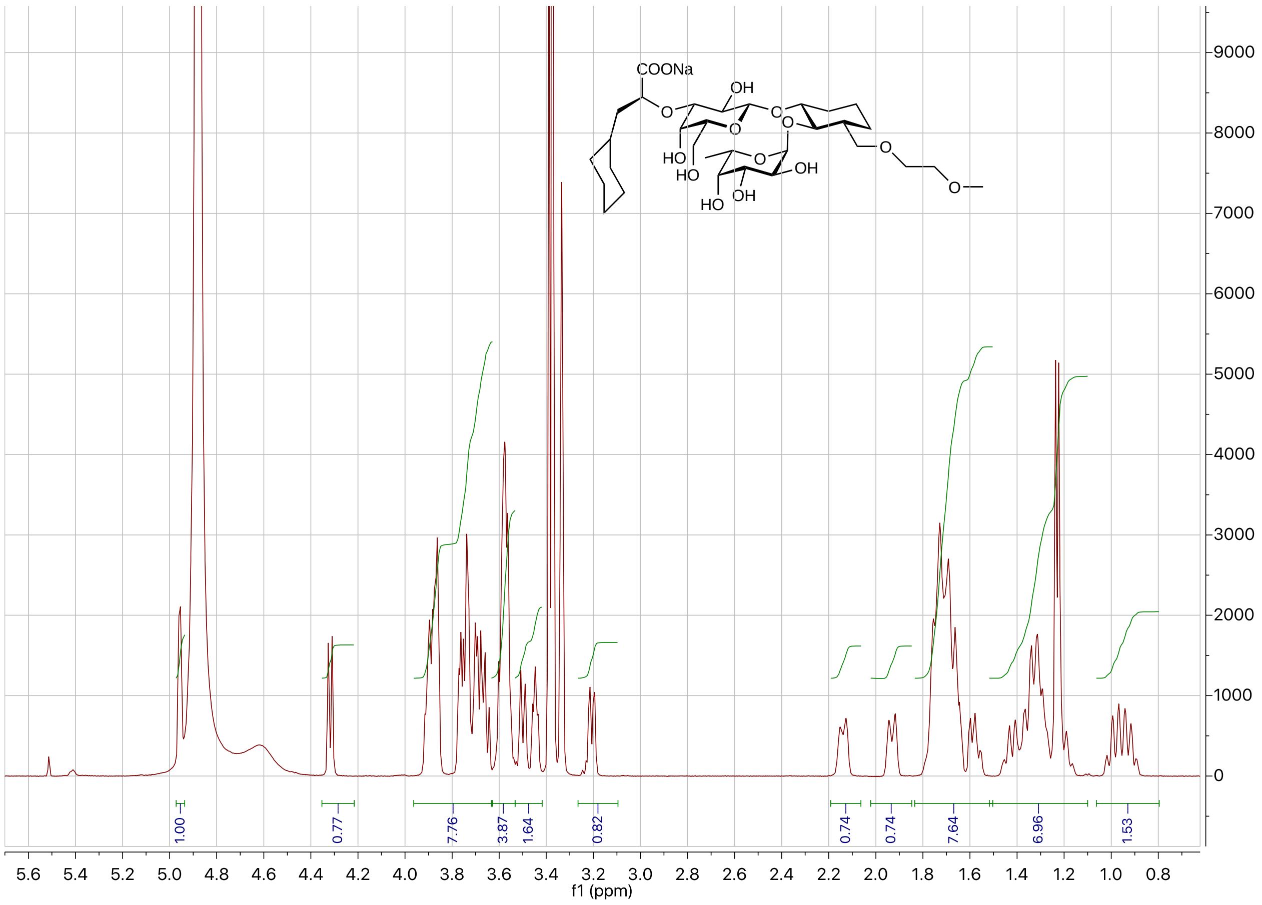
COONa
OH
HO
HO
OH
HO
OH
1.00
0.77
7.76
3.87
1.64
0.82
0.74
0.74
7.64
6.96
1.53
f1 (ppm)

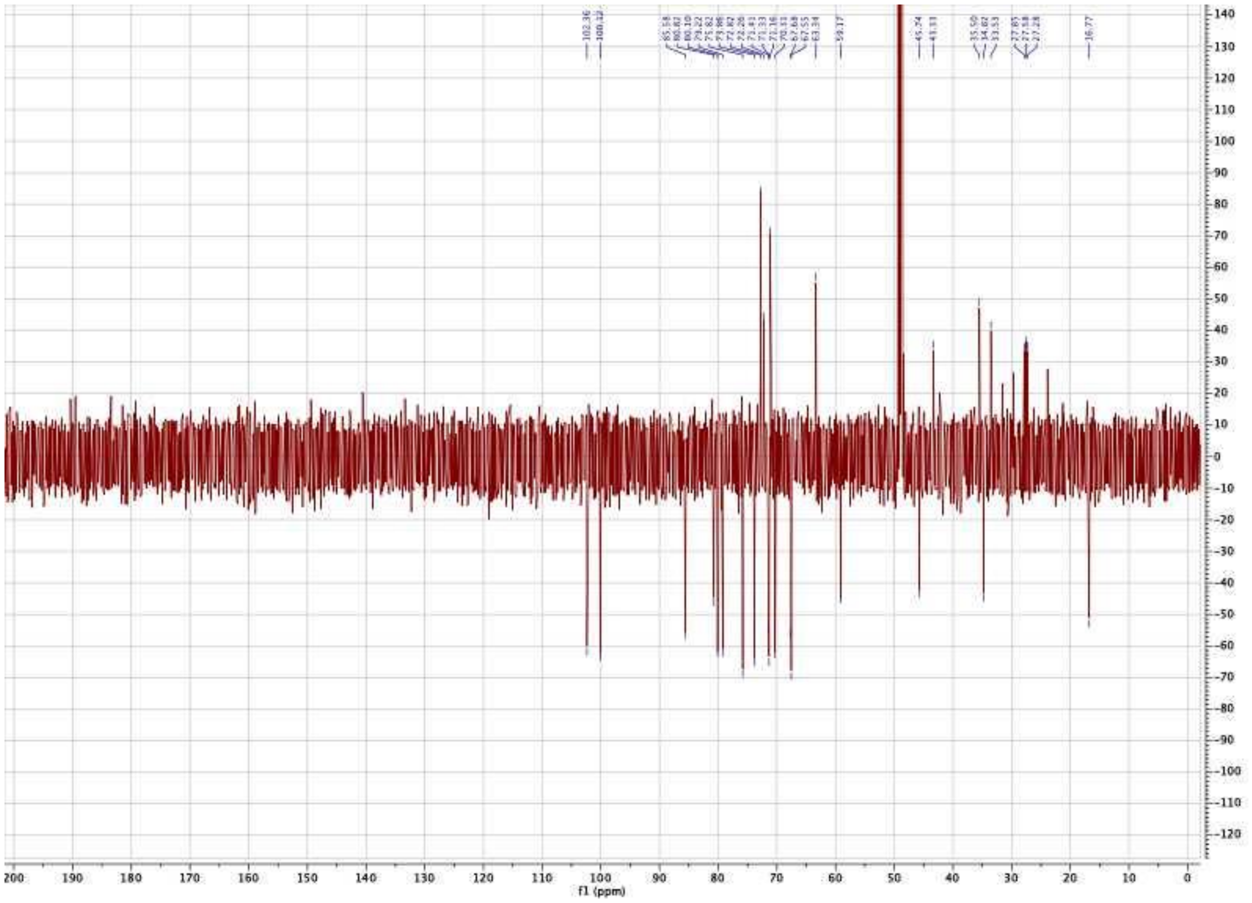
f1 (ppm)

Compound 2m

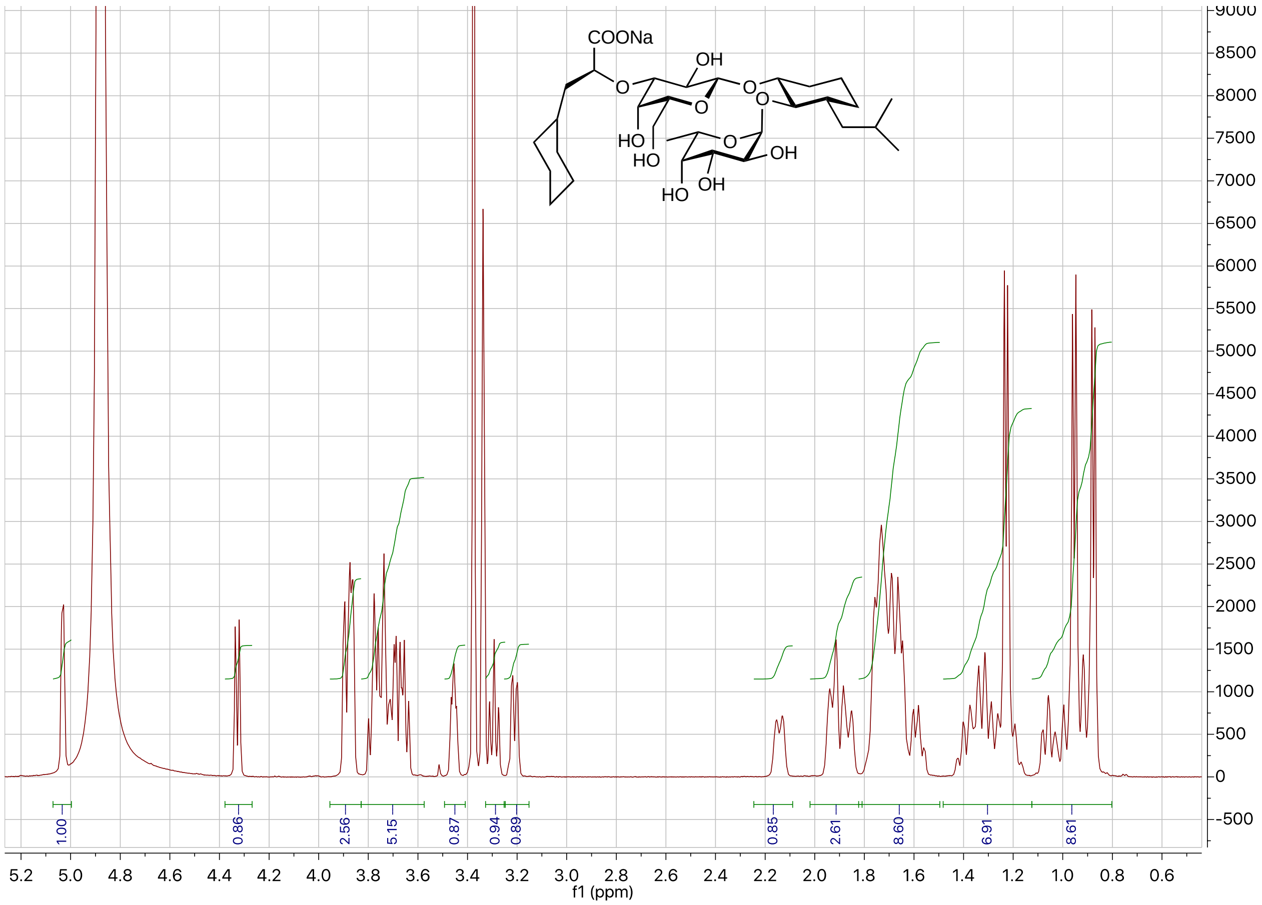
COONa
OH
O
HO
HO
OH
HO
OH
1.00
0.86
2.56
5.15
0.87
0.94
0.89
0.85
2.61
8.60
6.91
8.61
f1 (ppm)

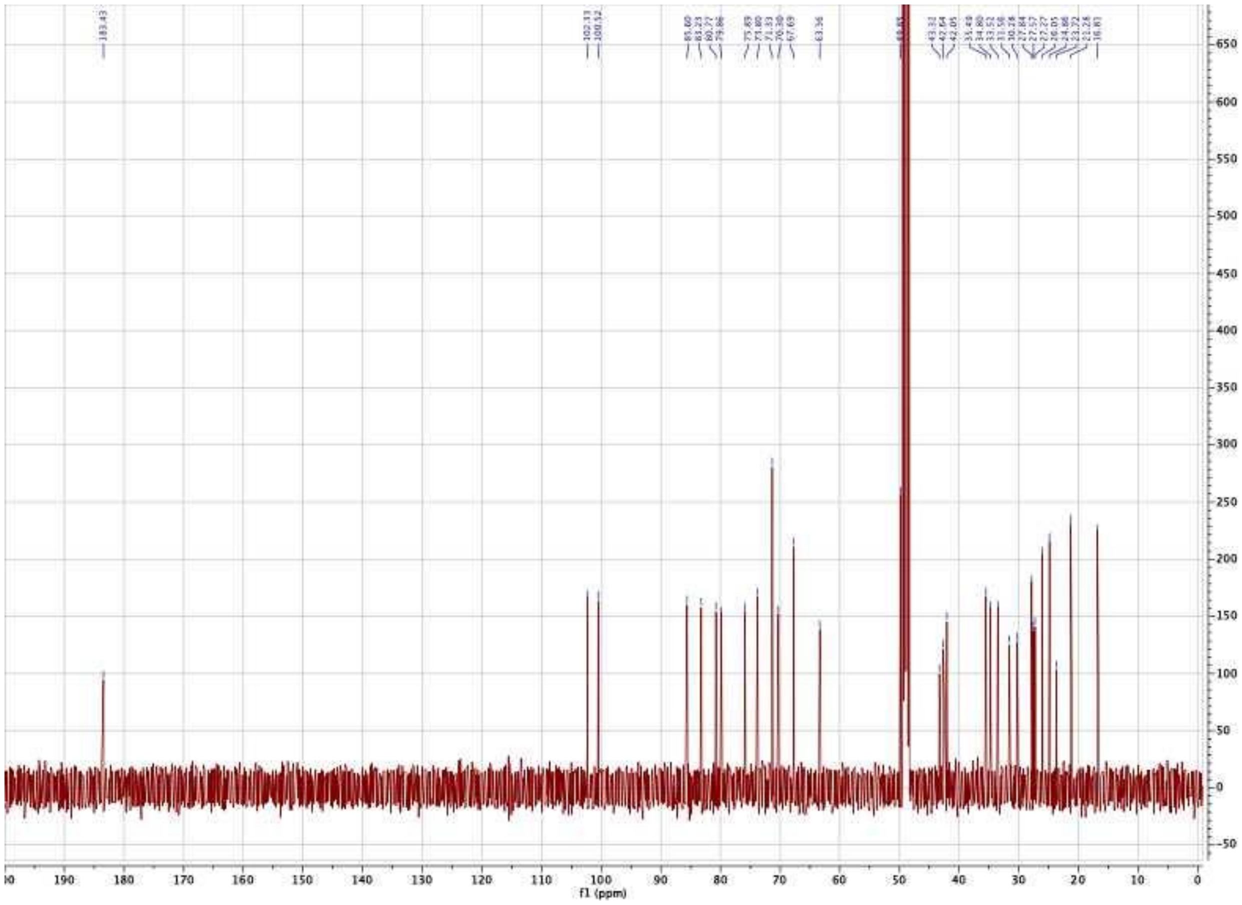
f1 (ppm)

Compound 2n

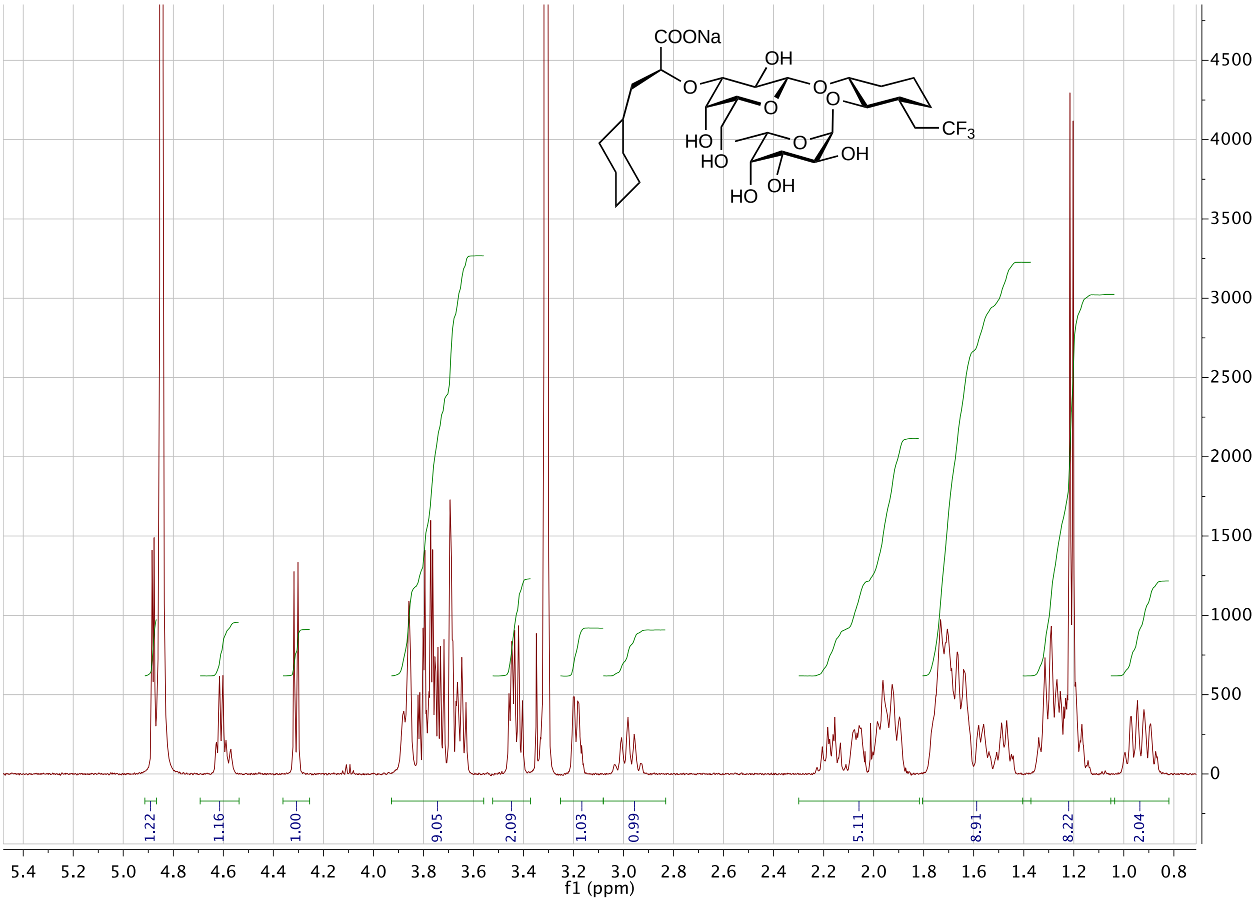
COONa
OH
O
O
O
O
HO
HO
OH
HO
OH
O
CF3
1.22
1.16
1.00
9.05
2.09
1.03
0.99
5.11
8.91
8.22
2.04
f1 (ppm)

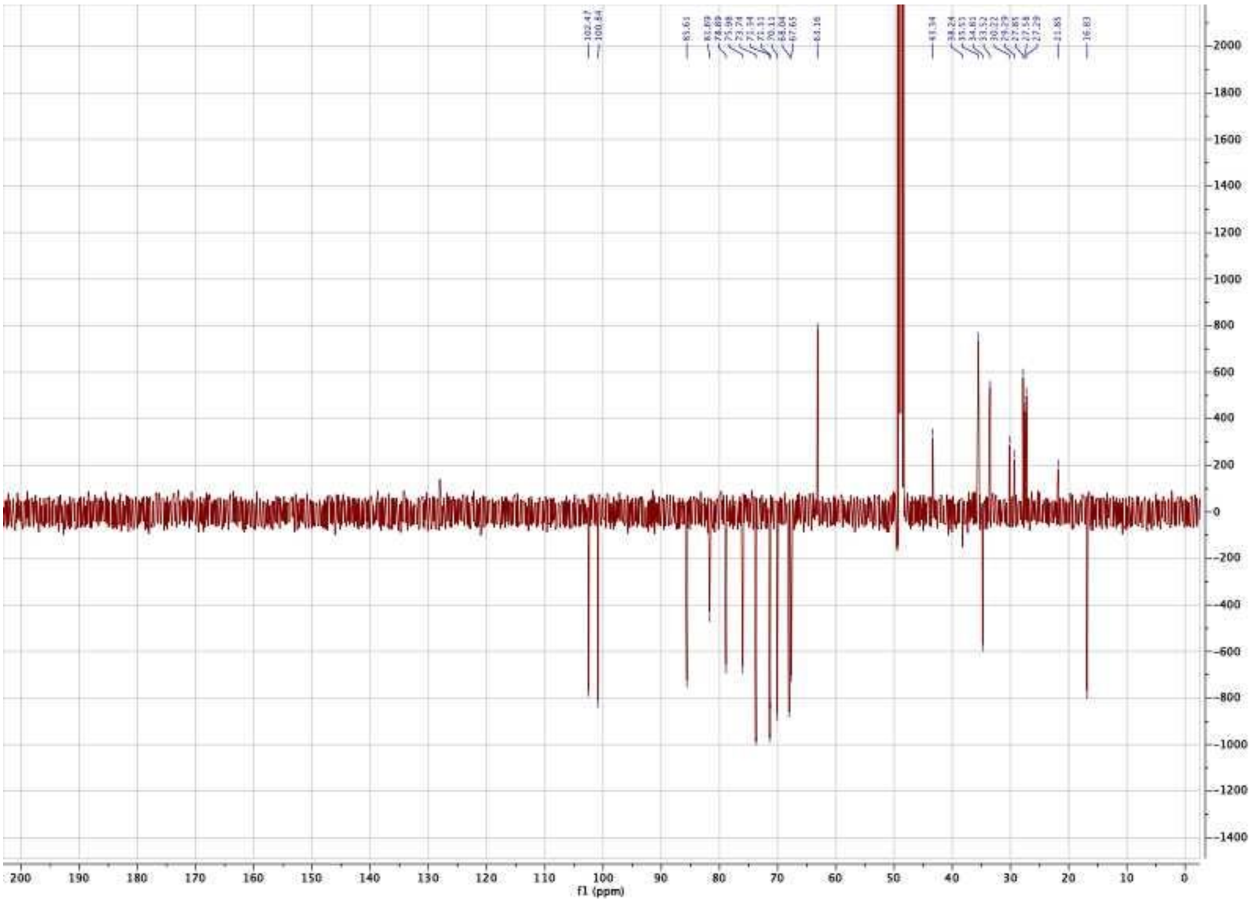
f1 (ppm)

6. HPLC traces

Method: Agilent HPLC 1100/1200 with ELSD. A: H_2O + 0.1% TFA; B: MeCN + 0.1 % TFA; Column: Waters Atlantis T3, 3 µm, 2.1x100 mm; Gradient: 5% B to 95% B over 20 min; flow rate: 0.5 mL/min.

Compound 2d

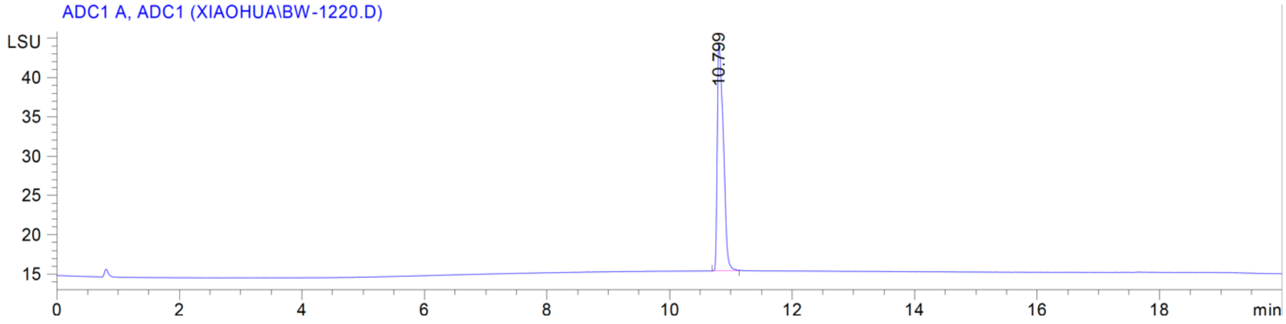

Peak #	RetTime [min]	Type	Width [min]	Area [LSU*s]	Height [LSU]	Area %
1	10.799	BV	0.0876	200.12408	28.95453	100.0000
Totals :				200.12408	28.95453	

Compound 2e

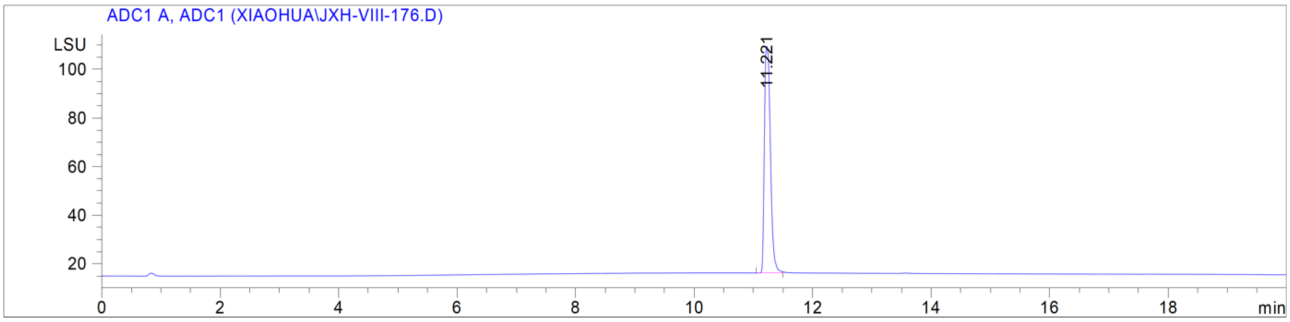

Peak #	RetTime [min]	Type	Width [min]	Area [LSU*s]	Height [LSU]	Area %
1	11.221	VV	0.0932	656.98834	93.29330	100.0000
Totals :				656.98834	93.29330	

Compound 2f

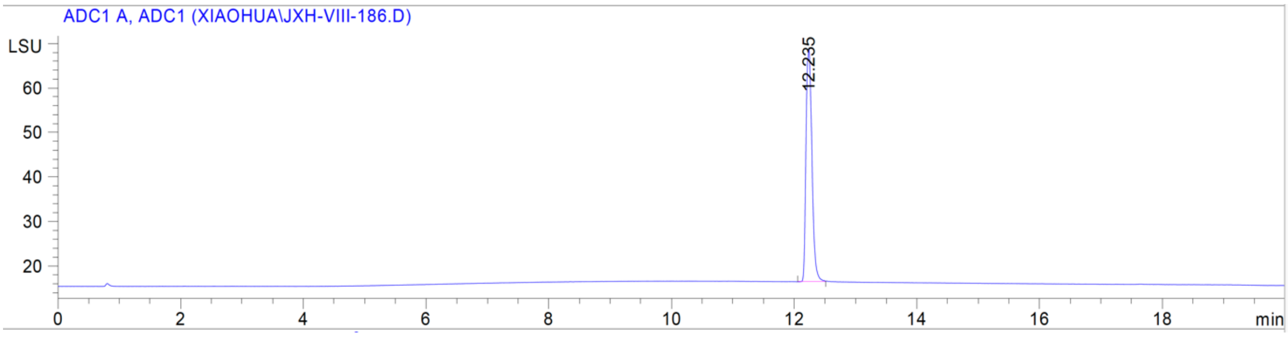

Peak #	RetTime [min]	Type	Width [min]	Area [LSU*s]	Height [LSU]	Area %
1	12.235	BV	0.0851	351.54156	52.53082	100.0000
Totals :				351.54156	52.53082	

Compound 2g

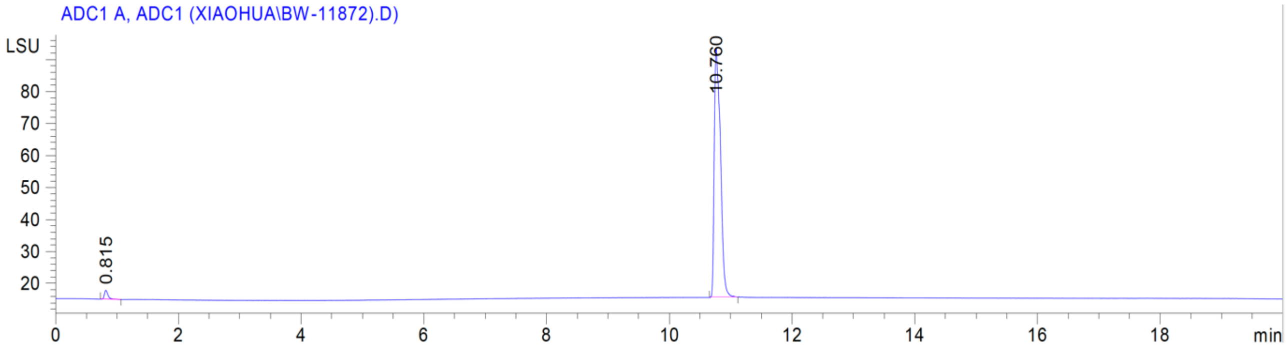

Peak #	RetTime [min]	Type	Width [min]	Area [LSU*s]	Height [LSU]	Area %
1	0.815	BB	0.0618	11.43823	2.79106	2.0505
2	10.760	BV	0.0957	546.38055	78.06431	97.9495
Totals :				557.81878	80.85537	

Compound 2h

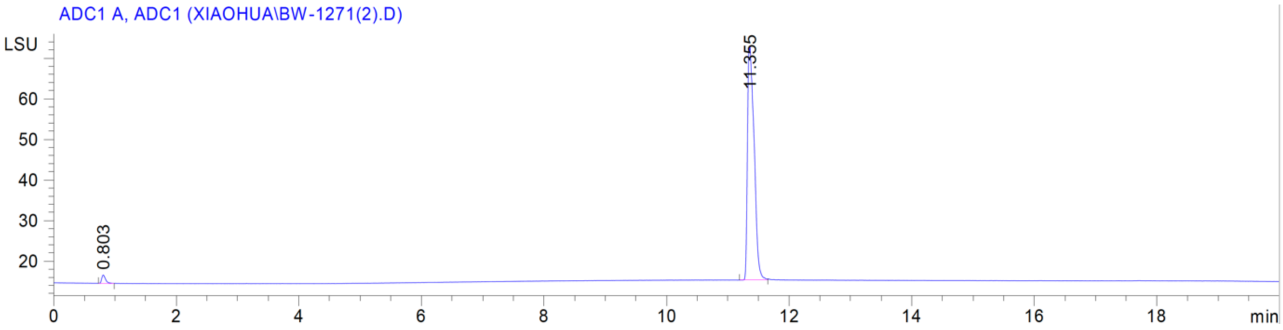

Peak #	RetTime [min]	Type	Width [min]	Area [LSU*s]	Height [LSU]	Area %
1	0.803	BB	0.0634	8.91833	2.06498	2.0121
2	11.355	BV	0.0939	434.31705	57.64245	97.9879
Totals :				443.23538	59.70743	

Compound 2i

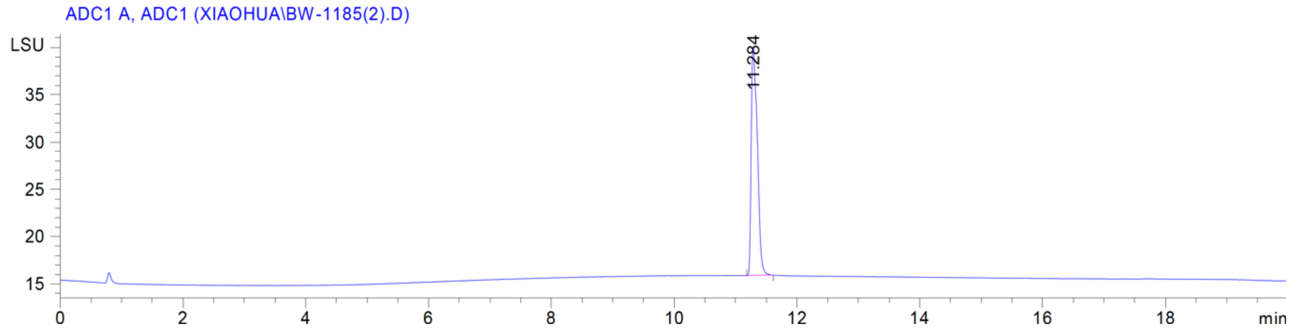

Peak #	RetTime [min]	Type	Width [min]	Area [LSU*s]	Height [LSU]	Area %
1	11.284	BB	0.0959	168.05083	24.23498	100.0000
Totals :				168.05083	24.23498	

Compound 2j

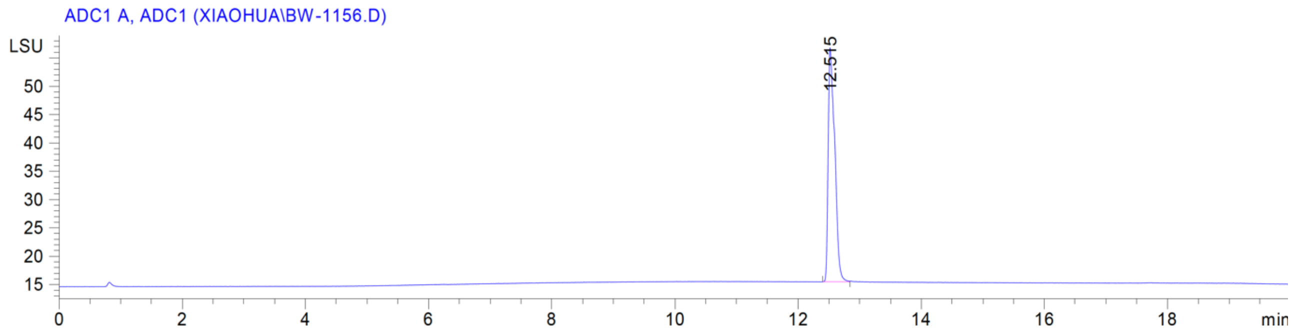

Peak #	RetTime [min]	Type	Width [min]	Area [LSU*s]	Height [LSU]	Area %
1	12.515	BV	0.0994	305.49240	41.22823	100.0000
Totals :				305.49240	41.22823	

Compound 2k

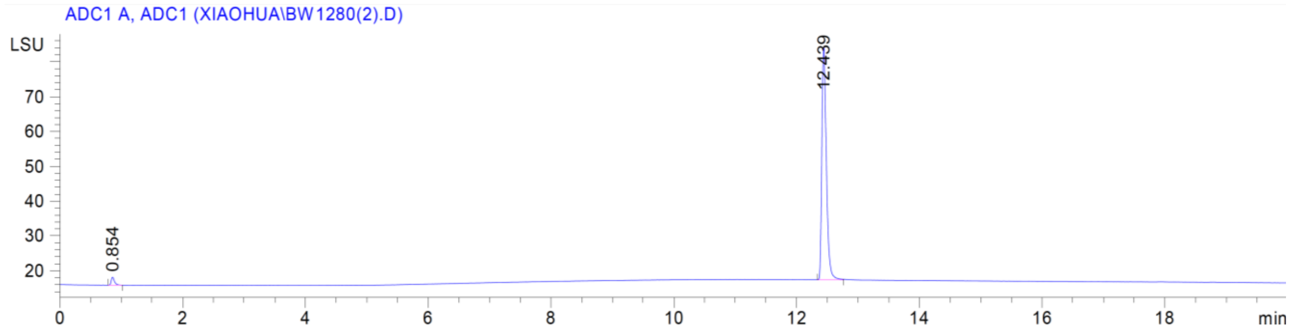

Peak #	RetTime [min]	Type	Width [min]	Area [LSU*s]	Height [LSU]	Area %
1	0.854	BB	0.0567	8.56341	2.28421	2.4318
2	12.439	BV	0.0737	343.57901	67.11153	97.5682
Totals :				352.14242	69.39573	

Compound 2l

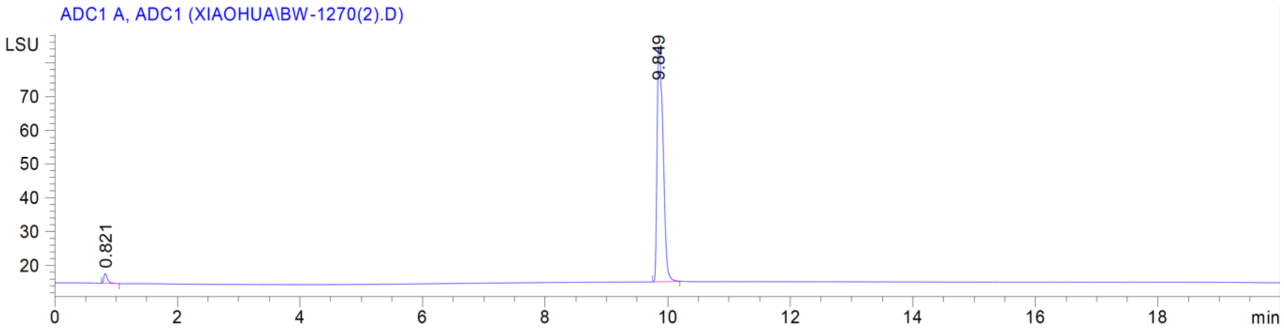

Peak #	RetTime [min]	Type	Width [min]	Area [LSU*s]	Height [LSU]	Area %
1	0.821	BB	0.0603	12.07567	2.91523	2.4663
2	9.849	BV	0.0880	477.55151	69.63211	97.5337
Totals :				489.62719	72.54734	

Compound 2m

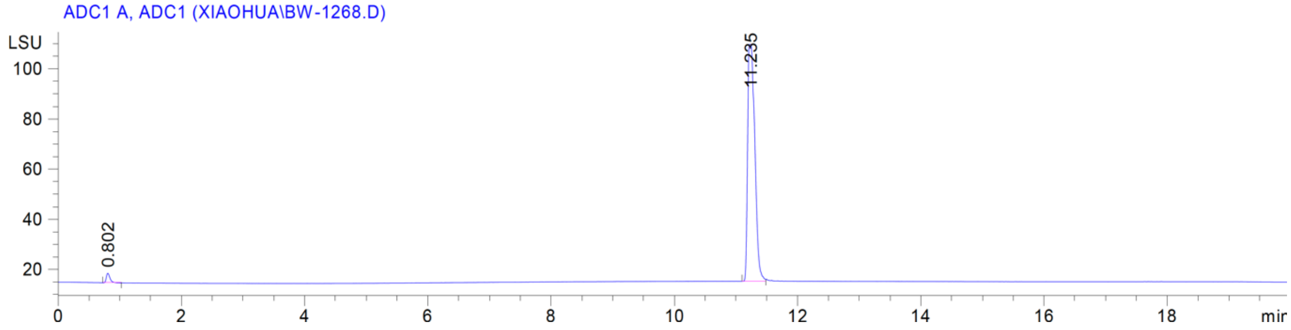

Peak #	RetTime [min]	Type	Width [min]	Area [LSU*s]	Height [LSU]	Area %
1	0.802	BB	0.0640	15.92679	3.79434	2.0415
2	11.235	BV	0.1114	764.22626	94.46172	97.9585
Totals :				780.15305	98.25607	

Compound 2n

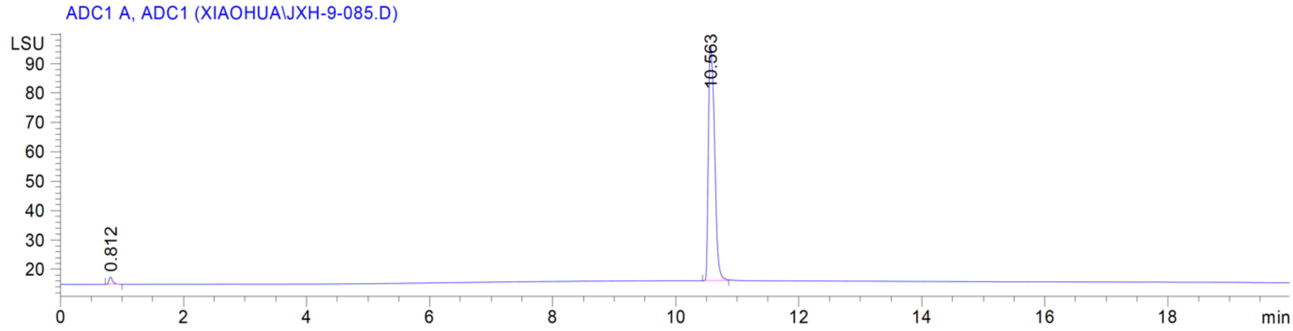

Peak #	RetTime [min]	Type	Width [min]	Area [LSU*s]	Height [LSU]	Area %
1	0.812	BB	0.0636	10.51678	2.47395	1.8390
2	10.563	BV	0.0997	561.36322	80.16837	98.1610
Totals :				571.88000	82.64232	

7. References

1. Barnier, J.-P.; Morisson, V.; Volle, J.; Blanco, L. Chemo-enzymatic preparation of optically active endo-bicyclo [4.1.0]heptan-2-ols. *Tetrahedron Asymmetry* **1999**, *10*, 1107–1117
2. Li, J.; Tan, C.: Gong, J.; Yang, Z. Palladium-catalyzed oxidative rearrangement of tertiary allylic alcohols to enones with oxygen in aqueous solvent. *Org. Lett.* **2014**, *16*, 5370–5373
3. Chen, Q.-Y.; Wu, S-W. Methyl fluorosulphonyldifluoroacetate; a new trifluoromethylating agent. *J. Chem. Soc. Chem. Commun.* **1989**, 705–706
4. Rotticci, D.; Norin, T.; Hult, K. Mass transport limitation reduce the effective stereospecificity in enzyme-catalyzed kinetic resolution. *Org. Lett.* **2000**, *2*, 1373–1376
5. Ter Halle, R.; Bernet, Y.; Billard, S.; Bufferne, C.; Carlier, P.; Delaitre, C.; Flouzat, C.; Humbolt, G.; Laigle, J.C.; Lombard, F.; et al. Development of a practical multikilogram production of (*R*)-seudenol by enzymatic resolution. *Org. Process Res. Dev.* **2004**, *8*, 283–286
6. Sato, S.; Mori, M.; Ito, Y.; Ogawa, T. An efficient approach to *O*-glycosides through $CuBr_2$—Bu_4NBr mediated activation of glycosides. *Carbohydr. Res.* **1986**, *155*, C6–C10.
7. Thoma, G.; Magnani, J.L.; Patton, J.T.; Ernst, B.; Jahnke, W. Preorganization of the bioactive conformation of sialyl Lewis^x analogues correlates with their affinity to E-selectin. *Angew. Chem. Int. Ed.* **2001**, *40*, 1941–1945.
8. Schwizer, D.; Patton, J.T.; Cutting, B.; Smieško, M.; Wagner, B.; Kato, A.; Weckerle, C.; Binder, F.P.; Rabbani, S.; Schwardt, O.; et al. Pre-organization of the core structure of E-selectin antagonists. *Chem. Eur. J.* **2012**, *18*, 1342–1351
9. Cooper, A. Thermodynamics and Interaction. In *RSC Tutorial Chemistry Text No. 16. Biophysical Chemistry*; Humphrey, S., Macpherson, J., Taylor, P., Eds.; The Royal Society of Chemistry: Cambridge, UK, 2004; pp. 109–110.
